# Dye-sensitized solar cells strike back

**DOI:** 10.1039/d0cs01336f

**Published:** 2021-09-30

**Authors:** Ana Belén Muñoz-García, Iacopo Benesperi, Gerrit Boschloo, Javier J. Concepcion, Jared H. Delcamp, Elizabeth A. Gibson, Gerald J. Meyer, Michele Pavone, Henrik Pettersson, Anders Hagfeldt, Marina Freitag

**Affiliations:** Department of Physics “Ettore Pancini”, University of Naples Federico II 80126 Naples Italy; School of Natural and Environmental Science, Newcastle University Bedson Building NE1 7RU Newcastle upon Tyne UK marina.freitag@newcastle.ac.uk; Department of Chemistry, Ångström Laboratory, Uppsala University P.O. Box 523 751 20 Uppsala Sweden anders.hagfeldt@uu.se; Chemistry Division, Brookhaven National Laboratory Upton New York 11973 USA; Department of Chemistry and Biochemistry, University of Mississippi, University MS 38677 USA; Department of Chemistry, University of North Carolina at Chapel Hill Chapel Hill North Carolina 27599 USA; Department of Chemical Sciences, University of Naples Federico II 80126 Naples Italy; Dyenamo AB Teknikringen 38A 114 28 Stockholm Sweden; University Management and Management Council, Vice Chancellor, Uppsala University Segerstedthuset 752 37 Uppsala Sweden

## Abstract

Dye-sensitized solar cells (DSCs) are celebrating their 30th birthday and they are attracting a wealth of research efforts aimed at unleashing their full potential. In recent years, DSCs and dye-sensitized photoelectrochemical cells (DSPECs) have experienced a renaissance as the best technology for several niche applications that take advantage of DSCs' unique combination of properties: at low cost, they are composed of non-toxic materials, are colorful, transparent, and very efficient in low light conditions. This review summarizes the advancements in the field over the last decade, encompassing all aspects of the DSC technology: theoretical studies, characterization techniques, materials, applications as solar cells and as drivers for the synthesis of solar fuels, and commercialization efforts from various companies.

## Abstract 2

Dye-sensitized solar cells (DSCs) are celebrating their 30^th^ birthday and they are attracting a wealth of research efforts aimed at unleashing their full potential. Righteous font designed by Astigmatic and licensed under the Open Font License.

## Graphical abstract

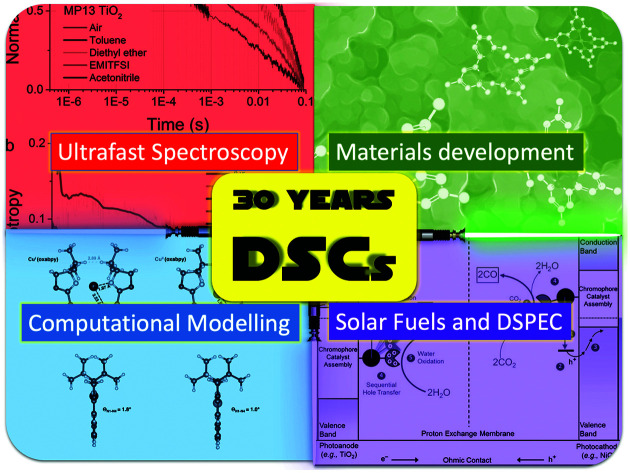

## 1 Introduction

Unprecedented changes in the world's energy production are required to meet with the urgent need to replace fossil fuels to mitigate their effects on climate change, and to keep pace with the ever-increasing global demand for energy. This calls for a rapid shift towards large scale implementation of renewable energy sources, of which sunlight has by far the largest potential. The challenge for scientists is to explore new materials for the creation of devices that can be mass-produced and efficiently convert light energy into electricity or solar fuels at a lower cost with sustainability in mind. Since renewable energy sources currently account for only about 10% of the total energy supply^1^ (29% of the total electricity supply), there is room for a large increase in energy production from solar cells in the near future.

The Sun is the largest source of energy when taking into account both renewable and non-renewable sources, as it supplies the world with 173 000 TW of energy each year.^2^ In other words, more energy from the Sun reaches the Earth in one hour than the human population consumes in a year. Photovoltaic electricity generation has grown at an average rate of more than 34% each year over the last 10 years, making it the world's fastest developing energy technology.^3^ However, photovoltaic cells contribute only 1% of the global energy production. The International Energy Agency (IEA) predicts a 50% increase in renewable electricity production from 2019 to 2025.^4^ This fast rise in the capacity of users to produce their own energy offers new possibilities and problems for utilization on a global level. Distributed solar PV systems in residential and commercial buildings as well as in industries are projected to establish a strong market position, and their installed capacity is estimated to almost double to 320 GW by 2025. The Si-based solar technology is presently that most established in manufacturing. Alternative technologies generally offer comparable efficiency to Si (*e.g.* GaAs or CIGS) in single-junction systems, but they remain expensive owing to manufacturing and material costs. Third-generation photovoltaic devices – hybrid solar cells – use cheap and abundant raw materials with the potential of high efficiencies.^4^

Exactly 30 years ago, in 1991, Michael Grätzel and his research group realized a new kind of solar cell: the dye-sensitized solar cell, DSC, or Grätzel cell.^5^ It is a very promising alternative to classical inorganic p–n junction solar cells as it combines molecular systems and nanoparticles to create a device that mimics photosynthesis, with the objective of turning sunlight into a renewable, reliable, and low-cost source of energy closer to existence. The first demonstration of dye injection into a single crystal semiconductor was provided by Gerischer in 1966,^6,7^ but it was Grätzel's introduction of a mesoporous semiconductor layer that led to the breakthrough in DSC technology. In DSCs, dyes are responsible for light absorption and charge separation and, therefore, for the conversion of photons to electrons. Dyes are bound to mesoporous semiconductors, which are only used to collect the resulting free electrons and transport them to the electrode as current.^8^ Electrons flow back into the system through a charge transport material, which regenerates the dye molecules, thus closing the circuit.^9–11^ DSC devices exhibit impressive energy efficiencies of over 13% under full sun illumination.^12^ Further, they are based on inexpensive starting materials and simple production techniques.^13,14^ Some concern has been raised about the sealing of liquid junction solar cells.^15–18^ Therefore, improvements in sealing strategies or the substitution of the liquid electrolyte with a solid charge transfer material will have a large influence on commercialization.^19–23^

With no clear third generation solar cell technology being dominant for mass production given significant concerns across all technologies, it is expected that DSCs will have years of thriving development ahead toward high efficiency outdoor applications. Additionally, DSCs are exceptional among third generation technologies with regard to specific applications. DSCs can be designed with a high degree of flexibility concerning shape, color, and size, as well as suitability for unique deployment scenarios. DSCs remain a competitive third generation alternative photovoltaic technology for several reasons including: (i) simple preparation methods, which will help to convert solar energy in a sustainable way, (ii) fabrication without the use of toxic materials, and (iii) design flexibility, which allows DSCs to be implemented in many different environments, from transparent smart windows to consumer electronics and indoor applications, which enables the powering of the next digital revolution of widely distributed sensors forming the Internet of Things (IoT).

The research progress during the past ten years in the field of DSCs is marked by important breakthroughs towards their use for a sustainable future. Relentless endeavours made it possible to achieve high efficiencies for DSCs in outdoor and indoor environments. These considerable advances were made by developing new panchromatic rigid-structure dye systems, new redox shuttles and hole transport materials, and by gaining new knowledge about the dyes' and redox shuttles' fundamental behavior. Under full sun illumination (standard AM1.5G), power conversion efficiencies have reached 13% (certified value)^12^ and 14% (non certified) with co-sensitized organic dyes.^24,25^ Under artificial light sources, efficiencies were pushed above 34%.^12,26^ The new redox couples and electrolytes based on cobalt and copper coordination complexes are able to regenerate the dye with less than 0.2 V driving force, which allows for the fabrication of systems with lower thermal losses. Current research and developments are the perquisite to improve efficiencies beyond 20%. Here, this review offers an updated overview of advanced characterization methods and current research trends of this transitioning technology, from the perspectives of device and molecular modelling to state-of-the-art techniques and novel device structures. Every device element, from metal oxides and nanomaterials to new hole transporter materials, dopants, and counter-electrodes, is addressed. Additional applications and constructs are discussed including p-type DSCs, tandem DSCs, and dye-sensitized solar fuel production. Past and current commercialization efforts are also showcased.

### 1.1 Light and energy

All photovoltaic devices, such as solar cells, convert solar radiation into electricity on the basis of the photovoltaic effect, discovered by the French physicist Alexandre Edmond Becquerel.^27^ The photovoltaic effect is linked to the photoelectric one, a phenomenon in which electrons are expelled when light shines on a conducting material. For the explanation of this phenomenon, Albert Einstein received the 1921 Nobel Prize in physics, introducing new quantum principles.^28^ It is described as the appearance of an electric voltage between two electrodes attached to a solid or liquid system when light shines onto it.

**Fig. 1:**
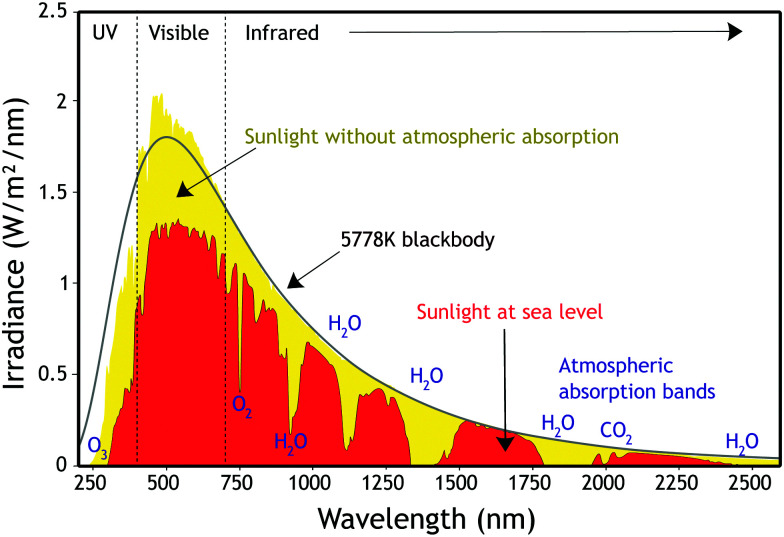
Solar irradiance spectrum. Artwork created by Nick84 and released under Creative Commons BY-SA 3.0 license, ref. 32.

In space, the solar spectrum resembles that of a black body at a temperature of 5778 K and includes a wide range of wavelengths, from X-rays to radio waves, with the main peak in the visible range (see Fig. 1). While travelling through Earths atmosphere, parts of the spectrum are filtered out (*e.g.* X-rays) and the solar spectrum reaching the planet surface is different compared to space. The light path through the atmosphere is defined as air mass (AM).^29^ As the solar spectrum distribution varies during the day and at different locations, a standard reference spectrum was established in order to compare the performance of photovoltaic devices from various manufacturers and research labs. The AM1.5 Global (AM1.5G) spectrum has a combined power intensity of 1000 W m^−2^ (100 mW cm^−2^) and is used as standard for the efficiency measurement of solar cells.^30,31^ The irradiance of sunlight, whose curve is shown in Fig. 1, is defined as the amount of energy of a certain light wavelength shone on a unit area per unit of time, J s^−1^ m^−2^ nm^−1^ (W m^−2^ nm^−1^). This spectral irradiance can be integrated over all wavelengths to obtain the overall irradiance in W m^−2^.

**Fig. 2:**
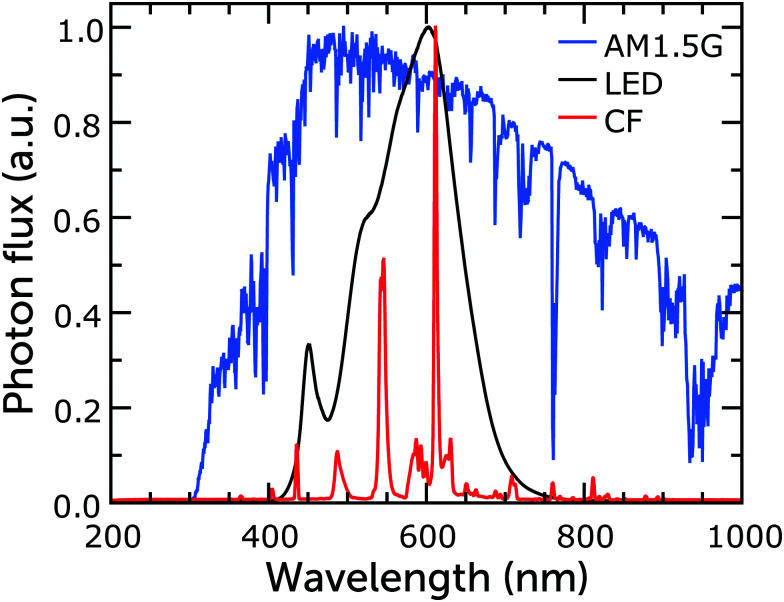
Normalized emission spectra of warm white fluorescent and LED bulbs, and of the AM1.5G standard. Reproduced from ref. 38 with permission from The Royal Society of Chemistry, copyright 2021.

While DSCs perform well under sunlight, since dye light absorption profiles are commonly limited to the visible part of the solar spectrum, they perform even better when illuminated by artificial light sources, whose emission spectrum is similar to the visible range of that of the Sun (Fig. 2).^26,33–37^ Since any indoor light intensity is orders of magnitude smaller than sunlight and the spectra between the different light sources vary considerably, from an experimental point of view indoor lighting conditions are quite different from the solar irradiance outdoors. The intensity of typical indoor lighting has illuminance values ranging from 200 to 1000 lx (lux, which corresponds to lumen per unit area, lm m^−2^). For comparison, AM1.5G light has an illuminance value of about 100 000 lx. Illuminance is similar to irradiance (measured in W m^−2^), but it defines light intensity in terms of human eye perception rather than energy. Illuminance cannot be converted to irradiance *via* a simple mathematical operation and while the latter can be used to quantify solar cell performance directly, the former cannot. At the same illuminance, in fact, different light spectra will produce different irradiance. For example, a light bulb emitting blue light with 1000 lx illuminance will produce more irradiance than a bulb emitting red light with the same illuminance. Only after the lamp spectrum has been determined can the illuminance be obtained from irradiance using eqn (1):1
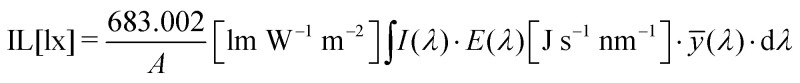
where IL is the illuminance, *I*·*E* is the irradiance (considering the area *A* outside of the integral), given by the product of the light intensity *I* and the photon energy *E*, and *ȳ* is the dimensionless photopic luminosity function of the human eye centered at about 555 nm.

In the case of sunlight measurements there are several guidelines that describe standard experimental conditions, as well as how to test the solar cell, see *e.g.* ASTM standard E948.^39^ For indoor measurements, however, no standard has been defined yet.

### 1.2 Operation principles and structure

The basic components of a dye-sensitized solar cell are the dye-sensitized semiconductor electrode (the working electrode or photoanode), the redox electrolyte and the counter electrode. A monolayer of dye molecules adsorbed on the semiconductor surface is responsible for light absorption in the device. In conventional DSCs, the semiconductor has an n-type character: electrons in the conduction band are responsible for electrical conductivity of the material. Furthermore, the semiconductor has a wide bandgap and does not significantly contribute to solar light absorption. By far, the most applied semiconductor in DSCs is TiO_2_ with the anatase crystal structure, which has a bandgap of ∼3.2 eV and absorbs only UV light. TiO_2_ will be assumed as the semiconductor for the remainder of this part, noting here that a large number of semiconductors can actually be used in DSCs.

A flat and dense TiO_2_ electrode with an adsorbed dye monolayer does not absorb enough light to give practically relevant solar-to-electric conversion efficiencies. In order to harvest a large part of the solar spectrum, TiO_2_ electrodes possessing high-surface areas are used, such as the mesoporous TiO_2_ electrode. This electrode consists of numerous interconnected nanoparticles that are typically about 20–30 nm in size. The porosity of the electrode is about 50% and its surface area can be several hundred times larger than the projected area. As such, the amount of dye adsorbed is also several hundred times larger than for a flat surface. Dye molecules that are chemically bound to the TiO_2_ have the best performances in the DSC. These molecules are also in contact with the redox electrolyte that fills the pores of the mesoporous electrode. The redox mediator transports positive charges to the counter electrode, which is typically located in parallel close to the working electrode.

**Fig. 3:**
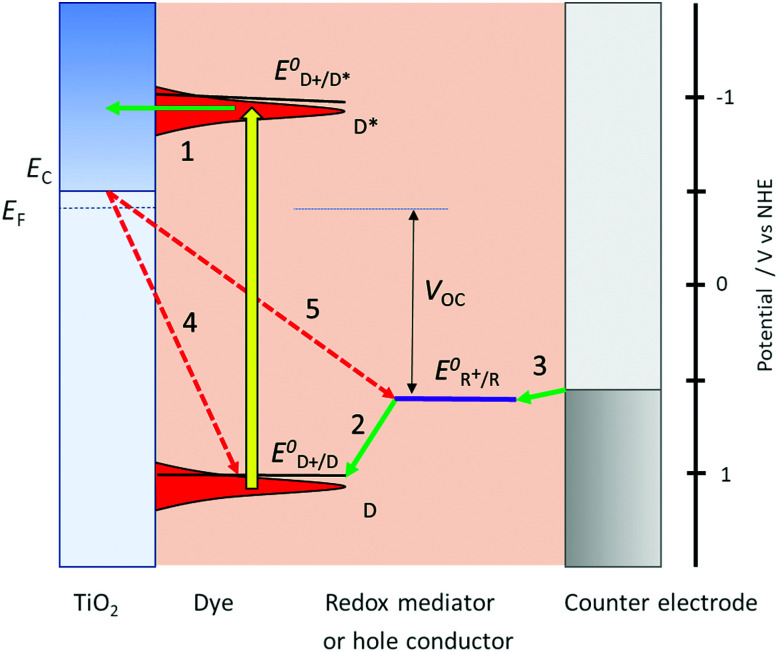
Basic diagram of the dye-sensitized solar cell, displaying working mechanism and energy levels.

Photoinduced electron transfer from a dye molecule to the conduction band of TiO_2_ is the first step in the working mechanism of a dye-sensitized solar cell, see Fig. 3. When light is absorbed by the dye (D), an electron is excited to a higher energy level. The excited dye (D*) can subsequently inject an electron into the conduction band of TiO_2_, which provides a variety of acceptor levels (reaction (1) in Fig. 3). This electron transfer process occurs on the femto- to picosecond time scale.

Electrons in the mesoporous semiconductor are charge compensated by ions in the surrounding electrolyte, and their transport is driven by electronic drift-diffusion. Electrons are collected at the electrode contact on a millisecond time scale under full sunlight illumination. The slow and light-dependent electron transport is generally explained using a multiple trapping model with an exponential trap distribution below the conduction band,^40^ however the nature of the traps is still debated. In recent work, it was found that upon electron accumulation into mesoporous TiO_2_, cations adsorb onto the semiconductor surface.^41^ This could lead to electrostatic traps for the electrons in mesoporous TiO_2_ and account for the observation of similar trap distributions for different types of metal oxides.

The sensitized TiO_2_ is in contact with an electrolyte containing a redox mediator (R^+^/R) that regenerates the dye (*i.e.* reduction of the oxidized dye D^+^, reaction (2) in Fig. 3), and also transfers positive charges from the working to the counter electrode, by means of diffusion of R^+^. At the counter electrode R^+^ is reduced to R (reaction (3)). The dye regeneration process is typically on the microsecond time scale and must be fast enough to prevent recombination of electrons from the semiconductor to the oxidized dye (reaction (4)). Electrons can also recombine with the oxidized form of the redox mediator (reaction (5)).

Fig. 3 also provides the basic energy level diagram of the DSC. The ground-state energy level of the dye is located just below *E*^0^(D^+^/D), the standard reduction potential of the dye, and is often referred to as the HOMO (highest occupied molecular orbital) level. The energy level of the excited dye D* is obtained by adding the absorbed photon energy. The lowest-lying excited state level is obtained by adding *E*_0–0_ (the zero–zero transition energy), which is generally obtained experimentally from the intercept of normalized absorption and fluorescence spectra. This level is often referred to as the LUMO (lowest unoccupied molecular orbital) level.

D* levels should be higher than the conduction band edge *E*_C_ of the semiconductor to ensure sufficient driving force for efficient photoinduced electron injection. Fluorescence of the dye and non-radiative decay processes are competing with the injection reaction. For optimum DSC performance, D* and *E*_C_ should possess sufficient electronic overlap, so that a high quantum yield of injection is obtained, while at the same time *E*_C_ should be as high as possible to obtain a good output voltage in the DSC.

There should also be good matching between the energy levels of dye and redox mediator: sufficient driving force for reduction of the oxidized dye is needed to drive this reaction fast enough to prevent losses through electron/dye recombination. On the other hand, the driving force should not be excessive, as it lowers the voltage output of the DSC.

The voltage output of the DSC is the potential difference between working electrode and counter electrode, see Fig. 3. The potential of the counter electrode is close to that of the redox potential of the electrolyte, and equal to it when no current is flowing, under open-circuit conditions. The potential of the working electrode is equal to the Fermi level of the semiconductor. The Fermi level *E*_F_ is given by:2
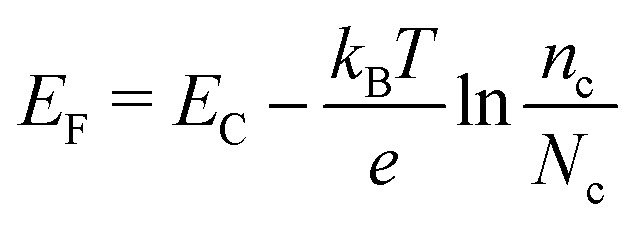
where *k*_B_ is the Boltzmann constant, *T* the absolute temperature, *e* the elementary charge (*k*_B_*T*/*e* is 0.0257 V at room temperature), *n*_c_ is the density of conduction band electrons, and *N*_c_ is the effective density of electronic states at the bottom of the conduction band. *N*_c_ is about 10^20^ cm^−3^ for TiO_2_ anatase. Under solar cell operation, *n*_c_ should as be high as possible to obtain a Fermi level close to the conduction band and a high output voltage. This requires relatively slow electron recombination kinetics.

### 1.3 Device structures

**Fig. 4:**
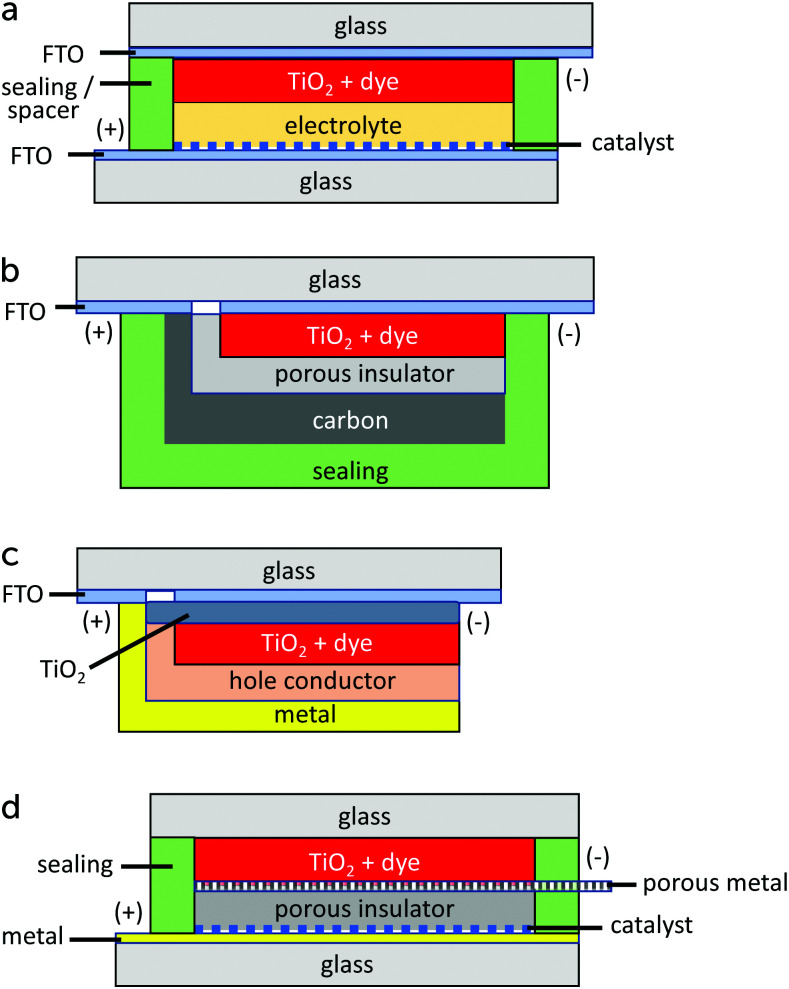
Device structures for dye-sensitized solar cells: (a) sandwich cell, (b) monolithic cell with carbon counter electrode, (c) solid-state DSC (monolithic), and (d) conducting glass-free DSC design.

The standard device structure for the DSC is the sandwich cell, in which both working and counter electrodes are based on conducting glass substrates that are placed face-to-face, with a thin layer of the redox electrolyte in between (Fig. 4a). The distance between the electrodes is usually determined by a thermoplastic frame that also acts as the sealing, and it is typically about 25 μm. An even narrower spacing is favorable, as this decreases the resistance due to redox mediator diffusion in the electrolyte.^42^ Fluorine-doped tin oxide (FTO)-coated glass is most frequently used as conducting glass in DSCs. FTO glass provides a good compromise between high chemical and thermal stability, low sheet resistance and high solar light transmittance. The photoelectrode consists of FTO glass with the mesoporous TiO_2_ film sintered on top. An optional thin and dense TiO_2_ layer (the so-called blocking layer), whose function is to decrease electron recombination from the FTO to the redox electrolyte, can be located between the FTO and the mesoporous TiO_2_. A light-scattering TiO_2_ layer can be added on top of the mesoporous layer to improve light capture in the device. The counter electrode comprises FTO glass with a catalyst, such as Pt nanoparticles, carbon, or a conducting polymer deposited onto of it. The sandwich structure allows for (semi-)transparent solar cell devices and the possibility for illumination from either side, provided that the counter electrode is transparent.

Monolithic DSC structures have advantages over the sandwich structure from a fabrication and cost point of view. Only one FTO glass substrate is used, onto which the different layers are screen-printed: first the mesoporous TiO_2_, then a porous insulating layer and finally a porous carbon layer that acts as counter electrode and electrical conductor (Fig. 4b). The redox electrolyte is infiltrated in all three layers, and a back sealing covers the whole device. This device structure is well suited for scaling up to modules with series or parallel interconnections. The highest reported efficiency for a monolithic DSCs with carbon counter electrodes is 7.6%.^43^ The carbon electrode in the monolithic DSC can be replaced by other conductors. For instance, highly-doped PEDOT films have been used in combination with a porous polyethylene separator film, reaching an efficiency of 7.7%, while also allowing for flexible devices.^44^ Recently, a Ni metal foil with Cr coating and Pt catalyst was implemented instead of the carbon electrode, and an efficiency of 8.0% was achieved.^45^

In a solid-state DSC, the liquid redox electrolyte is replaced with a solid hole transporting material (HTM). It is also commonly a monolithic structure, see Fig. 4c.^46^ A critical step in the fabrication is the infiltration of the hole conductor into the mesoporous TiO_2_ layer. Solution-based methods do not result in complete pore filling.^22^ Furthermore, a thin capping HTM layer is needed, onto which the metal contact is evaporated.

It is possible to avoid FTO-coated glass altogether in DSC structures. Several types of back-contact DSC devices have been developed, where the mesoporous TiO_2_ film is contacted at the back with a porous metal film^47^ or a metal mesh.^48^ A suitable metal is titanium, which forms a passivating oxide layer. Alternatively, a stainless steel mesh can be used if it is coated with a thin passivating layer. The counter electrode can also be Ti metal, but it should then be provided with a suitable catalyst. A possible layout of a DSC avoiding conducting glass is shown in Fig. 4d. The advantages of such a DSC are a higher solar light transmittance of the top glass, and a very low sheet resistance of the working and counter electrodes, allowing for much larger area solar cells.

## 2 Characterization

### 2.1 Power conversion efficiency and *J–V* characteristics

The efficiency of a solar cell is its most important performance parameter. We will refer to it as the power conversion efficiency (PCE), in order to clearly distinguish it from quantum efficiencies. The PCE is usually obtained from the current density (current per unit area, *J*) *vs.* potential (*V*) characteristics of the solar cell, recorded under illumination by a solar simulator. The standard measurement condition is illumination with 100 mW cm^−2^ light with AM1.5G spectral distribution, while the cell is kept at 25 °C.^39^

*J–V* curves are recorded using a source meter or a potentiostat that can apply a controlled potential to the device and measure the current. Typically, *J–V* curves are recorded using voltage steps of 5 or 10 mV. After each voltage step some delay time should be applied (more than 100 ms) before the current measurement is done, in order to allow for the current to reach a stable value.^49^ If the chosen delay time is too short, *J–V* curves recorded in the forward and reverse direction are not identical: hysteresis is observed. While hysteresis in *J–V* curves has been widely discussed in the field of perovskite solar cells, it has not attracted much attention in the DSC field. The origin of hysteresis in DSC is attributed to: (i) capacitive currents, caused by (dis)charging of the mesoporous electrode after the potential step,^50^ and (ii) mass transport in the electrolyte and resulting concentration gradients in the redox couple concentrations.^51^ Hysteresis becomes very apparent in DSCs with practical electrolytes that are more viscous than the volatile acetonitrile-based electrolytes that are used for record devices.

**Fig. 5:**
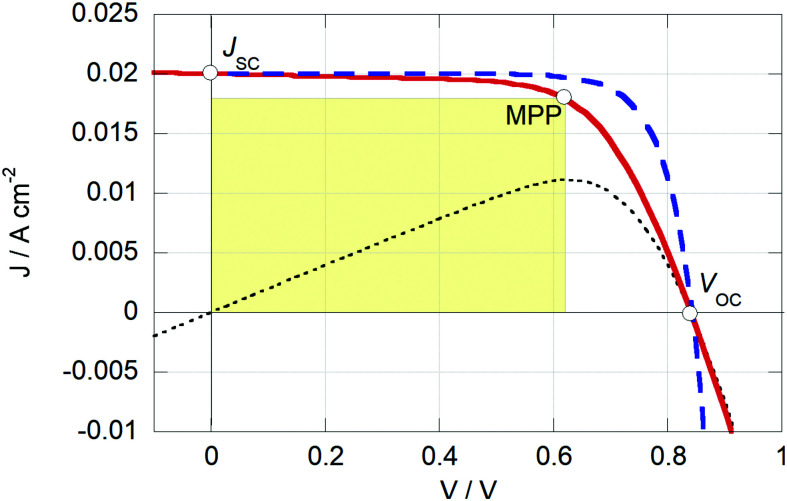
Simulated *J–V* curves of a solar cell using the Shockley diode model with (red line) and without (blue stripes) series and parallel resistance losses. *R*_s_ and *R*_p_ are 5 and 1000 Ω cm^2^, respectively; *J*_s_ = 1.5 nA cm^−2^; *n* = 2. The resistance losses reduce the PCE from 13.1% to 11.2%, due of the reduced fill factor (from 78% to 66%). The black dotted line the is the device's power output with resistance losses. The yellow square represents the device's power output.

From the *J–V* curve several parameters can be determined: *J*_SC_, the current density at zero applied potential; and *V*_OC_, the open-circuit potential, which is the potential found at zero current. At the maximum power point (MPP) the power output of the device (which is the product of *J* and *V*) reaches a maximum, *P*_MPP_, see Fig. 5. The fill factor (FF) is the ratio between *P*_MPP_ and the product of *V*_OC_ and *J*_SC_. A high value of the FF (closer to 1) gives a more square-looking curve and indicates the ability of the solar cell to deliver current and potential at the same time. The PCE is given by eqn (3), where *P*_light_ is the power density of the incoming light.3
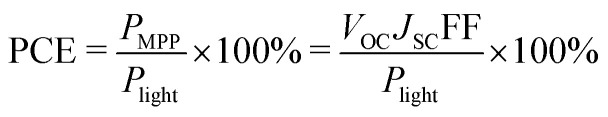


In order to correctly calculate the PCE, the active area of the solar cell device needs to be determined accurately. The most reliable method used in the DSC field is to place a black metal mask with an aperture – the area of which is used for the PCE calculation – directly on top of the solar cell. Also, any light entering from the sides should be blocked. This ensures that no light from outside the aperture area is channeled into the solar cell. The aperture area should be either similar to, or smaller than the DSC working electrode.^52^ If a small aperture is used, part of the DSC is not illuminated. This, however, does not affect the measured PCE much since the non-illuminated areas of the DSC do not contribute much to recombination current in most cases. It is useful to record the *J–V* curve in the dark as well for further analysis of the solar cell, which should not use the aperture area, but instead the measured working electrode area for correct analysis.

**Fig. 6:**
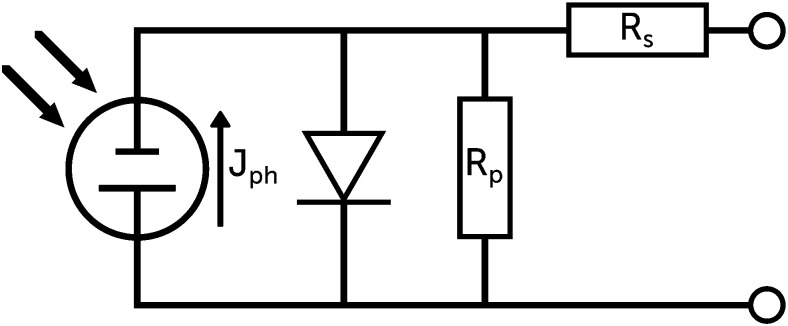
Representation of a solar cell as a schematic circuit.

The general shape of the *J–V* curve of a DSC is well-described by the Shockley diode equation with additional resistive losses, see eqn (4),4
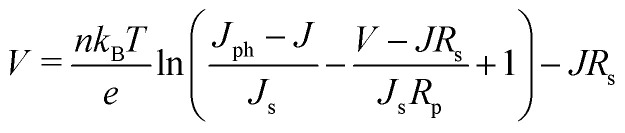
where *n* is the diode quality factor, *k*_B_ the Boltzmann constant, *T* the absolute temperature, *J*_ph_ the generated photocurrent density, *J*_s_ the reverse bias saturation current density, and *R*_s_ and *R*_p_ the series and parallel (or shunt) resistances (units: Ω cm^2^), respectively, see circuit in Fig. 6 and eqn (4). The series resistance originates from the resistance of the conducting glass, the charge transfer resistance at the counter electrode and the resistance due to diffusion of the redox mediator in the electrolyte. The parallel resistance can originate from physical contact between the working and counter electrodes, but it can also describe part of the electron recombination, which is not described by the diode.

MPP tracking is an alternative method to obtain the PCE of a solar cell. The perturb-and-observe method is frequently applied where a step-wise change in potential is made and it is checked whether the product of *J* and *V* increases or decreases; then, depending on the outcome, the next step is made in either the positive of negative potential direction. MPP tracking is a useful method to prove that the DSC is a stable and regenerative system.

### 2.2 *J–V* characterization in ambient light conditions

Although the practicalities of solar cell measurement in ambient light (indoor) conditions are the same as those described above for sunlight simulation, the interpretation of the results is more complex. A brief overview of the challenges and best practices for reporting ambient light *J–V* measurements is provided here, while a more detailed discussion can be found elsewhere.^38,53^

As detailed in eqn (3), PCE is a function of the power provided by the light source, *P*_light_. In the case of sunlight there is a unique source of light, with well-known characteristics and a constant, standardized value of *P*_light_. Indoor, on the other hand, there is a great variety of different light sources. This leads to the conclusion that, while in simulated sunlight measurements the reported PCE value of a solar cell can always be translated to the device's absolute power output *via* a simple mathematical operation, the same does not apply to ambient light measurements. In the latter case, in fact, *P*_light_ is unknown, and it is the experimentalist's responsibility to measure it accurately for the light source in use. Therefore, when performing and reporting about indoor *J–V* measurements: (i) extra care should be taken in the determination of *P*_light_ for the correct computation of the PCE value, (ii) the make and model of the light source should always be specified, together with its emission spectrum, and (iii) the *P*_MPP_ value should always be reported alongside the PCE value. This last point is particularly important to facilitate the comparison of results from different laboratories, because a given solar cell configuration may have a very similar *P*_MPP_ output when illuminated by different light sources, but very different PCE values depending on the overlap between the device absorption and the light source emission spectra.

During practical experiments, in the case of sunlight, the adjustment of the light intensity to the desired value is easily achieved through the use of a reference cell calibrated by a certification authority. However, there cannot be a calibrated reference cell in the case of indoor measurements, unless every laboratory in the world agrees to use the same light bulb. Light intensity determination in ambient light experiments is usually carried out with the use of a lux meter, which provides a value of the illuminance at the measuring spot. However, lux meters are generally bulky tools, and their correct placement inside the testing equipment could be cumbersome. This difficulty arises from one more hurdle that ambient light measurements must overcome compared to simulated sunlight experiments: In the latter case, the intensity of the light source is about two orders of magnitude higher than that present in a common laboratory room. As such, the testing equipment can be easily placed on an open laboratory bench and the eventual contribution to the device photocurrent of the light present in the room will be negligible. In the former case, however, the intensity of the light source is of the same order of magnitude of that present in the laboratory room. Therefore, the testing equipment must be properly encased, so that it is completely isolated from the laboratory environment.

### 2.3 Incident photon-to-current conversion efficiency (IPCE)

In an IPCE measurement, monochromatic light – typically generated by passing white light through a monochromator – falls onto the solar cell and the short-circuit photocurrent is recorded as a function of the light's wavelength. The IPCE is calculated using eqn (5) and is normally plotted as a function of wavelength, yielding a spectrum that is sometimes referred to as the photocurrent action spectrum.5
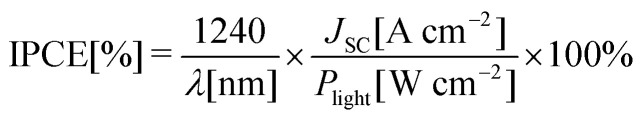


In the equation, *λ* and *P*_light_ are the wavelength and the power density of incident light, respectively. IPCE can be measured using DC or AC methods. In the DC method, only monochromatic light is used, while in the AC method chopped monochromatic light is applied, and a constant white light can be added. The AC photocurrent response is measured using a lock-in amplifier. The two methods should yield the same result, provided that the photocurrent scales linearly with light intensity and that the chopping frequency in the AC mode is sufficiently low.

Integration of the IPCE spectrum with respect to the AM1.5G flux (*ϕ*_AM1.5G_) gives a calculated value of the *J*_SC,IPCE_ (eqn (6)):6
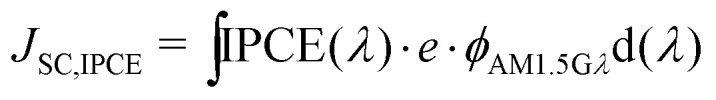


A good match between *J*_SC,IPCE_ and *J*_SC_ measured using a solar simulator gives added confidence in the validity of IPCE and *J*_SC_ measurements. Significant differences can point to calibration errors of the systems.

### 2.4 Impedance spectroscopy

Small-modulation techniques are particularly useful to study complex systems like the DSC. We can distinguish between electrical modulation techniques, such as electrochemical impedance spectroscopy, and optical modulation techniques, such as transient photovoltage (TPV), discussed below.

Electrochemical impedance spectroscopy (EIS) is a widely used general technique in science and technology. A small sinusoidal potential modulation with an amplitude of about 10 mV is superimposed onto a base potential, and the amplitude and phase-shifts of resulting sinusoidal current changes are measured. This is repeated for a large series of frequencies – for DSC typically in the 10^5^–10^−1^ Hz range – to obtain a complete EIS spectrum. The impedance is given by *z* = d*V*/d*I* and is often represented as a complex number: *z* = *z*′+ *jz*′′, where *j* is 
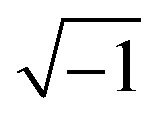
, *z*′ is the real part of the impedance, and *z*′′ the imaginary part, which is phase-shifted by 90°. The real part of the impedance reflects resistance, while the imaginary part originates from capacitance and inductance. For a resistor the impedance is independent of frequency, *z* = *R*, while for a capacitor *z* = −(*jωC*)^−1^, where *C* is the capacitance and *ω* the angular frequency. An equivalent circuit, consisting of electrical elements *R*, *C*, *L* (inductance), CPE (constant phase element, a non-ideal capacitor), and *Z*_d_ (diffusion impedance or Warburg element) is used to fit the experimental EIS spectrum.

**Fig. 7:**
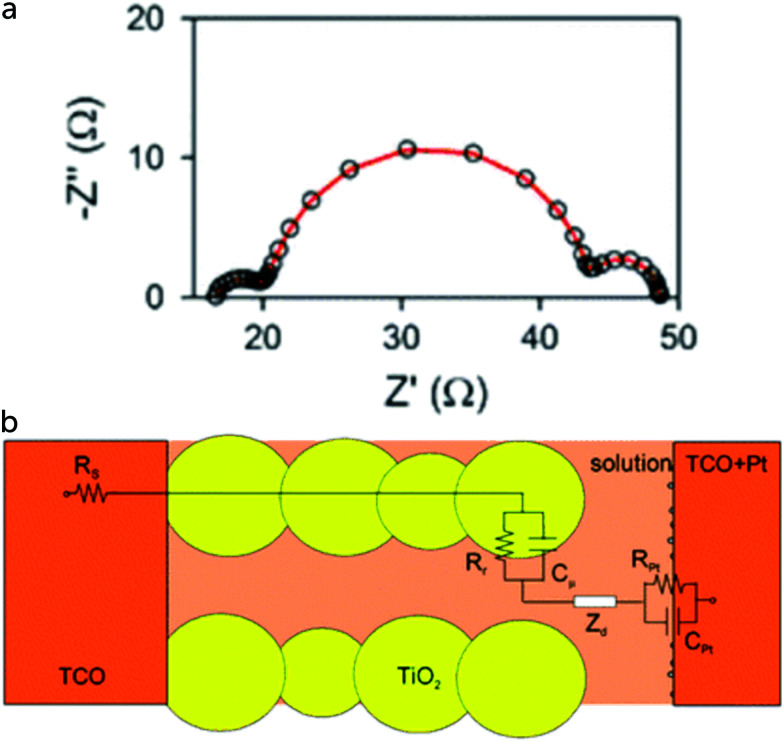
(a) Impedance spectrum (Nyquist plot) of a dye-sensitized solar cell under illumination, recorded at *V*_OC_. (b) Schematic model to fit the EIS under these conditions. Adapted from ref. 54 with permission from the PCCP Owner Societies, copyright 2011.

A convenient EIS analysis of DSC is done under illumination at open-circuit conditions. An example is shown in Fig. 7,^54^ where 3 semicircles can be found, corresponding to three processes in the DSC with significantly differing time constants. The left-hand semicircle, at higher frequencies, is due to the charge transfer resistance at the counter electrode (*R*_CE_) and to the double layer capacitance at the counter electrode/electrolyte interface (*C*_CE_), giving a time constant *τ*_CE_ = *R*_CE_·*C*_CE_. At intermediate frequencies, the recombination resistance at the mesoporous TiO_2_/electrolyte interface, *R*_rec_, and the capacitance of the mesoporous TiO_2_, *C*_TiO2_, form the second semicircle. The electron lifetime in TiO_2_, *τ*_e_, is given by *τ*_e_ = *R*_rec_·*C*_TiO2_. At the lowest frequencies, the impedance due to diffusion of the redox mediator in the electrolyte, *Z*_d_, forms the third semicircle. *Z*_d_ is given by *Z*_d_ = *R*_d_ ·(*jω*/*ω*_d_)^−1^ tanh(*jω*/*ω*_d_), where *R*_d_ is the diffusion resistance and *ω*_d_ is *D*/*L*^2^, with *D* the diffusion coefficient and *L* the effective electrolyte layer thickness.^55^ The high frequency intercept at the *Z*′ axis is the series resistance caused largely by the conducting glass *R*_TCO_.

An EIS measurement in the dark at the same applied potential would yield different results: there is for instance no electron recombination to oxidized dye molecules. Furthermore, there could be a rather large current flow in the device, which leads to potential drops and a less well-defined Fermi level in the mesoporous TiO_2_. The local concentrations of the redox mediator in the device will also be different. However, the advantage of a dark EIS measurement is that it allows for the direct probing of the sensitizer influence on recombination resistance from electrons in TiO_2_ transferring to the redox shuttle in the absence of increased electrode heat and without competing processes such as recombination to the dye.^56^

### 2.5 Opto-electrical transient techniques

Opto-electrical transient measurements and charge extraction methods provide a very useful tool for understanding processes occurring in dye-sensitized solar cells. Detailed description and analysis of such techniques can be found elsewhere.^57,58^ Opto-electrical transient techniques include photocurrent/voltage transients, that can be performed either as small or large modulation techniques.

**Fig. 8:**
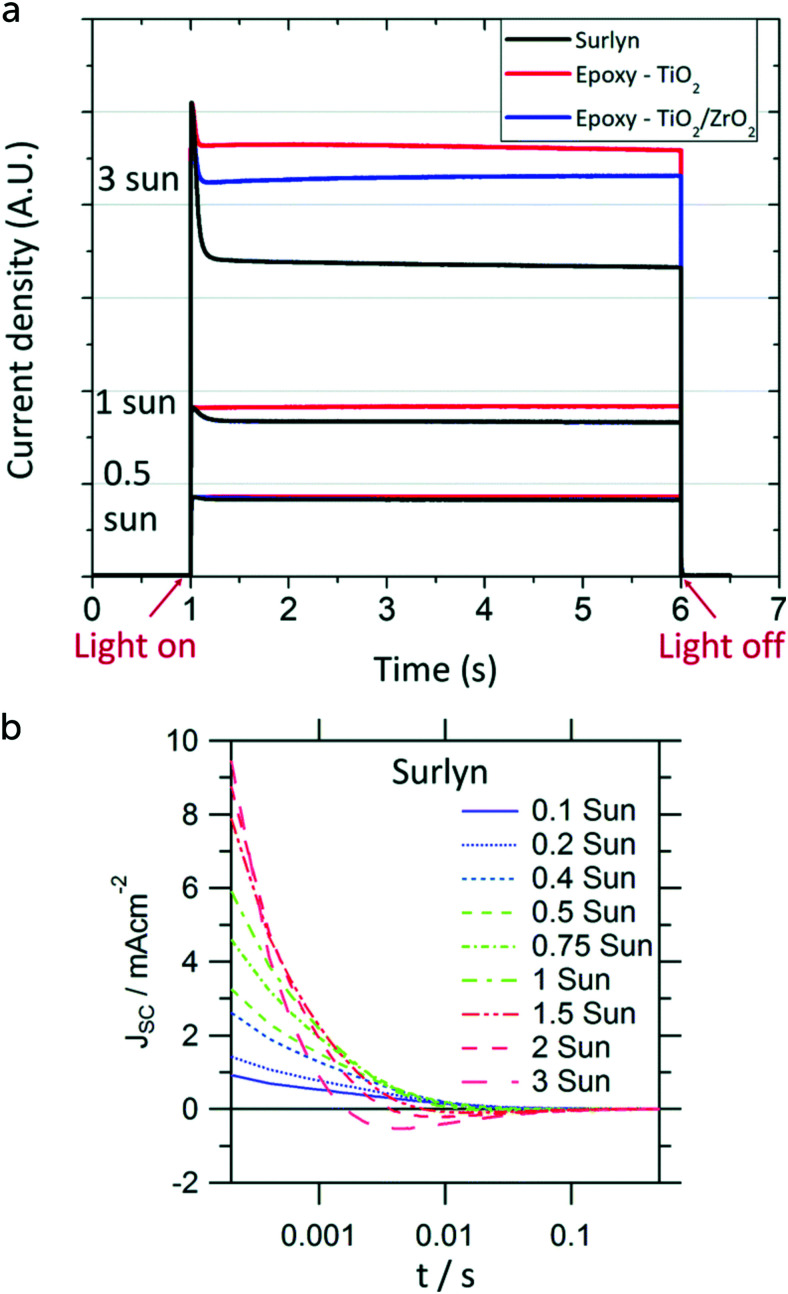
Photocurrent transients of a DSC with a Cu complex-based electrolyte. (a) Under high light intensities and with a relatively thick electrolyte layer (Surlyn: 30 μm) a clear spike is found in the photocurrent onset transient. (b) After switching the light off, a reversal of current can be found in the photocurrent decay transient, due to accumulation of oxidized redox species in the mesoporous electrode, which are reduced by electrons in the TiO_2_. Adapted from ref. 59 with permission from the PCCP Owner Societies, copyright 2017.

Light off/on modulation is easy to perform experimentally and can give useful information. Short-circuit photocurrent transients can provide evidence for accumulation or depletion of the redox mediator in different parts of the DSC. For instance, if the concentration of oxidized redox mediator is too low at the counter electrode, a high value of *J*_SC_ cannot be maintained and electrons in TiO_2_ will have to recombine with the oxidized dye or redox mediator. Such a situation can occur in viscous electrolytes when the oxidized form of the mediator is present in too low concentration, see Fig. 8.^59^

Charge extraction methods provide information about the accumulated electrons in the mesoporous TiO_2_ electrode as a function of potential and/or light intensity. During the extraction, part of the accumulated electrons may recombine before being collected. The extracted charge should therefore be considered as a lower limit of the actual accumulated charge. Integration of the photocurrent decay transient over time gives a good measure of the accumulated charge in mesoporous TiO_2_ electrodes under short-circuit illumination conditions. To obtain the charge under open-circuit illumination conditions, a double switch is needed: light is switched off and simultaneously the cell is switched from open-circuit to short-circuit conditions. Plotting the extracted charge as a function of the *V*_OC_ gives a useful trend that can be used to assess band-edge changes, for instance as a function of the sensitizer or of additives to the electrolyte.

**Fig. 9:**
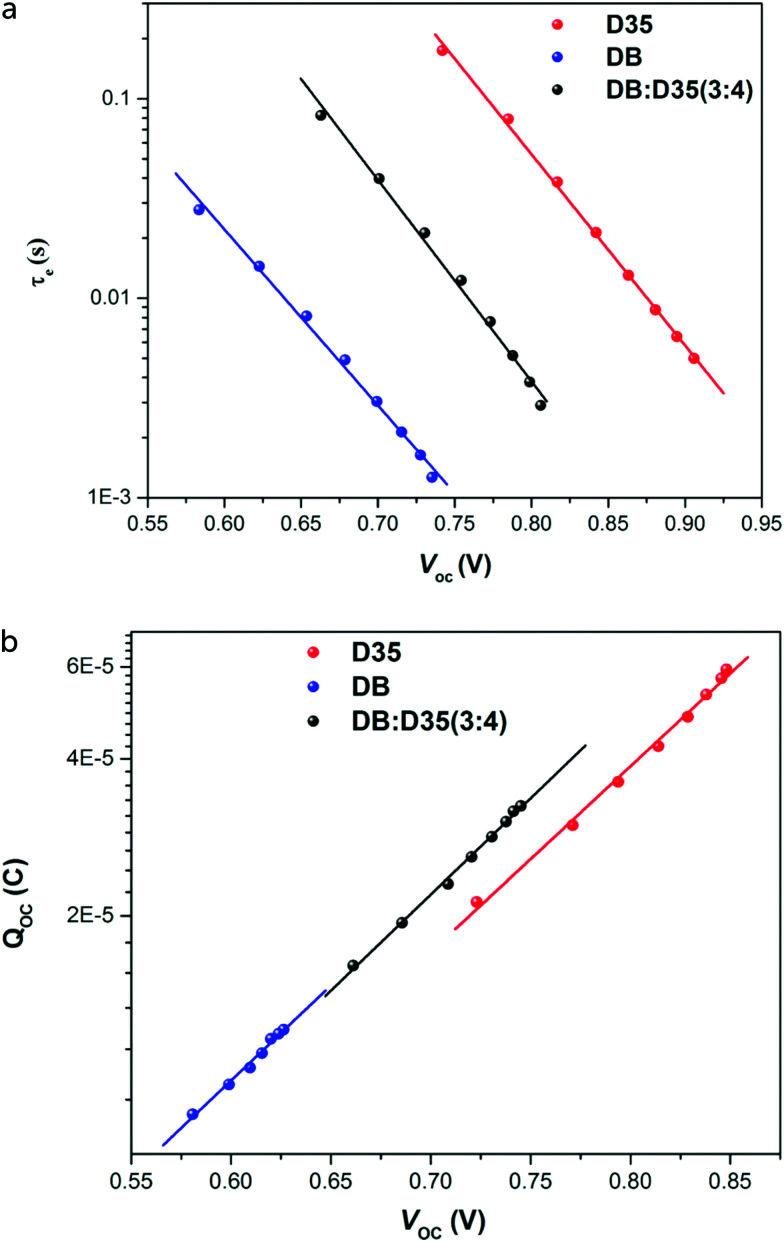
(a) Electron lifetime and (b) accumulated charge as a function of *V*_OC_ for DSCs with a cobalt-based electrolyte, sensitized with D35, Dyenamo blue (DB), or both. Band-edge shifts of the different dyes are small, however a large difference in electron lifetime is found. Adapted with permission from ref. 60. Copyright 2016 American Chemical Society.

Small optical modulation techniques, namely transient photocurrent (TPC) and photovoltage (TPV), provide information on electron transport in the mesoporous TiO_2_ and electron recombination, respectively. The modulation can be in the form of a sine wave: the technique is then called IMPS or IMVS (intensity-modulated photocurrent or voltage spectroscopy, respectively), and multiple frequencies are analyzed. Alternatively, the modulation is in the form of a small pulse or of a step, and the response is recorded in the time domain. Similar information can be obtained from EIS measurements, but TPC and TPV in the time domain have the advantage of being a rapid measurement that can be analyzed quickly, since the photocurrent or photovoltage response to a small light modulation has a simple exponential form, where the time constant is the electron transport time (provided that no significant recombination takes place) for photocurrent transients, or the electron lifetime *τ*_e_ for photovoltage transients. Fig. 9 gives an example of charge extraction and photovoltage transient results for different dyes used in co-sensitized DSC devices.^60^

### 2.6 Spectroscopy

An important attribute of the mesoporous anatase thin films introduced by Grätzel and O'Regan is that they are amenable to spectroscopic characterization from the visible to the terahertz region (400 nm–3 mm) in transmission mode with high signal-to-noise ratios.^5^ Spectroscopic studies have provided keen insights into the fundamental electron transfer reactions responsible for electrical power generation and recombination reactions that lower efficiency. Such spectroscopic data has also been used to test existing theories of interfacial electron transfer.^61^ Steady-state spectroelectrochemical measurements provide thermodynamic information on the dye-sensitized interface, while pulsed or modulated light excitation provides access to kinetics. In this section, insights gained over the last ten years from spectroscopic studies of dye-sensitized interfaces are presented. Unless otherwise stated, sensitized anatase TiO_2_ thin films immersed in organic electrolyte solvents at room temperature can be assumed.

Emphasis is placed on the kinetics and mechanisms for photo-induced interfacial charge separation, sensitizer regeneration, and charge recombination. The sensitizer ground and excited state reduction potentials are often taken from measurements in fluid solution and are assumed to remain unchanged upon surface anchoring. However, there is now growing evidence that the physical location of sensitizers within the electric double layer results in behavior very different from that in a fluid solution, a point that is elaborated upon here.^62^ An interesting observation is that the sensitizer redox chemistry rarely obeys the Nernst equation when anchored to TiO_2_. Recall that a 59 mV change in the applied potential should result in a factor of ten change in concentration at room temperature, but for sensitizers anchored to TiO_2_ it typically implies a ∼100 mV potential step. This behavior is typically quantified by the introduction of a “non-ideality” factor (*α*) in the modified Nernst equation (eqn (7)).7
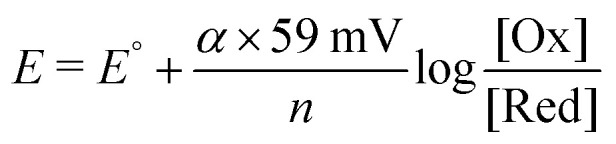


Insights into the origin(s) of this non-ideal equilibrium redox chemistry came from metalloporphyrin sensitizers that had two adjacent quantifiable redox couples when surface anchored, Co(iii/ii) and Co(ii/i).^63^ The Co(iii/ii) reduction was nearly ideal yet the Co(ii/i) process had a large non-ideality factor of 1.6 ≤ *α* ≤ 2.5. Such behavior was not easily rationalized with a “Frumkin” model wherein intermolecular interactions influence the redox equilibria. Instead, the data were most consistent with a model wherein a fraction of the electric field was present across the inner Helmholtz plane of the electric double layer. The results indicated that non-ideality was most significant when the TiO_2_(e^−^) concentration was high with a percentage potential drop of only ∼15% for the Co(iii/ii) couple and 45% for Co(ii/i).^63^

Further insights into non-Nernstian redox chemistry were gained from sensitizers where a redox active center closest to the oxide surface showed a higher non-ideality factor *α* = 1.4 ± 0.2 than a more remote center with *α* = 1.1 ± 0.1.^64^ This suggested that proximity to the oxide surface and location within the electric double layer contribute to non-Nernstian behavior. The impact of the electric field on the spectroscopic and the non-exponential kinetics described below remains unknown. More fundamental research is needed to fully elucidate the origin(s) of this intriguing interfacial redox chemistry.

#### 2.6.1 Photoinduced, interfacial charge separation

Light-initiated transfer of an electron from a sensitizer to a semiconductor provides a molecular means to convert light into potential energy in the form of an interfacial charge-separated state comprised of an oxidized sensitizer and an injected electron. The charge separation mechanism that has received the most attention from a practical and fundamental point of view involves light absorption to form a sensitizer excited state followed by electron transfer to the semiconductor, a process that is often called electron injection.^65^ This is the focus here. In addition to the aforementioned one, two alternative mechanisms have been identified to create an interfacial charge separated state with light. In a photogalvanic-type mechanism, the sensitizer excited state is first reduced by an electron donor followed by electron transfer from the reduced sensitizer to the semiconductor. In some cases, it has proven difficult to distinguish this mechanism from the case where the excited state is the donor.^66^ The second involves specific classes of dyes that form strong adducts that give rise to a new absorption band(s) due to direct charge transfer to the semiconductor.^67^ While these latter two mechanisms are well documented in the dye-sensitization literature, they have received less mechanistic and practical attention over the last ten years.

##### 2.6.1.1 Excited-state electron injection

It has been known for some time that electron transfer from a photoexcited sensitizer to TiO_2_ can occur on ultrafast femtosecond time scales.^65^ If such excited-state electron injection was quantitative and general, a wide variety of sensitizers and light absorbing materials could be widely employed. Unfortunately, this is not the case. Below, excited-state injection is discussed for inorganic charge transfer excited states and organic sensitizers.

###### Inorganic charge transfer excited states

**Fig. 10:**
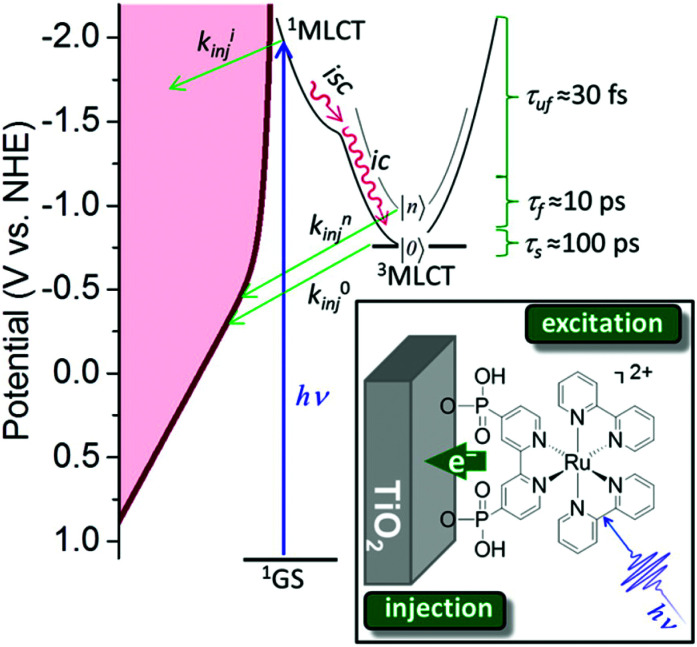
The energetic overlap of the initially-formed Frack-Condon state (^1^MLCT) and the photoluminescence ^3^MLCT with the acceptor states in anatase TiO_2_ at pH 1. Intersystem crossing (isc) and internal conversion (ic) compete kinetically with excited-state injection. Inset shows the structure of a Ru(ii) sensitizer undergoing excited-state injection. Adapted with permission from ref. 68. Copyright 2016 American Chemical Society.

A recent advance in excited-state injection was garnered from a kinetic study of [Ru^II^(4,4′-(PO_3_H_2_)_2_-2,2′-bipyridine)(LL)_2_]^2+^ sensitizers, where (LL) is an ancillary 2,2′-bipyridine ligand that tuned the excited-state potentials from −0.69 to −1.03 V *vs.* NHE.^68^ Excited-state injection showed biphasic kinetics occurring mainly at the 3–30 ps and 30–500 ps range in acidic aqueous solution. The slower process was assigned to injection from the thermally-equilibrated excited state with rate constants that were directly correlated to the excited-state potential *E*°(Ru^III/II^*). Strong photoreductants transferred electrons to TiO_2_ more quickly than did weaker excited state reductants. Electrochemical measurements were used to estimate the TiO_2_ acceptor state distribution and the overlap with *E*°(Ru^III/II^*) was correlated with the injection rate constant. Such behavior is expected based on Gerischer's model for interfacial electron transfer. The faster injection components were not analyzed in detail and were assigned to injection from higher energy unequilibrated excited states. The data indicate that the commonly reported non-exponential kinetics for electron injection can be rationalized by a continuous decrease in the injection rate constants that accompany excited-state relaxation from the initially formed Franck–Condon state to the thermally-equilibrated photoluminescent state (Fig. 10).^68^

**Fig. 11:**
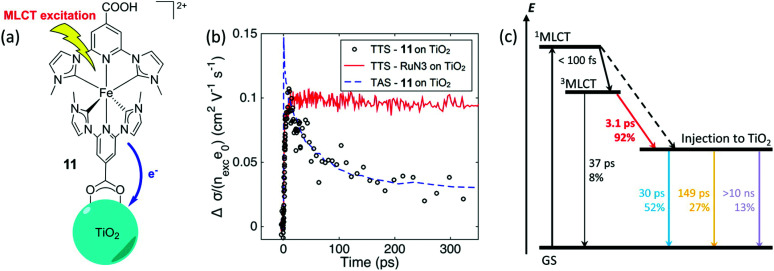
(a) Chemical structure of the N-heterocyclic Fe(ii) carbene complex anchored to TiO_2_. (b) Transient absorption and terahertz kinetic data for the iron carbene complex and for N3. (c) A Jablonski-type diagram. Reprinted with permission from ref. 75. Copyright 2016 American Chemical Society.

Historically, Fe(ii) diimine complexes have resulted in very low excited-state injection yields and there is now a detailed theoretical^69,70^ and experimental^71,72^ understanding of this. In brief, the charge transfer excited states are rapidly deactivated through low-lying metal-centered states. The exciting discovery of luminescent N-heterocyclic Fe(ii) carbene complexes with long-lived excited states has dramatically changed this landscape.^73–77^ A comprehensive study with electron paramagnetic resonance spectroscopy, transient absorption and terahertz spectroscopies as well as quantum chemical calculations revealed an injection yield of 0.92 from the MLCT excited state.^74^ Such injection yields were unprecedented for charge transfer excited states based on iron sensitizers. The key to success was the realization of a 18 ± 1 ps charge transfer excited state whose lifetime exceeds that of iron polypyridyl complexes by about a thousand-fold. The nearly quantitative injection yield has motivated many to explore related Fe(ii) carbene complexes with ground state Fe(iii/ii) potentials favorable for regeneration with donors like iodide.^75–77^ First row transition metal sensitizers based on Cu(i) and Co(i) have also been found to inject electrons efficiently into TiO_2_ (Fig. 11).^78–80^

###### Organic excited states

The late Charles Schmuttenmaer reported novel terahertz injection studies of porphyrins and metalloporphyrins anchored to TiO_2_ and SnO_2_.^79–82^ The long-term goal of these studies was dye-sensitized water oxidation, and high potential porphyrins that were weak excited state reductants was the predominant focus. The injection yields were often less than unity on to TiO_2_ surfaces and were enhanced on SnO_2_ by virtue of a ∼0.5 eV more positive conduction band edge. On both substrates and similar to the ultrafast injection studies with Ru(ii) sensitizers, more rapid injection was observed with porphyrins that were stronger photoreductants in the fluorescent singlet excited state. The THz measurements were made in the absence of an electrolyte. An interesting aspect of the porphyrin sensitizers is the presence of low-lying triplet states whose population was shown to impact the injection yield. The orientation of the porphyrin with respect to the oxide surface was also controlled by functional groups for surface binding on the aromatic porphyrin ring or through axial ligation in metalloporphyrins. It is interesting to note that injection from porphyrins with hydroxamate binding groups was as good as that measured with the more commonly used carboxylate groups.^79^

Ultrafast excited-state injection studies of porphyrins anchored to TiO_2_ through well-defined rigid linkers have been reported.^83^ Application of a time domain vibrational spectroscopy pump degenerate four-wave mixing technique enabled identification of the Raman-active modes triggered by light absorption. The spectral data were assigned to modes based on the linker group and that localized on the porphyrin ring. The data suggested that this four-wave mixing technique can distinguish between vibrational modes generated by light absorption from those generated by excited-state injection.^83^

In a related study, excited-state injection by (perylene-9-yl)carboxylate into TiO_2_ was shown to be complete within 12 fs.^84^ The ultrafast transient absorption data mapped the decay of the singlet excited state and the appearance of the oxidized perylene. Nonadiabatic quantum dynamic simulations indicated that injection was complete within 20 fs, in close agreement with the experimental value. The reorganization energy for electron transfer was estimated to be 220 meV. Non-equilibrium modes in the 1000–1800 cm^−1^ region were assigned to in-plane asymmetric vibrations of the perylene sensitizers. The agreement between theory and experiment in these studies indicates that these are powerful tools for quantifying vibronic effects at dye-sensitized interfaces.^84^

#### 2.6.2 Sensitizer regeneration

Upon excited-state injection the oxidized sensitizer is reduced by an electron donor present in the electrolyte in a process known as sensitizer regeneration. It is not sufficient for the oxidized sensitizer to be thermodynamically competent of donor oxidation, the reaction must occur more rapidly than the competitive recombination (cr), *i.e.* the electron transfer from the semiconductor to the oxidized sensitizer, with rate constant *k*_cr_. The most common and successful donor by far is iodide, with Co(ii) diimine complexes also having a long history. Emergent new mediators based on Fe(iii/ii) and Cu(ii/i) transition metal complexes have been characterized by transient spectroscopic techniques.

The classical iodide/triiodide redox mediators have been the subject of several prior reviews and are only summarized here.^85–87^ Iodide oxidation yields a metastable species in di-iodide, I_2_^−^˙, either through the iodine atom intermediate I˙ + I^−^ → I_2_^−^˙ or (possibly) through a concerted pathway. Di-iodide is unstable with respect to disproportionation: 2I_2_^−^˙ → I_3_^−^ + I^−^. In acetonitrile solutions, the one-electron reduction of I_3_^−^ by TiO_2_(e^−^) is thermodynamically uphill and the equilibrium concentration of I_2_ is small. These factors allow for efficient transport of the injected electrons with minimal recombination. Iodide oxidation happens on a time scale of hundreds of nanoseconds for most sensitizers. Many researchers concluded that the regeneration by iodide was completely optimized using quantitative Incident Photon-to-Current Efficiency (IPCE) in the short circuit condition. However, at the open-circuit or power point conditions, where the number of electrons in each nanocrystallite is large, there is now clear evidence that regeneration is non-quantitative.^88,89^ The regeneration quantum yield, *Φ*_reg_, has been determined spectroscopically by eqn (8), where *k*_reg_ is the pseudo-first-order regeneration rate constant at molar donor concentration [D].8
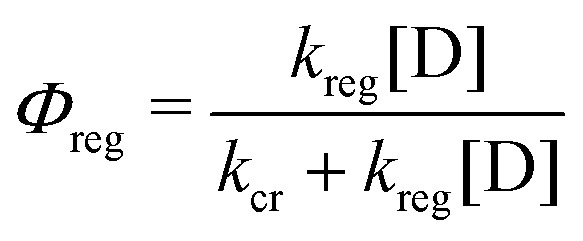


Nanosecond transient absorption kinetic measurements were made with D–π–A sensitizers as a function of the applied potential to simulate conditions along the current–voltage curve. It was found that *Φ*_reg_ decreased from unity to 0.83 at the open-circuit condition with 0.5 M I^−^. For 0.3 M [Co(bpy)_3_]^2+^, the quantum yield decreased to 0.60.^88^ Irradiance-dependent photoelectrochemical measurements with the classical N3 sensitizer provided the same conclusion: regeneration is quantitative at short-circuit and non-quantitative at the open-circuit and power point conditions.^89^ For alternative oxides, such as SnO_2_, regeneration has also been shown to be non-optimal due to the more rapid recombination.^90^ Realization that regeneration can be better optimized to enhance fill factors and open-circuit photovoltages continues to inspire researchers to design interfaces capable of more rapid regeneration without a significant loss of free energy.

**Fig. 12:**
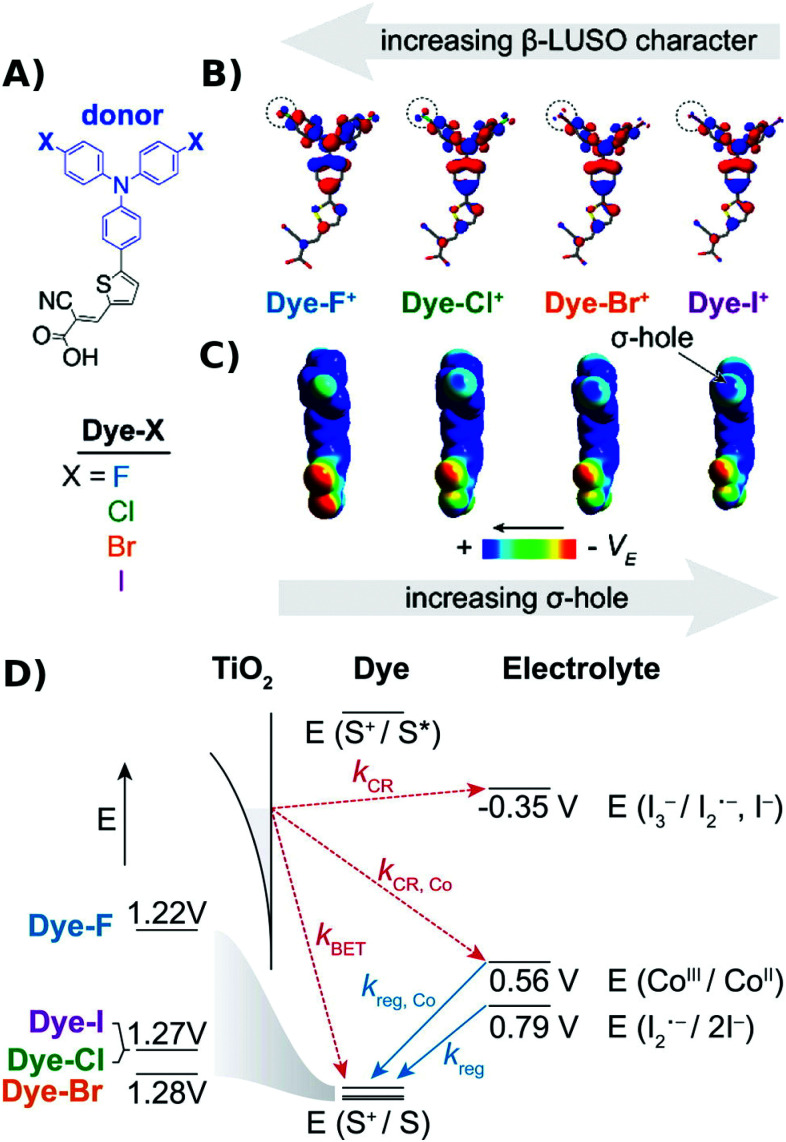
(A) Molecular structures of the Dye-X series. (B and C) DFT models of the singly oxidized forms of Dye-X showing (B) the β-LUSO and (C) the existence of σ-holes on the poles of the terminal halogen substituents for the series, with the exception of Dye-F. (D) Scheme of energy levels and electron transfer processes. Adapted with permission from ref. 92. Copyright 2016 American Chemical Society.

Regeneration kinetics have been enhanced with sensitizers competent of halogen and chalcogen bonding.^91–93^ Kinetic regeneration studies of organic D–π–A sensitizers where the triphenylamine donor was substituted with halogen atoms were conducted, Fig. 12. In their oxidized form the presence of a σ-hole for halogen bonding was apparent in the sensitizers with Br and I. Transient spectroscopic studies revealed a correlation between the sensitizer halogen bonding ability and the second-order regeneration rate constant by iodide, yet no trend was observed with [Co(bpy)_3_]^2+^, which is incapable of halogen bonding. While the power conversion efficiency enhancements were small, these studies provided compelling evidence that halogen bonding can be utilized to enhance regeneration kinetics and yields at dye-sensitized/TiO_2_ interfaces.

A notably rapid regeneration process was reported for highly cationic Ru(ii) sensitizers, [Ru(tmam)_2_(dcb)]^6+^, where tmam is the quaternary ammonium derivative, *i.e.* 4,4′-bis-(trimethylaminomethyl)-2,2′-bipyridine.^94^ When anchored to TiO_2_, these sensitizers showed clear evidence of ion pairing with iodide and an anionic cobalt redox mediator (*K*_eq_ > 10^4^ M^−1^) in acetonitrile. Injection and regeneration on time scales of less than 10 ns were achieved using Co mediators. Diffusion limitations associated with sensitizer regeneration were improved by ion pairing and the IPCE nearly doubled.^94^

**Fig. 13:**
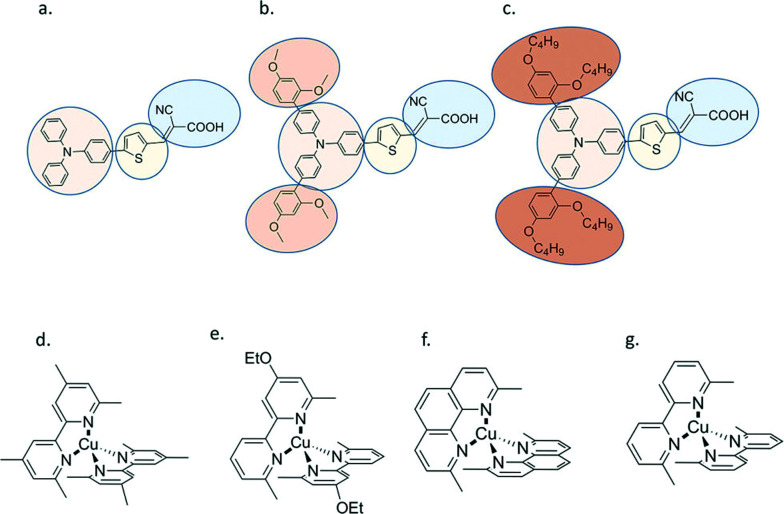
Molecular structures of (a) D5, (b) D45 and (c) D35 dyes, and (d) [Cu(tmby)_2_]^2+/+^, (e) [Cu(eto)_2_]^2+/+^, (f) [Cu(dmp)_2_]^2+/+^ and (g) [Cu(dmby)_2_]^2+/+^ complexes. Reprinted with permission from ref. 95. Copyright 2018 American Chemical Society.

An interesting aspect of Cu(ii/i) bipyridyl mediators is that the two redox states often have very different coordination environments.^95–102^ The Cu(i) redox state is typically four-coordinate with a pseudo-tetrahedral geometry, while Cu(ii) is subject to a Jahn–Teller distortion that is often manifest in five-coordinate complexes with the fifth ligand derived from solvent or counter-ion. In a comprehensive study with three different D–π–A sensitizers, regeneration by the four Cu(i) diimine mediators shown was investigated, Fig. 13.^95^ These mediators possess methyl groups in the 6,6′ positions of bipyridine and the 4,7 positions of 1,10-phenathroline that prevent planarization of the two ligands in the Cu(ii) state, resulting in a significant positive shift in *E*°(Cu^II/I^). For two of the three sensitizers, the regeneration rates increased with thermodynamic driving force and *Φ*_reg_ ∼ 1 in all cases. Regeneration by [Cu(eto)_2_]^+^ was so rapid that in some cases it was unclear experimentally whether injection occurred first or whether a photogalvanic mechanism was operative. Prior work revealed that these Cu diimine complexes were able to quench the sensitizer excited states.^96^ Density functional theory calculations were used to estimate the reorganization energy – *λ* – for regeneration in the presence and absence of Lewis-basic 4-*tert*-butylpyridine (*t*BP). Interestingly, this analysis indicated that *t*BP binding to Cu(ii) had a dramatic ∼1 eV increase in *λ* that was predicted to result in charge recombination in the normal region, with Marcus inverted recombination in the absence. The ability to tune redox reactivity with external Lewis bases is a novel aspect of these mediators that may be further optimized for dye-sensitized solar cell applications.^95–102^

A significant advance in regeneration at dye-sensitized p-type NiO was realized with tris(acetylacetonato)iron mediators, abbreviated [Fe^III/II^(acac)_3_]^0/−^.^103^ The second-order regeneration rate-constant measured spectroscopically was large, 
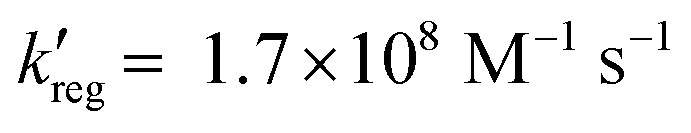
. At the mediator concentrations employed, this rate constant indicated a regeneration yield *Φ*_reg_ = 0.99. This is a particularly notable advance as these iron mediators significantly enhanced the efficiency of dye-sensitized p-type materials.^103^

#### 2.6.3 Charge recombination

The recombination of an injected electron with an oxidized dye leads to ground-state products and usually results in a loss of more than 1 eV of free energy. For charge transfer excited states based on Ru polypyridyl sensitizers, it has been known for decades that recombination occurs on a micro- to millisecond time scale with non-exponential kinetics. Interestingly, porphyrins have been reported to show recombination on the pico- to nanosecond time scale to an extent that was dependent on the porphyrin geometry.^104,105^ The relationship between “average” observed rate constants derived from transient spectroscopic data and the underlying electron transfer rate constant has been less clear. An early model assumed that the oxidized sensitizer remained fixed at the injection site while the injected electron underwent thermally-activated random walk between traps states prior to recombination.^106–108^ When trapping/detrapping was rate-limiting, the observed rate constant reported only on this process. Recent polarized light experiments have shown that the electronic hole, *i.e.* the oxidized sensitizer, does not stay at the injection site, but rather undergoes intermolecular electron transfer amongst sensitizers, a process often referred to as “hole-hopping”. Polarized light generates an anisotropic population of interfacial states whose time-dependent reactivity clearly demonstrates that hole hopping followed excited-state injection under a variety of experimental conditions.^109–111^ Monte Carlo simulations revealed that an oxidized sensitizer could circumnavigate an entire anatase nanocrystal by hole-hopping before charge recombination occurred.^110^

**Fig. 14:**
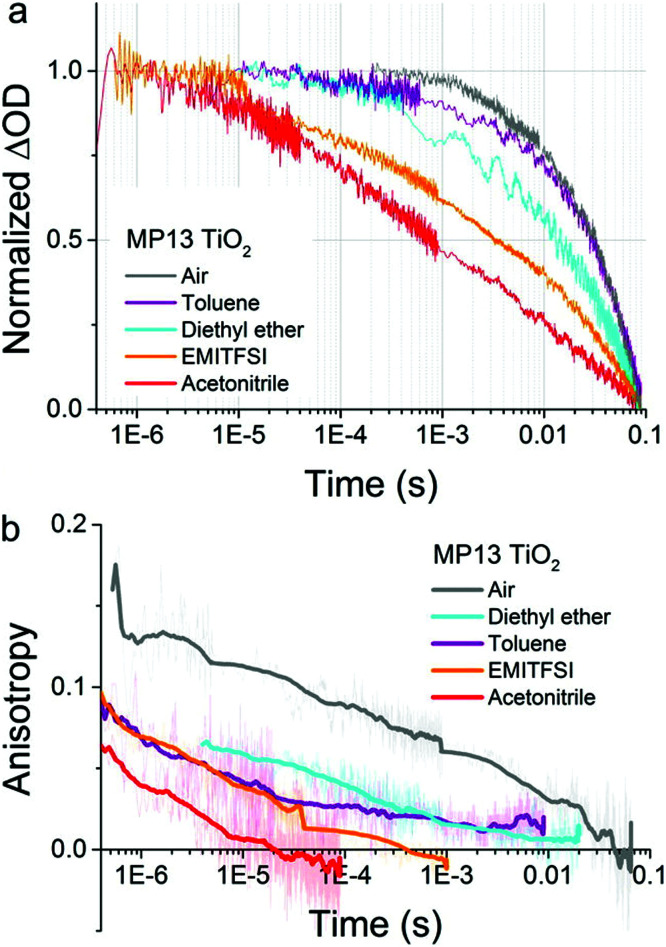
(a) Transient absorption and (b) transient absorption anisotropy spectroscopy on MP13 sensitized TiO_2_ films on glass immersed in different environments. The films were pumped with pulsed laser excitation at 430 nm while the oxidized dye signal was probed at 770 nm. The solid lines in (b) are obtained by calculating a moving average of the raw data (also displayed in background). Adapted with permission from ref. 112. Copyright 2016 American Chemical Society.

The discovery that hole-hopping rates were directly correlated with charge recombination kinetics represents an important finding.^112^ Sensitizers that undergo rapid S + S^+^→ S^+^ + S hole-hopping were shown to recombine more rapidly than those that hop more slowly. An example is shown in Fig. 14, where the transient absorption data reports on the charge recombination reaction while the anisotropy reports on hole-hopping. For the D–π–A sensitizer mp13, both hole-hopping and charge recombination responded in a similar fashion to changes in the solvent or external environment.

Studies of a homologous series of four sensitizers that maintain the *cis*-Ru(NCS)_2_ coordination environment with one surface anchoring group show that they undergo rapid hole-hopping.^113,114^ The hole-hopping rate constants – *k*_hh_ – measured electrochemically spanned about a factor of seven and followed the same trend as did the charge recombination kinetic data.^114^ Subsequent temperature and surface coverage-dependent kinetic studies with sensitizers that displayed very different hole-hopping rates also supported the conclusion that rapid hole-hopping promotes charge recombination.^115^ Interestingly, no correlation between the activation energy for hole-hopping or charge recombination was evident with the solvent dielectric, but both dynamic processes could be tuned by the addition of inert salts to the solvent or by controlling access of electrolyte cations to the oxide surface.^116^ These results lead to the conclusion that undesired recombination of charges may be reduced by limiting lateral hole-hopping. This implies that hole-hopping may play a greater role in charge recombination than transport of the injected electrons.^112^ Control of the intermolecular distance between sensitizers and the electrolyte tunes the charge recombination reaction and can favor conditions where the transient spectroscopic data reflects the true interfacial electron transfer event.

Absorption of a photon initiates the formation of one injected electron and one oxidized sensitizer. They are formed in equal numbers and a second-order recombination might be anticipated with the rate law as *r* = *k*[S^+^][TiO_2_(e^−^)]. An Ostwald isolation type approach where an applied potential was used to control the number of electrons and oxidized sensitizers identified the rate law as *r* = *k*[S^+^]^1^[TiO_2_(e^−^)]^1^.^117^ The Ostwald isolation conditions differ from those encountered in operational solar cells or in transient photovoltage measurements where alternative rate laws have been reported.^118^ In all cases, the injected electrons reside in spherical nanocrystallites interconnected in a mesoporous thin film, whereas the oxidized dye molecules are restricted to the quasi-two-dimensional oxide surface. Hence, charge recombination is an intriguing process where opposite charges on different sides of an interface come into close proximity before electron transfer occurs.

For fundamental recombination studies, transparent conductive oxide (TCO) materials have some advantages.^119–121^ They have a metallic character, which permits potentiostatic control of the Fermi level (*E*_F_) and, consequently, of the driving force for charge recombination, −Δ*G*° = *nF*(*E*°′ − E_F_). Quantifying *k*_cr_ as a function of −Δ*G*° allows analysis through Marcus-Gerischer theory and access to the total reorganization energy (*λ*) and to the electronic coupling. Studies with acceptors positioned at variable distances from a TCO surface provided a remarkable result: *λ* decreases to near zero when the acceptor is most proximate to the oxide surface.^121^ At distances greater than ∼20 Å in the diffuse part of the electric double layer, *λ* approximately equals the value expected for homogeneous reactions, *λ* ≈ 0.9 eV. Thus, dye-sensitization with transparent conductive oxides provides exciting opportunities to test interfacial electron transfer theories and to probe the impact of the electric double layer.

##### 2.6.3.1 Recombination to solution species

It was recently shown that under some conditions electron transfer from TiO_2_ to acceptors dissolved in fluid solution followed a first-order kinetic model.^122,123^ Excited-state injection followed by sensitizer regeneration with triphenylamine donors dissolved in solution were used to quantify the reaction TiO_2_(e^−^) + TPA^+^ → TiO_2_ + TPA. Interestingly, when the thermodynamic driving force for this reaction was large, first-order kinetics were operative, a non-intuitive result that suggests the TPA^+^ acceptors are electrostatically bound to the oxide surface allowing a uni-molecular-type recombination reaction. When −Δ*G*° was small, dispersive kinetics were observed and attributed to electron transport to the oxidized TPA. Temperature-dependent studies analyzed through transition state theory indicated that recombination occurs with a highly unfavorable entropy of activation.^122^ Activation energies were the same (within experimental error) – 12 kJ mol^−1^ – for all interfacial electron transfer reactions, indicating that the barriers for electron transport and interfacial electron transfer were similar. Eyring analysis indicated a substantial entropy change to the activation barrier.^123^

The TiO_2_(e^−^) + I_3_^−^ → reaction is known to be kinetically slow on a millisecond time scale, behavior that is typically attributed to an unfavorable positive Δ*G*°. The identity of Lewis acidic cations present in the electrolyte impacts the reaction kinetics.^124–126^ Alkaline and alkaline earth cations screen the electric field generated by the injected electrons and also influence charge recombination to organic acceptors.^126^ Interestingly, the SnO_2_(e^−^) + I_3_^−^ → reaction is much slower than for TiO_2_ and extends to the seconds time scale, presumably by virtue of the more positive SnO_2_ donor states.^90^

##### 2.6.3.2 Sensitizer–bridge–donor (S–B–D) acceptors

A successful approach for inhibiting unwanted charge recombination is to regenerate the oxidized sensitizer by intramolecular electron transfer.^127–130^ In this approach, electron transfer occurs from a donor D covalently linked through a bridge unit B to the oxidized sensitizer S. An interesting observation was that a relatively small structural change in the planarity of an aromatic bridge altered the electron transfer mechanism from adiabatic to non-adiabatic. Interestingly, recombination to S^+^ and D^+^ were the same for adiabatic transfer, while non-adiabatic transfer to D^+^ was markedly inhibited. The kinetic data revealed that recombination utilized a bridge-orbital pathway.^127^

In one study, the S^+/0^ and D^+/0^ reduction potentials were very similar such that excited state injection created a quasi-equilibrium *K*_eq_ = *k*_1_/*k*_−1_ that was quantified over an 80 °C temperature range, TiO_2_|S^+^–B–D ⇌ TiO_2_|S–B–D^+^. A significant barrier was measured under all conditions indicating that a true redox equilibrium was operative. The magnitude of *K*_eq_ was closer to unity for the phenyl bridge and hence 
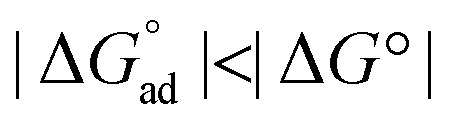
, as had been predicted theoretically. The van't Hoff shown for the adiabatic equilibrium clearly indicates Δ*H*° = *q*_p_ = 0, and that the equilibrium constants are determined solely by Δ*S*°. For the non-adiabatic equilibrium, Δ*H*°= ± 7.0 kJ mol^−1^.^128^ The results show that the magnitude of Δ*G*° is decreased when adiabatic pathways are operative, a finding that should be considered in the design of S–B–D sensitizers for dye-sensitized solar cell applications.^129,130^

## 3 Theory and computational studies

**Fig. 15:**
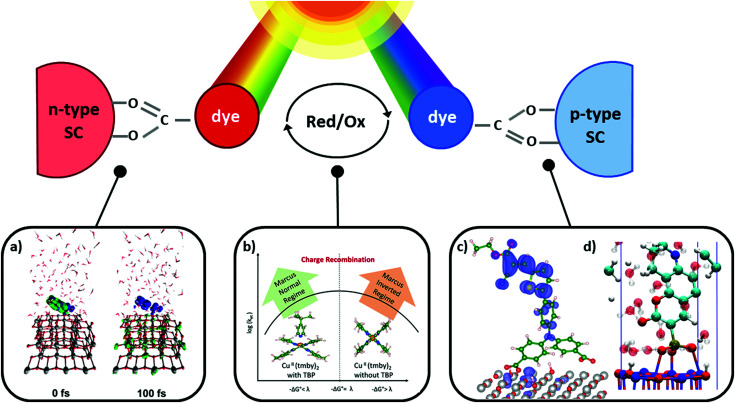
Examples of recent computational studies on DSC components. (a) electron (green) and hole (blue) densities at the beginning of the simulation (*t* = 0 fs) and upon electron injection (*t* = 100 fs) for benzohydroxamic acid anchored on TiO_2_ with full explicit water solvation. Adapted with permission from ref. 135. Copyright 2020 American Chemical Society. (b) Analysis of charge transfer parameters in Cu-based electrolytes. Adapted with permission from ref. 95. Copyright 2018 American Chemical Society. (c) Isosurfaces of band-decomposed charge density of the lowest unoccupied band of the push–pull dye T1/NiO system. Adapted with permission from ref. 136. Copyright 2019 American Chemical Society. (d) Anchoring geometry of C343 as a model dye on NiO during the molecular dynamics simulation in explicit water. Adapted with permission from ref. 137. Copyright 2017 American Chemical Society.

DSCs offer a unique playground for fundamental studies of complex phenomena concerning sunlight harvesting, charge and mass diffusion across multi-layer heterogeneous interfaces, and electrochemistry. Theory and computation have been key players in providing the scientific foundation to understand and dissect DSC devices, starting from isolated components (*e.g.* dyes, electrodes) and elementary processes up to electron/ion transport properties at hybrid organic–inorganic and liquid–solid interfaces.^131–134^ This section presents a brief outline of the state-of-the-art theoretical methods addressing these systems and processes, with a particular focus on cutting-edge studies from the last ten years (Fig. 15).

### 3.1 Theoretical background

Simulation of sunlight conversion to electricity in DSCs calls for the application of several theoretical methods to tackle complex materials and processes that span across several scales of space and time. Light harvesting, dye/electrode charge transfer, electron transport to the charge collector, oxidized dye regeneration, electrolyte diffusion, and reduction at the counter electrode are all processes that occur at different places and with different time frames, from femtoseconds to milliseconds. Therefore, the simulation approach must be multi-scale, starting from the elementary processes at the nano scale and adding step-by-step the effects coming from larger (longer) space (time) scales.

Initially, the quantum mechanical (QM) interactions among electromagnetic radiation, electrons, and nuclei need to be properly described. Within this framework, Density Functional Theory (DFT) is the current method of choice for the electronic structure of materials and interfaces,^138^ and its extension to Time-Dependent DFT (TD-DFT) has also enabled the effective description of excited state properties.^139^ However, the application of Kohn–Sham DFT and the related TD-DFT still suffers from the approximate nature of the unknown exchange–correlation (XC) density functional.^140^ This flaw is very relevant for modeling within the context of DSCs as it can jeopardize DFT results reliability in predicting charge transfer processes involving strongly correlated materials (*e.g.* transition metal oxide-based electrodes) and non-covalent weak interactions (*e.g.* dispersion forces).^141^ Recent theoretical advances in XC formulations and other effective approaches have been able to amend most of these drawbacks, but often only on a case-specific base. Moreover, DSC molecular and solid-state components have been traditionally studied within different numerical approximations, with no or little overlap, which has hindered an easy transfer of theoretical advancements from one DSC component to the other. For example, successful TD-DFT approaches for molecular dyes are not numerically feasible for solid-state electrodes. *Vice versa*, new approaches beyond DFT (*e.g.* GW^142,143^ and RPA^144^) for bulk-extended materials are still not feasible for realistic hybrid interfaces. Thus, the following sections will discuss: (i) the best available approaches for each DSC component, (ii) the relevant physico-chemical properties to be computed, and (iii) how the results from first-principles calculations can be implemented in multi-scale models to predict the overall DSC power conversion efficiency.

### 3.2 Theoretical description of sensitizers and molecular components

Since the earliest characterization of Ru-based^145,146^ and organic^147^ dyes, the computer power and theoretical machinery for modeling excited states of molecular species has considerably grown.^148^ The advancements in XC functionals (long-range corrected hybrid^149^ and double hybrid^150^) and in TD-DFT algorithms (*e.g.* analytical first derivatives) allowed the molecular design of dyes with specifically tailored properties for application in n-type^151,152^ and p-type^153^ photoelectrodes. The combination of long-range corrected density functionals like CAM-B3LYP or ω-B97X and triple-z quality basis sets such as 6-311++G(d,p) and def2_TZVP have provided excellent results even for the challenging cases of intra-molecular charge-transfer excitations.^154^ When TD-DFT fails, excited-state properties can still be obtained by means of wavefunction-based methods (*e.g.* CASPT2,^155^ NEVPT2^156^ and EOM-CCSD^157^), whose major limit is the dye size, due to their high computational cost.

A key strategy to avoid undesired charge recombination is based on the development of push–pull dyes, where the excited electron is localized close to the electrode (for standard n-type DSCs^158^) or exposed to the solvent (in photocathodes^159^). The molecular design of new dyes with such characteristics has been greatly aided by the topological analysis of electron density changes upon photoexcitation, such as the combination of TD-DFT and density-based charge-transfer indexes.^160^ This approach is based on the analysis of the difference between the charge densities of the excited and the ground states and has been proven to be very effective for molecular dyes,^161^ including metal-based ones.^162,163^ Additionally, this approach has been recently updated to account for complex dye structures.^164^

A significant novel contribution of the DFT-based quantum chemistry approach is related to the new transition metal complexes developed as redox shuttle substitutes to the I^−^/I_3_^−^ electrolyte. First-principles approaches have been exploited to assess the molecular parameters related to their redox potential – to be compared with the dye HOMO energy level – in order to evaluate the driving force for dye regeneration,^165^ as well as to consider the reorganization energies upon oxidation within a diabatic charge transfer scheme based on Marcus theory.^166^ The results of hybrid DFT on Co and Cu complexes present certain levels of inaccuracy in predicting the redox potentials, with errors usually around 0.2–0.5 eV with respect to experimental data.^14^ This is due to the approximate nature of the XC density functional when comparing two systems with a different number of electrons. A much better agreement between theory and experiment is achieved in the computation of reorganization energies (*λ*) and corresponding charge transfer kinetic parameters.^95,167^

The accuracy in predicting such parameters (photoexcitation, redox potential, reorganization energies) largely depends on the approach used for modeling the chemical environment. A well-known and effective strategy to model the structure and properties of solvated systems is represented by focused models, where the system is partitioned into a chemically interesting core (*e.g.* the solute in a solution) and the environment, which perturbs the core, modifying its properties. While a level of theory as high as required is retained for the core, the environment is treated in a more approximate way. Two popular alternatives of such approaches are: (i) to consider the environment as a structure-less continuum as in the Polarizable Continuum Model (PCM),^168^ or (ii) to retain its atomistic resolution within a molecular mechanics (MM) description.^169^ Both alternative strategies can be effectively coupled to a QM description of the core, and can also be coupled together to overcome their respective limitations.^170^ In the context of DSC, PCM and hybrid QM/MM approaches have been extensively applied to account for the solvent effects on the physico-chemical properties of dyes and redox shuttles.^171^

### 3.3 Simulation of solid-state electrodes and heterogeneous interfaces

The first systematic computational studies on DSCs concerned the main components of the original Grätzel cell, focusing mostly on n-type semiconductor oxides (*e.g.* TiO_2_, ZnO, SnO_2_) and their interfaces with molecular dyes (*e.g.* dye anchoring groups).^131–134^ In the last decade, the quest for tandem cells has spurred theoretical studies also on p-type DSC components^172^ (p-type semiconductors, push–pull dyes, and their interfaces), which were barely studied in the first years of the modern DSC technology. In both cases, studies of electrode and counter electrode materials have relied on the periodic supercell DFT approach, mainly by employing plane-wave basis set and pseudo-potentials replacing core electrons.^173–176^ Standard local and semi-local XC functionals have been recently replaced mostly by DFT+*U*^177^ and hybrid HF-DFT^178^ for modeling the strong-correlated nature of the transition metal oxides that are commonly employed as electrodes in DSCs. The characterization of band structures with these methods can provide useful hints on the nature of the bandgap and the possible optical properties, as well as on electron/hole mobilities.^179^ Within this framework, recent studies have explored several possible alternatives to NiO for p-type DSC and tandem cells.^180,181^ While semi-local DFT (GGA) provides too low of a bandgap, the DFT+*U* approach strongly depends on the choice of the Hubbard-like *U*–*J* parameter. The hybrid HF-DFT approach tends to overestimate the bandgap, and the estimate is also affected by the choice of HF-like exact exchange percentage into the HF-DFT scheme. Methods based on Green function (GW) and on the Random Phase Approximation (RPA), as well as methods based on Bethe-Salpeter equation (BSE) and TD-DFT have the potential of providing results in quantitative agreement with experiments, but their feasibility is hindered by high computational cost.^182^ Besides these shortcomings, thanks to the relatively good accuracy in predicting bandgap centers by standard DFT and considering the Janak's theorem, it is possible to compute the absolute potentials *vs.* NHE of the electrode band edges within a surface slab approach.^183^ In particular, the conduction band (CB) is relevant for photoanodes, and the valence band (VB) is relevant for photocathodes. Comparing these values to the computed HOMO and LUMO energies of the dye provides a powerful tool to assess the quality of a dye/electrode combination. The dye LUMO must be higher in energy than the electrode CB in n-type DSCs and the dye HOMO must be lower than the electrode VB in the p-type counterpart to allow for convenient electron and hole injections, respectively.

In the last decade, the availability of more and more powerful computing facilities allowed the study of the dye/electrode interface at the full atomistic scale. From cluster-size electrodes with few atoms,^184,185^ computational tools now have the capability of simulating the full electrode surfaces with periodic boundary conditions, including the attached dyes^186^ and, in some cases, also the explicit solvent medium.^137^ The characterization of dye/electrode interfaces has provided great advancement in the understanding of the complex interfacial electronic processes.^187^ For both n- and p-type DSCs, it has been possible to assess the strength of the dye-surface anchoring,^188–190^ the role of dipole moment at the surface in tuning the electrode CB/VB edge potential,^191^ and the effects of surface polarization^192,193^ and the electrolyte solution^194^ on the dyes' electronic structure. The results allowed for a better design of dyes, with specific anchoring groups and with electron-donor/acceptor moieties well distributed into the dye molecular architecture.^195^

All these studies have paved the route to the recent implementation of real-time TD-DFT simulations of the dye/electrode interface after sunlight absorption and charge separation.^196–198^ With these approaches, mostly focused on n-type DSCs, it has been possible to dissect the specific mechanism and kinetics of charge transfer between the excited dye and the electrode, as well as of undesired charge recombination events.^194^ These studies still retain some empiricism, for example in the choice of some parameters that need to be fitted to experiments, but they certainly represent a frontier in the theoretical modeling of DSC interfaces, and we can expect further developments of these tools in the near future.

Last but not least, the importance of using the results from atomistic simulations in macroscopic modelling approaches must be mentioned. For example, the computed charge transfer rates can be implemented in a kinetic Monte Carlo approach for the simulation and interpretation of complex electrochemical measurements (*e.g.* impedance).^199^ At the same time, computed parameters derived from the isolated dye, the pristine electrode, and the dye/electrode interface can be conveniently cast in empirical formulae to obtain a realistic estimate of the photo-conversion efficiency.^200^

### 3.4 New horizons in modeling DSC devices

The great challenge of finding new materials and interfaces for DSCs requires further advancements in computational techniques. Although the atomistic description of complex materials and interfaces may still benefit from the accuracy and versatility of *ab initio* methods, new tools are emerging within the ongoing extraordinary revolution in computational sciences that involves Artificial Intelligence (AI) and Data Sciences. DSC development fits in these new approaches at different levels and, indeed, the first AI-based studies on DSC are now reality.^201^ On one hand, AI under Machine Learning-based approaches has been applied for electrode materials and dyes,^202–204^ tailoring specific structure–property relationships with deep-learning neural networks rather than first-principles equations. On the other hand, several tools are already available for automated screening and analysis of large datasets,^205^ compiled from experiments and/or advanced QM calculations, aimed at finding new, unexpected combinations of DSC components that maximize photo-conversion efficiencies, even at different light conditions.^206–208^ The future of these tools looks bright, together with their further integration within the new promising quantum information technologies.^209^

## 4 Materials

### 4.1 Nanostructured metal oxide electrodes

Nanostructured semiconductor electrodes provide a large surface area for dye adsorption, an essential feature for DSCs. The most commonly used type of nanostructured electrode in DSC is the mesoporous electrode, which is composed of 10 to 50 nm-sized nanocrystals and has a porosity of about 50%. Other types of metal oxide nanostructures that have been applied in DSC are nanotubes, nanorods, nanofibers, nanosheets, *etc.*

**Fig. 16:**
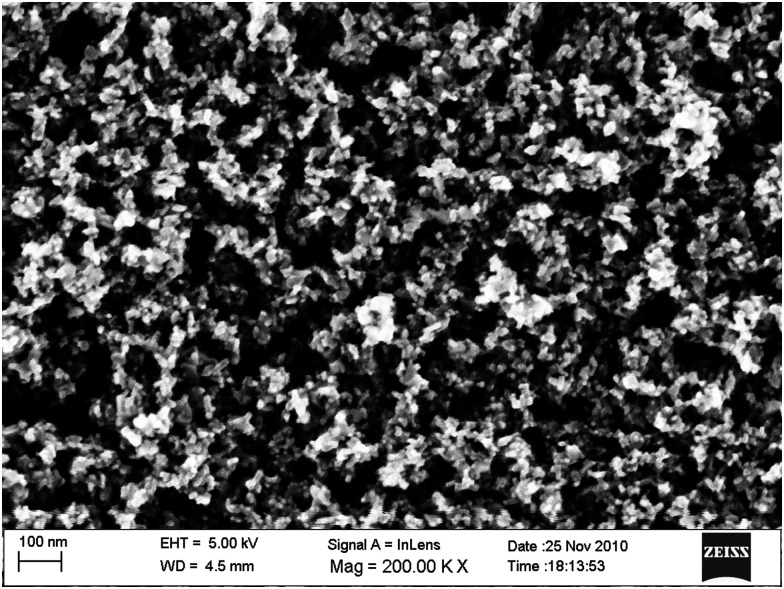
SEM image of a mesoporous TiO_2_ film made with the GreatCell Solar 18NR-T paste.

By far, the most used material for mesoporous electrodes is TiO_2_ with the anatase crystal structure (Fig. 16). This wide bandgap semiconductor has an indirect bandgap of 3.2 eV. The standard method for the preparation of mesoporous TiO_2_ electrodes is by screen printing of a suitable paste, followed by annealing in air at high temperature (400–500 °C) to burn out the organic additives required to make a paste with appropriate rheological properties and giving the required porosity. This heat treatment also gives a partial sintering of the TiO_2_ to make electronic connections between the particles and gives mechanical stability to the film. Depending on the precise composition, the mesoporous TiO_2_ film can be completely optically transparent, or have a slight white color. Several commercial suppliers offer suitable TiO_2_ screen printing pastes.

A light scattering layer containing ∼400 nm-sized TiO_2_ particles is frequently deposited on top of the mesoporous layer. This layer reflects transmitted light back into the active film and usually improves the efficiency for DSC devices that are illuminated through the FTO/glass substrate. Light-scattering particles can also be added to the mesoporous film paste to obtain a similar effect; the latter method is more appropriate for DSC with illumination from the counter electrode side. We refer to ref. 210 for further reading on application of light scattering in DSC.

For best performance, it is common in research papers to apply a TiCl_4_ treatment: mesoporous TiO_2_ films are immersed in an aqueous TiCl_4_ solution, leading to chemical bath deposition of an ultrathin layer of TiO_2_ (about 1 nm) onto the mesoporous electrode and the underlying conducting glass.^211^ A further heat treatment is used to crystallize the material and to remove water.^212^

The porosity and pore size of mesoporous films are particularly important for the use of alternative redox mediators, such as cobalt bipyridine complexes. In this case, a marked improvement of DSC performance was found at one sun illumination, from 1.4% to 4.8%, when the porosity was increased from 52% to 59%.^213^ Deviations from linearity of photocurrent *vs.* light intensity plots, as well as photocurrent transients clearly demonstrated the occurrence of mass transport limitations of the redox mediator. Yella *et al.* demonstrated that best performing DSCs with cobalt bipyridine redox mediator should have a thinner added TiO_2_ layer deposited by TiCl_4_ after screen-printing.^214^

Doping of TiO_2_ can give some positive effects by adding or removing trap states, changing the band edge levels, improving dye adsorption, and by stabilizing the anatase phase, as recently reviewed by Roose *et al.*^215^ For instance, a high *V*_OC_ of 1.45 V was obtained by Mg doping of TiO_2_ through an additional MgO/Al_2_O_3_ surface treatment and employing a bromide-based redox electrolyte.^216^ In highly efficient DSCs, however, the state-of-the art mesoporous TiO_2_ electrodes are not doped.

A large variety of TiO_2_ nanostructures have been tested in DSCs: one-dimensional structures such as nanotubes and oriented nanorod arrays,^217^ mesoporous microbeads^218^ and mesoporous single crystals.^219^ Templating methods provide a route to ordered mesoporous TiO_2_ materials, with soft-templating methods using surfactants and hard-templating methods using silica or polystyrene spheres.^220^ None of these structures, however, outperform standard mesoporous TiO_2_ electrodes under optimized conditions.

**Fig. 17:**
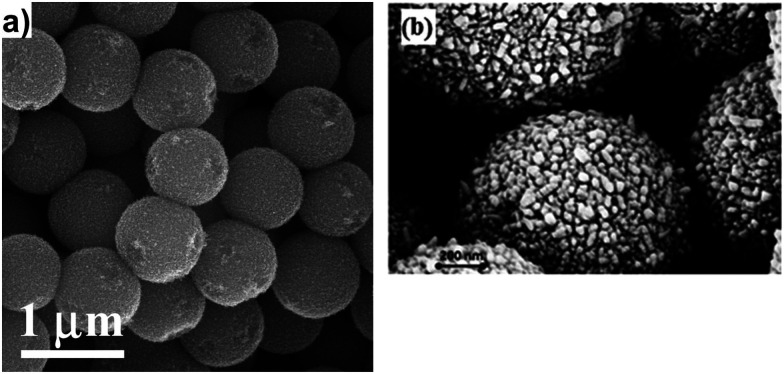
SEM micrographs of mesoporous TiO_2_ microbeads. (a) Adapted with permission from ref. 223. Copyright 2010 American Chemical Society. (b) Adapted from ref. 222 with permission from The Royal Society of Chemistry, copyright 2014.

In 1D structures (nanotubes and single crystalline nanorods), faster electron transport is often named as a potential advantage for these structures. In practice, however, the charge collection in mesoporous films is sufficiently high, so that no solar cell improvement can be expected on that basis. Mesoporous TiO_2_ microbeads are of potential interest for several reasons: first, a high PCE of 10.7% was achieved in a single printed layer;^218^ second, they can be annealed at high temperature and sensitized before application onto a (flexible) substrate. Furthermore, this and other structures with hierarchical architecture can have an advantage with respect to mass transport in the electrolyte. Mesoporous microbead electrodes outperformed standard mesoporous electrodes when using a more viscous MPN-based cobalt electrolyte at 1 sun light intensity.^221^ Microbead electrodes were also successfully applied in solid-state DSCs (Fig. 17).^222^

A disadvantage related to TiO_2_ as a material for the dye-sensitized solar cell is its photocatalytic activity:^224^ direct excitation of the semiconductor leads to highly energetic holes that can oxidize organic compounds. This lowers the long-term stability of DSC under illumination. Such degradation can be avoided by adding a UV-filter to the solar cell, but this will lead to additional cost. The UV activity of TiO_2_ is one reason to look into alternatives.

There are many other metal oxides that can be applied in the working electrode of a DSC. ZnO is the most investigated alternative to TiO_2_, in a wide variety of nanostructures.^225,226^ Its electron mobility is much higher than that of TiO_2_, but its (photo)chemical stability is lower. SnO_2_ is chemically very stable, has a higher bandgap than TiO_2_, but a lower conduction band edge energy, leading to a lower photovoltage in DSCs.^227^ Both ZnO and SnO_2_ are probably best applied in core–shell structures in DSCs, as discussed below. Table 1 lists alternative n-type semiconductor materials used in DSC that have obtained a PCE of more than 5%.

**Table 1:** Overview of different nanostructured metal oxide semiconductors used in DSC and their best performance in devices

Semiconductor	Bandgap (eV)	Nanostructure	Sensitizer – electrolyte	PCE (%)	Year	Ref.
TiO_2_ (anatase)	3.2	Mesoporous	ADEKA-1/LEG4 – Co(phen)_3_	14.3	2015	24
TiO_2_ (rutile)	3.0	Nanorod array	N719 – I^−^/I_3_^−^	11.1	2019	228
TiO_2_ (brookite)	3.2	Mesoporous	N719 – I^−^/I_3_^−^	8.2	2020	229
ZnO	3.2	Aggregated nanoparticles	N719 – I^−^/I_3_^−^	7.5	2011	230
SnO_2_	3.5	Nanoparticles/	N719 – I^−^/I_3_^−^	6.3	2013	231
Nb_2_O_5_	3.6	Nanorod array	N719 – I^−^/I_3_^−^	6.0	2013	231
Nb_3_O_7_(OH)	3.0	Nanorod array	N719 – I^−^/I_3_^−^	6.8	2013	231
Zn_2_SnO_4_	3.6	Aggregated nanoparticles	X73 – Co(phen)_3_	8.1	2020	232
BaSnO_3_	2.9	Mesoporous	N719 – I^−^/I_3_^−^	6.6	2019	233
Ba_0.8_Sr_0.2_SnO_3_	3.0	Mesoporous	N719 – I^−^/I_3_^−^	7.7	2019	233

**Fig. 18:**
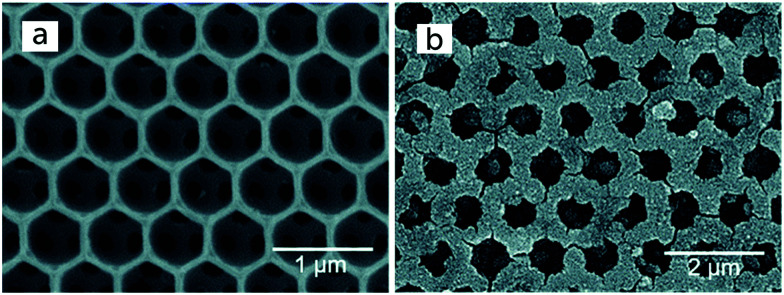
(a) Inverse opal SnO_2_ electrode; (b) after coating with a 170 nm shell of TiO_2_. Adapted from ref. 235 with permission from The Royal Society of Chemistry, copyright 2016.

Combinations of metal oxides have also been evaluated for DSC in a large number of studies. Scientifically most interesting are so-called core–shell structures, where a nanostructured electrode is covered by an ultra-thin layer of a different material, usually one with a higher bandgap. Deposition is performed by chemical bath deposition (using *e.g.* TiCl_4_ for deposition of TiO_2_) or by atomic layer deposition (ALD). The shell material can be a semiconductor or an insulator such as Al_2_O_3_ or SiO_2_: if sufficiently thin, adsorbed dyes can inject electrons into the core material through tunneling. Typically, rate constants for both electron injection and recombination are significantly reduced. This can lead to an improved solar cell efficiency if the injection efficiency is not significantly decreased. In addition, the shell can lead to added chemical stability (*e.g.* for Al_2_O_3_, SiO_2_, or TiO_2_ on ZnO). A few examples of core–shell structures will be given here: in ALD-deposited Al_2_O_3_ on mesoporous TiO_2_, the PCE increased from 6.2% to 8.4% upon 20 ALD cycles. This was partly caused by a higher recombination resistance and partly by a higher dye adsorption of the modified electrode.^234^ As another example, 3D-bicontinous inverse opal SnO_2_ structures were synthesized infiltrating a film of monodisperse polystyrene particles with SnCl_2_ in ethanol, followed by heating, see Fig. 18. A TiO_2_ shell was formed by chemical bath deposition using TiCl_4_. The resulting electrodes yielded an efficiency of 8.2% in DSCs, whereas TiO_2_/TiO_2_ inverse opal/shell structures yielded 7.2%.^235^

### 4.2 Sensitizers

Photoanodes based on molecular sensitizers at a semiconductor interface for DSCs require that the sensitizer absorbs solar energy and injects electrons into the semiconductor conduction band. Thus, the sensitizer controls the breadth of the solar spectrum used and the quantum yield for electron injection. Additionally, the sensitizer should promote long-lived charge separated states at the interface, and the oxidized sensitizer should rapidly undergo electron transfer from a reducing redox shuttle (RS) to limit the competitive electron back-transfer reaction from electrons in TiO_2_ to the oxidized dye. The sensitizer is also often tasked with providing insulating groups to protect electrons in TiO_2_ from recombining with the electrolyte. Recent progress in dye design with respect to these design criteria has fueled much of the increase observed in performance metrics. The atomistic level control with respect to dye design allows for the precise tuning of dye properties. One strategy that has been explored intensely is related to the design of a dye capable of absorbing photons across the visible spectrum and into the near infra-red (NIR) region to maximize the power conversion efficiency from a single photoanode-based device. Estimates of a practical efficiency limit at about 22% PCE are reported if driving forces for electron transfers to a semiconductor and from a redox shuttle to the oxidized dye can be kept to a combined 400 mV or less and the sensitizer can efficiently use photons as low in energy as ∼950 nm.^236^ Alternatively, an increasingly popular approach is to tailor chromophores to a specific spectral region to be used in co-sensitized or multiple-photoanode-based devices. This second approach increases the complexity of the device, but allows for higher theoretical PCEs. Using similar approximations of 400 mV free energies for electron transfers with the spectrum divided into three equal parts (wide, medium, and narrow optical gaps) from 400–950 nm leads to a practically possible PCE of ∼33%. Thus, significant gains in PCE are possible through research on multiple photoanode systems. Additionally, these materials are attractive for use with existing solar cell technologies as described below. For this strategy to work effectively, the sensitizer (and redox shuttle) needs to be custom tailored to each spectral region for minimal overpotential losses. Both single and multiple photoanode dye design approaches are discussed below with respect to both metal- and organic-based dyes. Notably, the literature with respect to dyes for DSCs is vast and growing rapidly with many exciting findings being reported weekly, which cannot all be highlighted (especially with regard to phthalocyanies, BODIPYs, DPP chromophores, multidonor systems, multiacceptor systems, dual anchor dyes, unique anchoring groups, and non-covalently bound dye–dye and dye-RS systems). The examples below serve to highlight recent select findings on high photocurrent, high photovoltage, deep NIR absorbing dyes, wide optical gap dyes, and high PCE dyes. Select design strategies being used within approximately the last decade are highlighted and should not be viewed as an exhaustive catalogue of dye design approaches.

#### 4.2.1 Metal coordination complexes

**Fig. 19:**
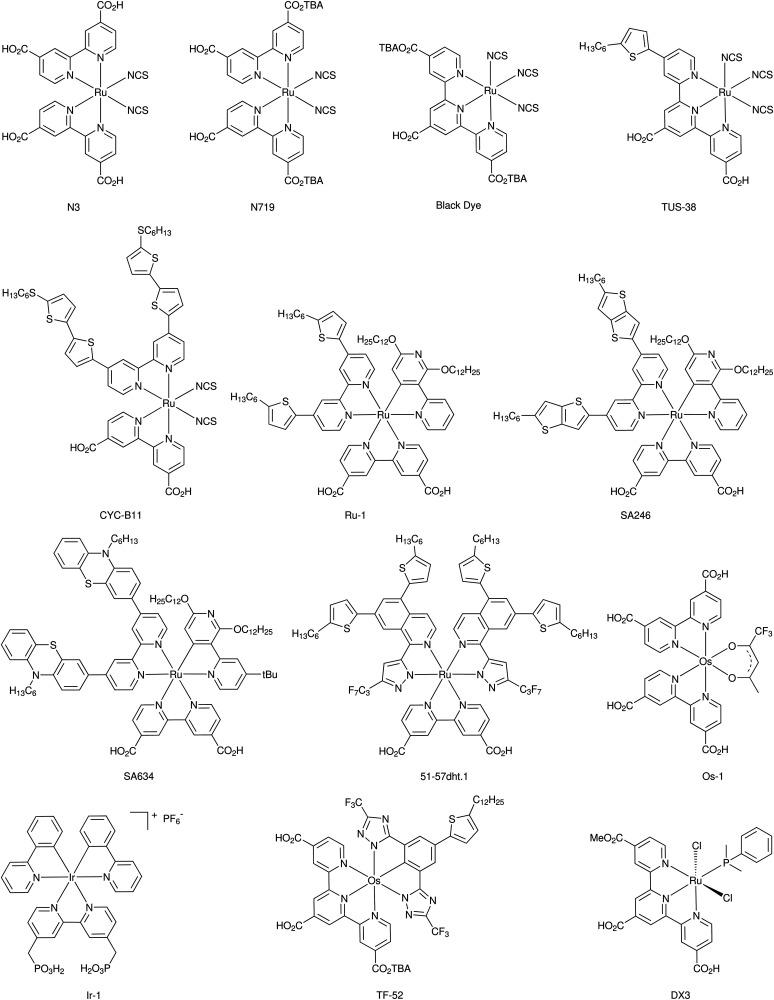
Examples of metal complex-based sensitizers.

Transition metal-based complexes were critical to the early development of DSCs and were the highest performing materials in the field for more than a decade after the modern mesoporous metal oxide construct inception. Dyes such as N3,^145^ N719,^212^ CYC-B11,^237^ and the Black Dye^238^ are commercial and remain common benchmarking materials in the DSC literature (Fig. 19). These dyes are used in a variety of DSC-based applications with many PCEs reported at >11%. Derivatives of these dyes such as TUS-38 – where a hexylthiophene replaces one of the three anchors of Black Dye – have shown further improved efficiencies (11.9% PCE).^239^ These dyes give excellent PCEs with the I^−^/I_3_^−^ redox shuttle; however, performances are generally diminished when the 1-electron metal-based redox shuttles, which have fueled the more recent increases in PCE to beyond 14%, are paired with metal-based dyes.^24^ TiO_2_ surface protection is generally considered to be lower with metal-based dyes, which often incorporate relatively few alkyl chains. These insulating alkyl groups have proven to be critical to sensitizer design with respect to organic dyes since they provide an umbrella type effect that slows electron transfers from the TiO_2_ surface to the electrolyte. Additional concerns about low metal-based sensitizer molar absorptivities arise due to reduced film thicknesses being used with transition metal-based RSs to limit TiO_2_ surface recombination sites and limit mass transport issues. Competitive electron transfer from the dye to the oxidizing RS directly rather than electron injection into the semiconductor conduction band have been noted as well.^240^ However, given that ultrafast electron transfer is often observed with transition metal-based sensitizers and the exceptionally broad IPCE spectrum that these materials can generate, the design of transition metal-based sensitizers that are compatible with Co and Cu RSs capable of high efficiency systems is an attractive area of research. Cyclometalated Ru complexes Ru-1, SA246, and SA634 incorporate four alkyl chains to insulate electrons in TiO_2_ from the electrolyte. This design leads to an 8.2–9.4% PCE with the use of a Co^3+/2+^ redox shuttle.^241–243^ The replacement of the NCS ligands commonly employed in the DSC literature on Ru complexes with the cyclometalated phenylpyridine-derived ligand leads to broad absorbing dyes with an IPCE onset near 800 nm. The incorporation of a pyrazolate-derived ligand onto a Ru complex with 6 alkyl chains gives dye 51–57dht.1.^244^ This complex was found to have good surface insulating properties leading to a PCE of 9.5% with a Co^3+/2+^ redox shuttle, which improved on the 9.1% PCE from a similar dye design.^245^ Given that the IPCE spectrum of many of these dyes is near 90% with the I^−^/I_3_^−^ RS and around 60–70% with Co^3+/2+^ RSs, systems that productively use the 20–30% of the IPCE spectrum not utilized with the Co^3+/2+^ shuttle are needed. The IPCE curve shape often resembles the absorption spectrum of the metal-based chromophore. This is typically only the case when regions of the absorption spectrum have a lower molar absorptivity and cannot efficiently absorb the available photons once the dye is anchored to a thin photoanode. Examples within the organic dye literature are discussed below where the IPCE does not resemble the absorption curve shape of these materials despite large valleys in the absorption spectrum. This is due to the absorption curve minima often sufficing to collect photons efficiently. However, metal-free dyes performing well with metal-based RSs have IPCE onsets that are 100–200 nm shifted to higher energies relative to broadly absorbing dyes such as N719. The blue-shift of organic sensitizers relative to transition metal-based systems lowers the possible photocurrent output from organic dyes; thus, strategies to boost the molar absorptivity and broaden the spectrum of 1-electron-compatible metal-based sensitizers are needed. Table 2 lists device parameters of DSCs fabricated with metal coordination complexes-based dyes referenced in this review, together with the electrolyte used.

**Table 2:** Photovoltaic characteristics of DSCs based on metal coordination complex dyes

Sensitizer	Electrolyte	Additives	*V*_OC_ (mV)	*J*_SC_ (mA cm^−2^)	FF (%)	PCE (%)	Year	Ref.
N719	I_2_, BMII	GuSCN, *t*BP	789	18.2	70.4	10.1	2008	212
CYC-B11	I_2_, LiI, DMII	GuSCN, *t*BP	743	20.05	77	11.5	2009	237
Black dye	I_2_, LiI, DMPII	*t*BP	727	20.43	72.4	10.75	2012	238
TUS-38	I_2_, LiI, EMII	*t*BP	702	23.43	72.2	11.88	2016	239
T7	I_2_, LiI, DMPII	*t*BP	760	16.7	70	8.9	2016	240
T7	Co(phen)_3_	LiClO_4_, *t*BP	800	10.1	70	5.7	2016	240
T5	I_2_, LiI, DMPII	*t*BP	680	19.5	67	8.9	2016	240
T5	Co(phen)_3_	LiClO_4_, *t*BP	670	4.05	52	1.4	2016	240
TF-1	I_2_, LiI, DMPII	*t*BP	670	16.7	68	7.7	2016	240
TF-1	Co(phen)_3_	LiClO_4_, *t*BP	570	6.85	39	1.5	2016	240
Ru-1	Co(phen)_3_	LiTFSI, *t*BP	837	13.2	78	8.6	2013	241
Ru-1	I_2_, LiI, PMII	GuSCN, *t*BP	715	16.3	75	8.7	2013	241
SA22	Co(phen)_3_	LiTFSI, NOP	827	12.25	75.5	7.9	2016	242
SA25	Co(phen)_3_	LiTFSI, NOP	810	10.68	77.9	6.9	2016	242
SA246	Co(phen)_3_	LiTFSI, NOP	845	14.55	74.7	9.4	2016	242
SA282	Co(phen)_3_	LiTFSI, NOP	794	9.89	78.5	6.3	2016	242
SA284	Co(phen)_3_	LiTFSI, NOP	794	11.28	76.9	7.0	2016	242
SA285	Co(phen)_3_	LiTFSI, NOP	807	11.85	73.6	7.2	2016	242
SA633	Co(phen)_3_	LiTFSI, *t*BP	819	13.68	71.5	8.0	2017	243
SA634	Co(phen)_3_	LiTFSI, *t*BP	845	13.89	70.0	8.2	2017	243
SA635	Co(phen)_3_	LiTFSI, *t*BP	809	13.03	72.1	7.6	2017	243
51–5ht	Co(bpy)_3_	LiTFSI, *t*BP	840	12.78	76.4	8.22	2016	244
51–5ht	Co(phen)_3_	LiTFSI, *t*BP	842	12.17	75.0	7.69	2016	244
51–5ht	I_2_, LiI, PMII	*t*BP	718	15.31	74.6	8.20	2016	244
51–57dht	Co(bpy)_3_	LiTFSI, *t*BP	844	13.56	74.2	8.49	2016	244
51–57dht	Co(phen)_3_	LiTFSI, *t*BP	898	12.32	75.4	8.34	2016	244
51–57dht	I_2_, LiI, PMII	*t*BP	727	14.17	74.3	7.66	2016	244
51–57dht.1	Co(bpy)_3_	LiTFSI, *t*BP	853	13.36	75.0	8.55	2016	244
51–57dht.1	Co(phen)_3_	LiTFSI, *t*BP	900	13.89	76.2	9.53	2016	244
51–57dht.1	I_2_, LiI, PMII	*t*BP	740	13.53	74.9	7.50	2016	244
TFRS-80a	Co(phen)_3_	LiTFSI, *t*BP	840	13.44	75.7	8.55	2014	245
TFRS-80a	I_2_, LiI, DMPII	*t*BP	780	14.49	66.8	7.55	2014	245
TFRS-80a	I_2_, DMPII	*t*BP	890	12.93	72.7	8.37	2014	245
TFRS-80b	Co(phen)_3_	LiTFSI, *t*BP	820	13.30	76.6	8.36	2014	245
TFRS-80b	I_2_, LiI, DMPII	*t*BP	680	10.39	68.1	4.80	2014	245
TFRS-80b	I_2_, DMPII	*t*BP	780	9.81	72.5	5.55	2014	245
TFRS-80c	Co(phen)_3_	LiTFSI, *t*BP	840	14.32	75.4	9.06	2014	245
TFRS-80c	I_2_, LiI, DMPII	*t*BP	730	14.84	65.1	7.06	2014	245
TFRS-80c	I_2_, DMPII	*t*BP	880	12.41	75.6	8.26	2014	245
Ir-1	Fe(bpy)_3_	*t*BP	870	0.014	48	0.60	2020	246
Os-1	I_2_, LiI, DMPII	None	320	23.7	36	2.7	2010	247
TF-5	I_2_, LiI, DMPII	*t*BP	640	18.0	71.6	8.25	2012	248
TF-51	I_2_, LiI, DMPII	*t*BP	560	20.1	66.4	7.47	2012	248
TF-52	I_2_, LiI, DMPII	*t*BP	600	23.3	63.3	8.85	2012	248
DX3	I_2_, LiI, DMPII	*t*BP	55 6	30.3	60.5	10.2	2015	249

Wide optical gap sensitizers are important for a number of applications and, within DSC literature, these systems are exceptionally valuable for use in multiple photoanode systems. With respect to these applications, generating a high photovoltage from the high-energy visible photons is critical to avoid thermal free energy losses. The overall PCE of the system is typically not the metric being pursued in these systems since they are often designed with tandem or multiple photoanode systems as the larger goal. Wide optical gap metal-based sensitizers are relatively rarely used in the literature with RSs capable of generating high photovoltages. This may in part be due to the higher photovoltage generating redox shuttles often being 1-electron metal-based RSs. As described above, the design of metal-based dyes that undergo efficient electron transfers with good charge separation lifetimes with metal-based RSs remains a key research direction. However, recently a cyclometalated Ir complex (Ir-1) based on two phenylpyridine ligands and a 4,4′-bis(phosphonomethyl)-2,2′-bipyridine ligand has been used in high photovoltage DSCs with the Fe(bpy)_3_^3+/2+^ redox shuttle to give 870 mV photovoltage under one-sun and 1.06 V under UV irradiation (Fig. 19).^246^

Narrow optical gap sensitizers are critical toward the use of lower energy photons in multiple photoanode-based devices (*e.g.* tandem solar cells). Within this region, the breadth of the IPCE spectrum (and *J*_SC_ generated) is a key performance metric with the goal being to combine these photoanodes into tandem-type systems. Metal-based sensitizers are exceptional in the >800 nm spectral region within DSC devices. Ru- and Os-based sensitizers specifically have shown exceptional deep NIR photon absorption and conversion properties. The ultrafast electron injection properties of these systems allows for efficient electron transfers prior to excited-state relaxation and therefore enables the efficient harvest of relatively low energy photons with minimal driving force needed for charge injection. Os-1 is a similar structure to N3 which uses two bipyridine-based ligands and a β-diketone in place of the NCS ligands of N3 (Fig. 19).^247^ Os-1 is broadly absorbing with an IPCE onset near 1100 nm and in excess of 70% across the visible spectrum. A PCE of 2.7% is reported which is low due to a poor *V*_OC_ (0.32 V) despite the high *J*_SC_ value of 23.7 mA cm^−2^. Os dye TF-52 was one of the first sensitizers to reach 1000 nm with a high peak IPCE (∼75%).^248^ A photocurrent of 23.3 mA cm^−2^ was reported with an efficiency of 8.85%. Light soaking at 60 °C with TF-52 reveals no significant change in PCE for this device over a 1000 hour measurement. Dye DX3 efficiently uses photons across the visible spectrum with an IPCE onset of ∼1100 nm. The peak IPCE value observed with this system is >80% with the IPCE remaining in excess of 80% from approximately 450 to 900 nm. A *J*_SC_ in excess of 30 mA cm^−2^ is observed from DSC devices using this dye. The deep NIR photon use of DX3 leads to the use of a DSC device made from this material in tandem with a perovskite solar cell with the DSC device being used as the narrow bandgap material (21.5% PCE tandem efficiency).^249^ These dyes are attractive for use in tandem type systems and represent the forefront of high percentage IPCE, broadly absorbing sensitizers. Design of sensitizers that retain high percentage IPCE values throughout the IPCE spectrum and extend IPCE wavelengths to beyond 1100 nm is an intriguing direction for this type of sensitizers that could have significant impact on tandem device designs.

#### 4.2.2 Organic sensitizers

**Fig. 20:**
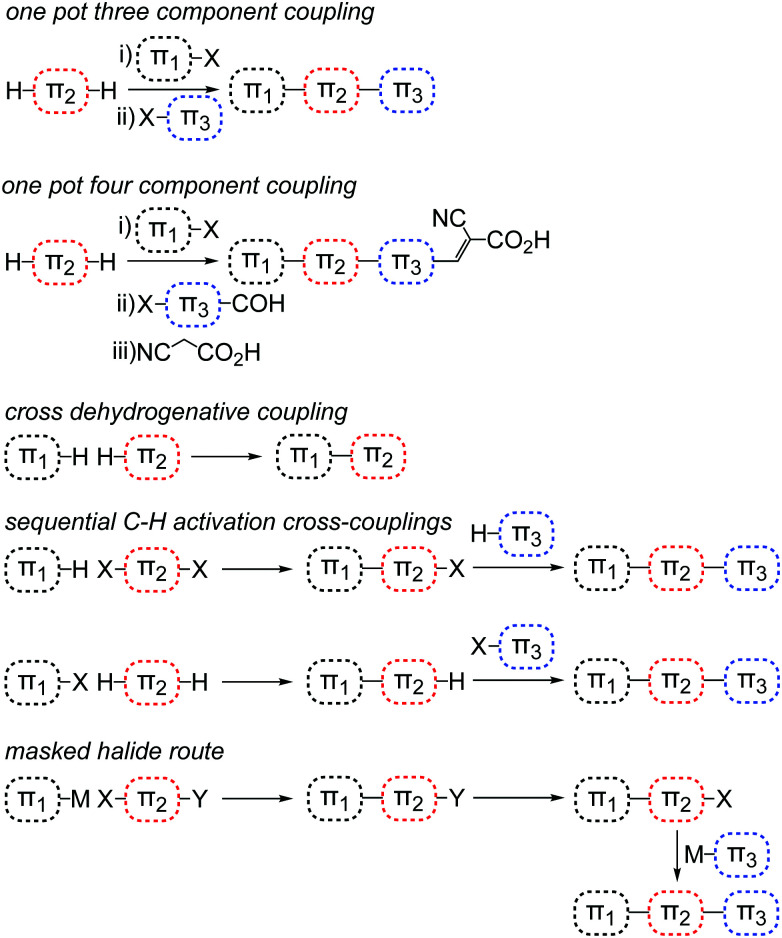
Contemporary rapid routes to complex organic dyes where X is a halide, M is a transmetallating reagent, and Y is a masked functionality such as a TMS group prior to halide conversion.

Organic dyes have been intensely explored within DSC devices over the last decade with progressively sophisticated designs giving a variety of chromophores tailored to probe various metrics. The demand for higher performing dyes for a range of DSC applications has been assisted by several notable synthetic approaches focused on rapid dye diversification strategies based on one-pot three-component couplings,^250^ one-pot four-component couplings,^251^ C–H activation-based cross couplings,^252,253^ sequential C–H activations,^254–259^ masked-halide approaches for sequential couplings,^260^ and cross-dehydrogenative couplings (Fig. 20).^261^ These types of contemporary routes in addition to traditional cross-couplings have in part fueled the rapid expansion of knowledge with regard to organic dyes in dye-sensitized systems. An infinite possibility for new dye designs exists, generally falling into two categories: intramolecular charge transfer (ICT) donor–acceptor type systems and inherent chromophore tuned systems. The donor–acceptor approach typically relies on building blocks which have little or no visible light absorption, but when combined can generate broadly absorbing dyes due to ICT events. The tunability of ICT systems relies primarily on adjusting electron donor or acceptor building block strengths. The inherent chromophore direction selects a molecule with desirable optical properties (*i.e.* porphyrins, phthalocyanines, squaraines, diketopyrrolopyrrole, BODIPY, *etc.*) and tunes the dye photophysical properties with added functionality. Both approaches utilize π-systems with increased or decreased conjugation lengths to adjust optical energy gaps. Both design approaches have found widespread use in the design of dye-sensitized systems with intriguing properties. Table 3 lists device parameters of DSCs fabricated with organic dyes referenced in this review, together with the electrolyte used.

**Table 3:** Photovoltaic characteristics of DSCs based on organic dyes

Sensitizer	Electrolyte	Additives	*V*_OC_ (mV)	*J*_SC_ (mA cm^−2^)	FF (%)	PCE (%)	Year	Ref.
D149	I_2_, LiI, BMII	*t*BP	644	19.86	69.4	8.85	2008	262
D205	I_2_, LiI, BMII	*t*BP	710	18.68	70.7	9.40	2008	262
WS-66	I_2_, LiI, DPMII	*t*BP	757	12.97	71	7.01	2017	263
WS-67	I_2_, LiI, DPMII	*t*BP	711	15.91	73	8.25	2017	263
WS-68	I_2_, LiI, DPMII	*t*BP	705	17.73	67	8.42	2017	263
WS-69	I_2_, LiI, DPMII	*t*BP	696	19.39	67	9.03	2017	263
IQ4	Co(bpy)_3_	LiClO_4_, *t*BP	771	14.69	68.8	7.79	2014	264
IQ4	I_2_, LiI, DMII	GuSCN, *t*BP	737	15.33	75.5	8.53	2014	264
YA421	Co(bpy)_3_	LiClO_4_, *t*BP	803	15.76	71.2	9.00	2014	264
YA421	I_2_, LiI, DMII	GuSCN, *t*BP	741	15.41	71.1	8.12	2014	264
YA422	Co(bpy)_3_	LiClO_4_, *t*BP	890	16.25	73.7	10.65	2014	264
YA422	I_2_, LiI, DMII	GuSCN, *t*BP	741	14.40	68.2	7.28	2014	264
DPP13	I_2_, LiI, DMII	GuSCN, *t*BP	705	16.2	67	7.60	2013	265
DPP13	Co(bpy)_3_	LiClO_4_, *t*BP	743	15.6	78	8.97	2013	265
DPP14	I_2_, LiI, DMII	GuSCN, *t*BP	680	16.6	68	7.73	2013	265
DPP14	Co(bpy)_3_	LiClO_4_, *t*BP	716	15.2	76	8.23	2013	265
DPP15	I_2_, LiI, DMII	GuSCN, *t*BP	684	16.9	65	7.44	2013	265
DPP15	Co(bpy)_3_	LiClO_4_, *t*BP	745	17.6	75	9.81	2013	265
DPP17	I_2_, LiI, DMII	GuSCN, *t*BP	700	16.3	63	7.13	2013	265
DPP17	Co(bpy)_3_	LiClO_4_, *t*BP	761	17.9	74	10.1	2013	265
D21L6	I_2_, LiI, DMII	GuSCN, *t*BP	714	13.81	72.1	7.11	2010	266
C218	I_2_, LiI, DMII	GuSCN, *t*BP	768	15.84	73.5	8.95	2010	266
AP25	I_2_, LiI, DMII	GuSCN, *t*BP	527	19.9	65	6.8	2020	267
PB1	I_2_, LiI, DMII	GuSCN, *t*BP	704	12.1	75	6.50	2016	268
PB2	I_2_, LiI, DMII	GuSCN, *t*BP	648	12.7	75	6.24	2016	268
DP1	I_2_, LiI, DMII	GuSCN, *t*BP	680	10.9	75	5.61	2016	268
DP2	I_2_, LiI, DMII	GuSCN, *t*BP	697	13.7	76	7.41	2016	268
C268	I_2_, DMII, EMII	sulfolane, NBB, GuSCN	718	16.76	72.3	8.7	2018	269
D35	Co(bpy)_3_	LiClO_4_, *t*BP	920	10.7	68	6.7	2010	270
D35	I_2_, LiI, TBAI	*t*BP	910	9.38	65	5.5	2010	270
Y123	I_2_, LiI, DMII	GuSCN, *t*BP	757	13.6	70	7.2	2011	271
Y123	Co(bpy)_3_	LiClO_4_, *t*BP	855	14.6	70	8.8	2011	271
Y123	Co(bpy-pz)_2_	LiClO_4_, *t*BP	1020	12.54	69.4	8.87	2012	272
Y123	Cu(tmby)_2_	LiTFSI, *t*BP	1030	13.6	74	10.3	2018	273
WS-70	Cu(tmby)_2_	LiTFSI, *t*BP	1060	13.2	77	11.0	2018	273
WS-72	Cu(tmby)_2_	LiTFSI, *t*BP	1100	13.3	78	11.6	2018	273
L348	Cu(tmby)_2_	LiTFSI	1170	6.4	72.0	5.3	2018	274
L349	Cu(tmby)_2_	LiTFSI	1160	11.0	71.7	9.2	2018	274
L350	Cu(tmby)_2_	LiTFSI	1140	13.0	76.0	11.2	2018	274
L351	Cu(tmby)_2_	LiTFSI	1060	11.2	76.3	9.1	2018	274
NT35	Cu(tmby)_2_	LiTFSI, MBI	950	5.96	79.1	4.5	2021	12
XY1b	Cu(tmby)_2_	LiTFSI, MBI	1010	15.26	76.3	11.8	2021	12
MS4	Cu(tmby)_2_	LiTFSI, MBI	1170	8.86	73.0	7.6	2021	12
MS5	Cu(tmby)_2_	LiTFSI, MBI	1240	8.87	73.3	8.0	2021	12
SC-1	Co(bpy)_3_	LiTFSI, *t*BP	828	14.70	76.2	9.3	2017	275
SC-2	Co(bpy)_3_	LiTFSI, *t*BP	856	16.62	74.5	10.6	2017	275
SC-3	Co(bpy)_3_	LiTFSI, *t*BP	920	16.50	75.8	11.5	2017	275
C272	Co(phen)_3_	LiTFSI, *t*BP	897	15.81	74.4	10.6	2015	276
C275	Co(phen)_3_	LiTFSI, *t*BP	956	17.03	77.0	12.5	2015	276
R4	Co(bpy)_3_	LiTFSI, *t*BP	852	17.25	75.4	11.1	2018	277
R6	Co(bpy)_3_	LiTFSI, *t*BP	850	19.69	75.4	12.6	2018	277
H1	Co(bpy)_3_	LiTFSI, *t*BP	931	14.33	72.3	9.7	2019	278
H2	Co(bpy)_3_	LiTFSI, *t*BP	903	15.47	74.0	10.3	2019	278
ZL001	Co(bpy)_3_	LiClO_4_, *t*BP	887	20.57	70.0	12.8	2019	279
ZL003	Co(bpy)_3_	LiClO_4_, *t*BP	956	20.73	68.5	13.6	2019	279
ADEKA-2	Co(bpy)_3_	LiClO_4_, *t*BP	821	15.1	75.2	9.32	2014	280
ADEKA-1	Co(bpy)_3_	LiClO_4_, *t*BP	848	16.1	76.2	10.4	2014	280
ADEKA-1	Co(Cl-phen)_3_	LiClO_4_, *t*BP, NaClO_4_, TBAPF_6_, TBPPF_6_, HMIPF_6_, TMSP, MP	1036	15.6	77.4	12.5	2014	280
SFD-5	Br_2_, BMIBr, TPABr	GuSCN, *t*BP	960	6.16	53	3.1	2016	216
ADEKA-3	Br_2_, BMIBr, TPABr	GuSCN, *t*BP, TMSP, MP, H_2_O	1450	4.77	56	3.9	2016	216
AP11	Fe(bpy)_3_	LiTFSI, *t*BP	1260	3.50	63	2.9	2019	281
AP14	Fe(bpy)_3_	LiTFSI, *t*BP	1320	3.40	63	2.7	2019	281
AP16	Fe(bpy)_3_	LiTFSI, *t*BP	1290	3.10	65	2.6	2019	281
AP17	Fe(bpy)_3_	LiTFSI, *t*BP	1270	2.90	58	2.2	2019	281
RR9	Fe(bpy)_3_	LiTFSI, *t*BP	1420	2.8	47	1.9	2018	282
YD2	I_2_, LiI, DMII	GuSCN, *t*BP	770	1 8.6	7 6.4	11	2010	283
YD2	Co(bpy)_3_	LiClO_4_, *t*BP	825	14.9	69	8.4	2011	284
YD2-o-C8	Co(bpy)_3_	LiClO_4_, *t*BP	965	17.3	71	11.9	2011	284
GY21	Co(bpy)_3_	Not specified	615	5.03	79.8	2.52	2014	285
GY21	I_2_, PMII	LiTFSI, *t*BP	552	11.50	75.1	4.84	2014	285
GY50	Co(bpy)_3_	Not specified	885	18.53	77.3	12.75	2014	285
GY50	I_2_, PMII	LiTFSI, *t*BP	732	18.45	65.7	8.90	2014	285
SM371	Co(bpy)_3_	LiTFSI, *t*BP	960	15.9	79	12.0	2014	286
SM315	Co(bpy)_3_	LiTFSI, *t*BP	910	18.1	78	13.0	2014	286
SGT-020	Co(bpy)_3_	LiClO_4_, *t*BP	825	15.6	7 4.7	9.6	2017	287
SGT-021	Co(bpy)_3_	LiClO_4_, *t*BP	819	17.9	75.4	1 1.1	2017	287
SGT-130	Co(bpy)_3_	LiClO_4_, *t*BP	810	16.84	72.08	9.83	2017	288
SGT-136	Co(bpy)_3_	LiClO_4_, *t*BP	804	18.35	74.84	11.04	2017	288
SGT-137	Co(bpy)_3_	LiClO_4_, *t*BP	825	19.39	73.98	11.84	2017	288
SGT-137	I_2_, LiI, DMPII	*t*BP	690	18.55	68.9	8.9	2020	25
SGT-146	Co(bpy)_3_	LiTFSI, *t*BP	834	16.39	74.6	10.2	2020	25
SGT-146	I_2_, LiI, DMPII	*t*BP	674	18.54	72.9	9.2	2020	25
SGT-147	Co(bpy)_3_	LiTFSI, *t*BP	839	17.15	73.5	10.5	2020	25
SGT-147	I_2_, LiI, DMPII	*t*BP	702	18.46	67.6	8.8	2020	25
SGT-148	Co(bpy)_3_	LiTFSI, *t*BP	849	17.12	72.9	10.6	2020	25
SGT-148	I_2_, LiI, DMPII	*t*BP	698	18.71	68.4	8.9	2020	25
SGT-149	Co(bpy)_3_	LiTFSI, *t*BP	898	17.49	72.2	11.4	2020	25
SGT-149	I_2_, LiI, DMPII	*t*BP	713	19.32	71.1	9.8	2020	25
SM63	I_2_, LiI, DMII	GuSCN, *t*BP	700	14.43	73	7.35	2016	289
LD14-C8	I_2_, LiI, DMII	GuSCN, *t*BP	730	15.72	74	8.45	2016	289
WW-3	Co(bpy)_3_	LiTFSI, *t*BP	744	9.81	76.7	5.6	2014	290
WW-4	Co(bpy)_3_	LiTFSI, *t*BP	500	3.00	29.9	0.3	2014	290
WW-5	Co(bpy)_3_	LiTFSI, *t*BP	766	18.87	73.3	10.3	2014	290
WW-6	Co(bpy)_3_	LiTFSI, *t*BP	840	17.16	73.8	10.6	2016	291
WW-7	Co(bpy)_3_	LiTFSI, *t*BP	708	8.05	77.7	4.4	2016	291
WW-8	Co(bpy)_3_	LiTFSI, *t*BP	733	8.27	78.6	4.8	2016	291
WW-9	Co(bpy)_3_	LiTFSI, *t*BP	770	15.93	75.2	9.2	2016	291
YD22	I_2_, LiI, PMII	*t*BP	700	14.92	72.43	7.56	2016	292
YD23	I_2_, LiI, PMII	*t*BP	740	17.10	71.41	9.00	2016	292
YD24	I_2_, LiI, PMII	*t*BP	730	17.29	72.46	9.19	2016	292
YD25	I_2_, LiI, PMII	*t*BP	720	15.22	72.66	7.93	2016	292
YD26	I_2_, LiI, PMII	*t*BP	790	15.26	73.24	8.79	2016	292
YD27	I_2_, LiI, PMII	*t*BP	790	15.45	73.07	8.92	2016	292
YD28	I_2_, LiI, PMII	*t*BP	760	14.07	70.60	7.58	2016	292
XW1	I_2_, LiI, PMII	*t*BP	716	14.99	66	7.13	2014	293
XW2	I_2_, LiI, PMII	*t*BP	680	15.73	64	6.84	2014	293
XW3	I_2_, LiI, PMII	*t*BP	694	15.60	68	7.32	2014	293
XW4	I_2_, LiI, PMII	*t*BP	702	16.22	70	7.94	2014	293
C1	I_2_, LiI, PMII	*t*BP	780	11.21	65	5.67	2014	293
XW9	I_2_, LiI, PMII	*t*BP	740	16.17	68.9	8.2	2015	294
XW10	I_2_, LiI, PMII	*t*BP	739	17.51	68.0	8.8	2015	294
XW11	I_2_, LiI, PMII	*t*BP	727	18.26	70.1	9.3	2015	294
XW14	I_2_, LiI, PMII	*t*BP	725	17.07	70	8.6	2015	295
XW15	I_2_, LiI, PMII	*t*BP	720	18.02	67	8.7	2015	295
XW16	I_2_, LiI, PMII	*t*BP	734	17.92	70	9.1	2015	295
XW17	I_2_, LiI, PMII	*t*BP	700	18.79	72	9.5	2015	295
SGT-021	Co(bpy)_3_	LiTFSI, *t*BP	848	1 6.9	7 5.8	1 0.8	2019	296
SGT-023	Co(bpy)_3_	LiTFSI, *t*BP	739	3.4	79.5	2.0	2019	296
SGT-025	Co(bpy)_3_	LiTFSI, *t*BP	8 19	1 4.1	7 8.4	9.1	2019	296
XW26	I_2_, LiI, PMII	*t*BP	708	11.37	69.13	5.57	2017	297
XW27	I_2_, LiI, PMII	*t*BP	710	14.08	72.26	7.17	2017	297
XW28	I_2_, LiI, PMII	*t*BP	715	19.38	72.96	10.14	2017	297
LG1	I_2_, LiI, DMII	*t*BP	710	17.43	71	8.89	2017	298
LG2	I_2_, LiI, DMII	*t*BP	710	15.45	72	7.87	2017	298
LG3	I_2_, LiI, DMII	*t*BP	710	12.10	72	6.17	2017	298
LG4	I_2_, LiI, DMII	*t*BP	710	15.02	68	7.30	2017	298
LG5	I_2_, LiI, DMII	*t*BP	680	21.01	71	10.20	2017	298
LG6	I_2_, LiI, DMII	*t*BP	690	19.55	71	9.64	2017	298
LG7	I_2_, LiI, DMII	*t*BP	660	13.38	69	6.21	2017	298
ZZX-N7	I_2_, LiI, DMII	GuSCN, *t*BP	732	15.39	63.33	7.51	2015	299
ZZX-N8	I_2_, LiI, DMII	GuSCN, *t*BP	741	14.25	69.97	7.78	2015	299
ZZX-N9	I_2_, LiI, DMII	GuSCN, *t*BP	656	15.46	70.57	7.53	2015	299
YD2-o-C8T	I_2_, LiI, DMII	GuSCN, *t*BP	730	15.6	68	7.7	2015	300
YD2-o-C8	I_2_, LiI, DMII	GuSCN, *t*BP	780	17.3	65	8.8	2015	300
PZn-HOQ	I_2_, LiI, DPMII	GuSCN, *t*BP	576	6.48	67.8	2.53	2014	301
DPZn-HOQ	I_2_, LiI, DPMII	GuSCN, *t*BP	595	7.81	66.4	3.09	2014	301
DPZn-COOH	I_2_, LiI, DPMII	GuSCN, *t*BP	602	4.22	69.4	1.76	2014	301
mJS1	Co(bpy)_3_	LiTFSI, *t*BP	833	10.55	76.2	6.69	2021	302
mJS2	Co(bpy)_3_	LiTFSI, *t*BP	845	5.47	75.2	3.48	2021	302
mJS3	Co(bpy)_3_	LiTFSI, *t*BP	814	3.73	76.8	2.33	2021	302
bJS1	Co(bpy)_3_	LiTFSI, *t*BP	823	12.52	77.9	8.03	2021	302
bJS2	Co(bpy)_3_	LiTFSI, *t*BP	849	16.59	75.9	10.69	2021	302
bJS3	Co(bpy)_3_	LiTFSI, *t*BP	836	16.48	75.5	10.42	2021	302
LWP12	Co(bpy)_3_	LiTFSI, *t*BP	731	12.07	73.8	6.5	2016	303
LWP13	Co(bpy)_3_	LiTFSI, *t*BP	706	10.06	78.0	5.5	2016	303
LWP14	Co(bpy)_3_	LiTFSI, *t*BP	805	17.22	74.1	10.3	2016	303
SM85	I_2_, LiI, DMII	GuSCN, *t*BP	578	13.4	71	5.7	2019	304
H2PE1	I_2_, LiI, PMII	*t*BP	540	5.26	73	2.06	2017	305
LS-01	I_2_, LiI, PMII	*t*BP	530	12.58	70	4.67	2017	305
LS-11	I_2_, LiI, PMII	*t*BP	520	16.13	64	5.36	2017	305
XW40	I_2_, LiI, PMII	*t*BP	730	18.67	68.3	9.3	2019	306
XW48	I_2_, LiI, PMII	*t*BP	755	18.34	70.2	9.7	2019	306
XW48	Co(bpy)_3_	LiTFSI, *t*BP	803	15.20	73.2	8.9	2019	306
XW49	I_2_, LiI, PMII	*t*BP	753	18.09	69.6	9.5	2019	306
XW49	Co(bpy)_3_	LiTFSI, *t*BP	837	15.60	72.9	9.5	2019	306
XW50	I_2_, LiI, PMII	*t*BP	761	18.96	70.2	10.1	2019	306
XW50	Co(bpy)_3_	LiTFSI, *t*BP	843	16.24	73.9	10.1	2019	306
XW51	I_2_, LiI, PMII	*t*BP	781	20.07	70.2	11.1	2019	306
XW51	Co(bpy)_3_	LiTFSI, *t*BP	844	15.24	75.6	9.7	2019	306
XW41	I_2_, LiI, PMII	*t*BP	695	16.77	70.1	8.16	2019	307
XW60	I_2_, LiI, PMII	*t*BP	715	16.77	73.1	8.8	2020	308
XW61	I_2_, LiI, PMII	*t*BP	775	21.41	74.7	12.4	2020	308
XW62	I_2_, LiI, PMII	*t*BP	762	20.70	73.2	11.6	2020	308
XW63	I_2_, LiI, PMII	*t*BP	763	20.63	73.7	11.6	2020	308
ISQ1	Iodolyte Z-50		544	8.99	68.4	3.34	2018	309
ISQ2	Iodolyte Z-50		558	9.62	68.7	3.68	2018	309
ISQ3	Iodolyte Z-50		576	10.02	72.0	4.15	2018	309
SQ1	Iodolyte Z-50		579	8.33	71.1	3.43	2016	310
sQ2	Iodolyte Z-50		649	12.56	71.5	5.8	2016	310
SQ3	Iodolyte Z-50		606	9.05	69.8	3.83	2016	310
SQ4	Iodolyte Z-50		622	10.10	68.7	4.31	2016	310
SQ5	Iodolyte Z-50		660	19.82	68.9	9.0	2016	310
SQ6	Iodolyte Z-50		648	14.20	68.5	6.30	2016	310
SQ7	Iodolyte Z-50		646	16.67	69.9	7.53	2016	310
YR1	I_2_, LiI, DMII	GuSCN, *t*BP	524	2.88	69	1.04	2013	311
YR2	I_2_, LiI, DMII	GuSCN, *t*BP	563	2.77	73	1.14	2013	311
YR3	I_2_, LiI, DMII	GuSCN, *t*BP	604	7.26	74	3.27	2013	311
YR4	I_2_, LiI, DMII	GuSCN, *t*BP	613	8.53	74	3.85	2013	311
YR5	I_2_, LiI, DMII	GuSCN, *t*BP	605	7.80	74	3.49	2013	311
YR6	I_2_, LiI, DMII	GuSCN, *t*BP	642	14.8	71	6.74	2013	311
TS3	I_2_, LiI, DMII	GuSCN, *t*BP	622	13.1	73	5.95	2013	311
JD10	I_2_, LiI, DMII	GuSCN, *t*BP	635	16.4	70	7.30	2013	311
T-PA	I_2_, LiI, DMII	GuSCN, *t*BP	644	9.6	72.2	4.6	2015	312
DTP-PA	I_2_, LiI, DMII	GuSCN, *t*BP	642	5.9	73.5	2.8	2015	312
DTT-CA	I_2_, LiI, DMII	GuSCN, *t*BP	644	13.1	71.6	6.0	2015	312
DTT-PA	I_2_, LiI, DMII	GuSCN, *t*BP	621	3.7	76.3	1.8	2015	312
DTS-CA	I_2_, LiI, DMII	GuSCN, *t*BP	682	19.1	68.3	8.9	2015	312
DTS-PA	I_2_, LiI, DMII	GuSCN, *t*BP	676	10.4	70.5	5.0	2015	312
PBut-SC2-T	I_2_, LiI, DMII	GuSCN, *t*BP	650	13.4	70.4	6.1	2015	313
PBut-SC12-T	I_2_, LiI, DMII	GuSCN, *t*BP	660	16.3	70.1	7.5	2015	313
PSil-SC12-T	I_2_, LiI, DMII	GuSCN, *t*BP	650	15.2	71.2	7.1	2015	313
PSil-SC12-DTS	I_2_, LiI, DMII	GuSCN, *t*BP	690	16.0	69.6	7.6	2015	313
TSQa	I_2_, LiI, DMPII	None	450	8.05	59	2.13	2013	314
TSQb	I_2_, LiI, DMPII	None	450	8.89	61	2.43	2013	314
MSQ	I_2_, LiI, DMPII	None	520	5.25	69	1.88	2013	314
JK-216	I_2_, LiI, DMPII	*t*BP	610	13.93	74.0	6.29	2011	315
JK-217	I_2_, LiI, DMPII	*t*BP	583	13.73	70.2	5.54	2011	315
WCH-SQ10	I_2_, LiI	None	374	9.25	51	1.77	2012	316
WCH-SQ11	I_2_, LiI	None	391	9.06	55	1.96	2012	316
PSQ9	Iodolyte Z-50		577	17.07	70.35	6.93	2019	317
PSQ10	Iodolyte Z-50		579	16.93	69.83	6.84	2019	317
HSQ2	I_2_, LiI, DMPII	None	584	11.55	61	4.11	2014	318
HSQ3	I_2_, LiI, DMPII	None	581	13.95	57	4.60	2014	318
HSQ4	I_2_, LiI, DMPII	None	558	15.61	65	5.66	2014	318
SPSQ1	I_2_, LiI, DMPII	*t*BP	627	6.51	73	2.98	2016	319
SPSQ2	I_2_, LiI, DMPII	*t*BP	670	7.94	74	3.95	2016	319
L1	Cu(tmby)_2_	LiTFSI, *t*BP	910	9.4	71	6.1	2020	26
WS-68/WS-5	I_2_, LiI, DPMII	*t*BP	746	14.08	67	7.67	2017	263
WS-5/WS-69	I_2_, LiI, DPMII	*t*BP	753	19.56	68	10.09	2017	263
AP25/D35	I_2_, LiI, DMII	GuSCN, *t*BP	551	24.5	63	8.4	2020	267
C268/SC-4	I_2_, DMII, EMII	Sulfolane, NBB, GuSCN	779	18.10	71.0	10.0	2018	269
XY1b/Y123	Cu(tmby)_2_	LiTFSI, MBI	1050	15.74	79	13.1	2018	320
MS5/XY1b	Cu(tmby)_2_	LiTFSI, MBI	1050	15.84	81.3	13.5	2021	12
ADEKA-1/LEG4	Co(phen)_3_	LiClO_4_, NaClO_4_, TBAPF_6_, TBPPF_6_, HMIPF_6_, *t*BP, TMSP, MP, CPrBP, CPeBP, COcBP	1014	18.27	77.1	14.3	2015	24
ADEKA-1/SFD-5	Co(phen)_3_	LiClO_4_, NaClO_4_, TBAPF_6_, TBPPF_6_, HMIPF_6_, *t*BP, TMSP, MP	1035	16.07	77.3	12.86	2015	321
SGT-020/HC-A4	Co(bpy)_3_	LiClO_4_, *t*BP	864	15.8	76.6	10.5	2017	287
SM315/HC-A4	Co(bpy)_3_	LiClO_4_, *t*BP	893	16.4	79.4	11.6	2017	287
SGT-021/HC-A4	Co(bpy)_3_	LiClO_4_, *t*BP	910	17.5	75.3	12.0	2017	287
SGT-137/HC-A1	Co(bpy)_3_	LiClO_4_, *t*BP	884	18.37	76.7	12.45	2017	288
XW1/C1	I_2_, LiI, PMII	*t*BP	746	17.53	71	9.24	2014	293
XW2/C1	I_2_, LiI, PMII	*t*BP	697	18.22	70	8.96	2014	293
XW3/C1	I_2_, LiI, PMII	*t*BP	705	18.42	70	9.05	2014	293
XW4/C1	I_2_, LiI, PMII	*t*BP	736	20.15	71	10.45	2014	293
XW9/C1	I_2_, LiI, PMII	*t*BP	764	17.01	71.8	9.3	2015	294
XW10/C1	I_2_, LiI, PMII	*t*BP	753	18.24	74.2	10.1	2015	294
XW11/C1	I_2_, LiI, PMII	*t*BP	746	19.52	74.0	10.6	2015	294
XW9/WS-5	I_2_, LiI, PMII	*t*BP	770	17.70	74.1	10.1	2015	294
XW10/WS-5	I_2_, LiI, PMII	*t*BP	765	19.01	76.4	11.0	2015	294
XW11/WS-5	I_2_, LiI, PMII	*t*BP	760	20.33	74.4	11.5	2015	294
XW14/WS-5	I_2_, LiI, PMII	*t*BP	765	18.54	70	9.9	2015	295
XW15/WS-5	I_2_, LiI, PMII	*t*BP	763	18.88	71	10.1	2015	295
XW16/WS-5	I_2_, LiI, PMII	*t*BP	773	19.01	72	10.4	2015	295
XW17/WS-5	I_2_, LiI, PMII	*t*BP	748	20.30	72	10.9	2015	295
SGT-021/HC-A1	Co(bpy)_3_	LiTFSI, *t*BP	849	19.2	76.8	12.6	2019	296
SGT-023/HC-A1	Co(bpy)_3_	LiTFSI, *t*BP	761	9.2	79.9	5.6	2019	296
SGT-025/HC-A1	Co(bpy)_3_	LiTFSI, *t*BP	837	17.3	76.0	11.0	2019	296
PZn-HOQ/BET	I_2_, LiI, DPMII	GuSCN, *t*BP	573	6.87	66.8	2.63	2014	301
PZn-HOQ/BET	I_2_, LiI, DPMII	GuSCN, *t*BP	605	8.33	67.7	3.41	2014	301
XW40/Z1	I_2_, LiI, PMII	*t*BP	748	19.59	71.9	10.55	2019	307
XW41/Z1	I_2_, LiI, PMII	*t*BP	726	19.63	71.5	10.19	2019	307
XW51/Z2	I_2_, LiI, PMII	*t*BP	738	20.13	7 0.5	10.5	2020	308
TSQa/MSQ	I_2_, LiI, DMPII	None	440	11.57	56	2.82	2013	314
SPSQ1/N3	I_2_, LiI, DMPII	*t*BP	635	15.60	73	7.20	2016	319
SPSQ2/N3	I_2_, LiI, DMPII	*t*BP	656	17.10	73	8.20	2016	319
XY1/L1	Cu(tmby)_2_	LiTFSI, *t*BP	1080	15.9	67	11.5	2020	26
XY1/D35	Cu(tmby)_2_	LiTFSI, *t*BP	1070	15.3	67	11.0	2020	26
D35/Dyenamo blue	Co(bpy)_3_	LiClO_4_, *t*BP, TPAA	920	15.5	73.3	10.5	2016	322
SGT-149/SGT-021	Co(bpy)_3_	LiTFSI, *t*BP	912	20.86	73.2	13.9	2020	25
SGT-149/SGT-021	I_2_, LiI, DMPII	*t*BP	722	22.05	70.6	11.3	2020	25

**Fig. 21:**
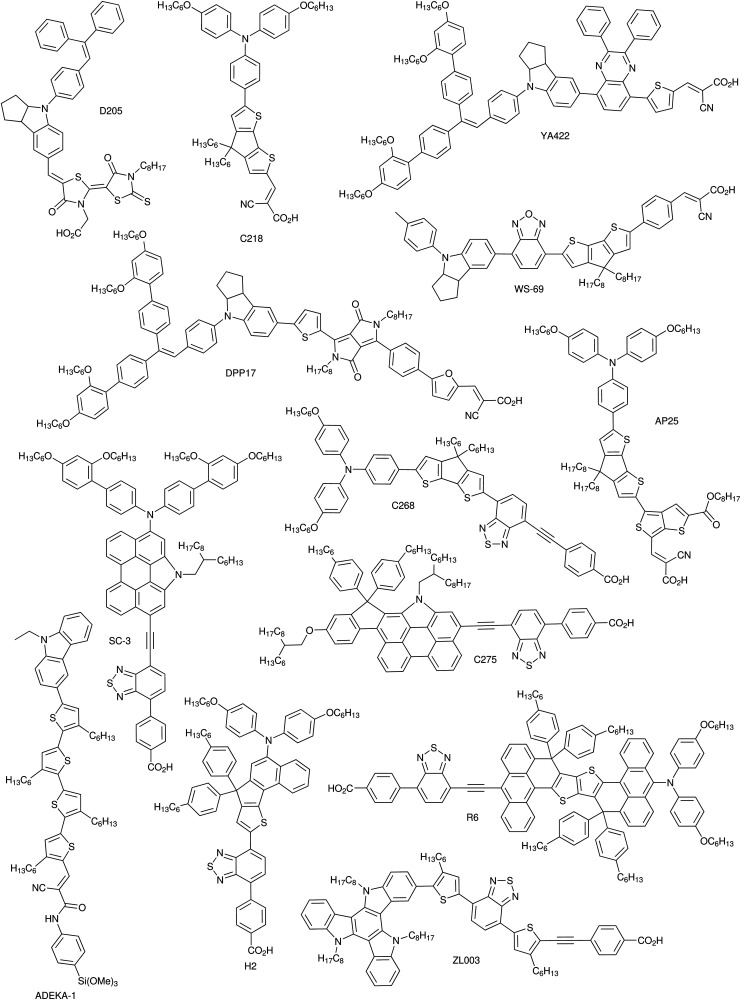
Examples of high-performing organic charge transfer dyes used in DSC devices.

The highest performing DSC dyes are typically based on amine donors.^323^ These groups are tunable in donation strength, offer reversible oxidation potentials, and have multiple positions for addition of insulating groups. Indoline-based donor dyes have been a popular class of materials in the DSC literature. Relatively early success with indoline use in an organic dye was found when D205 demonstrated a PCE of 9.4% as a donor–acceptor (D–A) dye design with a rhodanine acceptor (Fig. 21).^262^ This PCE value was reported to be the highest observed for an organic dye at the time and fueled wide-spread use of the indoline donor with varied π-bridges and acceptors. WS-69 uses an indoline donor group along benzoxa diazole (BOD), cyclopentadithiophene (CPDT), and phenyl-cyanoacrylic acid (CAA) moieties to generate a device with an IPCE onset nearing 800 nm, which resulted in a *J*_SC_ of 19.4 mA cm^−2^ and a PCE of 9% as a single dye device.^263^ The use of indoline in a donor–π–bridge–acceptor (D–π–A) design allowed expansion of the IPCE onset from 700 nm with D205 to 800 nm with WS-69. A PCE in excess of 10% could be obtained when co-sensitization strategies were employed with WS-69. Increasing the bulk of the indoline donor used with D205 and utilizing a D–A′–π–A design with a quinoxaline auxiliary acceptor gives dye YA422.^264^ The increased bulk of the donor group led to a dye compatible with a Co-based electrolyte for a PCE of 10.7% without an added co-sensitizer. The use of the same donor on YA422 on a diketopyrrolopyrrole (DPP)-based dye (DPP17) again lead to a >10% PCE device with a bright blue chromophore valuable for aesthetic applications.^265^

**Fig. 22:**
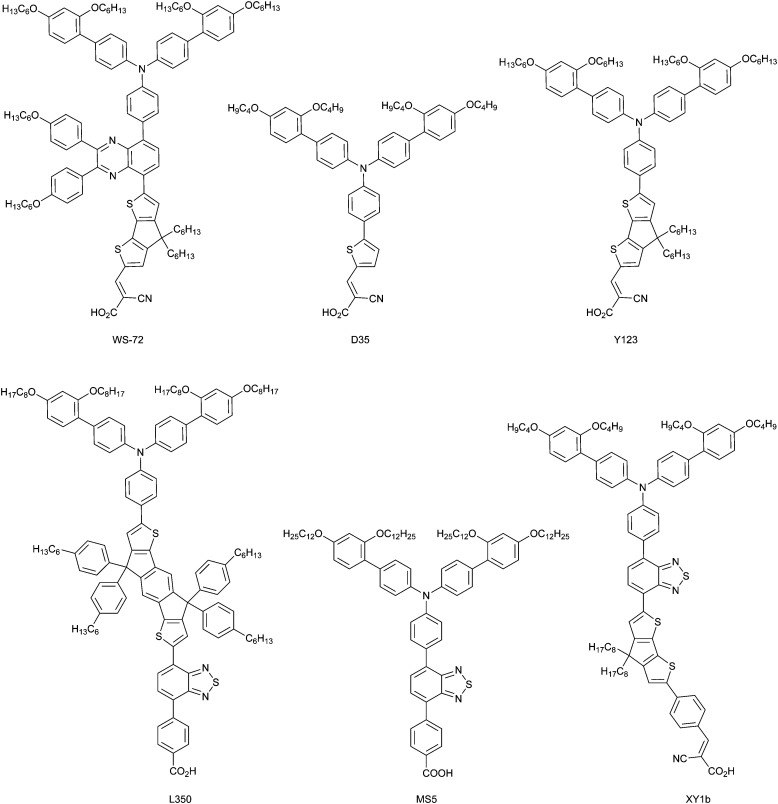
Examples of high-performing organic charge transfer dyes used in DSC devices with “umbrella” type donors.

One of the most popular classes of amine donors used in dye design is based on triarylamines (TAAs). TAAs are typically stable and the symmetric aryl groups, before conjugation with the acceptor, allow for ease of incorporation of alkyl chains in multiple dimensions. C218 is a TAA donor-based dye with a CPDT π-bridge and a CAA acceptor which demonstrated a ∼9.0% PCE with an IPCE onset near 700 nm (Fig. 21). In ionic liquid-based devices, exceptional stabilities were noted with nearly no loss in performance under full sun soaking conditions at 60 °C.^266^ A 3,4-thienothiophene (3,4-TT) group was inserted between the CPDT and CAA groups of C218 to give AP25.^267^ The 3,4-TT building block is proaromatic by valence bond theory upon ICT, and excited-state aromaticity is observed computationally.^268^ Proaromatic groups allow for lower energy excitations, which enables the use of lower energy NIR photons. An exceptional photocurrent (*J*_SC_ = 25 mA cm^−2^) for an organic dye-based DSC device was reported when AP25 (Fig. 21) was co-sensitized with D35 (Fig. 22). AP25-based DSC devices have an IPCE onset of 900 nm with a peak value of near 90% and the D35-co-sensitized devices showed a PCE of 8.4%. The broad IPCE of the AP25-based DSC device is attractive for use as a narrow optical gap material in tandem and sequential series multijunction (SSM) systems,^324–326^ yielding DSC devices with PCEs exceeding 10% for both the two and three photoanode devices with an up to 2.1 V open circuit voltage. Replacing the CAA group of C218 with a BTD and a benzoic acid linked with an alkyne group gives C268, which has an IPCE onset red-shifted by 50 nm relative to C218.^269^ C268 was shown to densely pack on the surface of TiO_2_ with a co-sensitizer, which enabled the fabrication of possibly the first >10% PCE ionic liquid-based DSC device. Exceptional stability of ionic liquid-based C268 DSC devices is reported during light soaking at 60 °C or at 85 °C when thermally stressed.

Amine donor group design has given rise to some of the highest performance DSC devices by enabling the use of 1-electron redox shuttles typically based on Co^3+/2+^ and Cu^2+/+^.^95,327^ For these positively charged 1-electron redox shuttles to facilitate productive electron transfers within the DSC device, exquisite surface protection is needed to slow the recombination reaction of electrons in TiO_2_ with the oxidized redox shuttle. The most common successful strategy employed with respect to dye design is the use of alkylated donor groups with alkyl chains extending in three dimensions to provide an “umbrella” of insulating groups to protect electrons at the TiO_2_ surface. One of the first and most widely used materials to demonstrate this concept is the dye D35, which illustrated the benefits of Co^3+/2+^ redox shuttles relative to I^−^/I_3_^−^ (Fig. 22).^270^ The thiophene π-bridge of D35 was expanded to a CPDT π-bridge to give Y123 with the same CAA acceptor.^271,272^ The expansion of the π-bridge conjugation length gave a red-shift of the absorption spectrum and allowed for an increase in PCE from 6.7 to 8.8% based on a cobalt redox shuttle. Building from the D35/Y123 D–π–A design, an auxiliary acceptor (A′) strategy was employed with dye WS-72 by insertion of a quinoxaline group between the TAA donor and the CPDT bridge to give a D–A′–π–A design (Fig. 22).^273^ The D–A′–π–A dye design is reported to enable more favorable electron transfers with extended charge separation durations while red-shifting the absorption spectrum relative to the D–π–A design.^328^ The D–A′–π–A design often showed not to lower the ground state oxidation potential value significantly despite extending conjugation, which allowed for the continued use of RSs with more positive values in DSC devices for an increase in the theoretical *V*_OC_. WS-72 was found to minimize voltage losses when paired with the bis-(4,4′,6,6′-tetramethyl-2,2′-bipyridine)copper(ii/i) ([Cu(tmby)_2_]^2+/+^) redox shuttle leading to an 11.6% PCE DSC device with a *V*_OC_ in excess of 1.1 V. The same device and redox shuttle could be solidified to give a solid-state device operating at 11.7% PCE, which is claimed to be the highest known solid-state DSC PCE at the time of the report. L350 uses an indacenodithiophene (IDT) π-bridge with a similar donor group to Y123 and a benzothiadiazole (BTD)-benzoic acid acceptor.^274^ This design led to a positive ground state oxidation potential (1.04 V *vs.* NHE) which allowed for the use of the [Cu(tmby)_2_]^2+/+^ redox shuttle system to give a 1.14 V open-circuit voltage solar cell for a PCE of 11.2% under full sun conditions. Under low light conditions (1000 lux), an impressive PCE of 28.4% could be obtained. Interestingly, L350 has an optical energy gap of 1.82 eV as estimated from the IPCE onset, which indicates that only 680 mV of total absorbed energy was required to drive both the electron transfer to TiO_2_ and the regeneration reaction from the redox shuttle. XY1b uses a similar design to that of dye WS-72 with a BTD group in place of the quinoxaline group and a phenyl spacer between the CPDT and CAA groups. Through the use of XY1b, co-sensitizer Y123, redox shuttle [Cu(tmby)_2_]^2+/+^, and a direct contact PEDOT counter electrode, a PCE of 13.1% could be obtained under full sun conditions. A 32% PCE at 1000 lux was reported which exceeds the values reported to date with commonly used materials such as silicon and GaAs systems under low light conditions.^320^ Very recently Zhang *et al.* have introduced a new dye – MS5 – with a particularly long *n*-dodecyl “umbrella” alkyl chain and a favorable ground state oxidation potential in respect to the Cu(tmby)_2_ redox couple, leading to a record device *V*_OC_ of 1.24 V for a copper redox shuttle-based device.^12^ The co-sensitization of MS5 with the broader-absorbing XY1b dye resulted in a DSC with a certified PCE of 13.0%, the highest certified efficiency reported to date, while a batch of such devices reached an average 13.5% efficiency when measured in the laboratory. These devices also retained 93% of their initial efficiency after 1000 h of full sun light soaking at 45 °C.

The use of extended π-conjugation systems as donor groups has been an increasing popular strategy for increasing light absorption and improving device PCEs. SC-3 is a perylene-based dye with a bulky diarylamine donor substituted onto a phenanthrocarbazole group (Fig. 21).^275^ A BTD-benzoic acid acceptor was used with SC-3 to give a dye reported to undergo electron injection from non-relaxed, hot excited states. The fast electron injection coupled with good surface protecting gave the dye 11.5% PCE. Notably, replacing the diarylamine group on SC-3 with an arylether group planarized by a ring fusion strategy led to dye C275, with a higher PCE of 12.5% owing to a high voltage (>950 mV) when using the Co(phen)_3_^3+/2+^ RS system.^276^ R6 is designed with a central thienothiophene component fused to two anthracene groups.^277^ A diarylamine donor and a BTD group with a benzoic acid acceptor complete the conjugated system. Two tetra-substituted sp^3^-hybridized carbons provide alkyl groups extending above and below the dye conjugated plane to increase solubility and reduce aggregation. R6-based DSC devices have an IPCE onset near 800 nm and give a 12.6% PCE using a Co(bpy)_3_^3+/2+^-based electrolyte. The devices show a remarkable stability and offer a blue dye for use in aesthetically-driven applications. Dye H2 incorporated a donor group with four alkyl chains with BTD as a π-bridge and benzoic acid as an anchoring group.^278^ This arrangement led to a high photovoltage (900 mV) when paired with a cobalt redox shuttle, indicating minimal recombination losses due to transfer of an electron from the TiO_2_ surface to the oxidized redox shuttle. Exceptional stability was observed from a dye analogue during light soaking studies, but ultimately the DSC device PCE was limited by the absorption range of the dye which had an IPCE onset of ∼750 nm. ZL003 was designed with a novel donor group with three alkylated nitrogens, a bisthiophene-substituted benzothiadiazole (BTD), and a benzoic acid anchoring group. This design resulted in exceptional surface protection with minimal recombination losses for a photovoltage loss of only 106 mV based on the theoretical obtainable photovoltage assuming no shift in the TiO_2_ conduction band taken as −0.5 V *versus* NHE.^279^ Notably, ZL003 was found to up-shift the Fermi level of TiO_2_ by approximately 600–700 mV, which likely contributed to the high photovoltage observed (956 mV) from the ZL003 device with the Co(bpy)_3_^3+/2+^ RS. The exceptional surface protection, rapid hot electron injection occurring out of locally excited states from the dye to TiO_2_, and the broad IPCE onset nearing 800 nm led to the highest performing single-dye DSC device reported in the literature at 13.6% PCE.

A large number of anchoring group strategies have been reported in the literature, with strategies often focused on finding strong binding groups which retain facile electron transfer from the photoexcited dye to TiO_2_. The use of carboxylic acid-based systems is the most popular strategy in the literature owing to their relative ease of preparation and exceptional performance with respect to electron injection. One of the most intriguing motivations for replacing carboxylic acid anchoring groups in DSCs is highlighted with the discovery of ADEKA-1 (Fig. 21).^24,280,321^ ADEKA-1 features a siloxane-based anchoring group as a tight binding group to TiO_2_. The siloxane anchoring group enabled the use of a co-sensitizer (LEG4, which is similar to Y123 with OC_4_H_9_ rather than OC_6_H_13_ alkyl chains on the amine donor, Fig. 22) and a tremendous number of surfaces protecting groups of varied shapes and sizes. This type of extensive co-sensitization is challenging unless a significant difference in anchor binding group strength is present. This strategy has led to the highest performing single DSC device reported in the literature at 14.3% PCE. It is noteworthy that since this discovery, siloxane anchoring groups remain underexplored with respect to incorporation into dye designs which may be due to challenges with identifying the composition of the anchoring group after purification.^329^

##### 4.2.2.1 Wide optical gap organic sensitizers

**Fig. 23:**
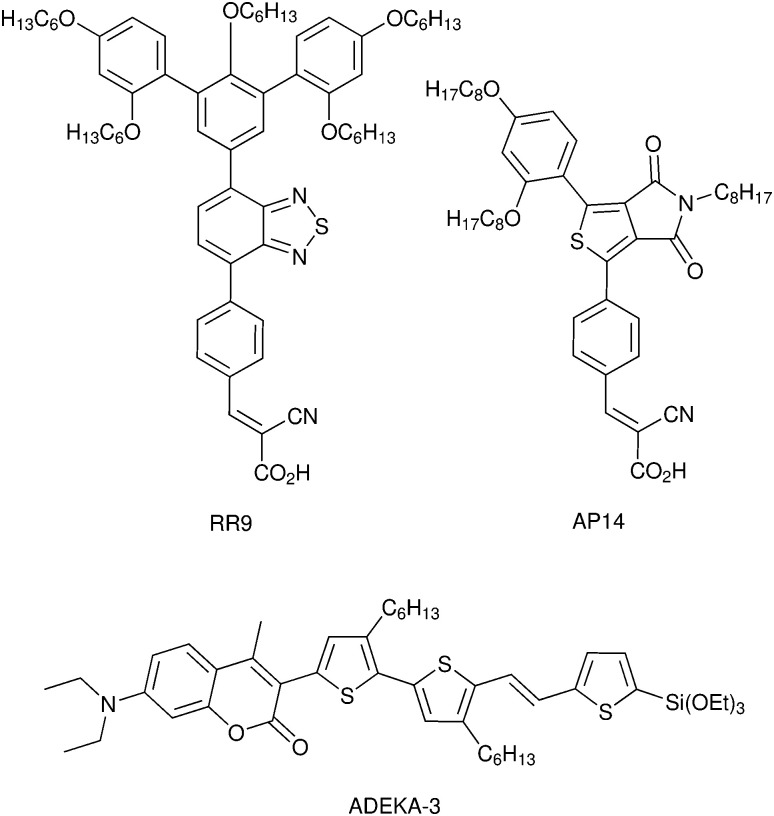
Examples of high voltage dye-designs.

A growing body of work is focusing on the design of wide optical gap dyes which have applications in multijunction or tandem DSC devices as the initial photoactive layer and in photoelectrochemical cell systems. For SSM or tandem systems, the photovoltage output from the wide optical gap dye-based DSC is a critical parameter since higher *V*_OC_ values allow for less free energy waste from high energy visible light (blue) photons. A common objective is to position the dye excited-state energy level near the CB energy of an n-type semiconductor to minimize free energy loss and to position the ground state oxidation potential of the dye positive enough to drive challenging electron transfer reactions. Initial high photovoltage DSCs focused on the use of the Br^−^/Br_3_^−^ RS system with wide optical gap dyes. Through the use of Mg-doped TiO_2_, to shift the CB to a more negative potential, and the Br^−^/Br_3_^−^ RS, a theoretical photovoltage of 1.5 V can be obtained.^216^ A wide optical gap dye with a siloxane-based anchor and a coumarin weak donor (ADEKA-3) was used to give a 1.45 V device at room temperature with 1.5 V observed at 5 °C. A PCE of 3.9% was observed for the room temperature DSC device (Fig. 23). AP14 is designed with an electron deficient thienopyrroledione bridging a benzene with an ether donor and a benzene with a CAA acceptor.^281^ A 1.73 V *versus* NHE oxidation potential was measured for AP14 which is positive enough to drive the oxidation of Fe(bpy)_3_^2+^ in DSC devices to give a 1.32 V device. RR9 is comprised of a BTD π-bridge and a pentaalkylated aryl ether-based weak donor group.^282^ While the ground state oxidation potential of RR9 is less positive (1.56 V *versus* NHE) than that reported for AP14, the DSC devices exhibited a higher *V*_OC_ value of 1.42 V, which was the record high voltage for a room temperature DSC device without the use of TiO_2_ doping at the time of the report. This device was used in a three active layer SSM DSC device (6-terminal, series wired) as the top layer to give a 3.3 V device where the photovoltage output is >1 V per layer. These systems are inherently limited due to the light absorption of Fe(bpy)_3_^3+/2+^; however, they provide proof of principle examples of the value of the dye design strategy and indicate the importance of finding a redox shuttle at ≥1.4 V oxidation potential *versus* NHE that does not absorb visible light for use in SSM or tandem device systems.

##### 4.2.2.2 Porphyrins

**Fig. 24:**
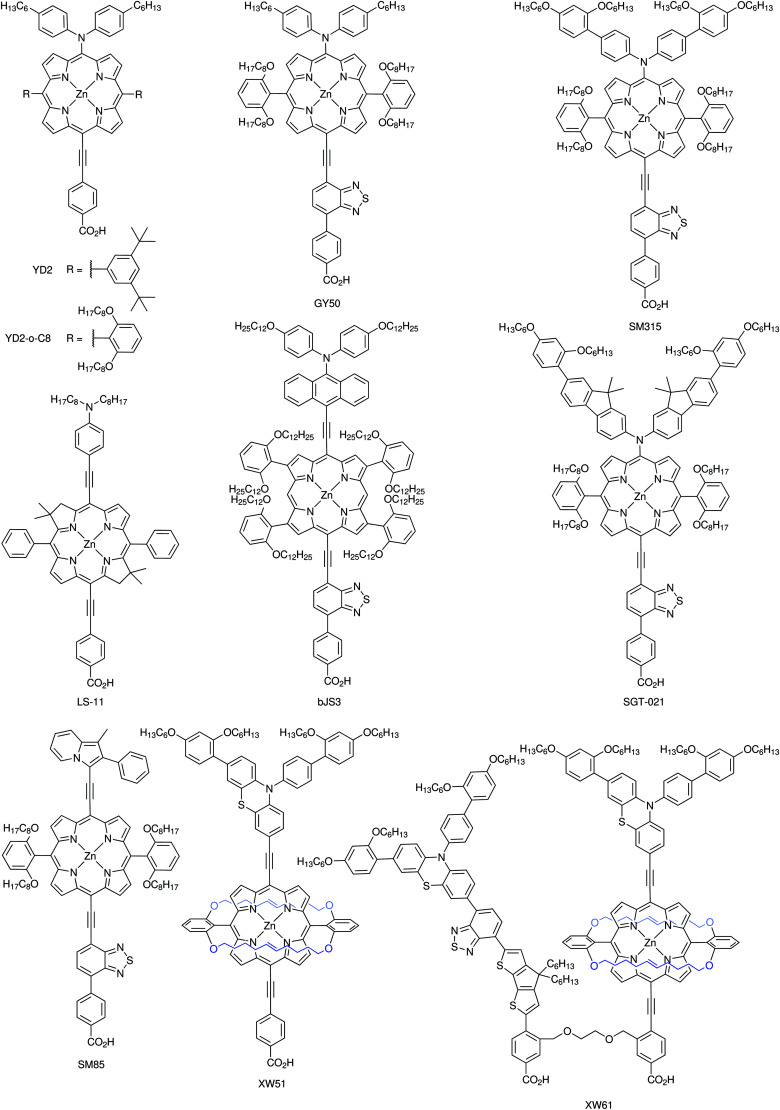
Select porphyrin examples discussed in this review.

Porphyrins are a primary focus of dye design research due in part to porphyrins being one of the first classes of dyes to show comparable and higher PCEs in DSC devices relative to ruthenium complexes. The donor-porphyrin-acceptor construct is one of the most successful design strategies. In 2010, zinc porphyrin dye YD2 demonstrated an impressive 11% PCE without employing any precious metal, and using a diarylamine donor and benzoic acid acceptor at opposite *meso* positions of the porphyrin core (Fig. 24).^283^ Substitution of the remaining two *meso* positions with de-aggregating *tert*-butyl-substituted aryls is a key part of this design, although dyes are known with these two *meso* position being differentiated with high performances.^330^ YD2-o-C8 is a derivative of YD2 with bis-*ortho*-substituted alkyl ether substituents on a benzene ring to better disrupt aggregation of the porphyrin dye.^284^ A complementary organic photosensitizer (Y123, Fig. 22) was used as a co-sensitizer to increase the performance of the YD2-o-C8 device in the 500–650 nm region where porphyrins are relatively weakly absorbing. This co-sensitization gave the highest performing DSC device at the time with a PCE of 12.3%. The landmark PCE was made possible by the use of a 1-electron-based cobalt RS which gave a *V*_OC_ of nearly 1 V. The introduction of a BTD group near the benzoic acid anchor led to GY50, which better absorbs photons in the 500–650 nm range and eliminated the need for the use of a co-sensitizer.^285^ A 12.8% PCE was obtained from a single dye DSC device with a *J*_SC_ of 18.5 mA cm^−2^ using a cobalt-based electrolyte. This high *J*_SC_ value was made possible by both red-shifting the Q-band when introducing the BTD group and increasing the absorptivity of the dye throughout the visible spectral region. Comparatively, GY50 with an iodine-based electrolyte system gave a PCE of only 8.9%, which highlights the critical importance of 1-electron-based RSs with regard to high power conversion efficiencies in DSCs. The diarylamine donor group of GY50 was expanded to include an additional aryl group with four total donor-group alkyl chains on SM315 for better TiO_2_ surface insulation, aimed to slow the recombination of electrons at the TiO_2_ surface with the cobalt-based electrolyte. This strategy led to a ∼25 mV increase in *V*_OC_ for SM315 relative to GY50, resulting in the first DSC device reported to reach 13.0% PCE.^286^ A benzene group on the donor of SM315 was replaced with a fluorene group to give SGT-021.^287,296^ When benchmarked against SM315, a higher photovoltage (20 mV increase) and photocurrent (1.1 mA cm^−2^ increase) were obtained. When a non-porphyrin-based organic sensitizer was used as a top cell in a mechanically stacked tandem device, an impressive 14.6% PCE could be obtained.^288^ Through the incorporation of a D–π–A dye with an exceptionally effective amine donor design to promote favorable charge separation durations, a co-sensitized device with SGT-021 and SGT-149 gave a high PCE of 14.2%.^25^

To improve further on the exceptional efficiencies described above, the use of lower energy photons (>750 nm) is needed. Numerous strategies have emerged with respect to porphyrin dye design aiming to reduce aggregation through novel constructs, improve spectral response both in the visible and NIR *via* building block incorporation, co-link of chromophores, and design of supramolecular assembly strategies (tailored aggregation) as referenced and discussed below. With respect to the linear donor-porphyrin-acceptor design with *meso*-substituted de-aggregating groups, common general methods for extending the absorption range focus on adding donor groups,^289–295^ fusing non-amine donor groups for π-extended donor groups,^331^ or adding acceptor groups^296–301^ as the D and A component to promote lower energy ICT events within the D–porphyrin–A structure. The use of a π-extended donor group has shown promise for improving DSC device performances as well. Specifically, the introduction of an anthracene group between the amine donor and porphyrin (mJS3) resulted in a red shift of both the Soret and Q-band relative to no added anthracene group.^302,303^ However, the PCE of mJS3 dropped significantly compared to a benchmark YD2-o-C8 DSC cell under identical conditions (2.3 % *versus* 9.8 %) primarily due to loss of photocurrent with possible aggregation-limited performance for mJS3. De-aggregating groups at the β positions of the porphyrin were explored in the same study and termed a “double fence” porphyrin due to the use of two de-aggregating aryl groups on each side of the porphyrin (see dye bJS3). The double fence strategy shows minimal changes to the dye energetics in solution, and led to a 10.4% PCE cell, which was higher performing than YD2-o-C8 under identical conditions. The massive improvement from 2.3% to 10.4% based on the shift from *meso* to β-substituted de-aggregative aryls certainly warrants more investigation in this direction. An alternative strategy for red-shifting the porphyrin absorption spectrum has recently been presented which focuses on purposefully inducing aggregation of porphyrin-based dyes with a planarized indolizine donor to allow for an aggregate-induced red-shifting of the absorption spectrum.^304^ This approach allowed for the shifting of the absorption spectrum substantially on TiO_2_*versus* solution (710 nm onset in solution, 875 nm onset on TiO_2_) and provided an under-explored method of absorbing deeper into the NIR spectral region post-synthesis.

Bacteriochlorins are a class of materials related to porphyrins and are known as a type of hydroporphyrin. These building blocks have been used in DSC dye LS-11 with exceptional NIR photon use until 870 nm in DSC devices.^305^ LS-11 shows a relatively intense Q-band (112 000 M^−1^ cm^−1^) compared to many porphyrin-based dyes and multiple absorption features throughout the visible spectral region. However, due to a peak IPCE response of ∼60% and a modest open circuit voltage (0.52 V), the PCE was limited to 5.4%. Further exploration of this class of materials is intriguing given the rare use of NIR photons beyond 800 nm.

Doubly-strapped porphyrins have also shown promise in DSC devices by minimizing aggregate formation thorough the introduction of carbon chains bridging the *meso* positions such as with dye XW51.^306,307^ This strategy leads to a high PCE of 11.1% with the I^−^/I_3_^−^ RS system. XW51 has demonstrated exceptional stabilities over the course of 1000 hours of ageing.^306^ XW51 was covalently linked to a “companion” D–A′–π–A organic dye with a complementary absorption spectrum for a 12.4% PCE from an I^−^/I_3_^−^ RS-based cell generating 21.4 mA cm^−2^ of photocurrent with a remarkable photostability to light soaking.^308^ Significantly diminished performances were reported with a cobalt electrolyte (10.7% PCE), likely due to recombination of electrons in TiO_2_ with the oxidizing electrolyte. Strategies aimed at complete aggregation mitigation and shifting the absorption spectrum onset of porphyrins to lower energy remain intriguing directions for this class of materials.

##### 4.2.2.3 Squaraines

**Fig. 25:**
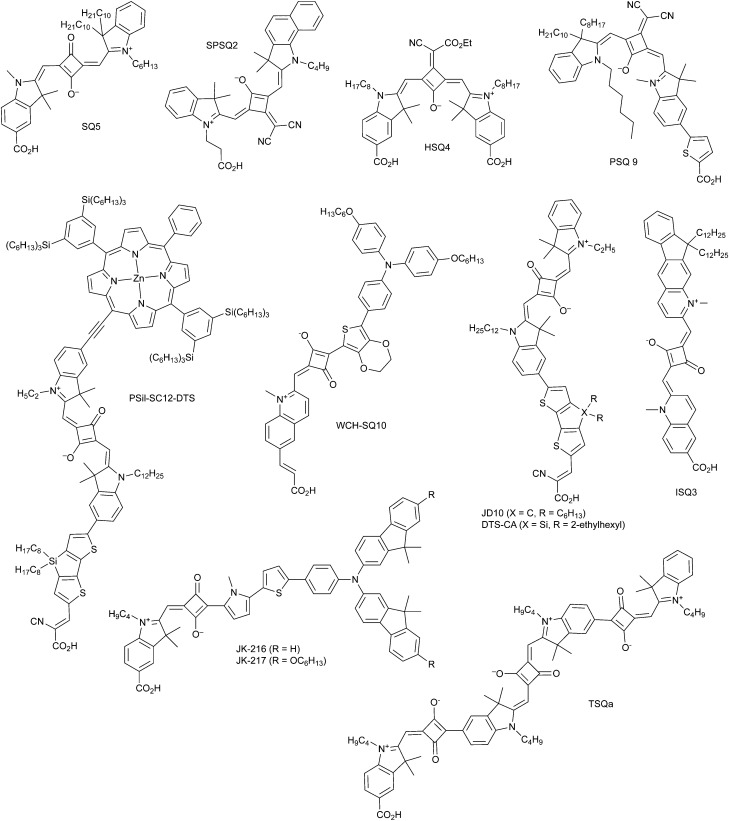
Examples of squaraine-based dyes.

Squaraine dyes are a popular class of materials in dye-sensitized systems owing to their strong absorption into the NIR spectral region. Squaraine-based dyes have shown some of the deepest NIR photon use in DSC devices known.^309^ Squaraines typically absorb intensely in the NIR region often between 600–900 nm with molar absorptivities often above 100 000 M^−1^ cm^−1^; however, absorption is typically weak in the higher energy spectral region. The literature surrounding this class of materials is expanding dramatically since high performing NIR absorbing chromophores are urgently needed to improve DSC devices. Select examples of squaraine dyes are discussed below (Fig. 25).

A series of squaraines with systematically varied alkyl groups in- and out-of the π-system plane were evaluated with alkyl group positions both near and far from the TiO_2_ surface.^310^ Extending the out-of-plane alkyl groups on the indoline building block furthest from the surface was found to have a dramatic effect on overall DSC device performance. Under identical conditions, the PCE increased from 3.4% with methyl groups in place of long alkyl chains to 7.7% PCE for SQ5 (Fig. 25). Including alkyl chains at the indoline near the TiO_2_ anchor led to a decrease in PCE to 6.8% which was attributed to lower dye loading. Under fully optimized conditions with reduced chenodeoxycholic acid loadings, SQ5 reached a PCE of 8.9%. These findings are notably recent, and many of the examples discussed below use much shorter alkyl chains on the indoline portion of the dye far from the TiO_2_ surface. Addition of π-conjugated groups extending from the squaraine chromophore have been used to increase the absorption of dyes in the high energy region and to red-shift the strong NIR absorption further. A series of eight π-bridges were examined with the indoline-based squaraine core showing 4,4-dihexyl-4*H*-cyclopenta[2,1-*b*:3,4-*b*′]dithiophene (CPDT) as the highest efficiency π-bridge studied as part of dye JD10.^311^ Part of the high performance is attributed to the alkyl chains on CPDT out of the π-system plane leading to reduced aggregation and the introduction of a high energy absorption band upon incorporation of CPDT. Squaraine dyes in general benefit from co-sensitization with visible light-absorbing dyes and when JD10 was co-sensitized with D35 the efficiency could be improved to 7.9% PCE from 7.3% PCE without D35. Upon replacing the alkylated carbon of CPDT with an alkylated silicon atom to give a 4,4-bis(2-ethylhexyl)-4*H*-silolo[3,2-*b*:4,5-*b*′]dithiophene (DTS) group for dye DTS-CA, the PCE improved to 8.9%.^312^ DTS-CA was found to have low recombination rates and reduced aggregation, which contributed to the observed high performance. The high energy bands introduced by the CPDT and DTS groups in the 400–550 nm region were modest in intensity but had a strong effect on the IPCE curve in this region. To balance the dye's absorption intensity of the low- and high-energy photons, a porphyrin ring was added to the DTS-CA structure to give PSil-SC12-DTS, which absorbs strongly from 400–550 nm due to the porphyrin core.^313^ However, despite the balancing of the absorption bands, the peak percent IPCE of the devices with PSil-SC12-DTS dropped from ∼90% with DTS-CA to ∼70%, which was attributed to a lower charge injection efficiency.

DSCs are thought to reach a theoretical maximum practical PCE from a single active layer device near 950 nm.^236^ Very few dye designs have reached this value. The NIR absorption of squaraine chromophores places them relatively near to this value with IPCE onsets routinely near 800 nm. One approach aimed at a further red-shifting of the squaraine chromophore is based on the use of multiple squaraine building blocks on a single dye such as with TSQa.^314^ The common bis-indoline-squaraine chromophore has a solution absorption onset of approximately 700 nm. Through the introduction of multiple squaraine building blocks onto the bis-indoline-squaraine chromophore, a solution absorption onset >900 nm could be reached. An IPCE onset of near 1000 nm was obtained with TSQa; however, the peak IPCE was limited to <20%. The addition of multiple squaraine building blocks was found to dramatically lower the dye LUMO energy resulting in a low driving force for electron transfer to TiO_2_. A second approach to red-shifting squaraine-derived dyes focuses on the de-symmetrization of the commonly used bis-indoline chromophore to allow for the use of a donor–π–bridge group (triarylamine-thiophene-pyrrole based) with a single indoline-squaraine building block as with dyes JK-216 and JK-217.^315^ An IPCE onset of near 850 nm was obtained with the more red-shifted JK-217. The higher *V*_OC_ (610 mV) and FF (74%) with JK-216 led to a higher PCE of 6.3% than is observed with JK-217 (*V*_OC_ = 583 mV, FF = 70%, PCE = 5.5%). Importantly, both dyes were shown to be stable to prolonged light soaking (1000 h at 60 °C) and function well in solid-state devices. WCH-SQ10 is comprised of a triarylamine-3,4-ethylenedioxythiophene donor–π–bridge with a squaraine-quinoline-based structure.^316^ This design lead to an IPCE onset beyond 1000 nm to give one of the deepest NIR photon accessing organic dyes known. Interestingly, a symmetric core bis-quinoline squaraine dye (ISQ3) shows appreciable light harvesting efficiency on TiO_2_ reaching 1000 nm, but an IPCE onset near 850 nm.^309^ This suggests significant influence of the electrolyte on the dye absorbance energy with quinoline-squaraine based materials.

Dicyanomethylene-based squaraine materials show significant red shifts of the absorption spectrum onset relative to the keto squaraine core. Dye PSQ9 has a broad IPCE spectrum reaching ∼850 nm and generating >17 mA cm^−2^ of photocurrent. Due to a modest photovoltage (577 mV) – as is common in the NIR region with dye sensitized solar cells – the overall power conversion efficiency was limited to 6.9% PCE.^317^ An ethyl cyanoacetate-derived squaraine dye (HSQ4) with dual anchors was shown to have a substantially increased stability relative to mono-anchored squaraine dyes with no change in PCE after 1000 hours.^318^ In this same study, the ethyl cyanoacetate group was found to give a dye with a significantly higher excited state oxidation potential than a dicyanomethylene derived dye, which correlated to a higher IPCE peak value (80% *versus* 70%). Dicyanomethylene squaraines without a conjugated anchoring group have also been shown to function well within co-sensitized DSC devices.^319^ SPSQ2 was found to increase the performance of N3-based devices by red-shifting the IPCE onset leading to an improved *J*_SC_ (14.9 mA cm^−2^ without SPSQ2 and 17.1 mA cm^−2^ with SPSQ2) and improved PCE (7.1% *versus* 8.2%).

With substantial recent progress having been shown in co-sensitized DSC devices and in deep NIR photon absorption, continued vigorous research within the area of squaraine dyes is likely and warranted. Notably, the majority of squaraine dye-based DSC devices in the literature rely on the 2-electron I^−^/I_3_^−^ RS system, which inherently limits the PCEs of DSC devices. Progressive improvements have been observed with squaraine dyes reaching ∼9% PCE to date with the I^−^/I_3_^−^ RS. Similar to the breakthrough performances enabled with porphyrin-based sensitizers, a squaraine dye design that functions well with 1-electron RSs such as Co- and Cu-based systems is needed. This advance in porphyrin designs shifted the PCE from ∼9% to ∼13% when Co RS-compatible dyes were discovered. A similar discovery would greatly benefit squaraine research.

##### 4.2.2.4 Multifunctional DSCs

**Fig. 26:**
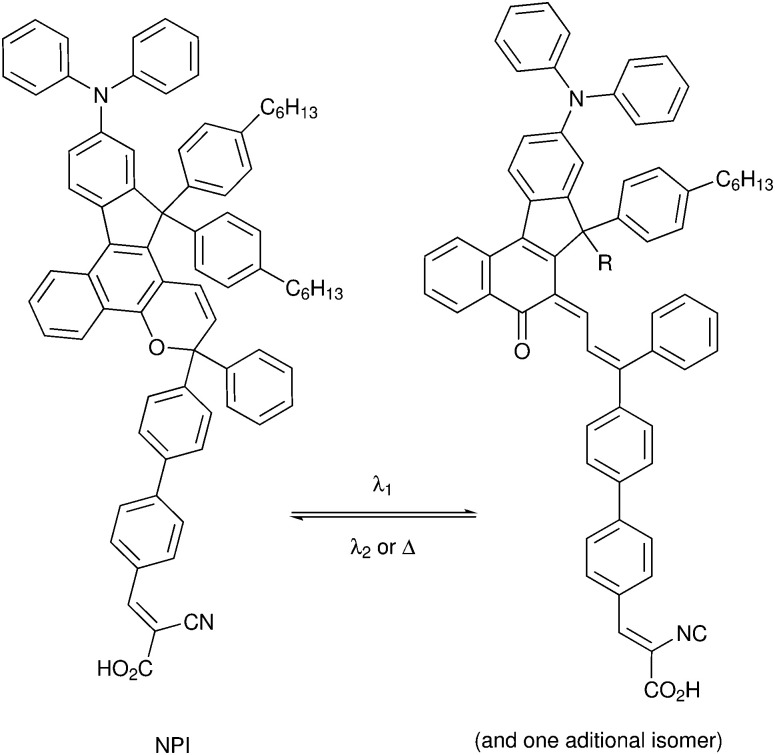
Photoresponsive NPI in a non-visible light absorbing state (left) and a visible light absorbing state (right).

DSCs have shown exceptional performances as described above in terms of low light intensity use and in tandem of SSM device designs. Additionally, DSCs are intriguing materials for aesthetically important devices owing to the wider range of colors available from the dyes used in these devices. Given the molecular nature of the chromophores being used, photochromic dyes offer a possible strategy for accessing materials with dynamic optical properties and electricity production. DSCs have been shown to operate as photo-chromo-voltaic cells that can be converted from transparent states to visible light absorbing states with the NPI dye (Fig. 26). The use of photochromic dyes is intriguing for building-integrated photovoltaics which can exist in semi-transparent states at night and as visible light absorbing states in the daytime. A key challenge with this approach consists in synthesizing dyes with reasonable power conversion efficiencies in the visible light absorbing state since visible light is competitively used within the devices to both drive electron transfers to the metal oxide semiconductor, and to convert the dye back to the non-visible light absorbing state. The use of diphenyl-naphthopyran has shown exceptional promise in allowing for a PCE >4% with good device stability (50 days tested).^199^ Interestingly, the diphenyl-naphthopyran building block also allows for thermal conversion or light intensity-based conversion back to a transparent state giving a self-adjusting transmission. Continued research in this area is promising with regard to building integrated photovoltaic markets.

### 4.3 Charge transport materials

Although they had been neglected in the early stages of DSC development, charge transport materials (CTMs) are an essential part of this technology and therefore some of the most significant advances in the field of the past decade were made through progress on this component.^23,332–338^ Research on CTMs branched into the development of materials, the study of their properties and the fundamental understanding of charge transport within the materials and devices. CTMs are responsible for electron transfer between the electrodes and they must be able to regenerate the oxidized dye following light absorption and to be reduced at the counter electrode. Charge transport materials are not only essential for the solar cell efficiency, but they also determine its overall stability. All parameters defining the efficiency of solar cells including the short-circuit photocurrent density (*J*_SC_), open-circuit photovoltage (*V*_OC_) and the fill factor (FF) are influenced by the properties of charge transport materials and their interface interaction with the electrodes.^15,336,339–341^ The photocurrent density, even if largely determined by the photon-to-electron conversion abilities of dyes,^342,343^ is still influenced by the charge transport abilities and recombination pathways of the CTM.^344^ The *V*_OC_ depends on the energy alignment between the Fermi level of the TiO_2_, the ground state of the dye and the overpotential to the CTM.

CTMs can be integrated in DSCs in the liquid, quasi-solid and solid state.^10^ Liquid CTMs or electrolytes in solar cells comprise an organic, aqueous or ionic solvent with a redox couple, for example I^−^/I_3_^−^,^345–347^ copper^14,95,96,346,348–353^ or cobalt^270,284,286,337,354–356^ coordination complexes or organic molecules.^357^ For DSCs to become commercially viable, significant efforts are being made to develop quasi-solid- and solid-state charge transport materials to ensure sustainability and stability. These CTMs are usually based on organic molecules and polymers^333,358,359^ or on inorganic and coordination metal complexes. The fundamental differences between the various charge transport materials are the charge mobility and mechanism.^10^ While in liquid electrolytes there is a prevalence of ionic conductivity, in polymeric and solid-state CTMs the mechanism can be a combination of ionic and electronic transport, or a predominantly electronic process.^360^

#### 4.3.1 Liquid electrolytes and redox mediators

Liquid electrolytes are an important component of all electrochemical devices, including capacitors, fuel cells, and batteries (*e.g.* lithium-ion batteries), in addition to DSCs. Redox couples and additives are usually dissolved in a liquid solvent. By using dopants/additives, several photovoltaic characteristics of DSCs can be optimized: the redox couple potential, the semiconductor surface state, the semiconductor conduction band edge, recombination kinetics, and photovoltaic parameters.

In order to transport charges between the electrodes efficiently, charge transport materials in DSCs must fulfill several requirements:^361–363^ (i) a redox potential that provides the minimal overpotential, but with a driving force high enough to efficiently regenerate the dye, (ii) low recombination rates with the metal oxide semiconductor and the conductive substrate, (iii) minimal mass transport limitations for fast diffusion through the mesoporous semiconductor towards the counter electrode, (iv) absence or minimization of unwanted chemical and physical interactions with other components of the solar cell to improve overall stability, (v) no or minimal competitive light absorption with respect to the dye.

Currently, there is no ideal electrolyte system that fulfills all requirements, but there are several successful systems that have been discovered, and their advantages and drawbacks will be outlined. Of all the requirements above, the most important characteristics of a redox couple for highly efficient DSCs are fast dye regeneration and slow charge recombination.^10^Table 4 lists device parameters of DSCs employing various liquid electrolytes referenced in this review, together with the dye used.

**Table 4:** Photovoltaic characteristics of DSCs employing various redox mediator couples

Mediator	Sensitizer	*V*_OC_ (mV)	*J*_SC_ (mA cm^−2^)	FF (%)	PCE (%)	Year	Ref.
I^−^/I_3_^−^	N719	846	17.73	75	11.18	2005	145
Br^−^/Br_3_^−^	ADEKA-3	1450	4.77	56	3.9	2016	216
I^−^/IBr_2_^−^	N3	790	12.8	64	6.4	2007	364
I^−^/I_2_Br^−^	N3	640	9.2	41	2.4	2007	364
Co(bpy)_3_	D35	936	12.05	69.1	7.80	2018	95
Co(bpy)_3_	D45	810	13.40	73.0	7.93	2018	95
Co(bpy)_3_	D5	713	9.45	72.8	4.91	2018	95
Co(bpy)_3_	N719	620	3.8	76	1.8	2011	365
Co(bpy)_3_	Z907	744	14.0	62	6.5	2011	365
Co(bpy)_3_	D9L6	688	10.7	72	5.32	2012	165
Co(bpy)_3_	D21L6	852	12.3	63	6.63	2012	165
Co(bpy)_3_	D25L6	854	10.8	63	5.51	2012	165
Co(bpy)_3_	Y123	855	14.6	70	8.8	2011	271
Co(bpy)_3_	YD2	825	14.9	69	8.4	2011	284
Co(bpy)_3_	YD2-o-C8	965	17.3	71	11.9	2011	284
Co(bpy)_3_	SM371	960	15.9	79	12.0	2014	286
Co(bpy)_3_	SM315	910	18.1	78	13.0	2014	286
Co(bpy)_3_	MK2	826	13.7	69	7.8	2013	366
Co(bpy)_3_	LEG1	815	8.80	60	4.3	2013	367
Co(bpy)_3_	LEG2	830	11.2	51	4.7	2013	367
Co(bpy)_3_	LEG3	915	8.9	68	5.5	2013	367
Co(bpy)_3_	LEG4	805	12.1	68	6.6	2016	355
Co(bpy)_3_	C218/MKA253	810	12.2	69	6.9	2016	355
Co(phen)_3_	D35	910	7.3	62	4.2	2015	368
Co(phen)_3_	ADEKA-1/LEG4	1014	18.27	77.1	14.3	2015	24
Co(phen)_3_	Z907	700	3.6	56	1.4	2015	368
Co(Me_2_bpy-pz)_2_	D35	1020	6.1	61	3.7	2013	166
Co(bpy-pz)_2_	D35	1020	5.3	68	3.6	2013	166
Co(py-pz)_3_	D35	900	2.5	66	1.5	2013	166
Co(Mepy-pz)_3_	D35	880	0.78	58	0.4	2013	166
SBCC	D35	905	5.19	53.8	2.53	2014	369
Co(phen)_3_/Co(EtPy)_2_	Z907	750	5.1	58	2.2	2015	368
Co(phen)_3_/Co(EtPy)_2_	D35	920	8.4	67	5.1	2015	368
Co(PY5Me_2_)(*t*BP)	MK2	993	8.1	76	6.1	2012	337
Co(PY5Me_2_)(NMBI)	MK2	940	11.8	77	8.4	2012	337
Co(bpyPY4)	MK2	757	14.7	75	8.3	2013	366
Co(ttb)	LEG4	810	11.6	57	5.4	2016	355
Co(ttb)	C218/MKA253	805	13.0	60	6.6	2016	355
Cu(SP)(mnt)	N719	660	4.4	44	1.3	2005	370
Cu(dmp)_2_	N719	790	3.2	55	1.4	2005	370
Cu(dmp)_2_	C218	932	11.29	66	7.0	2011	346
Cu(dmp)_2_	LEG4	1020	12.6	62	8.3	2016	96
Cu(dmp)_2_	Y123	1060	13.61	69.2	10.3	2016	14
Cu(dmp)_2_	D5	1130	9.02	73.6	7.53	2018	95
Cu(dmp)_2_	D45	1020	9.90	74.1	7.48	2018	95
Cu(dmp)_2_	D35	1140	11.40	70.6	9.22	2018	95
Cu(dmp)_2_	G3	860	3.8	59	1.9	2016	351
Cu(dmp)_2_	D	750	4.7	36	1.3	2018	371
Cu(phen)_2_	N719	570	0.48	43	0.12	2005	370
Cu(bpye)_2_	LEG4	904	13.8	71.8	9.0	2016	372
Cu(bpye)_2_	Y123	627	13.2	65	5.6	2020	352
Cu(dmby)_2_	Y123	1070	14.15	68.7	10.0	2016	14
Cu(dmby)_2_	D5	1070	9.85	71.2	7.53	2018	95
Cu(dmby)_2_	D45	956	11.85	68.0	7.71	2018	95
Cu(dmby)_2_	D35	1130	11.53	60.2	7.84	2018	95
Cu(tmby)_2_	Y123	1040	15.53	64.0	10.3	2016	14
Cu(tmby)_2_	D5	837	10.79	67.4	6.10	2018	95
Cu(tmby)_2_	D45	984	12.52	67.3	8.30	2018	95
Cu(tmby)_2_	D35	1110	12.81	66.1	9.44	2018	95
Cu(tmby)_2_	L348	1170	6.4	72.0	5.3	2018	274
Cu(tmby)_2_	L349	1160	11.0	71.7	9.2	2018	274
Cu(tmby)_2_	L350	1140	13.0	76.0	11.2	2018	274
Cu(tmby)_2_	L351	1060	11.2	76.3	9.1	2018	274
Cu(tmby)_2_	WS-70	1060	13.2	77	11.0	2018	273
Cu(tmby)_2_	WS-72	1100	13.3	78	11.6	2018	273
Cu(tmby)_2_	D35/XY1	1030	16.19	68	11.3	2017	348
Cu(tmby)_2_	Y123/XY1b	1050	13.1	79	13.1	2018	320
Cu(tmby)_2_	XY1	1000	13.3	67	8.9	2020	26
Cu(tmby)_2_	L1	910	9.4	71	6.1	2020	26
Cu(tmby)_2_	XY1/L1	1080	15.9	67	11.5	2020	26
Cu(eto)_2_	D5	828	10.12	71.5	6.00	2018	95
Cu(eto)_2_	D45	978	12.59	66.7	8.21	2018	95
Cu(eto)_2_	D35	1120	11.93	66.3	8.84	2018	95
Cu(2-mesityl-4,7-dimethyl-1,10-phenanthroline)_2_	G3	720	9.3	66	4.4	2016	351
Cu(2-*n*-butyl-1,10-phenanthroline)_2_	D	610	6.3	53	2.0	2018	371
Cu(2-*n*-butyl-1,10-phenanthroline)_2_	G3	860	10.1	66	5.7	2018	373
Cu(2-*n*-butyl-1,10-phenanthroline)_2_	G4	780	10.1	63	4.9	2018	373
Cu(2-mesityl-1,10-phenanthroline)_2_	G3	830	11.4	59	5.6	2018	373
Cu(2-mesityl-1,10-phenanthroline)_2_	G4	840	11.7	54	5.3	2018	373
Cu(2-tolyl-1,10-phenanthroline)_2_	G3	870	11.1	62	6.0	2018	373
Cu(2-tolyl-1,10-phenanthroline)_2_	G4	870	11.1	62	6.0	2018	373
Cu(2-phenyl-1,10-phenanthroline)_2_	G3	880	8.0	69	4.9	2018	373
Cu(2-phenyl-1,10-phenanthroline)_2_	G4	810	10.2	58	4.8	2018	373
Cu(oxabpy)	Y123	920	9.75	69	6.2	2018	353
Cu(1)	Y123	689	5.7	77	3.1	2020	352
Cu(2)	Y123	693	10.2	72	4.7	2020	352
Cu(3)	Y123	792	7.9	75	4.3	2020	352
K_4_Ni[Fe(CN)_6_]	N3	790	8	70	4	2011	375
Fe(bpy)_3_	RR9	1420	2.8	47	1.9	2018	282
Ferrocene	Carbz-PAHTDTT	842	12.2	73	7.5	2011	374
Me_10_Fc	Carbz-PAHTDTT	437	6.6	40	1.1	2012	376
Et_2_Fc	Carbz-PAHTDTT	641	13.3	50	4.2	2012	376
EtFc	Carbz-PAHTDTT	669	12.8	56	4.8	2012	376
BrFc	Carbz-PAHTDTT	671	9.3	48	3.0	2012	376
Br_2_Fc	Carbz-PAHTDTT	599	4.4	46	1.2	2012	376
Mn(acac)_3_	K4	765	7.8	73	3.9	2014	377
Mn(acac)_3_	MK2	733	8.6	69	4.4	2014	377
Mn(acac)_3_	N719	771	7.9	73	4.4	2014	377
Mn(CF_2_)_3_	MK2	800	4.95	69	2.72	2016	378
VO(salen)	D205/D131	740	12.3	59	5.4	2013	379
VO(hybeb)	N719	660	5.2	58	2	2015	380
T^−^/T_2_	Z907	687	15.9	72	7.9	2012	357
T^−^/T_2_	N719	630	14.25	68	6.10	2012	381
AT^−^/BAT	N719	670	13.76	68	6.27	2012	381
ET^−^/BET	N719	632	9.3	71	4.2	2013	382
TEMPO	D-149	830	9.4	70	5.4	2008	383
TEMPO	LEG4	965	7.74	73	5.43	2015	356
TEMPO	D205	880	9.88	75	6.5	2012	384
TEMPO	D205/D131	780	13.5	66	7.0	2012	384
AZA	D205	820	12.9	76	8.1	2012	384
AZA	D205/D131	850	13.3	75	8.6	2012	384
TMTU	D205	777	16.6	49	6.32	2013	385
TMTU	D102	770	13.8	54	5.74	2013	385
TMTU	D131	825	11.0	61	5.53	2013	385
TMTU	N719	626	10.3	50	3.22	2013	385
TMTU	Z907	642	8.3	53	2.82	2013	385
HQ/BQ	N719	755	10.28	66.7	5.2	2013	386
HQ/BQ	CM309	755	12.10	67.8	6.2	2013	386
HQ/BQ	Y123	533	6.5	30	1.08	2018	387
PhHQ/PhBQ	Y123	528	6.3	39	1.3	2018	387
DTHQ/DTBQ	Y123	542	12.6	36	2.5	2018	387
ThymHQ/ThymBQ	Y123	455	10	44	2.0	2018	387

##### 4.3.1.1 Halide redox mediators

Initially, successful and efficient DSCs used the iodide/triiodide redox mediator.^87,145,388,389^ The I^−^/I_3_^−^ redox couple shows remarkable performance up to its record PCE of 11.9% (certified, 12.4% non-certified).^308,390^ The I^−^/I_3_^−^ redox couple fulfills several requirements for an ideal electrolyte and it was for several decades the benchmark for research and industry. Advantages of the I^−^/I_3_^−^ redox couple include a suitable redox potential for many dyes, small molecular size for high diffusion, good solubility in a wide range of solvents at high concentration for high conductivity, and good stability. However, it also has several drawbacks, which have initiated the search for alternative redox mediators: (i) substantial light absorption of the triiodide and other possible polyiodide species in the 400–500 nm range of the solar spectrum, (ii) corrosivity towards several components of DSCs including the materials used for counter electrodes and sealing, (iii) possible iodine diffusion out of the electrolyte stemming from its high vapor pressure, and especially (iv) the very large driving force of over 0.5 V for dye regeneration due to the two-electron oxidation steps from I^−^ to I_3_^−^. Consequently, the *V*_OC_ attainable from a DSC containing the iodide/triiodide electrolyte is smaller than what is theoretically possible given the choice of dye. Since the overall efficiency of a solar cell scales directly with its *V*_OC_, this large driving force constitutes a significant limitation of the I^−^/I_3_^−^ redox couple.^388^

The step towards iodide-free redox mediators begins with bromide/tribromide, which has a more positive potential of an additional 0.35 V, a two-electron transfer, and high solubility in many solvents. Thus, the electrolyte containing the bromide/tribromide redox system can lead to an increased photovoltage, but at the cost of lower *J*_SC_ values. Hanaya and co-workers successfully implemented the Br^−^/Br_3_^−^ electrolyte with the organic dye ADEKA-3 and a Mg^2+^-doped anatase TiO_2_ electrode, reaching a photovoltage over 1.4 V and a conversion efficiency close to 4%.^216^ The development bottleneck for the Br^−^/Br_3_^−^-based electrolyte remains the search for a suitable dye. Bi-Interhalogen redox systems, such as I^−^/IBr_2_^−^ and I^−^/I_2_Br^−^ were also tested in combination with ruthenium-based sensitizing dyes and reached conversion efficiencies up to 6.4%.^362,364^

Furthermore, pseudohalogen-based redox couples SCN^−^/(SCN)_2_ and SeCN^−^/(SeCN)_2_ have been studied with the hope to enhance *V*_OC_ in DSCs, because their redox potentials are 0.19 and 0.43 V more positive than that of the I^−^/I_3_^−^ redox couple, respectively. However, since dye regeneration efficiency with these systems is low, it only resulted in low photocurrents. SeCN^−^ has ambivalent reactivity and can interact with the dye from the Se and N side.^391^

##### 4.3.1.2 Transition metal coordination complexes

Cobalt-, iron-, copper-, nickel-, manganese- and vanadium-based complexes as one-electron outer-sphere redox couples are currently the most promising and successful candidates to replace the I^−^/I_3_^−^ system in DSCs.^11^ Their characteristics are suitable for the commercialization of DSCs because they have reversible electrochemical properties, structural tunability, and more positive Fermi level values, reduced visible light absorption and superior stability compared to I^−^/I_3_^−^. Metal complexes' electronic properties and redox chemistry can be readily adjusted by altering the central metal cation or, most importantly, the ligands. Marcus theory states that a driving force of 0.2 eV is adequate for outer-sphere single-electron-transfer processes to guarantee a rapid dye regeneration rate, leading to *V*_OC_ improvements.^95,166^ The development of novel redox mediators has attracted less interest than that of sensitizing dyes or other materials for different DSC components, but recent developments have renewed the attention to this aspect of DSCs.^392^

###### Cobalt coordination complexes

**Fig. 27:**
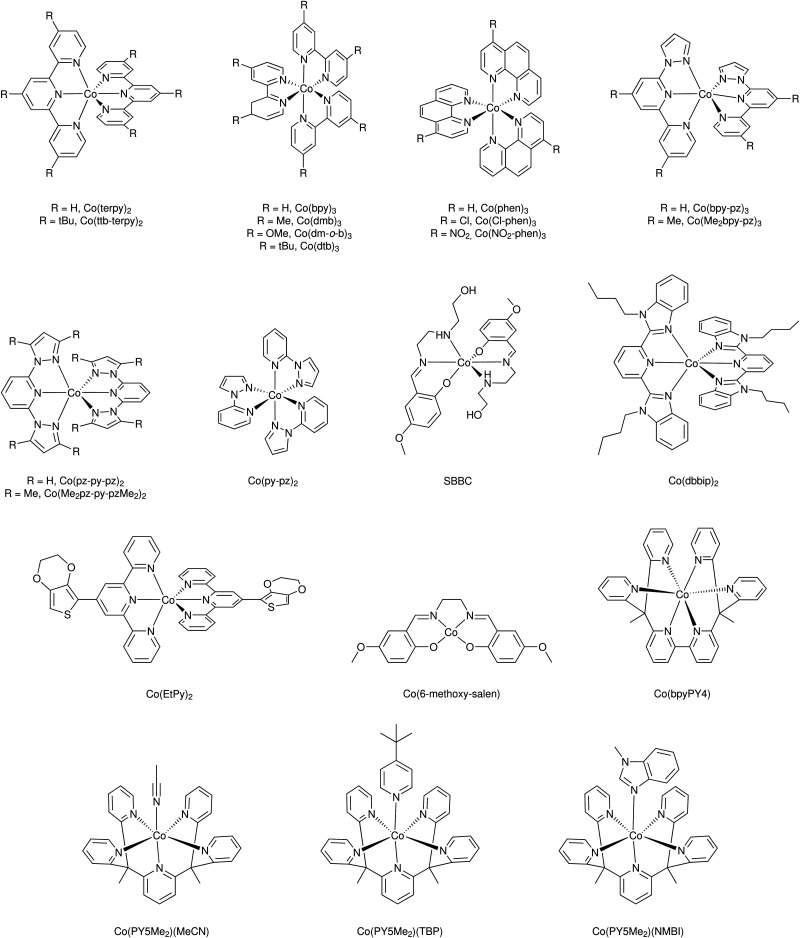
Chemical structures of cobalt coordination complexes-based redox mediators implemented in DSCs.

Tridentate (*e.g.* terpyridines) and bidentate (*e.g.* bipyridines and phenantrolines) ligands often form octahedral coordination complexes in the most common Co-based redox mediators.^166,365,367,393^ In 2010, the Hagfeldt group achieved the first successes in high-efficiency DSCs integrating transition metal complexes by combining a novel Co complex-based electrolyte with the organic dye D35. By introducing a succession of complexes with different ligands, the scientists developed a library of redox mediators with a diversity of redox characteristics.^270^ The initially achieved efficiency of 7% under 1 sun (*V*_OC_ of 0.92 V and *J*_SC_ of 10.7 mA cm^−2^) was reached with the [Co(bpy)_3_]^3+/2+^ redox couple (Fig. 27). In 2012, Mosconi *et al.* were able to show that the formation of an ion pair between the negatively-charged Ru dye and the positively-charged Co complex was responsible for the increase in recombination processes and consequent poor performance of DSCs implementing these systems. This was improved later with addition of larger blocking groups on the Ru dyes.^165^

A follow-up study by Feldt *et al.* on fundamental aspects of the regeneration and recombination processes of cobalt redox mediators also confirmed that a driving force of 0.25 eV was sufficient to ensure 84% dye regeneration.^166,343^ The introduction of this new redox mediator system led to a surge in dye development. In 2011 Tsao *et al.* increased the efficiency with the organic dye Y123, which had a high extinction coefficient thanks to the cyclopentadithiophene (CPDT) π-bridge. DSCs reaching a PCE of 8.8% (*V*_OC_ = 0.855 V, *J*_SC_ = 14.6 mA cm^−2^) under 1 sun were obtained in conjunction with a platinized FTO counter electrode.^271^ A new family of porphyrin-based dyes was introduced by Yella *et al.*, YD2 and YD2-o-C8, leading to an impressive PCE of 11.9% under full sun (*V*_OC_ = 0.965 V, *J*_SC_ = 17.3 mA cm^−2^).^284^

The PCE mark of 13% was passed by Mathew *et al.* with porphyrins improved through a triphenylamine-type hydrophobic donor, leading to dyes SM315 and SM371.^286^ The highest efficiency reported for DSCs to date is still that obtained with the [Co(phen)_3_]^3+/2+^ redox mediator by Kakiage *et al.*, who reached a PCE of 14.3% under full sun (*V*_OC_ = 1.01 V, *J*_SC_ = 18.2 mA cm^−2^) by cosensitizing the ADEKA-1 (MK2 dye variant with an alkoxysilyl binding group) and LEG4 dyes.^24^ A series of 2,2′-ethylenebis(nitrolomethylidene)diphenol-*N*,*N*′-ethylenebis(salicylimine) (salen)-based cobalt complexes was introduced by Nasr-Esfahani *et al.* in 2014 and reached a PCE of only 2.53% under full sun illumination.^369^ New complexes were developed by Koussi-Daoud *et al.* with a cobalt coordination complex Co(EtPy) _2_ featuring a terpyridine functionalized with 3,4-ethylenedioxythiophene (EDOT).^368^ This combination of an electron cascade to the PEDOT counter electrode lead to an enhanced cell efficiency of 5.1% with D35 at 1 sun. The group of U. Bach also introduced new cobalt-based redox mediators with 4-*tert*-butylpyridine (*t*BP) and *N*-methylbenzimidazole (NMBI). The tested complexes [Co(PY5Me2)(*t*BP)]^3+/2+^, [Co(PY5Me2)(NMBI)]^3+/2+^ and [Co(PY5Me2)(MeCN)]^3+/2+^ reached an efficiency of 8.4% under full sun (*V*_OC_ = 0.94 V, *J*_SC_ = 11.8 mA cm^−2^).^337^ They further introduced a hexadendate ligand in 2015 to increase the overall stability of cobalt redox mediators. Devices fabricated with this new Co complex, and MK2 or Y123 as dye produced a PCE up to 8.3% under full sun.^366,394^ In 2016, Freitag *et al.* introduced the new supramolecular, hemicage cobalt-based mediator [Co(ttb)]^3+/2+^ with the highly pre-organized hexadentate ligand 5,5′′,5′′′′-((2,4,6-triethyl benzene-1,3,5-triyl) tris(ethane-2,1-diyl))tri-2,2′-bipyridine (ttb) reaching the same performance as with [Co(bpy)_3_]^3+/2+^ (bpy = 2,2′-bipyridine) redox mediator and the LEG4 dye.^355^ Both hexadendate systems exhibit exceptional stability under thermal and light stress.

The addition of aqueous electrolytes aided in the advancement of stabilization and sustainability, and also required the development and use of appropriate hydrophilic dyes. The combination of MK2 and [Co(bpy)_3_]^3+/2+^ was utilized by Xiang and colleagues in 2013.^395^ They eventually achieved aqueous-based devices with a PCE of 5.0% at 1 sun illumination (*V*_OC_ = 0.687 V, *J*_SC_ = 9.8 mA cm^−2^). Dong *et al.* used the common strategy of introducing surfactants in DSCs and reached a PCE of 5.6% under full sun (*V*_OC_ = 0.821 V, *J*_SC_ = 10.17 mA cm^−2^) with the MK2 dye.^396^ In 2016, Ellis *et al.* introduced two complexes with high solubility in water, [Co(bpy)_3_](NO_3_)_2_ and [Co(phen)_3_]Cl_2_, and the new dye D51, with a shorter blocking group to allow better wetting in comparison to the organic dye D35. The initial performance reported was 1.4% and 3.4%, respectively, both under 1000 W m^−2^ illumination.^397^ In the same study, optimization of [Co(phen)_3_]Cl_3_ concentration allowed further performance enhancements to 4.8% and the use of [Co(bpy-pz)_3_]_3_]^3+/2+^ featuring chloride counter ions lead to a 5.5% PCE (*V*_OC_ = 0.9 V, *J*_SC_ = 8.1 mA cm^−2^) under full sun.^397^

For what concerns DSC operation in ambient light conditions, Venkatesan *et al.* used the Co(bpy)_3_ electrolyte in devices sensitized with different dyes.^398^ The best results were achieved with the Y123 dye, which yielded a PCE of 24.5% at 1000 lx light intensity.

Some disadvantages of cobalt complexes remain. They have a large molecular size leading to slow mass transport and diffusion, large reorganization energies between the oxidation states Co(ii) and Co(iii) increase the overall energy required to regenerate the dye, and their long-term stability is in question as the complexes in solution will likely undergo ligand exchange, which has to be structurally controlled.

###### Copper coordination complexes

As alternative redox mediators, Cu^2+/+^ complexes outperform both iodine- and Co-based electrolytes in combination with various dyes, which was made possible due to lower reorganization energy and minimized overpotential losses.^370,399^

The significant variations in coordination complex geometries between Cu(i) and Cu(ii) species, four-coordinate with tetrahedral geometry *vs.* four- to six-coordinate (square planar to tetragonal) geometry were anticipated to result in high reorganization energies. However, successful copper coordination complexes used in DSCs were developed by using sterically-hindered ligands to minimize the reorganization energy.

**Fig. 28:**
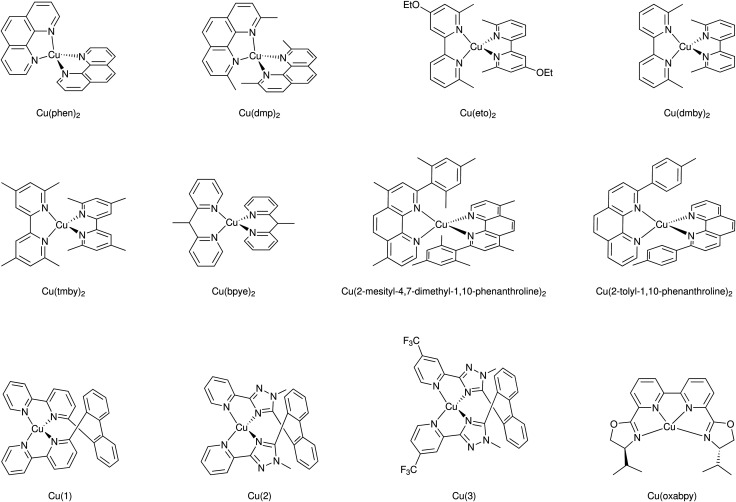
Chemical structures of copper coordination complexes-based redox mediators implemented in DSCs.

Hattori *et al.* achieved a maximum PCE of 1.4% for the first time using bis(2,9-dimethyl-1,10-phenantroline)copper(ii/i) complexes([Cu(dmp)_2_]^2+/+^), Fig. 28.^370^ This result was later improved by Bai *et al.*,^346^ who reached 7% PCE with the C218 organic dye followed by Freitag *et al.* in 2016, who achieved 8.3% PCE using the D–π–A LEG4 organic dye with a rather high open-circuit voltage of over 1.0 V. Freitag also discovered that the [Cu(dmp)_2_]^2+/+^ complex (redox potential of 0.93 V *vs.* NHE) can achieve good regeneration of the oxidized dye molecules with a driving force as small as 0.14 eV, thus minimizing internal energy losses.^96^ Cong *et al.* synthesised a novel Cu mediator – [Cu(bpye)_2_]^2+/+^ – featuring the 1,1-bis(2-pyridyl)ethane ligand. A PCE of 9.0% (*V*_OC_ = 0.90 V, *J*_SC_ = 14.1 mA cm^−2^) was achieved, which however declined to 6% after a short light ageing period.^372^ In 2017, Freitag and co-workers introduced two new redox couples based on Cu bipyridyl complexes, [Cu(dmby)_2_]^2+/+^ (0.97 V *vs.* NHE, dmby = 6,6′-dimethyl-2,2′-bipyridine) and [Cu(tmby)_2_]^2+/+^ (0.87 V *vs.* NHE, tmby = 4,4′,6,6′-tetramethyl-2,2′-bipyridine), which showed efficient organic Y123 dye regeneration at very low driving forces of 0.1 eV.^14^ The efficiency exceeded 10% under 1000 W m^−2^ AM1.5G illumination. In their follow-up work Saygili *et al.* examined the regeneration behavior and recombination processes of [Cu(dmby)_2_]^2+/+^, [Cu(tmby)_2_]^2+/+^, [Cu(eto)_2_]^2+/+^ (eto = 4-ethoxy-6,6′-dimethyl-2,2′-bipyridine), and [Cu(dmp)_2_]^2+/+^ in conjunction with organic dyes having various degrees of blocking groups: D5, D35, and D45.^95^ Their results indicated that DSCs with a combination of D35 and [Cu(dmp)_2_]^2+/+^ achieved a very high *V*_OC_ of 1.14 V without a decrease in *J*_SC_. Moreover, with a dye lacking recombination-preventing steric units such as D5, *V*_OC_ values as high as 1.13 V were possible with [Cu(dmp)_2_]^2+/+^ and [Cu(dmby)_2_]^2+/+^ electrolytes. Liu *et al.* introduced a series of indacenodithiophene (IDT)-based D–π–A organic dyes reaching high open-circuit voltage values (>1.1 V) and PCE values of 11.2% at 1 sun.^274^ Zhang *et al.* also employed [Cu(tmby)_2_]^2+/+^ in conjunction with the novel WS-72 dye, which reduced interfacial electron recombination.

Liquid-junction devices generated a notable *V*_OC_ of 1.1 V together with a PCE of 11.6% under simulated AM1.5G illumination. After drying the liquid electrolyte to create solid-state devices, the PCE increased to 11.7% (*J*_SC_ = 13.8 mA cm^−2^, *V*_OC_ = 1.07 V and FF = 79%).^273^

In 2017, the field of DSCs experienced a significant push towards indoor applications. Indoor illumination is very different to sun illumination, with an emission spectrum only in the visible and light intensities that are two to three orders of magnitude lower. With high power conversion efficiencies of indoor photovoltaics, the power output obtained under low light illumination is sufficient to power a range of wireless devices belonging to the family of Internet of Things (IoT). Freitag *et al.* developed a cosensitized DSC with D35 and XY1 dyes employing the [Cu(tmby)_2_]^2+/+^ redox couple. The reported PCE was 11.3% at 1 sun and 28.9% at 1000 lx (of a fluorescent light tube).^348^ A record PCE of 13.1% at full sun (and 32% at 1000 lx) was obtained by Cao *et al.* using a XY1 and Y123 dye mixture in conjunction with the [Cu(tmby)_2_]^2+/+^ redox mediator.^320^ In 2020, Michaels *et al.* presented co-sensitized DSCs, where the small organic dye L1 was combined with the XY1 dye to provide *V*_OC_ and performance values of 910 mV and 34.0%, respectively, at 1000 lx (11.5% at 1 sun). These DSCs were able to power IoT devices capable of machine learning under ambient light.^26^ The current record of DSC efficiency in ambient light, with a PCE of 34.5% at 1000 lx, belongs to Zhang *et al.* with devices featuring a MS5/XY1b co-sensitized photoanode and the [Cu(tmby)_2_]^2+/+^ redox couple.^12^

Phenathroline complexes were further developed by Magni *et al.* They compared [Cu(2-mesityl-4,7-dimethyl-1,10-phenanthroline)_2_]^2+/+^ with [Cu(dmp)_2_]^+^ and its oxidized form [Cu(dmp)_2_Cl]^+^, which is penta-coordinated. They achieved a maximum 4.4% PCE when coupling these electrolytes with the π-extended benzothiadiazole dye G3. They also analyzed the differences in the steric hindrance effect caused by either the methyl groups in [Cu(dmp)_2_]^+^ or the two mesityl rings of [Cu(2-mesityl-4,7-dimethyl-1,10-phenanthroline)_2_](PF_6_)_2_, proposing that the latter cause a smaller conformational modification upon oxidation/reduction compared to the former, acting as a “kiss-lock enclosure” that leads to a more negative redox potential.^351,400^

Colombo *et al.* developed novel [Cu(2-mesityl-4,7-dimethyl-1,10-phenanthroline)_2_]PF_6_ and [Cu(2,9-dimethyl-4,7-diphenyl-1,10-phenanthroline)_2_]PF_6_ redox couples with a Fe(ii) co-mediator for DSC applications^400^ and later introduced a series of Cu complexes with different substituents in the *α*-positions of phenanthroline, with appropriate redox potentials and a distorted tetragonal geometry.^401^ Dragonetti *et al.* studied a heteroleptic Cu dye with [Cu(2-*n*-butyl-1,10-phenanthroline)_2_]^2+/+^ and [Cu(dmp)_2_]^2+/+^ redox couples. [Cu(dmp)_2_]^2+/+^ devices yielded lower photocurrents compared to those based on [Cu(2-*n*-butyl-1,10-phenanthroline)_2_]^2+/+^ due to a higher extinction coefficient of the former, result in agreement with reduced IPCE values at 475 nm when the dmp-based electrolyte was employed.^371^ [Cu(2-*n*-butyl-1,10-phenanthroline)_2_]^2+/+^ with the new Cu-based dye D achieved the highest PCE of 2% (*J*_SC_ = 6.3 mA cm^−2^, *V*_OC_ = 0.61 V and FF = 0.53). The [Cu(2-mesityl-1,10-phenanthroline)_2_]^2+/+^ shuttle produced the best PCE of 3.7% under full sun (*J*_SC_ = 5.9 mA cm^−2^, *V*_OC_ = 0.81 V and FF = 0.77).^402^ Benazzi *et al.* developed homoleptic Cu complexes redox couples with low molar absorption coefficient with substituted 1,10-phenanthrolines ([Cu(2-tolyl-1,10-phenanthroline)_2_]^2+/+^, [Cu(2-phenyl-1,10-phenanthroline)_2_]^2+/+^, and [Cu(2-*n*-butyl-1,10-phenanthroline)_2_]^2+/+^.^373^

Another polypyridyl complex was presented by Hoffeditz *et al.*, a Cu redox shuttle with the 1,8-bis(2′-pyridyl)-3,6-dithiaoctane (PDTO) ligand. This work showed the ligand exchange processes in the electrolyte upon oxidation from Cu(i) to Cu(ii) with the common additive *t*BP.^403^ The impact of *t*BP substitution on Cu(ii) species of complexes with bidentate ligands was also studied by Wang *et al.*, who found that *t*BP replaces the original ligand to form the [Cu(*t*BP)_4_]^2+^ species, which is a poor electron acceptor, leading to high voltages and charge collection efficiencies.^404^ Heteroleptic Cu(i)-based dyes were investigated by Karpacheva *et al.* together with homoleptic Cu(ii/i) redox couples with a maximum efficiency of 2.06%. The researchers introduced electron-donating methoxy groups in Cu(4,4′-dimethoxy-6,6′-dimethyl-2,2′-bipyridine)_2_ to decrease the oxidation potential compared to Cu(dmby)_2_. The performance improvement with the former electrolyte was obtained thanks to a significant *J*_SC_ increase and despite a decrease in *V*_OC_.^405^

Michaels *et al.* introduced new copper complexes redox mediators with the tetradentate ligand 6,6′-bis(4-(*S*)-isopropyl-2-oxazolinyl)-2,2′-bipyridine – [Cu(oxabpy)]^2+/+^. The ligand allowed to lock the complex in a square-planar geometry, leading to minimized reorganization energies. The gel-like [Cu(oxabpy)]^2+/+^ complexes showed considerable enhancement of charge transport performance.^353^ In 2020 Rodrigues *et al.* introduced a series of three copper redox shuttles with pre-organized tetradentate ligands, which were tested computationally, electrochemically, and in solar cell devices for performance. The rigid tetradentate ligand design achieved a high *J*_SC_ (14.1 mA cm^−2^) and more effective electron transfer reactions, which led to an improved *V*_OC_ value for one of the copper redox shuttle-based devices.^352^

###### Iron coordination complexes

An electrolyte based on iron complexes is of high interest as it would represent a sustainable, low cost and non toxic option. In 2012 Daeneke *et al*. introduced an aqueous hexacyanoferrate electrolyte for DSC. With a high-extinction-coefficient organic dye, MK2, the assembled solar cells reached *V*_OC_ = 0.761 V, *J*_SC_ = 7.21 mA cm^−2^, FF = 75% and PCE = 4.1%.^374^ Previously, in 2011 Rutkowska *et al.* successfully implemented a polynuclear electronically/ionically (redox) conducting mixed-valent inorganic material such as nickel(ii) hexacyanoferrate(iii/ii) – ([NiFe(CN)_6_]^2−/−^ – with a redox potential of approximately 0.84 V *vs.* NHE, resulting in DSCs of *V*_OC_ = 0.790 V, *J*_SC_ = 8 mA cm^−2^, FF = 70% and PCE = 4%.^375^

**Fig. 29:**
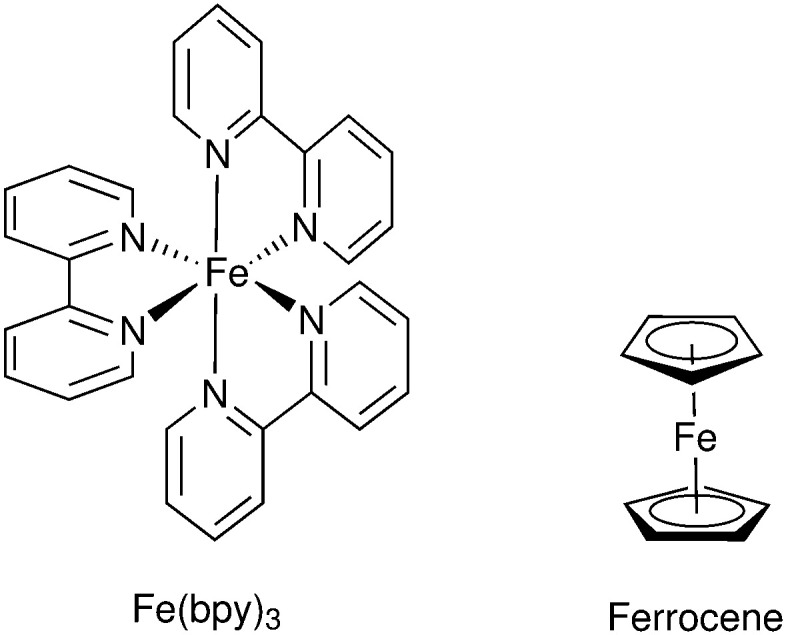
Chemical structures of iron coordination complexes-based redox mediators implemented in DSCs.

The bipyridine equivalent to cobalt complexes – [Fe(bpy)_3_]^3+/2+^ (Fig. 29) – has weaker Fe(ii)–N bonds than Co(ii)–N, resulting in a high redox potential of 1.37 V. Furthermore, the [Fe(bpy)_3_]^3+/2+^ redox couple is well known to be a stable, highly reversible redox system. The RR9 dye, with a low HOMO energy level, was designed to match the redox potential of [Fe(bpy)_3_]^3+/2+^ by Delcamp and co-workers. With a driving force of 0.19 eV, the DSCs reached a record *V*_OC_ of over 1.4 V and a PCE of 1.9%.^282^

The one-electron, outer-sphere iron-based redox couple ferrocenium/ferrocene (Fc^+^/Fc) has been extensively investigated in the DSC field thanks to its favourable kinetic properties and to its more positive redox potential, faster electron exchange and lower toxicity in comparison to the iodide/triiodide redox couple. Initial results showed that “plain” Fc^+^/Fc does not perform well in DSCs, due to high recombination of electrons from both the TiO_2_ layer and the substrate. Surface passivation, which included spray pyrolysis, atomic layer deposition (ALD), and silane treatment, was used to inhibit recombination. In a subsequent study, Daeneke *et al.* worked on reducing mass-transport limitations for electrolytes based on the Fc^+^/Fc redox couple, and addressed recombination issues by depositing thinner layers of TiO_2_ (18 nm blocking layer, 2.2 μm mesoporous layer and 4.4 μm scattering layer); *t*BP was also introduced in the electrolyte solution to further passivate the titania surface. Their devices were complemented by the Carbz-PAHTDTT organic dye and by a Pt counter electrode. Such devices performed better (*V*_OC_ = 0.842 V, *J*_SC_ = 12.2 mA cm^−2^, FF = 73%, and PCE = 7.5%) than reference DSCs (*V*_OC_ = 0.735 V, *J*_SC_ = 13.3 mA cm^−2^, FF = 62%, and PCE = 6.1%) and represent the best-performing cells based on the Fc electrolyte to date.^374,376^

###### Nickel coordination complexes

**Fig. 30:**
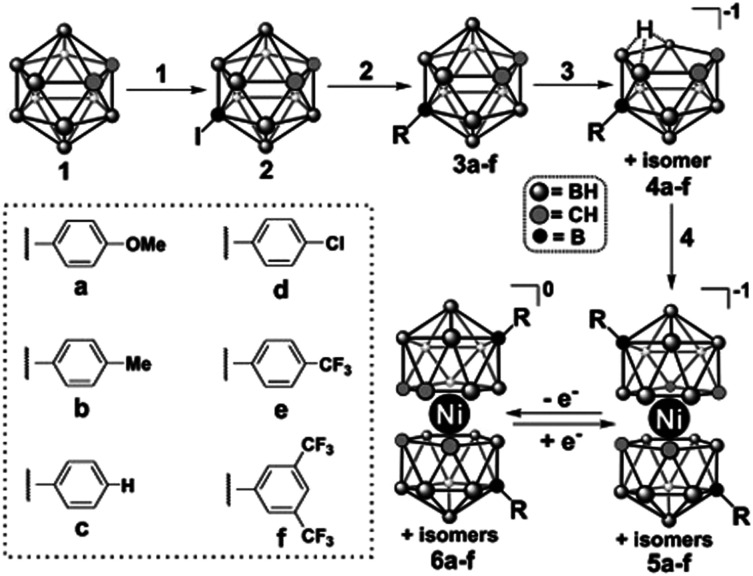
Starting with commercially available *o*-carborane, a five-step, high-yield synthetic strategy is used to create bis(dicarbollide) species from B(9)-functionalized derivatives of the parent carborane. Reprinted with permission from ref. 406. Copyright 2010 WILEY-VCH Verlag GmbH & Co. KGaA, Weinheim.

Nickel bipyridyl complexes have been tested in battery applications, where they can provide potentials in excess of 2.25 V, with very stable and pseudo-reversible anodic and cathodic half-cell reactions.^406,407^ For example Ni-bis(dicarbollide), which is comprised of two deboronated (*nido*-2) *o*-carborane ligands with *η*^5^ coordination, can perform several redox processes with net charges of −2, −1, and 0, corresponding to II–IV oxidation states of the Ni center (Fig. 30). Ni(iv/iii) bis(dicarbollide) complexes were used by Li *et al.* in DSCs, where they provided fast charge transport and a non-corrosive environment. Structural modification of the dicarbollide moiety at the B(9/12) positions with either electron donating or electron withdrawing groups allowed the creation of a class of ligands with different properties. These Ni(iv/iii)-dicarbollide mediators however had high reorganization energies during redox processes, which were due to a required *cis*-to-*trans* conformational rotation upon electron transfer and lead to low electron exchange rates.^408^ Spokoyny *et al.* created a series of redox mediators ranging in redox potentials from 0.37 V to 0.55 V *vs.* NHE and the highest *V*_OC_ was obtained for the 3,5-bis(trifluoromethyl)phenyl group, with *V*_OC_ = 0.850 V; PCEs were in the range between 0.7% and 2%. In DSCs with the N719 photosensitizer, the Ni redox couple with potential 0.77 V *vs.* NHE rendered a 1.5% efficiency, which was further improved up to *J*_SC_ = 6.3 mA cm^−2^ by modifying the photoanode with a nanoparticle-and-aerogel framework possessing a high surface area (13.6 μm thickness), which allowed to reach a PCE of 2.1% (*V*_OC_ = 0.628 V, *J*_SC_ = 5.3 mA cm^−2^, FF = 60%). Further investigations were focused on modification of Ni complexes and the search for an appropriate sensitizer to match this kind of redox couples.^406^

###### Manganese coordination complexes

The search for more sustainable and less toxic redox mediators based on coordination complexes for DSCs was extended to Mn(iv/iii) complexes. Manganese can be considered an interesting one-electron outer-sphere redox shuttle candidate because of its variety of accessible redox states (from +2 to +7), low toxicity and abundance. Ideally, the oxidized redox mediator species Mn_ox_, present at the TiO_2_ surface, should not significantly reduce the lifetime of TiO_2_ conduction band electrons before Mn_ox_ diffuses to the counter electrode. The undesired recombination reaction between electrons at the TiO_2_ surface and Mn_ox_ limits charge collection, as with the ferrocene/ferrocenium couple, and constrains the choice of alternative mediators, which require surface passivation. Some Mn(iii) complexes are known to undergo a spin change upon reduction (d^4^ to d^5^) that can slow down the undesired recombination.

**Fig. 31:**
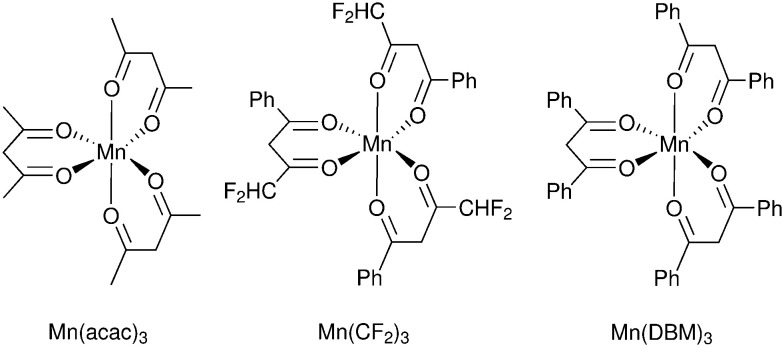
Chemical structures of manganese coordination complexes-based redox mediators implemented in DSCs.

The first example of application was reported in 2014 by Spiccia *et al.*, who focused on DSCs containing the commercially available [Mn(acac)_3_]^+/0^ (acac = acetylacetonate, Fig. 31) with a redox potential of 0.49 V *vs.* NHE and the MK2 dye, reporting an energy conversion efficiency of 4.4% under AM1.5G, 100 mW cm^−2^ conditions.^377^ Carli *et al.* followed up by developing the derivatives [Mn(CF_2_)_3_] (CF_2_ = 4,4-difluoro-1-phenylbutanate-1,3-dione) and [Mn(DBM)_3_] (DBM = dibenzoylmethanate).^378^ This series showed redox potentials in the range between 0.41 V and 0.69 V *vs.* NHE for [Mn(CF2)_3_]^3+/2+^ and [Mn(DBM)_3_]^3+/2+^.

###### Vanadium coordination complexes

**Fig. 32:**
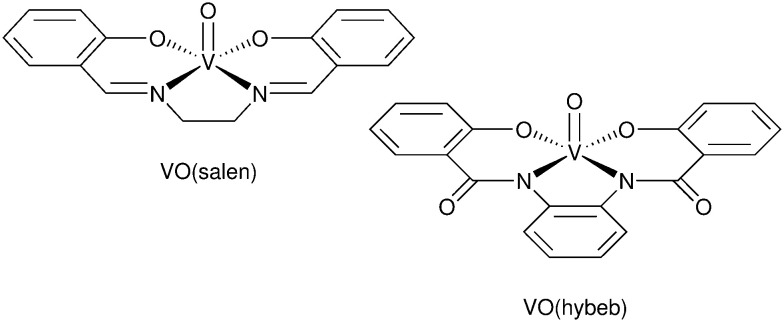
Chemical structures of vanadium coordination complexes-based redox mediators implemented in DSCs.

Fundamental electrochemical research on the kinetics and mechanisms of vanadium(v/iv) redox couple reactions in a range of electrolytes, especially for redox flow batteries, is ongoing. For DSCs, in 2013 Nishide and co-workers featured an electrochemically-reversible and fast redox mediator VO(salen) (salen = *N*,*N*′-ethylene-bis(salicylideneiminate)), Fig. 32, reaching a conversion efficiency of 5.4% (*V*_OC_ = 0.74 V and *J*_SC_ = 12.3 mA cm^−2^) in a co-sensitized DSC with D205/D131.^379^ In 2015 Apostolopoulou *et al.* introduced the oxidovanadium(iv) reversible redox couple [VO(hybeb)]^2−/−^ (where hybeb^4−^ is a tetradentate diaminodiphenolate ligand) with a very low redox potential of −0.047 V *vs.* NHE. The electrolyte was tested in DSCs with the N719 dye reaching a performance of 2% (*V*_OC_ = 0.66 V, *J*_SC_ = 5.2 mA cm^−2^) under 1 sun illumination.^380^

##### 4.3.1.3 Small organic molecules

**Fig. 33:**
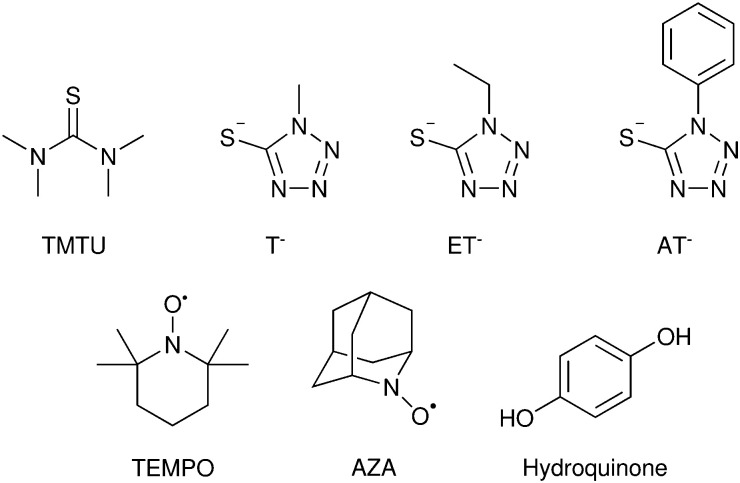
Chemical structures of small organic molecules-based redox mediators implemented in DSCs.

Various organic redox active molecules such as TEMPO^+^/TEMPO, AZA (2-azaadamantan-*N*-oxyl) Quinone or T^−^/T_2_ (T for 1-methyl-1-*H*-tetrazole-5-thiolate, T_2_ for the dimer) were tried to circumvent the limitations that still exist with coordination complex redox couples, including inefficient dye regeneration, mass transport limitations of large metal complexes or high electron recombination with the fast outer-sphere redox systems (Fig. 33).

In 2012 Burschka *et al.* reached a power conversion efficiency of 7.9% with a DSC based on the T^−^/T_2_ redox couple together with a PEDOT counter electrode.^357^ In the same year, Li *et al.* introduced a new thiolate/disulfide redox couple AT^−^/BAT,^381^ an analogue to T^−^/T_2_ with more positive redox potential and slower charge recombination reaching promising efficiencies of 6.07%. A year later, supramolecular lithium cation assemblies of crown ether were been used to replace conventional tetraalkylammonium counter-ions in thiolate/disulfide (ET^−^/BET)-mediated dye-sensitized solar cells, which exhibited high stability and efficiency of 6.61% under 1 sun illumination.^382^

The redox-active TEMPO was successfully implemented into DSCs as a redox mediator by Grätzel *et al.* and it improved the *V*_OC_ over the I^−^/I_3_^−^ electrolyte.^383^ Nitroxide derivatives were also studied as DSC mediators by other groups. However, the *V*_OC_ was enhanced to the detriment of the cell's short-circuit current density.^356,409^

Another organic radical – 2-azaadamantan-*N*-oxyl (AZA) – was used as a stable and highly reactive redox mediator in a DSC. AZA exhibited both an appropriate redox potential and significantly high values of diffusivity, heterogeneous electron-transfer rate, and electron self-exchange reaction rate. These properties gave rise to an enhanced electron-transfer mediation, which lead to a high fill factor and thus excellent photovoltaic performance to achieve a conversion efficiency of 8.6%.^384^

Liu *et al.* developed indoline- and ruthenium-based dye-sensitized solar cells with the organic redox couple tetramethylthiourea/tetramethylformaminium disulfide (TMTU/TMFDS^2+^). This redox couple worked best with the indoline dye D205, reaching a power conversion efficiency of 7.6% under AM1.5G 1 sun illumination. TMTU provided efficient charge collection and injection in all studied devices; however, while regeneration of indoline dyes was also very effective, the regeneration of ruthenium dyes was less so, leading to the decreased performance.^385^

The hydroquinone/benzoquinone (HQ/BQ) redox pair has increased interest in research as the electron transfer of the redox couple is a thermodynamically reversible process.^387^ In previous reports, the anionic hydroquinone species (TMAHQ/BQ) was used as a redox mediator in DSCs with the N719 dye as sensitizer and Pt as CE; these systems showed promising photovoltaic characteristics (*V*_OC_ = 75 5 mV, *J*_SC_ = 1 0.28 mA cm^−2^, FF = 66.7%, and PCE = 5.2 %). With the same redox mediator but with PEDOT as counter electrode and the organic dye CM-309, the following parameters were achieved: *V*_OC_ = 755 mV, *J*_SC_ = 12.10 mA cm^−2^, FF 67.8%, and PCE = 6.2%.^386^

##### 4.3.1.4 Ionic liquids

**Fig. 34:**
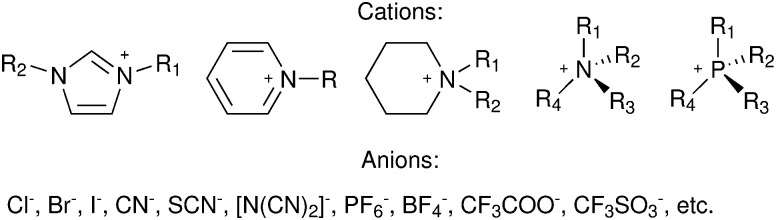
Examples of cations and anions used in ionic liquids.

The use of liquid electrolytes demands perfect sealing of the device to avoid leakage and evaporation of the solvents. To eliminate electrolyte leakage issues in traditional DSCs (*i.e.* cells with organic solvent-based electrolytes), ionic liquids are used as the electrolyte to improve cell durability. An ionic liquid (IL) is defined as a salt that is liquid at the operational temperature. From a DSC point of view, these molten salts can be described as electrolytes comprised solely of ions.^333,410–412^ Technically, the difference between ionic liquids and molten salts is given by the melting temperature and some physical characteristics: the former melt below 100 °C and present relatively low viscosity, while the latter melt at high temperatures and are more viscous. When the melting temperature is below 25 °C, we talk about room temperature ionic liquids (RTILs). Ionic liquids (Fig. 34) have found large use as electrolytes in DSCs thanks to the fact that they are chemically and thermally stable, that their viscosity can be adjusted as needed, that they are mostly non-flammable, that they possess high ionic conductivity, and that they are non-reactive in a large range of potentials. From a stability point of view, it is crucial that they have very low vapor pressure, which mitigates evaporation and leaking issues in devices. ILs can play two different roles within DSC electrolytes: they can act as solvents in fully liquid devices, and as organic salts in quasi-solid-state devices. These properties have made ILs a sustainable solution to the problematic use of organic solvents, and ILs with different substituents and ions were prepared and used as redox mediators in dye-sensitized solar cells.

Best performances with ILs were reached with imidazolium-based ionic electrolytes. Other IL cations employed are sulfonium, guanidinium, ammonium, pyridinium, or phosphonium, which were also tested as solvent-free electrolytes. The limitations in low diffusion and charge mobility of ILs in comparison to redox mediators in organic solvents remain. Several strategies were employed to improve the mass transport limitations by diluting the ionic liquid with organic solvents, compromising the system with the high volatility of organic solvents. Even in ILs with particularly low viscosity such as imidazolium dicyanamide, the diffusion of the triiodide anion is troublesome at low temperature, while efficiency at high temperature is limited by recombination reactions. An example of low-viscosity electrolytes is represented by the mixture of EMImSCN and PMImI ILs. The diffusion coefficient of triiodide in such electrolyte was 2.95 × 10^−7^ cm^2^ s^−1^, a value 1.6 times higher compared to an electrolyte comprised of PMImI only. DSCs fabricated with this mixed electrolyte in conjunction with the Z907 dye reached a PCE of 7%. ILs' potential advantage over organic solvents remains to be proved, while it is necessary to overcome the main drawbacks of high viscosity and low ion mobility.^413^

##### 4.3.1.5 Quasi-solid and solid polymer electrolytes

Depending on fabrication strategies, the inclusion of polymers can lead to either quasi-solid (gel) or solid electrolytes. In the former case, the polymer acts as a host matrix for a liquid electrolyte, and it swells to accommodate the liquid inside, forming a gelatinous material that prevents solvent leakage. In the latter case, the redox active components of the charge transport layer are embedded directly within the polymeric structure, without the presence of a solvent.

###### Gels and quasi-solid polymers

**Fig. 35:**
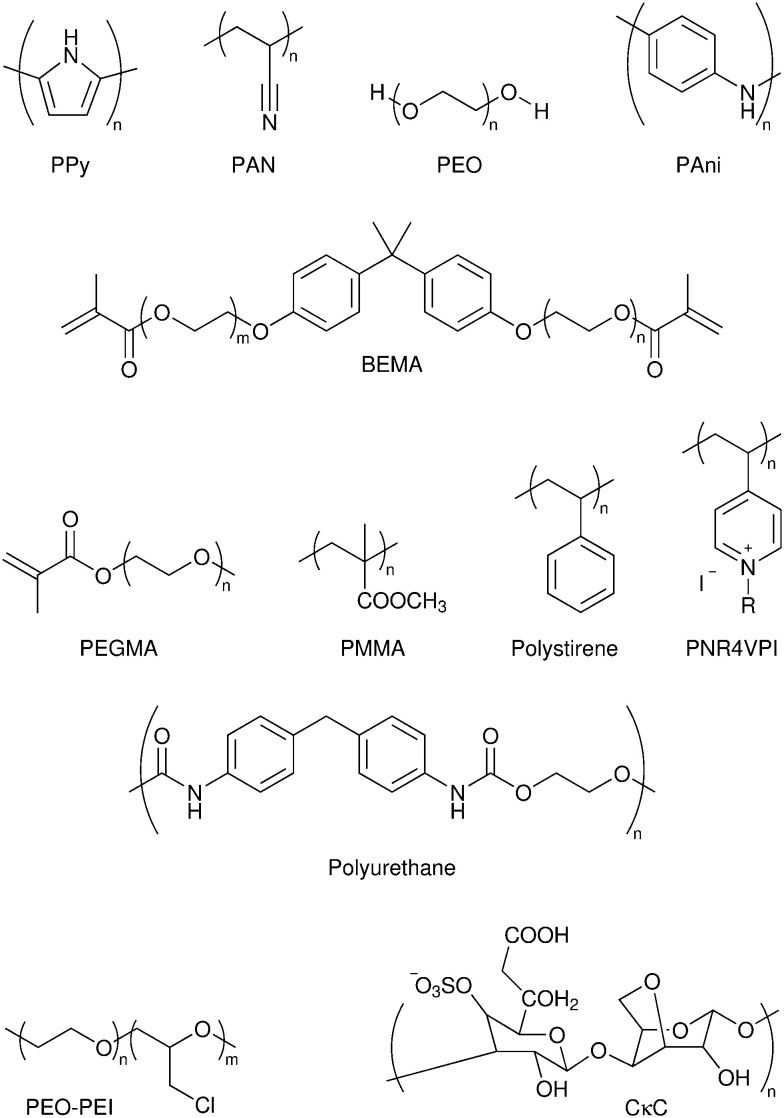
Chemical structures of polymer electrolytes used in DSCs.

Gel polymer electrolytes (GPEs) are designed to swell and host a liquid electrolyte in the order of tens to hundreds of times their own weight. They can infiltrate and create a contact with the photoanode very effectively in order to ensure fast dye regeneration and, at the same time, possess high conductivity, which leads to quick transport of charges towards the counter electrode.^333,338,412,414–420^ Polyacrylonitrile (PAN), poly(ethylene oxide) (PEO) derivatives, conducting polymers includ ing polypyrrole (PPy), polyaniline (PAni) and other polymers are the typical host materials (Fig. 35). Dimethyl carbonate (DMC), propylene carbonate (PC) and ethylene carbonate (EC) can be used as organic plasticizers with a large variety of polar solvents, ionic liquids and salts.^421,422^ A good portion of GPE work in DSCs can be credited to Bella and co-workers, as they showed long-term stability and efficiency of gel electrolytes. The specific approach to create an *in situ* electrolyte comprises the expansion of a monomer – bisphenol-A-ethoxylate dimethacrylate (BEMA) or poly(ethylene glycol) methyl ether methacrylate (PEGMA) – as well as a photoinitiator into the electrolyte and UV exposure of the assembled solar cell. To prove long-term stability, a DSC fabricated using this method with the LEG4 dye and an electrolyte containing the [Co(bpy)_3_]^3+/2+^ redox mediator was first placed in the dark at 60 °C for 1500 h and then subsequently exposed to full sun irradiation for 300 h at 40 °C. At the end of the ageing test the device (initial PCE of 6%) retained 90% of its initial performance.^359,423–425^ Using polyethylene glycol diacrylate (PEGDA) and PEGMA as copolymers, power conversion efficiencies of up to 4.41% (Table 5) were recorded.^426^ After inserting fillers based on metal organic frameworks (MOFs) or micro-fibrillated cellulose (MFC) into BEMA or PEGDA and PEGMA polymer blends, a dramatic increase in PCE (up to 7.03%) was observed.^340,427,428^

**Table 5:** Photovoltaic characteristics of DSCs based on polymer electrolytes

Matrix/polymer	Salt	Sensitizer	*V*_OC_ (mV)	*J*_SC_ (mA cm^−2^)	FF (%)	PCE (%)	Year	Ref.
PPVII	None	N719	637	13.61	71	6.18	2014	412
MPII:SiO_2_	I_2_, NMBI	Z907	700	13.67	73.1	7.0	2003	414
PVP	I_2_, KI, BMII	N3	626	15.72	55	5.41	2010	415
PVDF-HFP	I_2_, NMBI, DMPII	Z907	730	12.5	67	6.1	2003	416
BEMA:PEGMA	Co(bpy)_3_	LEG4	880	10.5	68	6.4	2015	417
BEMA:PEGMA	I_2_, NaI	N719	580	16.0	58	5.41	2013	424
PAN-VA	I_2_, LiI, *t*BP, DMPII	CYC-B11	743	18.8	76	10.58	2013	418
SGT-626	I_2_, LiI, *t*BP, DMPII	N719	764	17.55	72.53	9.72	2020	419
SGT-612	I_2_, LiI, *t*BP, DMPII	N719	782	15.27	76.6	9.1	2021	420
P(EO-EPI)	I_2_, LiI	N3	670	9.1	54	3.3	2008	421
Gelator 1	I_2_, LiI, DMPII	N719	670	12.8	67	5.91	2001	422
Gelator 2	I_2_, LiI, DMPII	N719	632	11.1	65.8	4.62	2001	422
Gelator 3	I_2_, LiI, DMPII	N719	640	11.1	63.4	4.49	2001	422
Gelator 4	I_2_, LiI, DMPII	N719	623	11.2	66.4	4.67	2001	422
PEO:CMC	I_2_, NaI, MPII	N719	750	10.03	69	5.18	2013	423
PEGDA:PEGMA	I_2_, NaI	N719	499	17.46	52	4.41	2014	426
Cellulose	I_2_, LiI, MPII	N719	590	8.39	67	3.33	2014	340
PEGDA:PEGMA:Mg-MOF	I_2_, NaI, MPII	N719	690	12.6	55	4.80	2013	427
BEMA:PEGMA:MFC	I_2_, NaI	N719	760	15.2	61	7.03	2014	428
PMMA	I_2_, BMII	N719	750	15.53	69	8.03	2013	358
Polystyrene beads	I_2_, BMII	N719	770	15.3	64	7.54	2012	429
Polyurethane	I_2_, LiI, BMII	N719	740	14.97	55	6.1	2011	430
PEO:TiO_2_	I_2_, LiI	N3	664	7.2	57.5	4.19	2002	431
HEII	I_2_, LiI, NMBI, MPII	MK2	733	14.66	69.3	7.45	2013	432
CkC	I_2_, NaI	N719	510	7.60	53	2.06	2015	433

The classic conductivity and diffusivity of the iodine/1-butyl-3-methylimidazolium iodide (BMII) redox system was similar to that of liquid electrolytes and, relative to conventional liquid DSCs, the resulting cells displayed increased stability.^358^ For devices filled with liquid electrolyte and directed dissolution of polystyrene nanobeads on the counter electrode, resulting in a gel electrolyte, PCEs of 7.54% were registered. The PCE of those devices was close to that of DSCs based on liquid electrolytes (7.59%).^429^ Finally, when polyurethane was used as gelation matrix, a PCE up to 6.1% was obtained.^430^

Some research has focused on the use of different nanosized additives, also known as nanofillers (NFs), to enhance charge transport in quasi-solid and solid electrolytes in order to improve solar cell stability and efficiency. Clays, metal oxides, metal nitrides, metal carbides, metal sulphides, and carbonaceous materials may all be used as nanofillers.^388,434^

Seo *et al.* used a combination of a PEO-based composite polymer electrolyte with I^−^/I_3_^−^ redox mediator and 5 wt% TiO_2_ nanoparticles, which not only improved the *V*_OC_ , but also the energy conversion efficiency to 9.2% at 100 mW cm^−2^ illumination.^435^ Lee and co-workers have made significant advances since then, including further development of titanium dioxide (TiO_2_) and titanium carbide (TiC) nanoparticles,^436^ and, most significantly, graphene oxide sponge (GOS) as nanofillers.^437^ The conversion efficiency of DSCs with TiO_2_ nanoparticles as filler was 7.65% in PEO, which is considerably lower than that of the liquid electrolyte reference devices with PCE of 8.34%. The fabricated liquid and quasi-solid DSCs employing TiC nanofillers both obtained a conversion efficiency of 6.3%. By using poly(vinylidene fluoride) PVDF as a co-regulating agent, the quasi-solid solar cells with TiO_2_ nanofillers achieved an efficiency of 8.32%, comparable to the liquid electrolyte. Furthermore, by including 4 wt% TiO_2_ nanoparticles as fillers into the printable electrolyte, the PCE was improved to 8.91%. The DSCs remained stable at 50 °C for 1000 h. The GOS nanofillers were added at a concentration of 1.5 wt% in printable electrolytes based on PEO and PVDF for quasi-solid-state dye-sensitized solar cells reaching energy conversion efficiency of 8.88%. Lee *et al.* also contributed to the development of quasi-solid-state dye-sensitized solar cells for low light conditions,^438–440^ with the electrolyte specifically optimised with poly(vinylidene fluoride-*co*-hexafluoropropylene) (PVDF-HFP). This was used to prepare polymer gel electrolytes as a gelator of liquid electrolytes with zinc oxide nanofillers resulting in a good performance at 200 lx of 20.11%.^441^ In addition, Ramesh and co-workers created a gel electrolyte with PVDF-HFP and PEO with SiO_2_ as nanofiller and the I^−^/I_3_^−^ redox pair having a high ionic conductivity of 8.84 mS cm^−1^ and resulting in DSCs with a PCE of 9.44%.^442^ Kim and co-workers also presented two types of triblock copolymers prepared by using functionized PEG as macro-RAFT agents: PEG-*b*-(P(AN-*co*-BMAAm))_2_ (SGT-602) and PEG-*b*-(P(AN-*co*-DMAAm))_2_ (SGT-604) with 13–15 wt% TiO_2_ nanofillers introduced into the gel electrolytes, resulting in efficiencies of 9.30% and 9.39% with SGT-602 and SGT-604, respectively.^443^

###### Solid polymers

Polymer electrolytes (PEs) aim at combining the advantages of liquid electrolytes (high ionic conductivity, diffusive transport, and interfacial contact characteristics) with the mechanical benefits of a polymer's resilience and flexibility.^333,338,444,445^ The majority of inorganic conductors in a host polymer consist of lithium salts (LiI, NaI, LiClO_4_, LiCF_3_SO_3_, LiSCN, NaSCN, NaClO_4_, LiPF_6_, *etc.*).

The selection of polymer hosts for PEs is based on the following characteristics: sufficiently polar and/or groups to form strong cation coordination and low impediment to bond rotation. Poly(ethylene oxide) (PEO) is the host polymer most widely used,^410,431^ although these systems typically exhibit poor conductivity (10^−8^ S cm^−1^),^333^ which can be increased with the use of blends of various polymers or copolymers and synthetically adapted monomers (Fig. 35).^333,446,447^

Li *et al.* introduced a solid-state electrolyte based on an imidazolium iodide compound co-functionalized with hydroxyethyl and ester groups (HEII) and studied the effect that different substituents on the imidazolium ring have on the ionic conductivity of the electrolyte and on the efficiency of solid-state DSCs built with it.^432^ Bella *et al.* contributed by constructing biodegradable polymers derived from seaweed as green chemistry-based PE. Carboxymethyl-da-caraageenan (CkC) and NaI/I_2_-based DSCs display high efficiency of power conversion up to 2.06%.^433^ Shortcomings of PEs are connected to insufficient pore filling and ionic conduction, which lead to low dye regeneration rates and fast electron recombination at the interfaces between the solid polymer electrolyte and the dye or the metal oxide semiconductor.

#### 4.3.2 Hole transport materials

Hole transporting materials (HTMs) transport charges within the materials themselves, not *via* movement of ions.^448,449^ As such, their mechanism of charge transport is best defined as electronic (or charge) hopping rather than diffusion. Due to the lack of molecular movement, solid-state DSCs (ssDSCs) based on an HTM layer work similarly to liquid DSCs while also maintaining the advantages of a solid-state system. For efficient DSCs, rapid carrier transport and low recombination rates are always necessary. In PV technology, good electronic and optical properties are not the only concern; stability also plays a very important role. On this regard, the choice of HTM can have a big impact on the stability of devices. The HTM needs to fulfill several requirements in order to allow the conversion of light to electricity during device operation: (i) its energy levels have to be compatible with the dye of choice. Its HOMO level (or valence band edge, VB) should be higher but close to that of the dye, in order to minimize the potential loss during charge (hole) transfer, while ensuring proper dye regeneration. At the same time, its LUMO level (or conduction band edge, CB) should be much higher than that of the dye, to deny back transfer of excited electrons. (ii) It needs to have good electronical properties such as high carrier mobility and long diffusion length in order to avoid charge losses during the extraction and transport processes. (iii) It needs to be chemically stable during both device fabrication and operation, which includes stability towards UV light, moisture, heat and oxygen. (iv) It should provide low operational costs, from both a purchase and a processing point of view.

New limitations emerge in the manufacturing of dye-sensitized solar cells that arise from the use of solid-state materials, such as poor pore filling of the mesoporous oxide layer. If large molecules with long molecular chains are introduced to mesoporous materials, they are unable to completely penetrate the mesoporous network.^450–453^ However, in 2011, Burschka *et al.* presented a ssDSC featuring spiro-OMeTAD with a PCE of 7.2%, thanks to a careful HTM layer optimization with the addition of p-dopants into the precursor solution.^454^ Given the high performance reached by Burschka, spiro-OMeTAD is often used as a benchmark HTM when presenting new ones, and it has therefore been used in combination with a large number of dyes.^455–459^ Nevertheless, this material poses many issues and a consensus has been established that affordable, new materials must be sought before ssDSCs' commercial feasibility can be achieved. More in depth, spiro-OMeTAD suffers from poor conductivity and hole mobility unless dopants are used, and it lacks stability over time.^458,460,461^

##### 4.3.2.1 Organic hole transport materials

Many organic compounds have been investigated as hole transfer materials for ssDSCs. The variety in synthesis helps researchers to develop new materials with the desired properties. New compounds allow the fine-tuning of energy levels, electronic properties, film-forming properties, and solubility in different solvents. Organic hole transport materials have well-defined compositions and molecular weights that ensures consistent properties in different batches. Compared to other compounds, these smaller molecules are better in penetrating the mesoporous layer of the photoanode.^462,463^

**Fig. 36:**
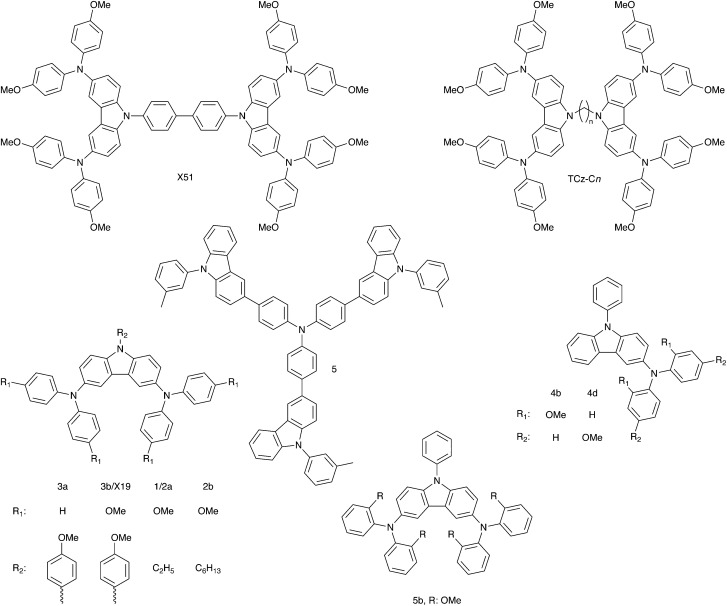
Examples of carbazole-based organic hole conductors.

**Fig. 37:**
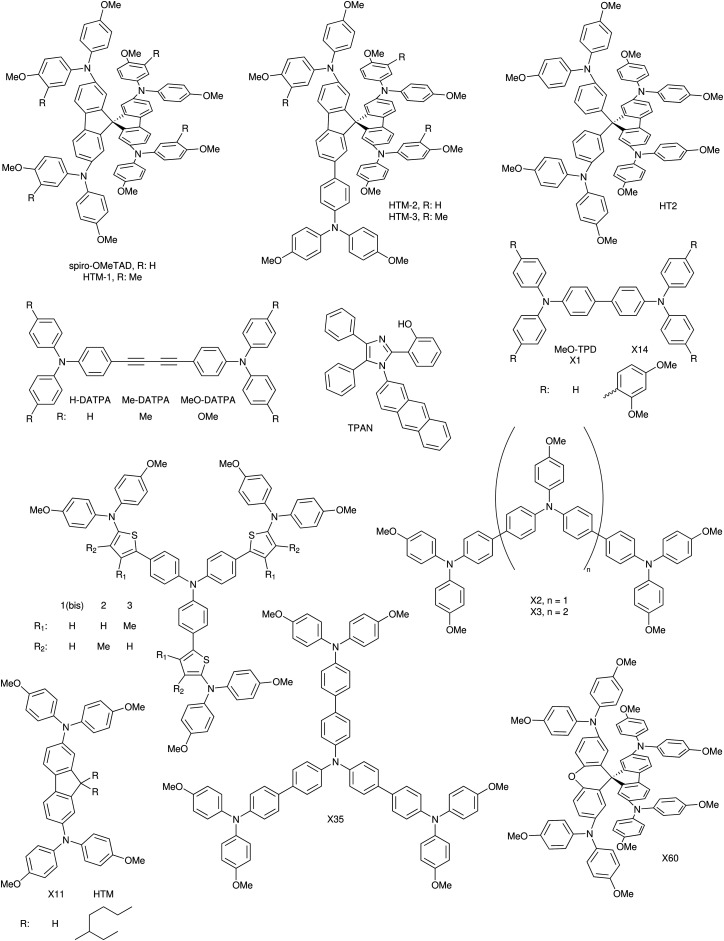
Examples of triphenylamine-based organic hole conductors.

Organic small molecules are the most common class of novel HTMs for ssDSCs. Most of the compounds referenced in this review have a triphenylamine (TPA) donor component in their composition: the nitrogen atom is a strong hole acceptor due to its lone electron pair and it is aided by the presence of three extra phenyl groups. It is possible to tune the energy levels of molecules containing the TPA group by adding substituents – usually the electron-donating group methoxy – to the aromatic rings not connected to the main body of the molecule. The methoxy group, in fact, destabilizes the electronic cloud in the TPA.^464^ A list of small molecular HTMs is reported along with their related dye and conversion efficiency in Table 6, and their chemical structures are represented in Fig. 36 and 37.

**Table 6:** Photovoltaic characteristics of DSCs implementing organic (small molecular and polymeric) hole transporting materials

HTM	Sensitizer	*V*_OC_ (mV)	*J*_SC_ (mA cm^−2^)	FF (%)	PCE (%)	Year	Ref.
Spiro-OMeTAD	Y123	986	9.5	76	7.2	2011	454
Spiro-OMeTAD	D102	710	8.06	53	3.03	2018	455
Spiro-OMeTAD	MKA253	780	12.4	63	6.1	2015	456
Spiro-OMeTAD	Z907	750	7.28	64	3.5	2013	457
Spiro-OMeTAD	ID504	760	9.76	64	4.8	2015	458
Spiro-OMeTAD	LEG4	900	10.10	70	6.36	2016	459
3a	D102	860	0.32	44	0.12	2014	465
3b	D102	680	6.32	41	1.75	2014	465
X19	LEG4	750	9.62	62	4.5	2014	466
X51	LEG4	920	9.27	70	6.0	2014	466
TCz-C3	D102	690	6.27	51	2.21	2018	455
TCz-C6	D102	590	0.86	38	0.20	2018	455
TCz-C12	D102	660	0.21	34	0.05	2018	455
H-DATPA	D102	620	0.67	37	0.15	2013	467
Me-DATPA	D102	700	1.13	43	0.34	2013	467
MeO-DATPA	D102	890	1.93	67	1.16	2013	467
MeO-TPD	LEG4	800	9.5	65	4.9	2013	468
HTM	Z907	750	8.5	51	3.3	2014	469
X1	MKA253	680	5.8	58	2.3	2015	456
X1	LEG4	880	9.44	69	5.8	2017	470
X11	MKA253	580	4.7	62	1.7	2015	456
X11	LEG4	655	8.2	55	3.0	2015	456
X2	LEG4	810	9.79	63	5.0	2015	471
X35	LEG4	890	9.81	63	5.5	2015	471
X3	LEG4	900	9.70	66	5.8	2013	457
X3	Z907	720	8.10	63	3.7	2013	457
X14	LEG4	910	9.71	71	6.1	2017	470
HTM1	ID504	820	9.34	63	4.8	2015	458
HTM2	ID504	800	7.08	38	2.2	2015	458
HTM3	ID504	800	7.00	38	2.1	2015	458
X60	LEG4	890	11.38	72	7.30	2016	472
PProDOT	N719	630	10.0	56	3.5	2012	473
PEDOP	D35	825	7.99	66	4.34	2014	474
PEDOP	D21L6	645	7.92	59	3.05	2014	474
PEDOP	Z907	440	1.97	53	0.46	2014	474
PEDOT	DPP07	770	11.13	65	5.54	2016	475
PPP-*b*-P3HT	CYC-B11	810	8.81	65.2	4.65	2014	476
P3HT	CYC-B11	750	7.71	61.1	3.53	2014	476
P3HT	N3	628	6.29	43	1.70	2014	477
P3HT	BzTCA	880	8.22	44	3.21	2014	477
P3HT	D102	720	11.37	58	4.78	2017	478

Debia *et al.* and Xu *et al.* concurrently developed an HTM (3b^465^ and X19,^466^ respectively) based on a carbazole core with a *p*-methoxyphenyl moiety attached to its nitrogen atom and a di(*p*-methoxyphenyl)amino group connected in *para* to each of its phenyl rings. In ssDSCs, 3b was tested with the D102 dye, while X19 with LEG4. These reports provide a good opportunity to highlight the importance of a good dye-HTM combination for what concerns the efficiency of charge transfer. In fact, the best device with 3b-D102 had a PCE of 1.75% (*V*_OC_ of 680 mV, *J*_SC_ of 6.32 mA cm^−2^, FF of 41%), while that with X19-LEG4 had a PCE of 4.5% (*V*_OC_ of 750 mV, *J*_SC_ of 9.62 mA cm^−2^, FF of 62%). The higher current in the latter case can be attributed to different light absorption properties of the two dyes, while the higher *V*_OC_ and FF are due to a lower series resistance. In a subsequent investigation, Xu *et al.* presented X51, also based on a carbazole core.^466^ X19 and X51 are structurally similar, but in the latter case there are two carbazole units that are linked together by a biphenyl moiety bonded to the carbazole nitrogens. As a result, X51's molecular weight almost doubles that of X19. X51 is more conductive than X19, leading to a reduced *R*_S_ in DSCs, allowing these devices to reach a PCE of 6.0% (*V*_OC_ of 920 mV, *J*_SC_ of 9.27 mA cm^−2^, FF of 70%). Benhattab *et al.* also connected two carbazoles together, but in this case with alkyl linkers of different lengths (propyl, TCz-C3; hexyl, TCz-C6; and dodecyl, TCz-T12), thus disconnecting electronically the two half molecules. Rather than increasing conjugation as in the case of X51, their efforts were directed to optimize the morphology of the HTM film. The best result was obtained with TCz-C3, with devices reaching a *V*_OC_ of 690 mV, *J*_SC_ of 6.27 mA cm^−2^, FF of 51% and PCE of 2.21%.^455^

Planells *et al.* studied four HTMs shaped as rods and comprised of a linear diacetylene core connecting two TPA groups.^467^ No devices were fabricated with MeS-DATPA (Fig. 37), while cell parameters were *V*_OC_ = 620 mV, *J*_SC_ = 0.67 mA cm^−2^, FF = 37% and PCE = 0.15% for H-DATPA; *V*_OC_ = 700 mV, *J*_SC_ = 1.13 mA cm^−2^, FF = 43% and PCE = 0.34% for Me-DATPA; and *V*_OC_ = 890 mV, *J*_SC_ = 1.93 mA cm^−2^, FF = 67% and PCE = 1.16% for MeO-DATPA. Johansson and co-workers demonstrated that light soaking of full DSCs dramatically improves the efficiency of the solar cell, indicating that ion migration occurs in the solid-state layer. The PCE of their MeO-TPD-based solar cells improved from 1.1% to 4.9% after light soaking.^468^ Yuan *et al.* and Liu *et al.* introduced new HTMs – HTM^469^ and X11^456^ – featuring a fluorene center and *p*-methoxyphenylamino groups connected to each benzene ring. A ssDSC with HTM reached a PCE of 3.3%, while one with X11 reached a PCE of 1.7% with the MKA253 sensitizer and of 3.0% with the LEG4 sensitizer.

Sun and co-workers designed a series of *p*-methoxy-substituted triphenylamine oligomers, which they used to make X1, X2, X3 and X35.^457,471^ Optimized devices led to the conclusion that to an increase in number of repeating units corresponded an increase in performance (see Table 6 for champion device details, for X3-based devices *V*_OC_ was 880 mV, *J*_SC_ was 9.23 mA cm^−2^, FF was 62% and PCE was 5.4%). Another effective hole conductor, X14, was created by Sun, Kloo and co-workers. This molecule also presented an expanded aromatic conjugation, since it featured *o,p*-dimethoxy-substituted phenyl moieties in place of the methoxy groups of X1. The extended conjugation deepened the HOMO level of X14 of about 200 meV compared to X1, while doubling the hole mobility of the former compared to the latter when adding LiTFSI to the HTM layer composition. In the experiments, solar cell efficiency was comparable between the two hole transporting materials. The best X1 samples were the ones that had a PCE of 5.8%, while those with X14 had a PCE of 6.1%. For comparison, the best device based on spiro-OMeTAD displayed a PCE of 5.9%.^470^ Malinauskas *et al.* have conducted a study on the long-term stability of spiro-OMeTAD-derived DSCs. They noticed that crystalline domains formed in the originally amorphous spiro-OMeTAD film when the devices were held at 60 °C, which proved the cause of the poor performance of those devices.^458^ In order to circumvent this limitation they changed spiro-OMeTAD's molecular structure to incorporate asymmetry, reaching high performances with a *V*_OC_ of 820 mV, *J*_SC_ of 9.34 mA cm^−2^, FF of 63% and PCE of 4.8%. HTM-2 and HTM-3, which were more substituted, were also less efficient, with a *V*_OC_ of 800 mV, *J*_SC_ of 7.08 mA cm^−2^, FF of 38% and PCE of 2.2%; and a *V*_OC_ of 800 mV, *J*_SC_ of 7.00 mA cm^−2^, FF of 38% and PCE of 2.1%; respectively.

Xu *et al.* synthesized X60, the only HTM that could provide comparable results with the benchmark set by Burschka. X60 has a spiro[fluorene-9,9′-xanthene] core linked to *p*-methoxy substituted diphenylamine side groups, and its spiro moiety costs less than 30 times that of spiro-OMeTAD. They did not have a spiro-OMeTAD-based reference cell, but an X60-based one featured a *V*_OC_ of 890 mV, *J*_SC_ of 11.38 mA cm^−2^, FF of 72% and PCE of 7.30%.^472^

##### 4.3.2.2 Polymeric hole transporting materials

Using polymers in ssDSCs is more difficult than using small molecules. In practice, for a compound to have excellent electronic properties is not enough. It is also critical to design the device such that the material may permeate the mesosoporous metal oxide and regenerate the dye. Most of the polymers examined here are capable of *in situ* polymerization; due to this process, monomer molecules can infiltrate the system, and after polymerization, the typically greater conductivity of macromolecules may be utilized. Each article delves into the polymerization process and also refers to the overall structure and characteristics of the monomer itself.

**Fig. 38:**
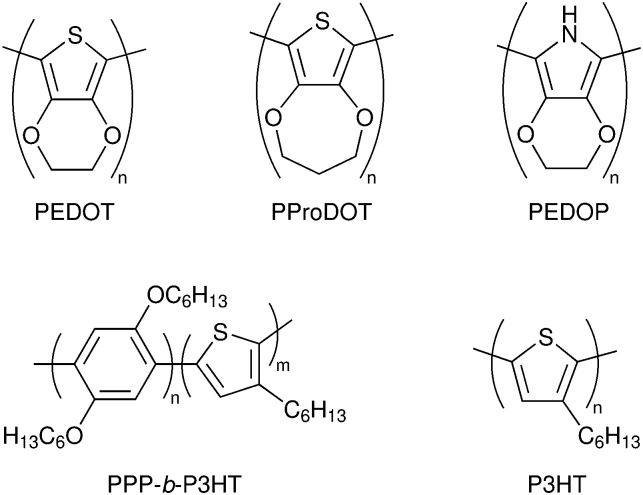
Examples of polymeric hole conductors.

Kim and co-workers introduced a polymer based on a propylenedioxythiophene monomer, ProDOT (Fig. 38).^473^ PProDOT is similar in structure to PEDOT, but it contains a propylene chain rather than an ethylene one. They employed a solid-state polymerization method in which a dibrominated ProDOT monomer was the starting material. This method is sluggish, but also very inexpensive. A solution of monomers was sprayed onto the photoanode. The solid monomer was put in an oven that was heated at 25 °C and allowed for polymerization to occur with the evaporation of Br_2_ as a side product. *Via* coupling with a platinized FTO counter electrode, *V*_OC_ of 630 mV, *J*_SC_ of 10.0 mA cm^−2^, FF of 56% and PCE of 3.5% was reached in terms of photovoltaic performance. Zhang *et al.* demonstrated the efficiency of PEDOP (poly(ethylenedioxypyrrole)) combined with three separate dyes in suppressing electron recombination, essentially demonstrating the importance of the dye in the system.^474^ The ssDSCs with D35 dye reached a PCE of 4.34%. D21L6, the second organic dye, performed somewhat worse, with a PCE of 3.05%. However, Zhang *et al.* demonstrated that the dye DPP07 is as efficient as LEG4 when combined with PEDOT, fabricating a device with a *V*_OC_ of 770 mV, *J*_SC_ of 11.13 mA cm^−2^, FF of 65% and PCE of 5.54%.^475^

Wang *et al.* investigated the properties of a pre-polymerized block copolymer of poly(3-hexylthiophene) and poly(2,5-dihexy-*p*-phenylene), and found that a PPP-*b*-P3HT-based solar cell achieved a *V*_OC_ of 810 mV, *J*_SC_ of 8.81 mA cm^−2^, FF of 65% and PCE of 4.65%.^476^

Liu *et al.* investigated the performance of P3HT with two different dyes. When sensitised with BzTCA, solar cells achieved a *V*_OC_ of 880 mV, *J*_SC_ of 8.22 mA cm^−2^, FF of 44% and PCE of 3.21%, demonstrating that organic dyes are better suited to operate with polymeric HTMs.^477^ Clément addressed P3HT's usual pore filling problems by creating a highly regioregular polymer with a medium molecular weight and limited dispersion.^478^ When P3HT with these properties was used in a system with a 2 μm thick TiO_2_ film, performance improved. Optimized devices had a *V*_OC_ of 720 mV, *J*_SC_ of 11.37 mA cm^−2^, FF of 58% and PCE of 4.78% after HTM deposition and an annealing step at 150 °C to enhance film morphology. In contrast, a device made using spiro-OMeTAD had a PCE of only 3.99%.

##### 4.3.2.3 Inorganic hole transporting materials

Organic HTMs are less stable in water and oxygen than inorganic materials. Generally, inorganic HTMs possess good electronic properties, good conductivity and high temperature stability.^479–481^ Although these inorganic HTMs already provide good stability in photovoltaic devices, their promise of efficiency remains unfulfilled. Table 7 lists device parameters of DSCs employing various inorganic HTMs referenced in this review, together with the dye used.

**Table 7:** Photovoltaic characteristics of DSCs implementing inorganic and metal complexes-based hole transporting materials

HTM	Sensitizer	*V*_OC_ (mV)	*J*_SC_ (mA cm^−2^)	FF (%)	PCE (%)	Year	Ref.
CsSnI_3_	N719	732	19.2	72.7	10.2	2012	21
Cs_2_SnI_6_	Z907	571	13.2	61.3	4.63	2014	482
Cs_2_SnI_6_	N719	631	14.7	68.1	6.32	2014	482
Cs_2_SnI_6_	Mix	623	16.9	66.1	6.94	2014	482
Cs_2_SnI_6_	Mix + PC^a^	618	18.6	68.0	7.80	2014	482
CuI	N3	739	14.5	69	7.40	2012	483
CuSCN	N719	578	10.52	55.6	3.39	2012	484
Cu(dmp)_2_	LEG4	1010	13.8	59	8.2	2015	485
Cu(tmby)_2_	Y123	1080	13.87	73.3	11.0	2017	486
Cu(tmby)_2_	WS-72	1070	13.8	79	11.7	2018	273
Cu(tmby)_2_	XY1:L1	1020	14.5	72	10.7	2020	26
Co(bpyPY4)	Y123	768	12.12	62	5.68	2016	394
Co(bpy)_3_	Y123	877	0.66	73	0.21	2016	394

a PC: photonic crystals.

Chung *et al.* used the tin-based perovskite compound CsSnI_3_ in a N719-sensitized ssDSC.^21^ With tin fluoride doped into semiconductors, the solar cell developed *V*_OC_ of 732 mV, *J*_SC_ of 19.2 mA cm^−2^, FFs of 72%, and a PCE of 10.2%. To circumvent the volatility of Sn(ii)-based perovskites, the Sn(IV)-compound Cs_2_SnI_6_ was implemented as hole transport material in solar cells, enabling to harvest holes from different photoanodes with different dyes.^482^ The PCE of the ssDSC sensitised with Z907 was 4.63%, whereas the PCE of the ssDSC sensitised with N719 was 6.32%. The highest results were obtained using a dye combination of N719, YD2-o-c8, and RLC5. This last system had a *V*_OC_ of 623 mV, a *J*_SC_ of 16.9 mA cm^−2^, a FF of 66%, and a PCE of 6.94%. The output with these dyes was increased even more after including photonic ZnO crystals in the device, reaching a *V*_OC_ of 618 mV, *J*_SC_ of 18.6 mA cm^−2^, FF of 68% with an overall PCE of 7.80% and showing stable output for over 800 hours.

Sakamoto *et al.* worked on copper iodide, a well-known HTM in the solar cell field. Their analysis discovered how the interface materials affect the formation of CuI layers. The degree of thiocyanate groups in both the dye and counter electrode was crucial for obtaining high efficiency. The variance of the SCN groups in the PEDOT:PSS-based counter electrode resulted in the systems having a greater than two-fold performance compared to those without SCN groups. The successful DSCs showed a *V*_OC_ of 739 mV, *J*_SC_ of 14.5 mA cm^−2^, FF of 69% and PCE of 7.4%.^483^

Out of the several p-type semiconductors examined for use as hole conductors, the chemical robustness of CuSCN is of particular interest owing to it being a polymeric semiconductor. The solar cells fabricated by Premalal *et al.* with this HTM included doped p-type copper sulphide nanoparticles and were coated onto a transparent conducting oxide base.^484^ Triethylamine hydrothiocyanate was used to dope CuSCN and obtain better conductivity; the resulting ssDSC reached a *V*_OC_ of 578 mV, *J*_SC_ of 10.52 mA cm^−2^, FF of 55% and PCE of 3.4%.

##### 4.3.2.4 Metal coordination complex hole transporting materials

Transition metal coordination complexes are a category of materials that incorporates the advantages and disadvantages of both organic and inorganic compounds. As organic compounds they retain an ease-of-processing, but with the high conductivities typical of inorganic compounds, which eliminate the need of p-dopants. The p-dopant is found in the compound itself, and it consists of a complex of the same metal with a higher oxidation state. Energy levels can be varied by modifying the ligand or metal center.^485,487–489^ Although liquid DSCs have greatly benefited from the implementation of transition metal complexes as electrolytes, as they are far more efficient and less corrosive than iodide/triiodide, only a handful of new compounds of this class have been tested in solid state DSCs so far.^284,286,320^ Despite this, the best-performing ssDSCs are those employing a metal complex as the hole conductor (see Table 7).

The first researchers to report on ssDSCs based on a metal complex hole conductor were Freitag *et al* with a phenanthroline-based copper complex with a phenanthroline-based copper complex ([Cu^II/I^(dmp)_2_]).^485^ Here, mixed oxidation states of the complex were introduced as solid-state hole transport material. The cell manufacturing technique was identical to the liquid cell construction, but the solvent was allowed to evaporate in air and a fresh injection was repeated until the air gap was filled with solid HTM. They were able to produce a *V*_OC_ of 1.01 V, *J*_SC_ of 13.8 mA cm^−2^, FF of 59% and PCE of 8.2%, surpassing the output of a spiro-OMeTAD-based reference device (5.6%) as well as that of a liquid junction DSC (6.0%).

Further improvements were made by the work of Grätzel and colleagues. Using the copper bipyridyl complex Cu(tmby)_2_ with the Y123 dye, the authors achieved a *V*_OC_ of 1080 mV, *J*_SC_ of 13.87 mA cm^−2^, FF of 73% and PCE of 11.0%.^486^

In later research, they developed a new dye for solar cells – WS-72 – able to reduce electron recombination and enhance their efficiency. A solid-state DSC with such dye and Cu(tmby)_2_ reached a *V*_OC_ of 1070 mV, *J*_SC_ of 13.8 mA cm^−2^, FF of 79% and PCE of 11.7%.^273^ Most recently, Michaels *et al.* established a new co-sensitization method using organic dyes XY1 and L1 sensitised solar cells, reporting the first numbers for indoor light conversion with solid-state DSCs of 30% at 1000 lx from a fluorescent lamp (10.7% in full sun).^26^

Kashif *et al.* employed a Co(iii/ii) coordination complex based on a polypyridyl hexadentate ligand: ([Co(bpyPY_4_)](OTf)_2.33_) and instead of slow solvent evaporation, the HTM solvent was extracted using vacuum.^394^ Kashif's top device reached a *V*_OC_ of 768 mV, *J*_SC_ of 12.12 mA cm^−2^, FF of 62% and PCE of 5.68%. For comparison, ssDSCs fabricated with the Co(bpy)_3_ metal complex, which usually yields excellent efficiencies in liquid DSCs,^366^ gave an output PCE of only 0.21% because of poor conductivity of the resulting HTM layer with this complex. This demonstrates that only certain metal complexes can be used as hole conductors in ssDSCs.

#### 4.3.3 Dopants and additives

Adding suitable chemical species to the electrolyte to fine-tune the semiconductor–electrolyte interface is the simplest way to increase photovoltaic performance. For the desired Fermi level upshift, nitrogen-heterocyclic compounds such as 4-*tert*-butylpyridine (*t*BP) and *N*-methylbenzimidazole (NMBI) are typically used to inhibit electron recombination and thus to improve the *V*_OC_.^490,491^ Consequently, as a regular additive, *t*BP is present in almost every electrolyte solution for liquid-junction DSCs. With iodine- and cobalt complexes-based electrolytes, *t*BP addition does not greatly affect ionic diffusion in solution, while in case of other coordination complex redox mediators it can have a negative effect. Saygili and co-workers introduced new bases – 2,6-bis-*tert*-butylpyridine (B*t*BP), 4-methoxypyridine (MOP) and 4-(5-nonyl)pyridine (NOP) – to copper-based redox mediator [Cu(tmby)_2_]^2+/+^, with significant effects on electrolyte properties.^492^ Guanidinium thiocyanate (GuSCN) has been found to increase both *V*_OC_ and *J*_SC_, as it accumulates its positive charge on the semiconductor surface, causing a positive conduction band edge shift, thus improving the efficiency of electron injection and at the same time slowing down recombination under open-circuit conditions. Another strategy was demonstrated by Boschloo and co-workers. They added a triphenylamine-based electron donor to a cobalt-based electrolyte and found a significantly improved performance as the oxidised dye molecules were regenerated extremely quickly, on the scale of sub-ns.^322,493,494^ The TPAA additive significantly suppressed the recombination of electrons in both TiO_2_ and oxidized dye molecules, indicating that there was a significant amount of recombination without it. In principle, such a sacrificial donor in the DSC electrolyte could give very high apparent PCE from *J–V* analysis. Moreover, MPP tracking during 250 h under 1 sun illumination demonstrated that every donor molecule had been cycled 3 × 10^5^ times without any apparent degradation.^322^

Additives in solid-state electrolytes and hole transport materials are added to the precursor solution prior to deposition in devices. Some, such as LiTFSI and *t*BP, are used to alter TiO_2_ energy levels and passivate its surface as they migrate towards it, allowing for improved charge injection and reduced recombination processes at the TiO_2_/HTM interface.^495–501^ In the solid state, they may have the added effect of changing the HTM film morphology. Additionally, certain dopants can directly influence the material. Studies demonstrate that the partial oxidation of the hole conducting substrate leads to increased hole mobility across the layer and, ultimately, conductivity. Oxidizing dopants are necessary for organic compounds and small molecules in particular (see Table 8 for differences in efficiency of DSCs with pristine and doped HTMs), and as an example they must be applied to the spiro-OMeTAD molecule to make it the ideal reference material for ssDSCs.^472,502,503^ Cappel *et al.* studied the p-doping properties of LiTFSI in the presence of light and air or N_2_ atmosphere and Snaith and co-workers continued the work providing a complete description of the doping properties of LiTFSI.^461,504^ Combined study results showed that oxidation of spiro-OMeTAD by molecular oxygen is activated by LiTFSI regardless of light exposure, while the latter alone is not capable of oxidation. This oxidation process in air has a detrimental side effect, as the redox process consumes Li^+^ ions, which also serve as additive on the titania surface.

**Table 8:** Photovoltaic efficiencies of DSCs with pristine and doped hole transporting materials

Dopant	Pristine efficiency (%)	Doped efficiency (%)	Year	Ref.
LiTFSI + O_2_	0	3	2013	461
FK102	2.3	5.6	2011	454
FK209	2.3	6.0	2013	505
FK269	2.3	6.0	2013	505
F4TCNQ	4.55	5.44	2012	506
SnCl_4_	2.52	3.40	2013	507
Spiro(TFSI)_2_	2.34	4.89	2014	460
TeCA	5.8	7.7	2015	508
TEMPO-Br	3.99	6.83	2018	509
DDQ	3.50	6.37	2018	510

**Fig. 39:**
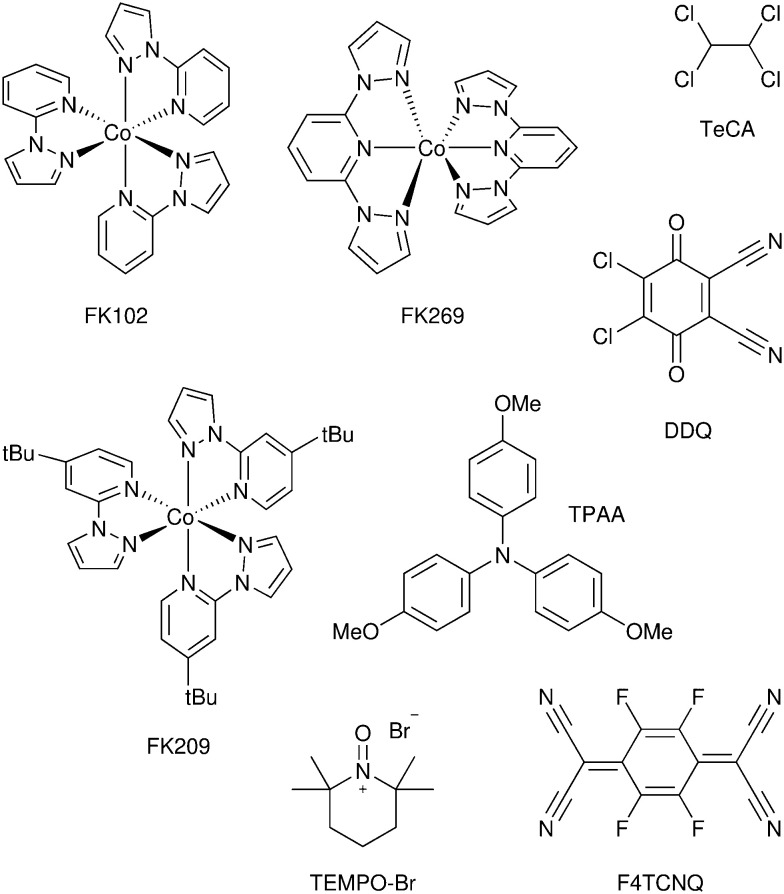
Examples of dopants for hole transporting materials.

A Co(iii) complex (FK102) has been used as oxidizing dopant in solar cells, which allowed them to attain relatively high efficiencies (Fig. 39).^454^ The complex oxidized spiro-OMeTAD in solution and the resulting Co(ii) species exhibited a low molar extinction coefficient. Upon doping the film's conductivity rose from 4.4 × 10^−5^ to 5.3 × 10^−4^ S cm^−1^, which boosted the overall performance from 2.3 to 5.6%. Two years later Burschka *et al.* proposed two new Co complexes with better performance, FK209 and FK269.^505^

Chen *et al.* oxidized spiro-OMeTAD with the Lewis acid 2,3,5,6-tetrafluoro-7,7,8,8-tetracyanoquinodimethane (F4TCNQ). The use of a dopant produced the spiro-OMeTAD^+^ species, which was confirmed by a UV-Vis measurement. They used pristine and doped HTMs in ssDSCs, resulting in an increase in efficiency from 0.01 to 0.33%.^506^ HTM layers with added lithium salt gave efficiencies of 4.55 and 5.44% with and without the presence of F4TCNQ, respectively. Han and colleagues studied a second Lewis acid, SnCl4, which increased conductivity fourfold. The efficiency was 3.4% with a 0.8% doping level of spiro-OMeTAD.^507^ McGehee and co-workers oxidized the hole conductor itself through the reaction of AgTFSI with Spiro-OMeTAD, removing the need of a p-dopant. Devices built with the pre-oxidized hole conductor demonstrated a significant efficiency increase from 0 to 4.67%.^460^

Xu *et al.* reported on 1,1,2,2-tetrachloroethane (TeCA), which they described as a co-solvent. The reason for this is that it is important to keep the TeCA-containing solution under UV light for one minute to allow the spiro-OMeTAD oxidation to take place. System efficiencies increased from 5.8% to 7.7%; for comparison, devices fabricated with FK209 yielded only 6.8% performance.^508^ TEMPO, previously reviewed among the redox mediators, has also been used as a dopant. Yang *et al.* reached solar cell efficiency of 6.83% by employing the bromide salt of the oxidised TEMPO.^509^ A recent study, published by Sun and colleagues, highlighted the effect of 2,3-dichloro-5,6-dicyano-1,4-benzoquinone (DDQ), an oxidant commonly used in chemical synthesis, on ssDSCs. Photovoltaic efficiencies improved from 3.50 to 6.37% when an small quantity of the dopant was introduced.^510^

### 4.4 Counter electrodes

The counter electrode (CE) has a major impact on the overall efficiency of DSCs and it performs two main functions: it receives electrons from the external circuit and transmits them into the cell – which necessitates a low resistance – and it acts as a catalyst for the reduction of the oxidized species of the redox mediator. A good CE for DSCs should have the following qualities: high catalytic activity towards the redox mediator, high conductivity, high reflectance, low cost, high surface area, high porosity, low charge-transfer resistance, high exchange current density, chemical resistance to corrosion, energy alignment meeting the potential of the electrolyte's redox couple and good processability for deposition.^511,512^ For DSCs, a great variety of CE preparation recipes has been demonstrated, including thermal and photo-decomposition,^513–516^ electrochemical deposition,^517–519^ chemical vapor deposition,^520^ and sputter deposition.^521–523^ The preparation methods greatly affect particle size, surface, morphology, and catalytic and electrochemical characteristics of the electrodes. Smaller particles and larger electrode surface areas provide more catalytic active sites and facilitate improved electrode operation.^524^

Platinum has traditionally been the most common counter electrode active material for DSCs, due to its excellent conductivity and catalytic activity, with PCEs over 12%.^284^ Nevertheless, Pt still has certain drawbacks to solve, including the high price and rarity of the raw material, poor stability over longer periods, as well as migration towards the photoanode and deposition on the TiO_2_ layer leading to cell shortage.^525–529^ Furthermore, due to energy level misalignment, Pt is not very effective in regenerating alternative redox couples such as coordination complexes, T_2_/T^−^ or polysulfide electrolytes.^339^ Fortunately, many other materials can be used as CE in DSCs.

**Fig. 40:**
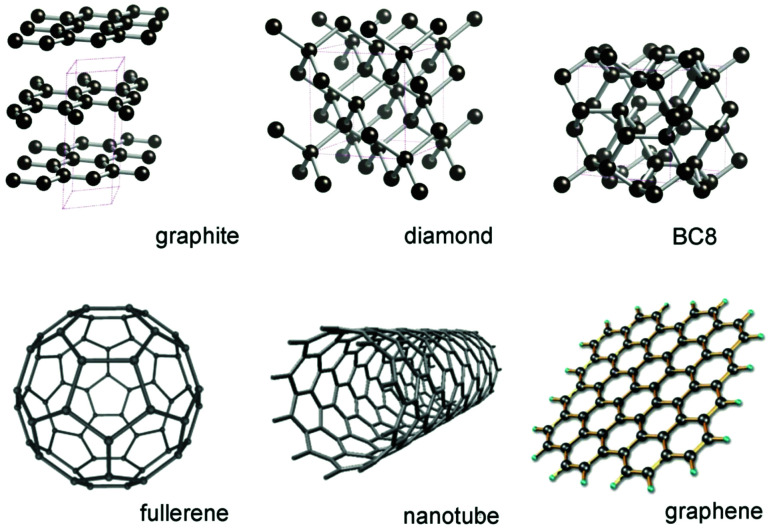
Structures of various carbon allotropes. Reprinted with permission from ref. 530. Copyright 2013 Mineralogical Society of America.

Carbon-based materials (Fig. 40)^530^ are attractive candidates to replace platinum as the CE material in DSCs thanks to advantages such as low cost, abundance, high surface area, high catalytic activity, high electrical conductivity, high thermal stability, corrosion resistance, and high reactivity for redox mediator reduction,^388^ among other characteristics. An FTO/Au/GNP (graphene nanoplatelets) stack was used as CE to reach a PCE of 14.3%.^24,531^ The inexpensive and easy preparation, and good stability improve the competitiveness of carbon materials. The key downsides of common CEs based on carbon compounds are an overall worse performance compared to platinized electrodes – in terms of conductivity and catalytic activity – when coupled with the I^−^/I_3_^−^ redox couple. Further, poor adhesion to the FTO substrate leads to electrode degradation.^532^ To mitigate these issues, in recent years researchers from Korea University have doped graphene nanoplatelets with various metals and halogens (Se, Te, Sb, F, I) to improve compatibility of carbon CEs towards the I^−^/I_3_^−^ redox couple. These electrodes proved more efficient than those based on Pt, and were also more stable.^533–536^

**Fig. 41:**
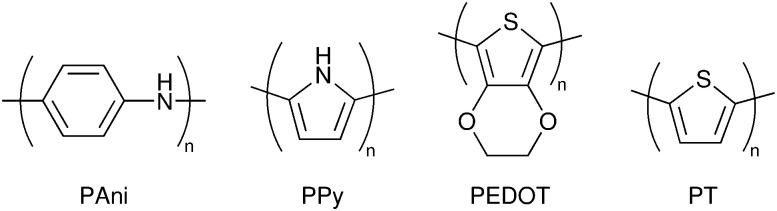
Repeating units of polymers used as counter electrode materials in DSCs.

Flexibility, translucency, and facile processing and tuning are all properties of conductive polymers that make them prime candidates as CE materials in DSCs (Fig. 41).^173,537^ PEDOT (poly(3,4-ethyleneedioxythiophene)), first discovered by the Bayer Lab in the 1980s, is a promising substrate for antistatic and opto-electronic applications due to its high conductivity, outstanding visible light transmittance and extraordinary stability.^517^ Although PEDOT is an insoluble polymer, it can be easily electrodeposited from its monomer in solution, resulting in excellent conductivity, much higher than that of polyaniline (PAni), polypyrrole (PPy) and polythiophene (PT).^537–539^ Moreover, the solution to PEDOT's insolubility problem was later solved by co-polymerization with poly(styrene sulfonate) (PSS). PEDOT:PSS is the market pioneer in transparent conductive polymers, it is water-soluble and allows fast manufacturing. Saito *et al.* investigated for the first time in 2002 PEDOT-based materials – specifically PEDOT:PSS and *p*-toluenesulfonate (TsO)-doped PEDOT – as CE for DSCs, deposited on FTO *via* chemical polymerization.^540^ The PCE of the cell with the PEDOT:TsO CE was almost the same as that with the Pt CE, while in the case of the PEDOT:PSS electrode it was shown that I^−^/I_3_^−^ oxidation/reduction processes occurred at higher potentials compared to the other two electrodes, which was attributed to a steric hindrance effect of the PSS component of the polymer.^540^ By using electrodeposition techniques, PEDOT is now being deployed in the most efficient DSCs, especially due to its high performance in combination with alternative redox mediators and hole transport materials. Tsao *et al.* showed how electropolymerized PEDOT CEs are much better performing with Co-based redox mediators compared to their Pt counterparts.^541^ Their best PEDOT-based cell reached a PCE of 10.3%, compared to 7.9% of a Pt-based one. The performance improvement was attributed to a much lower charge transfer resistance of PEDOT towards the Co complex compared to Pt. Freitag *et al.* achieved a PCE of 11.3% with a copper-mediated DSC featuring a PEDOT CE,^348^ recently surpassed by Grätzel *et al.* with a 13.5% PCE cell.^12^ One more advantage of PEDOT over Pt is that the former is a hole-selecting material. As such, it is possible to fabricate PEDOT-based sandwich-type solar cells without any spacing between the two electrodes without the risk of cell shortage.^320,348^

DSCs incorporating hybrid/mixed CEs outperform devices with single component CEs, thanks to the synergistic effects of the hybrid composite. However, the exact mechanism behind this success is still not fully understood on a fundamental level. Examples of efficient hybrid CEs include platinized PEDOT and a combination of graphene with PEDOT, PAni or Pt.^515,519,525,542,543^

## 5 P-type DSCs

### 5.1 Photocathodes

**Fig. 42:**
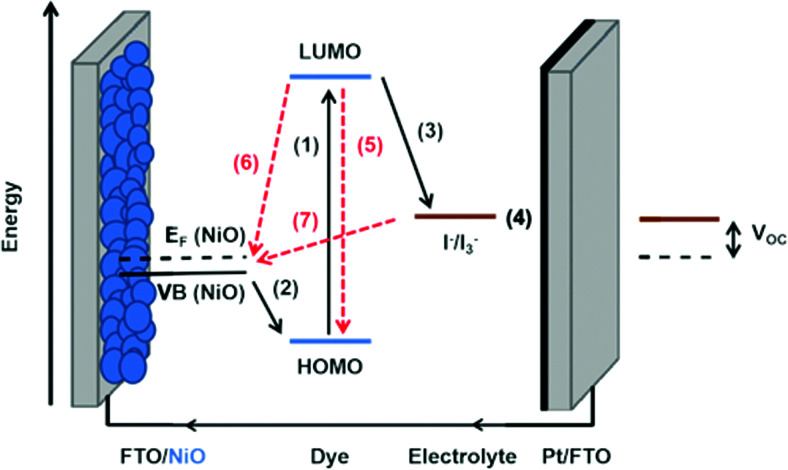
Schematic representation of the charge transfer processes occurring within a NiO-based p-DSC. Recombination processes shown in red. Processes 1–6 defined in the text. Adapted from ref. 544 with permission from The Royal Society of Chemistry, copyright 2019.

To increase the efficiency of dye-sensitized solar cells, it has been proposed that the TiO_2_-based photoanode could be combined in series with a second photoelectrode (*i.e.* a photocathode) in a tandem device.^544,545^ In a p–n tandem DSC, the light transmitted by the first photoelectrode can be captured by the second photoelectrode, extending the spectral response to the near IR. The *V*_OC_ becomes the sum of the two individual (n-type and p-type) devices. Therefore, there is an opportunity to collect more light more efficiently. In principle, tandem DSC devices (p–n DSCs) should overcome the thermodynamic limits of single-junction devices and achieve efficiencies above the Shockley–Queisser limit (theoretically up to 43%).^546–548^ Unfortunately, the efficiency of p-type DSCs is much lower than that of n-type DSCs, which limits the efficiency of p–n DSCs. For this reason, there has been increasing attention paid to the development of more efficient p-type DSCs in the last 20 years. In these devices the majority charge carriers in the semiconductor are positive holes (h^+^) and the current flows in the opposite direction to TiO_2_-based DSCs. Following excitation of the dye with light, electron transfer takes place from the valence band of the semiconductor to reduce the dye, as shown in Fig. 42.

Lindquist and coworkers reported the first p-DSC in 1999,^549^ which used a layer of NiO – a p-type semiconductor – instead of TiO_2_, and erythrosin B as the photosensitizer. This device had an overall PCE of 0.0076%. By 2010, this had been improved to 0.41% efficiency by improving the quality of the NiO and engineering a dye specifically for NiO.^550^ However, since the p-DSC efficiency was well below that of n-type devices, the tandem cell efficiency was severely limited (1.91%). Key limitations to the efficiency of p-DSCs include the rapid charge recombination at the dye/NiO and NiO/electrolyte interfaces. Developing photosensitizers that promote charge separation, together with new iodide-free redox mediators can lead to substantial improvements in device efficiencies. Further research into the mechanism, electron transfer dynamics and surface characterisation has enabled further improvements to be made over the following decade, which are summarised in the following sections. By the end of 2020 the highest tandem cell efficiency had reached 4.1%.^548^

### 5.2 Semiconductors

NiO is typically chosen as the p-type semiconductor, since it is straightforward to prepare, it has a high-lying valence band edge (0.3 V *vs.* SCE or 0.54 V *vs.* NHE at pH 7) and a wide bandgap (3.6–4.0 eV).^551–553^ There have been extensive articles and reviews on the various synthetic techniques and the challenges of applying NiO in p-DSCs.^554–559^ A comparison by Gibson *et al.* found that, based on the P1 dye, the best performance for mesoporous NiO electrodes was reached with a 1–2 μm thick film, with a crystallite size of *ca.* 20 nm and a specific surface area above 40 m^2^ g^−1^.^560^ The most commonly used synthetic technique is the sol–gel method, due to its simplicity and reproducibility, and pluronic triblock copolymer-templated NiO films satisfy these criteria, giving thicknesses of 1–2 μm and crystal sizes of 15–20 nm.^561^ Typically, these films are applied in the laboratory by the doctor blade technique, but Jousselme *et al.* attained promising results (*J*_SC_ = 3.42 mA cm^−2^) by inkjet printing a sol–gel precursor.^562^

Despite being straightforward to synthesize and deposit, there are several unfavourable characteristics of NiO. Firstly, whereas TiO_2_ is non-toxic, NiO is a group 1 carcinogen. The *V*_OC_ of NiO-based DSCs is limited to 100–200 mV because NiO has a high-lying valence band (0.54 V *vs.* NHE), which is advantageous in terms of electron transfer to photosensitizers, but leads to a small difference between the Fermi level in the NiO and the redox potential of the electrolyte. NiO also has a low charge diffusion coefficient (∼10^−8^ cm^2^ s^−1^)^561,563,564^ and the presence of high valence states (*e.g.* Ni^III^ and Ni^IV^) leads to rapid recombination at the dye/semiconductor and semiconductor/electrolyte interfaces.^565,566^ This leads to a small diffusion length for holes (2–3 μm), which means thin NiO films must be used.^566^ Strategies applied over the last 10 years to reduce recombination include applying compact blocking layers on the FTO substrate,^567^ chemical reduction of the NiO surface,^568^ surface treatment with an aqueous nickel salt,^569^ applying a thin, surface layer of Al_2_O_3_, B or TiO_2_,^570–572^ or adding organic surfactants such as chenodeoxycholic acid.^573^ Other approaches to improving the electronic properties (either by increasing the hole mobility or lowering the Fermi level) include doping or forming solid solutions with alkali or transition metals such as Li, Co, Mg.^574–577^ However, a competition between increasing *V*_OC_ and decreasing *J*_SC_ is frequently observed, possibly as a result of decreasing the driving force for electron injection if the valence band edge is shifted to more positive potential. The porosity, dye loading and hole transport can be improved by adding graphene or reduced graphene oxide to NiO.^578,579^ However, despite these modest improvements, the small built-in potential and poor fill factors (typically 30–40%) limit the solar cell efficiency to <1%.

Increasing the solar cell efficiency requires finding a replacement for NiO, ideally with a *ca.* 0.5 V deeper-lying valence band to match the *V*_OC_ of TiO_2_. This is difficult due to the trade-off between conductivity and transparency. Binary or ternary nickel oxides and oxysulfides have been tested in p-type DSCs, but in each case, if the *V*_OC_ was improved, the current was sacrificed. The potential reasons for this could be physical (insufficient surface area for the dye to adsorb or insufficient porosity for the electrolyte to diffuse), electronic (low dielectric constant or hole mobility) or surface properties such as the presence of high-valence Ni.

K-Doped ZnO thin films, which have high optical transparency (>85%) and a larger hole diffusion coefficient (10^−6^ cm^2^ s^−1^) than NiO, show some promise for p-DSCs (*J*_SC_ = 0.408 mA cm^−2^, *V*_OC_ = 82 mV, and PCE = 0.0012% with C343).^580^ More encouraging results have been achieved with tin-doped indium oxide (ITO) reaching PCEs of *ca.* 2%.^581,582^ Promising results have been obtained with CuO-based DSCs by applying nanoparticles, nanorods or nanowires.^583^ One-dimensional materials could overcome the shorter transport lifetime for holes in CuO compared to NiO. CuO electrodes are unstable towards I^−^/I_3_^−^ , so alternative redox mediators such as cobalt coordination complexes are required.^584^ An efficiency of 0.19% was reached in combination with zinc phthalocyanine sensitizers and cobalt-based redox mediators.^585–587^ However, CuO is not optically transparent (*E*_g_ = 1.4 eV^584^). Cu_2_O is more transparent but less stable than CuO. With C343, a Cu_2_O device gave a *V*_OC_ = 0.71 V, a *J*_SC_ = 1.3 mA cm^−2^, FF = 46%, and a PCE of 0.42%.^588^ Cu_2_O@CuO core–shell structures have been applied to improve the stability, but this has not yet improved the solar cell characteristics (*V*_OC_ = 315 mV, *J*_SC_ = 0.14 mA cm^−2^, PCE = 0.017%).^589^

Cu-Based delafossites (CuAlO_2_, CuGaO_2_, CuFeO_2_, CuBO_2_, CuCrO_2_ and CuCrO_2_) have been highlighted as potential p-type transparent conductive oxides.^590,591^ During the last 10 years, attempts have been made to exploit the deeper-lying valence band and high hole mobility of these materials compared to NiO in p-DSCs.^584,592–596^ Efficiencies of 0.04% have been recorded with CuAlO_2_, but with delafossites, as with doped NiO, a trade-off between *J*_SC_ (<1 mA cm^−2^) and *V*_OC_ (333 mV) has been found.^584,592,597^ Better efficiencies of up to 0.18% have been obtained with CuGaO_2_ in combination with P1 and I^−^/I_3_^−^.^598,599^ Doping with Mg, Fe and Al improves the specific surface area of CuGaO_2_ photocathodes and conversion efficiencies comparable with NiO have been reached with Mg:CuGaO_2_.^593,600,601^ The best results so far have been with CuCrO_2_, which reached 0.4% PCE with PMI-6T-TPA and the [Co(en)_3_]^2+/3+^ electrolyte, but although the *V*_OC_ (734 mV) was better than the equivalent NiO device, the *J*_SC_ (1.23 mA cm^−2^) was much lower.^602^ Successful attempts to improve the current include adding plasmonic Au nanoparicles,^603^ and doping with Mg, Ga and Co, but solar cell efficiencies with delafossites are yet to surpass NiO.^604–606^

Other proposed alternatives to NiO include mixed chalcogens. LaCuOS has been applied in p-DSCs with PMI-NDI dye but a low PCE (0.002%) was recorded, which the authors attribute to similar valence band edge energies of NiO and LaCuOS, rapid charge recombination and weak binding affinity for the dye on the surface.^607^ More encouraging results have been reported with spinel cobaltites (MCo_2_O_4_; M = Ni, Zn). A NiCo_2_O_4_ device with N719 reached a PCE = 0.785% (*V*_OC_ = 189 mV, *J*_SC_ = 8.35 mA cm^−2^, FF = 50%), which is exceptionally high compared to most other p-DSCs fabricated using the standard I^−^/I_3_^−^ electrolyte.^608,609^Table 9 lists the electrochemical properties of the referenced p-type semiconductors, together with the best cell efficiency obtained with them.

**Table 9:** Properties and characteristics of p-type metal oxides

Semiconductor	Bandgap (eV)	Valence band energy (eV *vs.* vacuum)	Dielectric constant	Max cell efficiency (%) – electrolyte used	Ref.
NiO	4.7–4	−4.94 to −4.7	9.7	2.51 – Fe(acac)_3_	103,555,571,573,610
K:ZnO	3.23	−5.7	Not reported	0.012 – I^−^/I_3_^−^	580
Sn:In_2_O_5_ (ITO)	4.1	−4.8	Not reported	1.96 – Fe(acac)_3_	582,611
CuO	1.41–1.82	−4.95 to −5.09	18.1	0.19 – I^−^/I_3_^−^	584,587,612
Cu_2_O	2.4	−5.20	12	0.42 – I^−^/I_3_^−^	588,613–615
CuAlO_2_	3.5	−5.68	10	0.037 – I^−^/I_3_^−^	590–592,616
CuCrO_2_	3.11	−5.44	Not reported	0.48 – Co(en)_3_	602,603,606
Au@SiO_2_:CuCrO_2_	3.11	Not reported	Not reported	0.31 – T^−^/T_2_	603
Mg:CuCrO_2_	Not reported	Not reported	Not reported	0.132 – I^−^/I_3_^−^	604
Ga:CuCrO_2_	3.25–3.30	−5.39	Not reported	0.100 – I^−^/I_3_^−^	606
AgCrO_2_	3.32	Not reported	Not reported	0.0145 – I^−^/I_3_^−^	595
CuGaO_2_	3.6–3.8	−5.29	0.96	0.182 – I^−^/I_3_^−^	598,599,617
CuFeO_2_	2.03–3.35	−4.9 to −5.13	Not reported	0.0103 – I^−^/I_3_^−^	597,618
LaOCuS	3.1	−4.94	4	0.002 – Co(dtb-bpy)_3_	607,619,620
NiCo_2_O_4_	2.06–3.63	−5.00	Not reported	0.785 – I^−^/I_3_^−^	608,621

### 5.3 Sensitizers

**Fig. 43:**
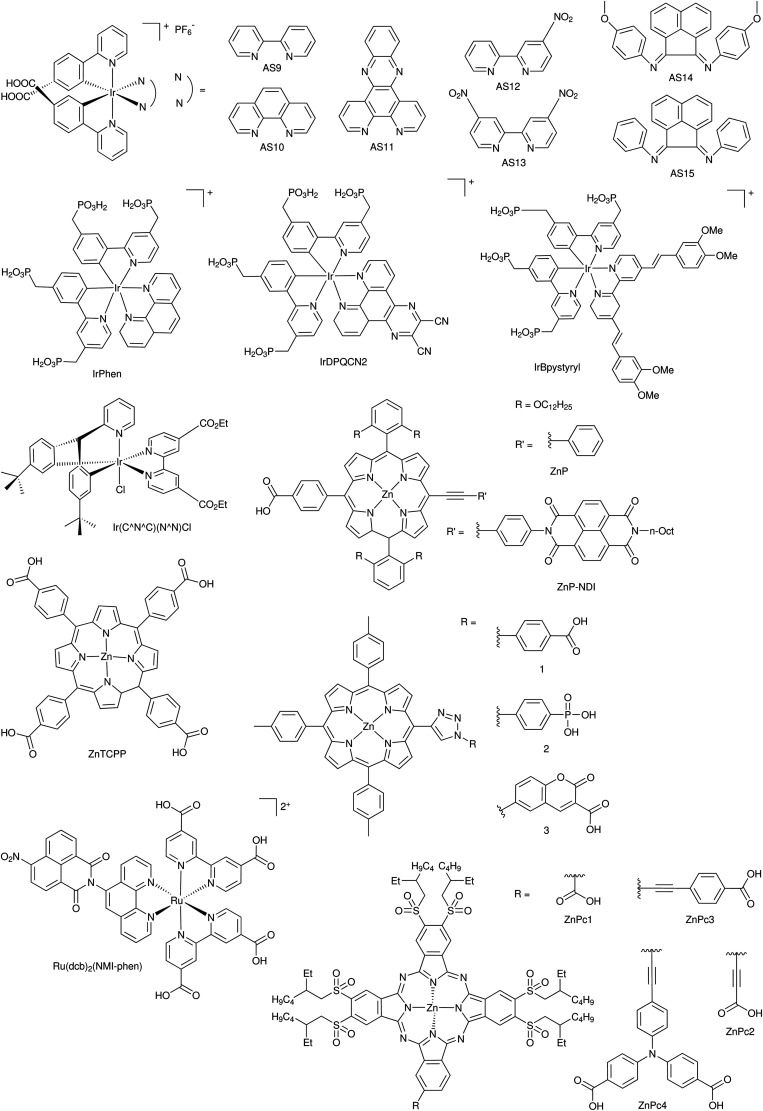
Examples of metal complex-based sensitizers for p-type DSCs.

In p-DSCs, the frontier orbitals of the dye must be arranged such that the HOMO lies at more positive potential than the valence band edge of the semiconductor, while the LUMO must be more negative than the redox potential of the electrolyte.^622–626^ Because the film thickness is limited by the diffusion length in NiO devices (see above), high extinction coefficients are required to capture all incident light. If the photocathode is to be positioned on the bottom of the cell, the dye needs to absorb red-NIR photons. In the first ten years of p-type DSC development commercial dyes were applied, but the first breakthroughs came from developing bespoke “push–pull” systems specifically designed for photocathodes.^550,564,627^ D–π–A systems, where the electron density is pushed away from the NiO surface on excitation of the dye, improve the charge-separated state lifetime and quantum efficiency. Over the last 10 years, a substantial number of different dye systems have been developed and tested in p-DSCs, typically with NiO.^544,610,628–630^ Metal complexes such as N719 and N3 generally give poor results in p-DSCs.^554^ There are a few examples of Ru-based dyes giving promising results with NiO, where there is some charge-transfer character directed away from the semiconductor surface (*e.g.* an anchoring group is positioned on the electron donating part of the molecule), see Table 10.^631–636^ Ir complexes (Fig. 43) have also been applied in p-DSCs due to their long lived and strongly oxidizing triplet excited states which favour hole injection into the semiconductor valence band.^637–640^ The *J*_SC_ of iridium photosensitizers is generally low due to the narrow absorption spectrum.

**Table 10:** Photovoltaic characteristics of p-type DSCs implementing metal coordination complexes-based sensitizers. IPCE values with the approximation sign are a visual estimate taken from the plotted data

Sensitizer	Electrolyte	*V*_OC_ (mV)	*J*_SC_ (mA cm^−2^)	FF (%)	PCE (%)	IPCE max (%)	Year	Ref.
K1	I_2_, LiI	96	2.91	32	0.09	14	2014	631
K2	I_2_, LiI	93	1.96	39	0.07	9	2014	631
O3	I_2_, LiI	93	3.04	35	0.099	∼20	2013	632
O13	I_2_, LiI	89	2.66	31	0.074	∼19	2013	632
O17	I_2_, LiI	92	2.69	34	0.085	∼16	2013	632
O8	I_2_, LiI	63	0.44	36	0.009	2.02	2012	633
O11	I_2_, LiI	79	1.16	36	0.033	5.49	2012	633
O12	I_2_, LiI	82	1.84	34	0.051	9.08	2012	633
O18	I_2_, LiI	93	3.43	33	0.10	Not reported	2014	636
SL1	I_2_, DMBII	104	2.25	34	0.079	18	2016	634
SL2	I_2_, DMBII	77	1.5	33	0.038	10	2016	634
[Ru(bpy)_2_(H1)	I_2_, LiI	95	4.06	36	0.14	Not reported	2017	635
IrPhen	Co(dtb-bpy)_3_	345	0.14	44	0.021	∼4	2014	637
IrDPQCN2	Co(dtb-bpy)_3_	508	0.25	54	0.068	∼6.2	2014	637
IrBpystyryl	Co(dtb-bpy)_3_	383	0.37	44	0.061	∼10.5	2014	637
1	I_2_, LiI	58	0.076	27	0.0012	2	2017	638
AS16	I_2_, LiI	94	0.69	42	0.028	17	2017	638
2	I_2_, LiI	134	0.069	40	0.0037	3	2017	638
AS17	I_2_, LiI	89	0.14	42	0.0052	5	2017	638
3	I_2_, LiI	77	0.16	45	0.0056	6	2017	638
AS18	I_2_, LiI	79	0.15	46	0.0055	6	2017	638
AS19	I_2_, LiI	104	0.45	42	0.02	∼28	2016	639
AS9	I_2_, LiI	90	0.68	36.6	0.022	∼15	2017	640
AS10	I_2_, LiI	90	0.66	37.6	0.022	∼21.5	2017	640
AS11	I_2_, LiI	70	0.45	38.1	0.013	∼11	2017	640
AS12	I_2_, LiI	90	0.36	40.1	0.013	∼13	2017	640
AS13	I_2_, LiI	100	0.82	38.7	0.032	∼26	2017	640
AS14	I_2_, LiI	100	1.12	36.8	0.043	∼21.5	2017	640

**Fig. 44:**
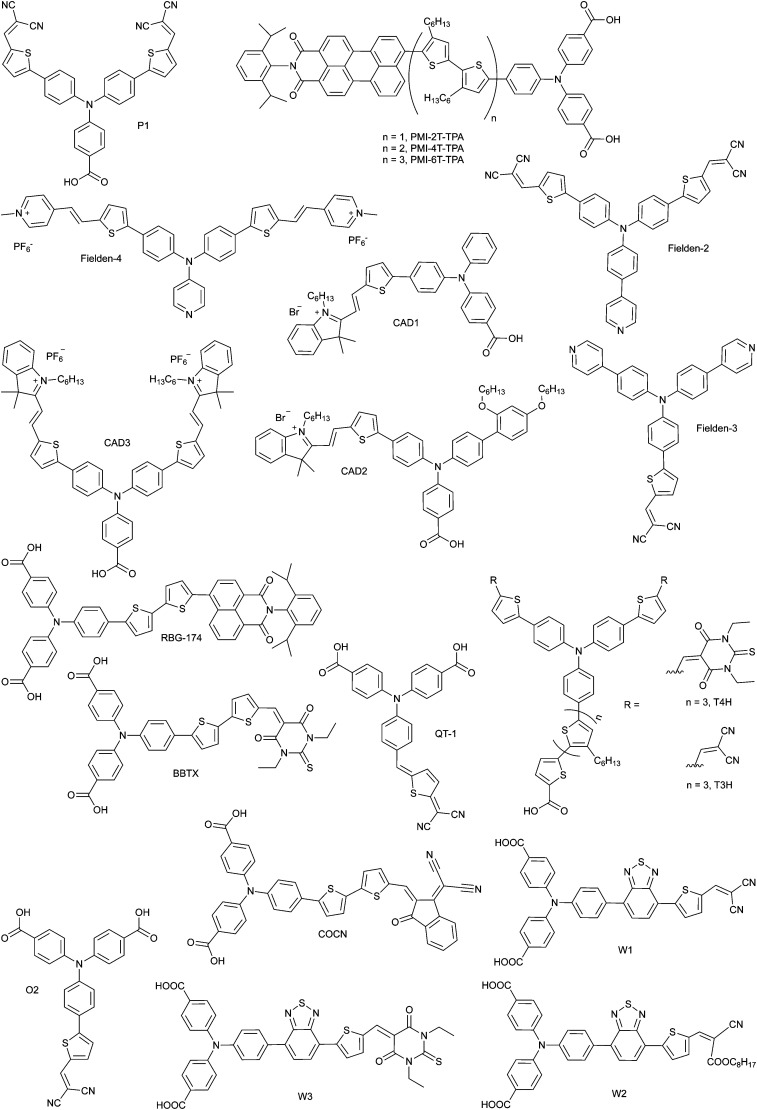
Examples of triphenylamine-based sensitizers for p-type DSCs.

Better results have been reported with metal-free systems (see Table 11). The push–pull dye P1 was one of the first organic dyes to achieve a reasonably high *J*_SC_. The design was based on the triphenylamine-based dyes used in n-type DSCs and many subsequent dyes for p-DSCs have since been based on this architecture.^563,641^ Optimised devices with P1 and I^−^/I_3_^−^ give IPCE = *ca.* 63%, and PCE of 0.16%, and P1 has become a benchmark dye for optimising new materials in p-DSCs.^550,564,642^ In the last decade since these breakthroughs, numerous arylamine-containing molecules have been designed for p-DSCs (Fig. 44), mostly with different acceptor or linker groups,^643–649^ and a few reports of modified anchoring structure.^626,650^ Dyes with two acceptor groups per triarylamine unit tend to have a higher absorption coefficient and produce a higher *J*_SC_. The highest *J*_SC_ reported for a p-DSC was produced using CAD3 with two cationic indolium groups as electron acceptors (*J*_SC_ = 8.21 mA cm^−2^, *λ*_max_ = 614 nm, *ε* = 95 000 M^−1^ cm^−1^).^643,651^

**Table 11:** Photovoltaic characteristics of p-type DSCs implementing organic sensitizers

Sensitizer	Electrolyte	*V*_OC_ (mV)	*J*_SC_ (mA cm^−2^)	FF (%)	PCE (%)	IPCE max (%)	Year	Ref.
1	I_2_, MBII	153	2.06	29	0.09	∼10	2010	550
2	I_2_, MBII	176	3.40	32	0.19	∼20	2010	550
3	I_2_, MBII	218	5.35	35	0.41	∼50	2010	550
P1	I_2_, LiI	89	5.37	33	0.16	54	2015	643
P1	Co(dtb-bpy)_3_	280	1.18	30	0.10	∼20	2016	652
C343	I_2_, LiI	208	0.951	32.4	0.064	7.1	2019	653
C343	Co(dtb-bpy)_3_	190	0.25	32	0.015	∼2	2009	627
PI	Co(dtb-bpy)_3_	80	0.26	26	0.006	∼3	2009	627
PINDI	Co(dtb-bpy)_3_	350	1.66	34	0.20	31	2009	627
Eosin B	I_2_, LiI	77	0.14	29	0.0032	Not reported	2008	554
Erythrosin J	I_2_, LiI	122	0.36	26	0.011	Not reported	2008	554
Rhodamine 101	I_2_, LiI	69	0.12	21	0.0022	Not reported	2008	554
Rhodamine 110	I_2_, LiI	80	0.15	25	0.0031	Not reported	2008	554
P4	I_2_, LiI	100	2.48	36	0.09	44	2009	641
P2	I_2_, LiI	63	3.37	31	0.07	32	2010	642
P3	I_2_, LiI	55	1.36	34	0.03	6	2010	642
P7	I_2_, LiI	80	3.37	35	0.09	26	2010	642
CAD3	I_2_, LiI	101	8.21	31	0.25	50	2015	643
GS1	I_2_, LiI	106	5.87	31	0.20	53	2015	643
QT-1	I_2_, LiI, DMII	120	8.2	34	0.33	60	2015	644
QT-1	Co(pz-py)_3_	226	6.5	34	0.50	Not reported	2015	644
zzx-op1	I_2_, LiI	96	5.70	38	0.21	50.1	2014	645
zzx-op1–2	I_2_, LiI	117	7.57	40	0.35	70.2	2014	645
zzx-op1–3	I_2_, LiI	115	6.68	40	0.31	∼57	2014	645
zzx-op2	I_2_, LiI	111	4.00	36	0.16	∼27	2014	646
zzx-op3	I_2_, LiI	109	3.80	36	0.15	∼20	2014	646
C1	I_2_, LiI	40	1.63	27	0.016	∼24	2017	647
C2	I_2_, LiI	59	2.41	29	0.040	∼22	2017	647
C3	I_2_, LiI	17	1.00	17	0.001	∼36	2017	647
SK2	I_2_, LiI	81	0.51	33	0.014	∼14	2016	648
SK3	I_2_, LiI	82	0.54	33	0.015	∼11.5	2016	648
SK4	I_2_, LiI	134	0.43	32	0.018	∼5.6	2016	648
RBG-174	I_2_, LiI	90	2.88	36.7	0.096	Not reported	2018	649
COCO	I_2_, LiI	91	2.45	35.9	0.080	Not reported	2018	649
BBTX	I_2_, LiI	88	4.32	33.0	0.126	Not reported	2018	649
COCN	I_2_, LiI	77	1.53	32.3	0.038	Not reported	2018	649
CW1	I_2_, LiI	93	3.54	35	0.114	∼36	2014	626
CW2	I_2_, LiI	118	4.05	34	0.160	∼42	2014	626
1	I_2_, LiI	50	0.83	43	0.018	∼25	2019	650
2	I_2_, LiI	103	1.6	36	0.060	∼25	2019	650
3	I_2_, LiI	49	0.87	32	0.014	∼22.5	2019	650
4	I_2_, LiI	66	0.83	33	0.018	∼25	2019	650
5	I_2_, LiI	86	1.11	37	0.036	∼25	2019	650
6	I_2_, LiI	70	0.84	23	0.014	∼21.3	2019	650
CAD1	I_2_, LiI	87	3.32	33	0.09	25	2014	651
CAD2	I_2_, LiI	96	3.25	33	0.10	17	2014	651
T3	I_2_, LiI	121	5.01	30.3	0.184	∼30	2015	654
T4	I_2_, LiI	119	5.31	32.9	0.208	∼32	2015	654
T5	I_2_, LiI	124	4.51	33.3	0.186	∼27	2015	654
T6	I_2_, LiI	133	4.02	33.3	0.178	∼23	2015	654
T3H	I_2_, LiI	133	5.56	30.5	0.226	∼32	2016	655
T4H	I_2_, LiI	152	6.74	31.0	0.317	∼38	2016	655
T1	I_2_, LiI	125	2.82	31	0.11	∼37	2014	656
T3	I_2_, LiI	144	4.01	33	0.19	∼45	2014	656
T4	I_2_, LiI	123	1.69	29	0.06	∼26	2014	656
BH_2_	I_2_, DMII	97	4.3	31	0.13	Not reported	2014	657
BH4	I_2_, DMII	128	7.4	30	0.28	Not reported	2014	657
BH6	I_2_, DMII	95	4.4	31	0.13	Not reported	2014	657
E1	Co(dtb-bpy)_3_	320	0.93	44	0.13	∼13	2016	652
E2	Co(dtb-bpy)_3_	320	0.78	41	0.10	∼9	2016	652
O2	I_2_, LiI	94	1.43	37	0.050	12.3	2011	658
O6	I_2_, LiI	97	1.04	37	0.037	13.5	2011	658
O7	I_2_, LiI	90	1.74	38	0.060	17.9	2011	658
QT-1	I_2_, LiI, DMII	120	8.2	34	0.33	60	2015	644
QT-1	Co(pz-py)_3_	226	6.5	34	0.50	Not reported	2015	644
EH122	I_2_, LiI, DMPII	134	4.39	30.3	0.178	∼28	2019	659
EH126	I_2_, LiI, DMPII	122	3.93	30.4	0.146	∼25.5	2019	659
EH166	I_2_, LiI, DMPII	131	3.47	28.4	0.129	∼20.5	2019	659
EH162	I_2_, LiI, DMPII	115	1.79	30.4	0.062	∼16	2019	659
EH174	I_2_, LiI, DMPII	137	4.84	31.2	0.207	∼28.5	2019	659
EH170	I_2_, LiI, DMPII	139	3.47	31.5	0.152	∼20	2019	659
BOD1	I_2_, LiI	70	0.56	38	0.015	Not reported	2020	660
BOD2	I_2_, LiI	40	0.48	29	0.006	Not reported	2020	660
BOD3	I_2_, LiI	60	0.21	29	0.003	Not reported	2020	660
1	I_2_, LiI	79	3.15	31	0.08	28	2014	661
Bodipy-CO_2_H	I_2_, LiI	95	1.48	36	0.05	20	2015	662
4	I_2_, LiI	97	1.60	38	0.06	27	2015	662
5	I_2_, LiI	109	3.70	35	0.14	44	2015	662
6	I_2_, LiI	95	1.58	35	0.05	23	2015	662
7	I_2_, LiI	106	5.87	31	0.20	53	2015	662
1	I_2_, LiI, BMII	79	0.61	25	0.012	3.2	2019	663
W1	I_2_, LiI	131	2.83	34.0	0.126	∼14	2015	664
W2	I_2_, LiI	121	4.16	33.0	0.166	∼17	2015	664
W3	I_2_, LiI	134	2.32	33.1	0.103	∼9	2015	664
1	I_2_, LiI	105	1.59	35.9	0.060	∼17	2011	665
2	I_2_, LiI	115	1.39	36.3	0.058	∼15	2011	665
3	I_2_, LiI	113	1.38	34.0	0.053	∼14	2011	665
4	I_2_, LiI	125	2.25	33.1	0.093	∼27.5	2011	665
5	I_2_, LiI	122	2.18	34.6	0.092	∼17	2011	665
6	I_2_, LiI	131	2.05	32.4	0.087	∼24	2011	665
S	I_2_, LiI	132	2.31	33.1	0.101	∼22.5	2011	665
p-SQ1	I_2_, LiI	117	1.22	37.1	0.053	∼6	2012	666
p-SQ2	I_2_, LiI	140	1.92	42.0	0.113	∼19	2012	666
BQI	I_2_, BMII	140	3.00	33	0.140	∼37	2017	571
BQII	I_2_, BMII	137	2.17	34	0.102	∼25	2017	571
I	I_2_, LiI	124	2.36	37	0.11	∼20	2013	667
II	I_2_, LiI	130	2.97	35	0.14	∼29	2013	667
PMI-CO_2_H	T^−^/T_2_	161	1.52	25.4	0.062	∼20	2020	668
PMI-HQ	T^−^/T_2_	164	2.21	23.8	0.086	∼21.5	2020	668
PMI-DPA	T^−^/T_2_	168	1.33	24.6	0.055	∼26	2020	668
PMI-acac	T^−^/T_2_	169	2.08	27.9	0.098	∼32	2020	668
PMI-PO_3_H_2_	T^−^/T_2_	181	1.27	17.7	0.041	∼20	2020	668
CAD4	I_2_, LiI	84	3.96	31.6	0.105	Not reported	2017	669
1	I_2_, LiI	41	0.31	31	0.004	10	2017	670
2	I_2_, LiI	53	0.53	30	0.009	5	2017	670
3	I_2_, LiI	61	1.17	32	0.023	11	2017	670
YK-1	I_2_, BMII	102	2.33	27.9	0.064	∼13	2018	671
YK-2	I_2_, BMII	93	1.95	29.5	0.054	∼11	2018	671
JW44	I_2_, LiI	75	1.29	31	0.030	∼21	2014	672
1	I_2_, LiI	57	0.28	35	0.006	5.4	2019	673
2	I_2_, LiI	74	0.45	35	0.012	8.2	2019	673
3	I_2_, LiI	76	0.51	37	0.014	9.8	2019	673
ZnP_ref_	I_2_, LiI	98	0.19	35	0.006	Not reported	2019	673
PP1	I_2_, LiI	132	1.45	36	0.069	10	2018	674
SQ	I_2_, LiI	85	1.18	34	0.034	∼24	2014	675
SQ	Co(dtb-bpy)_3_	85	0.12	30	0.0041	∼2	2014	675
PMI-NDI	I_2_, LiI	135	0.69	35	0.033	∼15	2014	675
PMI-NDI	Co(dtb-bpy)_3_	315	1.06	31	0.10	∼17	2014	675
SQ-PMI	I_2_, LiI	65	1.31	31	0.0026	∼24	2014	675
SQ-PMI	Co(dtb-bpy)_3_	95	0.34	28	0.009	∼4	2014	675
SQ-PMI-NDI	I_2_, LiI	95	2.73	32	0.083	∼25	2014	675
SQ-PMI-NDI	Co(dtb-bpy)_3_	175	1.17	27	0.055	∼22	2014	675
1	I_2_, LiI	100	1.89	33	0.063	∼26	2016	676
1	Co(dtb-bpy)_3_	198	0.49	24	0.024	∼11	2016	676
2	I_2_, LiI	84	1.44	33	0.040	∼23	2016	676
2	Co(dtb-bpy)_3_	134	0.41	24	0.013	∼7	2016	676
DPP-Br	I_2_, LiI	70	0.88	33	0.020	∼21	2016	676
DPP-Br	Co(dtb-bpy)_3_	103	0.26	28	0.007	∼5	2016	676
3	I_2_, LiI	90	2.03	33	0.062	∼35	2016	676
3	Co(dtb-bpy)_3_	330	2.06	30	0.205	∼26	2016	676
4	I_2_, LiI	76	1.72	32	0.041	∼24	2016	676
4	Co(dtb-bpy)_3_	370	1.95	29	0.21	∼25	2016	676
DPP-NDI	I_2_, LiI	81	1.79	34	0.048	∼30	2016	676
DPP-NDI	Co(dtb-bpy)_3_	292	1.56	29	0.13	∼28	2016	676
ISO-Br	I_2_, LiI	87	0.82	34	0.025	∼5	2015	677
ISO-Br	Co(dtb-bpy)_3_	182	0.80	23	0.033	∼8	2015	677
ISO-NDI	I_2_, LiI	96	1.27	33	0.040	∼7	2015	677
ISO-NDI	Co(dtb-bpy)_3_	260	1.54	25	0.100	∼13	2015	677
ZnP_ref_	I_2_, LiI, DMBII	98	0.19	35	0.006	Not reported	2016	678
ZnP-NDI	I_2_, LiI, DMBII	127	1.38	32	0.056	Not reported	2016	678
ZnP–TPA–NO_2_	I_2_, LiI, DMBII	107	0.29	38	0.012	Not reported	2016	678
TCPP	I_2_, LiI	128	0.8	39	0.04	Not reported	2014	679
ZnTCPP	I_2_, LiI	158	1.5	38	0.09	∼33	2014	679
ZnP–CO_2_H–NO_2_	I_2_, LiI, DMBII	113	0.49	36	0.020	∼16	2015	680
ZnP–eCO_2_H–NO_2_	I_2_, LiI, DMBII	114	0.48	35	0.019	∼16	2015	680
ZnP–CO_2_H–eNO_2_	I_2_, LiI, DMBII	98	0.43	32	0.013	∼14	2015	680
ZnP–eCO_2_H–eNO_2_	I_2_, LiI, DMBII	115	0.55	34	0.022	∼10	2015	680
ZnP–CO_2_H–eNDI	I_2_, LiI, DMBII	127	1.38	32	0.056	∼20	2015	680
ZnP–CO_2_H–eNDI	Co(dtb-bpy)_3_	195	0.5	31	0.03	Not reported	2015	680
ZnP–CO_2_H–BV^2+^	I_2_, LiI, DMBII	125	0.44	33	0.018	∼11.5	2015	680
3	I_2_, LiI	134	0.956	28.9	0.037	24.3	2019	653
3(Ni)	I_2_, LiI	206	1.199	33.2	0.082	26.0	2019	653
4	I_2_, LiI	195	1.353	33.0	0.087	23.0	2019	653
C_60_trZnPCOOH	I_2_, LiI	109	1.86	37	0.076	Not reported	2018	681
C_60_trZnPCOOH	Co(dtb-bpy)_3_	244	0.63	35	0.054	Not reported	2018	681
C_60_trZnPtrCOOH	I_2_, LiI	84	1.82	33	0.050	Not reported	2018	681
C_60_trZnPtrCOOH	Co(dtb-bpy)_3_	269	0.76	36	0.074	Not reported	2018	681
C_60_ZnPCOOH	I_2_, LiI	103	1.68	37	0.063	Not reported	2018	681
C_60_ZnPCOOH	Co(dtb-bpy)_3_	175	0.71	28	0.035	Not reported	2018	681
PhtrZnPCOOH	I_2_, LiI	68	0.69	33	0.015	Not reported	2018	681
PhtrZnPCOOH	Co(dtb-bpy)_3_	48	0.22	24	0.002	Not reported	2018	681
PMI-6T-TPA	Fe(acac)_3_	568	6.4	52	1.90	∼60	2018	682
ZnP0	Fe(acac)_3_	327	1.9	48	0.26	Not reported	2018	682
ZnP1	Fe(acac)_3_	465	4.4	45	0.92	∼43	2018	682
VG1-C8	Iodolyte Z-150	87	0.577	37.2	0.018	∼7	2016	683
VG10-C8	Iodolyte Z-150	102	0.435	40.9	0.018	∼7	2016	683
VG11-C8	Iodolyte Z-150	93	1.160	36.1	0.043	∼10	2016	683
Erythrosine B	Iodolyte Z-150	88	1.019	36.0	0.032	∼5.5	2016	683
BAI–COOH	I_2_, LiI	79	1.13	33	0.029	7.8	2018	684
CB5	EL-HSE	115	1.516	34.1	0.059	∼16	2018	685
CB6	EL-HSE	117	1.135	31.4	0.044	∼7	2018	685
CB7	EL-HSE	117	2.001	32.6	0.076	∼13	2018	685
CB8	EL-HSE	117	1.717	32.9	0.066	∼11	2018	685

**Fig. 45:**
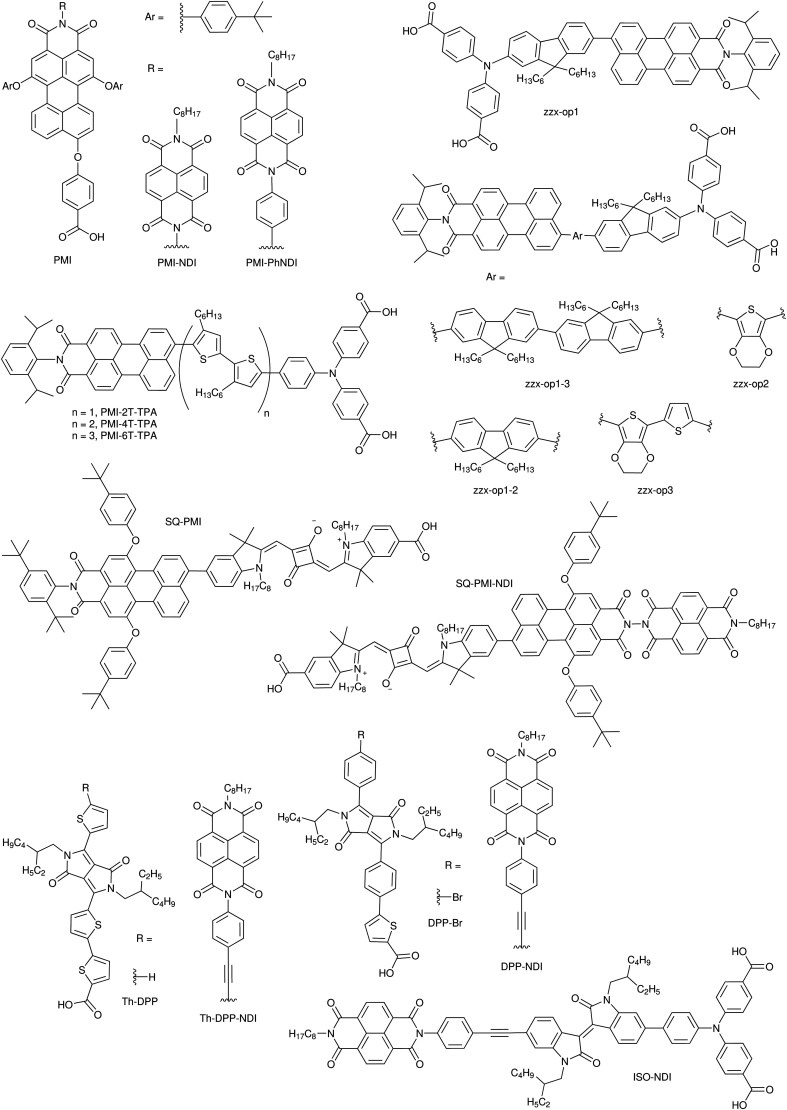
Examples of perylene monoimide- and naphthalene diimide-based sensitizers for p-type DSCs.

The π-linker (*e.g.* oligothiophenes, fluorenes) length can also be optimized to maximize the absorption coefficient, the breadth of the spectral response, the energy offset at the interfaces with the semiconductor and electrolyte, the dye loading, the charge-transfer efficiency and recombination rate.^652,654–657^ PMI-*n*T-TPA series with oligothiophene bridges of different lengths greatly increased device performances (PCE = 0.09%, 0.19% and 0.41% for *n* = 1, 2 and 3 respectively) by further extending the charge-separated state lifetime (Fig. 45).^550^ Other examples include PMI-4T-TPA (*J*_SC_ = 3.40 mA cm^−2^),^582^ T4H (*J*_SC_ = 6.74 mA cm^−2^),^655^ BH4 (*J*_SC_ = 7.40 mA cm^−2^),^657^ PMI-6T-TPA (*J*_SC_ = 7.0 mA cm^−2^),^686^ zzx-op1 (*J*_SC_ = 4.36 mA cm^−2^)^646^ and zzx-op1–2 (*J*_SC_ = 7.57 mA cm^−2^).^645^ Fairly small structural changes to the dye seem to have a big impact, for example comparing O2 (*J*_SC_ = 1.43 mA cm^−2^, *V*_OC_ = 94 mV, FF = 37%, PCE = 0.05%)^658^ to a thienoquinoidal dye (with a I^−^/I_3_^−^ electrolyte: *J*_SC_ = 8.20 mA cm^−2^, *V*_OC_ = 120 mV, FF = 34%, PCE = 0.33%; with a Co(iii/ii) electrolyte: *J*_SC_ = 6.5 mA cm^−2^, *V*_OC_ = 226 mV, FF = 34%, PCE = 0.50%).^644^ The EH series of p-type sensitizers with a D–A–π–A framework were prepared containing triphenylamine (TPA) as a donor, an electron-deficient 2,3-diphenylquinoxaline as the auxiliary acceptor, various thiophene derivatives as the π-linkers, methylene malonitrile as the electron acceptor, and carboxylic acid as the anchoring group.^659^ The p-DSC sensitized by EH174 with a bithiophene π-linker and with one anchoring group performed best (PCE = 0.207%, *J*_SC_ = 4.84 mA cm^−2^, *V*_OC_ = 137 mV, FF = 31.2%) and EH162 with an EDOT π-linker and double anchoring groups performed worst in the series.

**Fig. 46:**
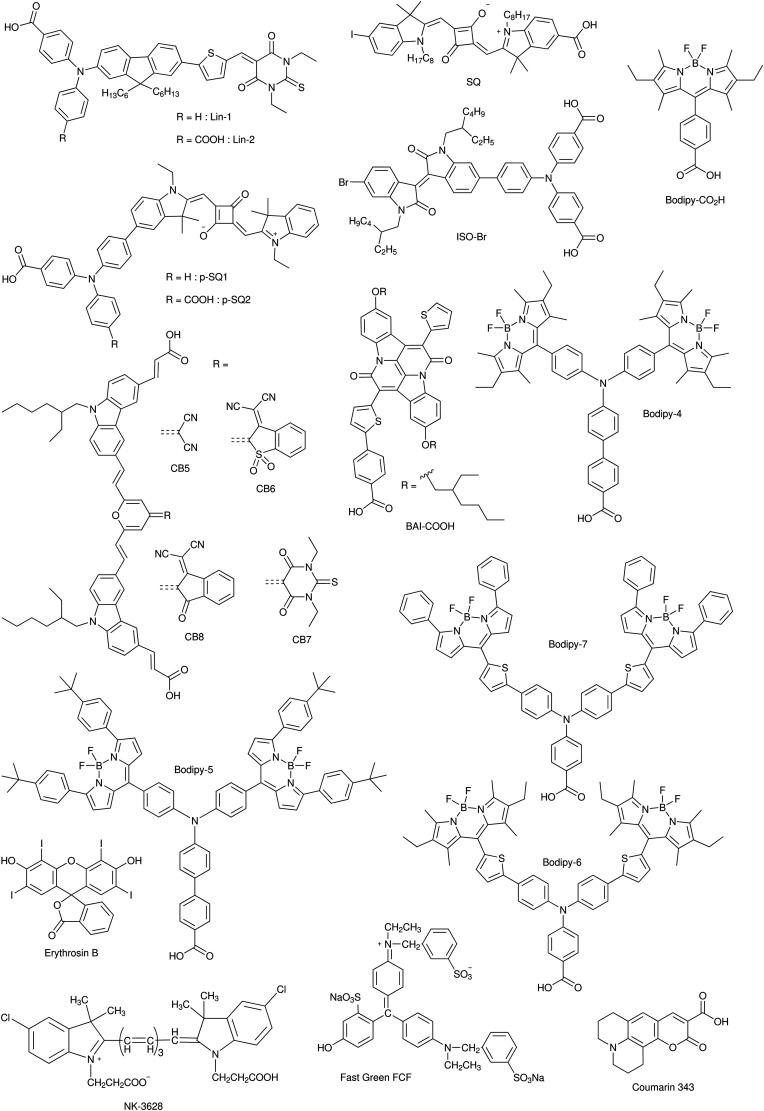
Examples of different sensitizers for p-type DSCs.

The importance of the push–pull structure and the influence of the thiophene π-spacer have been demonstrated with bodipy dyes (Fig. 46). These are relatively straightforward to synthesize and simple modifications to the structure can be made to tune the absorption and emission wavelengths across the visible spectrum.^660^ The performance of bodipy dyes anchored through benzoic acid at the *meso* position is quite low, but push–pull bodipy dyes with a triphenylamine donor linked through a thiophene spacer to the bodipy chromophore perform much better (*e.g.* bodipy-6 PCE = *ca.* 0.3% and *J*_SC_ = 3.15 mA cm^−2^).^661^ The electronic coupling between the donor and the chromophore is important and bodipy dyes with methyl pyrrole groups give a lower photocurrent compared to the pyrrole analogues (IPCE bodipy-4 = 27%, bodipy-7 = 53%, *J*_SC_ = 5.87 mA cm^−2^), which is attributed to better electronic communication with the NiO substrate.^662^ Kubo *et al.* reported a NIR-absorbing π-extended dibenzo-bodipy dye applied in p-type DSCs with a I^−^/I_3_^−^ electrolyte.^663^ Despite the push–pull structure – arising from the triphenylamine donor units and nitrothiophene acceptor – and the broad spectral response (up to 850 nm) the performance was still limited by rapid recombination at the dye/NiO interface (*V*_OC_ = 79 mV, *J*_SC_ = 0.61 mA cm^−2^, FF = 25%, PCE = 0.012%).

Generally, having two anchoring groups per triphenylamine unit is less favourable than having two acceptors because the extinction coefficient tends to be higher with two acceptors and the dye loading may be more compact.^659^ There have been some exceptions, such as the zzx-op series of fluorene-bridged biphenylamine-perylenemonoimide dyes, where the fluorene bridge was directly appended to biphenylamine to ensure good donor/acceptor coupling. W2 with an electron-withdrawing 1,3-benzothiadiazole bridge and an octyl-2-cyanoacrylate acceptor also performed well (*J*_SC_ = 4.16 mA cm^−2^, *V*_OC_ = 121 mV, FF = 33%, PCE = 0.166%).^664^ In certain cases, such as dye 3 *vs.* dye 5^665^ and p-SQ1 *vs.* p-SQ2,^666^ a double anchoring group can improve the solar cell performance through enhancing the binding strength between the dye and the semiconductor, thereby facilitating more efficient charge transfer, or by suppressing the dark current.^665,666^

Typically, carboxylic acid anchoring groups are used; however, until recently, there has been little research into whether or not this is the best choice.^687^ Alternative anchoring groups have been proposed, including pyridine,^571,626,650,667,668^ di(carboxylic acid)pyrrole,^669,670^ hydroxamic acid,^671^ di(carboxylic acid)triazole,^638^ catechol,^622^ carbodithioic acid,^622^ methyl phosphonic acid,^622^ acetylacetone (acac),^668,672^ alkoxysilane,^188^ coumarin,^673^ aniline,^668^ phosphonic acid,^668^ hydroxyqinoline,^668^ and dipicolinic acid.^668^ Phosphonic acid is one of the strongest binding groups and is resistant to both acid and base, but can present some synthetic challenges.^668,673^ Odobel *et al.* and Gibson *et al.* compared the charge-transfer dynamics at the dye/NiO interface for a number of anchoring groups and found that the anchoring group did not significantly influence the rates.^668,673^ This finding is consistent with the work of Housecroft *et al.* who compared the benchmark dye P1 with the phosphonic acid derivative PP1.^674^ The solar cell performance of both dyes was similar, PP1: PCE = 0.054–0.069%, IPCE = 10% at *λ*_max_ = ∼500 nm; P1: PCE = 0.065–0.079%, IPCE = 13.5% at *λ*_max_ = 500 nm.

Recombination at the dye/semiconductor surface appears to be a limiting factor to achieving high quantum efficiencies, unlike the analogous TiO_2_ devices.^688,689^ Perylene-based donor–acceptor dyads with varying acceptor units (such as either perylene itself coupled to a triarylamine donor, or NDI or C_60_ appended to a perylene) led to one of the most important breakthroughs in terms of extending the lifetime of the charge-separated state long enough to enable alternative redox mediators to be used (see below).^627^ The *J*_SC_ for PMI-6T-TPA and P1 were similar when I^−^/I_3_^−^ was used as the electrolyte (*J*_SC_ = 5.35 *vs.* 5.48 mA cm^−2^), but the *V*_OC_ was larger (218 *vs.* 84 mV), possibly due to reduced charge recombination at the electrolyte/electrode interface.^564^ Subsequently, there have been a number of reported dye series showing the benefits of the auxiliary acceptor on reducing charge recombination and, consequently, improving the device performance. These include Warnan *et al.*'s iodo-squaraines (SQ-PMI-NDI with I^−^/I_3_^−^: *J*_SC_ = 2.73 mA cm^−2^, *V*_OC_ = 95 mV, FF = 32%, PCE = 0.083%; with Co(iii/ii): *J*_SC_ = 1.17 mA cm^−2^, *V*_OC_ = 175 mV, FF = 27%, PCE = 0.055%),^675^ and Odobel *et al.*'s diketopyrrolopyrrole (DPP) and isoindigo series,^676,677^ which demonstrate the necessity for an appended NDI acceptor group to deliver good solar cell performance. NiO/Th-DPP-NDI produced a *J*_SC_ of 8.2 mA cm^−2^, which is comparable to the record dyes CAD3 and QT-1.

Porphyrin dyes have been applied in state-of-the-art n-type DSCs, providing record efficiencies. However, rapid electron–hole recombination has limited their application in p-type DSCs.^678,679,690^ Odobel *et al.* attempted to improve their performance by covalently attaching methyl viologen and naphthalene diimide (NDI) acceptors at the *meso* position (ZnP-NDI dye), but these systems were limited by inefficient regeneration by I^−^/I_3_^−^.^680^ Chernick *et al.* developed a series of free-base and nickel asymmetric push–pull porphyrins with alternating *meso* substituents, electron-withdrawing pentafluorobenzene, electron-donating/coordinating 4-pyridyl ligand, and an electron withdrawing/synthetically modifiable 4-cyanophenyl unit.^653^ The porphyrins performed similarly to C343 (IPCE = 26%, PCE = 0.082% for the nickel porphyrin). Coordinating an electron acceptor such as C_60_PPy through the metal center of zinc porphyrins improves the p-DSC performance.^673,679^ Better p-DSC results were reported by Coutsolelos *et al.* who applied three covalently-linked donor–acceptor zinc porphyrin-fullerene (ZnP-C_60_) dyads (C_60_trZnPCOOH, C_60_trZnPtrCOOH and C_60_ZnPCOOH) with a triazole ring spacer between the porphyrin and C_60_ or anchoring group.^681^ Long-lived charge-separated states were observed in all three cases, due to a shift in electron density from the chromophore to the acceptor. The lifetime was enhanced by the presence of the triazole spacer for the dyads in solution, but it made only a moderate impact on the rate of charge separation and recombination when the dyads were adsorbed on NiO. However, the triazole ring did improve the photovoltaic performance. The presence of the C_60_ acceptor improved the solar cell performance compared to the C_60_-free reference compound PhtrZnPCOOH (with I^−^/I_3_^−^ and C_60_trZnPCOOH: PCE = 0.076%; with Co(iii/ii) and C_60_trZnPtrCOOH: PCE = 0.074%). The best performance for a porphyrin photosensitizer in a NiO device so far was reported by Spiccia *et al.*^682^ ZnP1 contained a perylenemonoimide (PMI) electron acceptor linked through a fluorene and a Zn(ii) porphyrin with alkyl chains as a π-conjugated bridge to a di(*p*-carboxyphenyl)amine (DCPA) electron donor. The configuration led to a red-shifted absorption onset to the near-IR region (∼800 nm) compared to the PMI-free reference dye ZnP0 (∼650 nm) and the benchmark PMI-6T-TPA (∼700 nm). With the tris(acetylacetonato)iron(iii/ii) redox mediator, ZnP1 (PCE = 0.92%) outperformed the ZnP0 sensitiser (PCE of 0.29%) but despite the broader spectral response, it did not perform better that the benchmark PMI-6T-TPA dye (2.0% PCE), possibly due to aggregation on the NiO surface.

To complement the state-of-the-art dyes for n-DSCs, red-NIR absorbing dyes have been developed. This is important for tandem devices, where the aim is to increase the spectral response and the *V*_OC_. A well-known class of red-NIR absorbing dyes are squaraines such as the VG and p-SQ series.^666,683^ Indigo is a naturally occurring red-absorbing dye, but its poor solubility makes it challenging to apply in solar cells. A bay-annulated indigo (BAI) was applied in p-DSCs producing a promising photocurrent (*J*_SC_ = 1.14 mA cm^−2^), but the performance was limited by aggregation and charge recombination.^684^ Using a strong electron acceptor to lower the LUMO level in triphenylamine-based push–pull dyes shifts the absorption towards the red.^643^ Examples are COCO and COCN,^649^ the pyran-based dyes CB7 and CB8,^685^ and the CAD series.^649,651^

### 5.4 Electrolytes

The I^−^/I_3_^−^ liquid electrolyte is most frequently chosen for p-type DSCs for compatibility with n-type DSCs.^643^ The composition can be optimized for the p-type system by the choice of solvent, typically acetonitrile, and additives, for example using lithium salts to lower the valence band potential, promote charge transport, limit charge recombination and increase the *V*_OC_.^573,586,628,691,692^ Ionic-liquid iodide sources such as 1-butyl-3-methylimidazolium iodide (BMII), 1-ethyl-3-methylimidazolium (EMII) and dimethylpropylimidazolium (DMPII) have also been shown to give good performance.^586^

Drawbacks to the I^−^/I_3_^−^ redox mediator include strong light absorption in the blue region, its corrosivity and the small difference between the redox potential of this electrolyte (315 mV *vs.* NHE) and the Fermi level of NiO, which limits the *V*_OC_ of these devices to 100–200 mV.^628,693^ Exchanging I^−^/I_3_^−^ for a transparent alternative with a more negative redox potential can increase the *V*_OC_ of p-type DSCs. For example, the 5,5′-dithiobis(1-phenyl-1*H*-tetrazole) and sodium 1-phenyl-1*H*-tetrazole-5-thiolate couple has a redox potential of 245 mV *vs.* NHE, about 70 mV more negative than that of the iodide electrolyte.^668,694^ With optimised dyes, this electrolyte improved the *V*_OC_ compared to I^−^/I_3_^−^ and maintained a good *J*_SC_.^695,696^

**Fig. 47:**
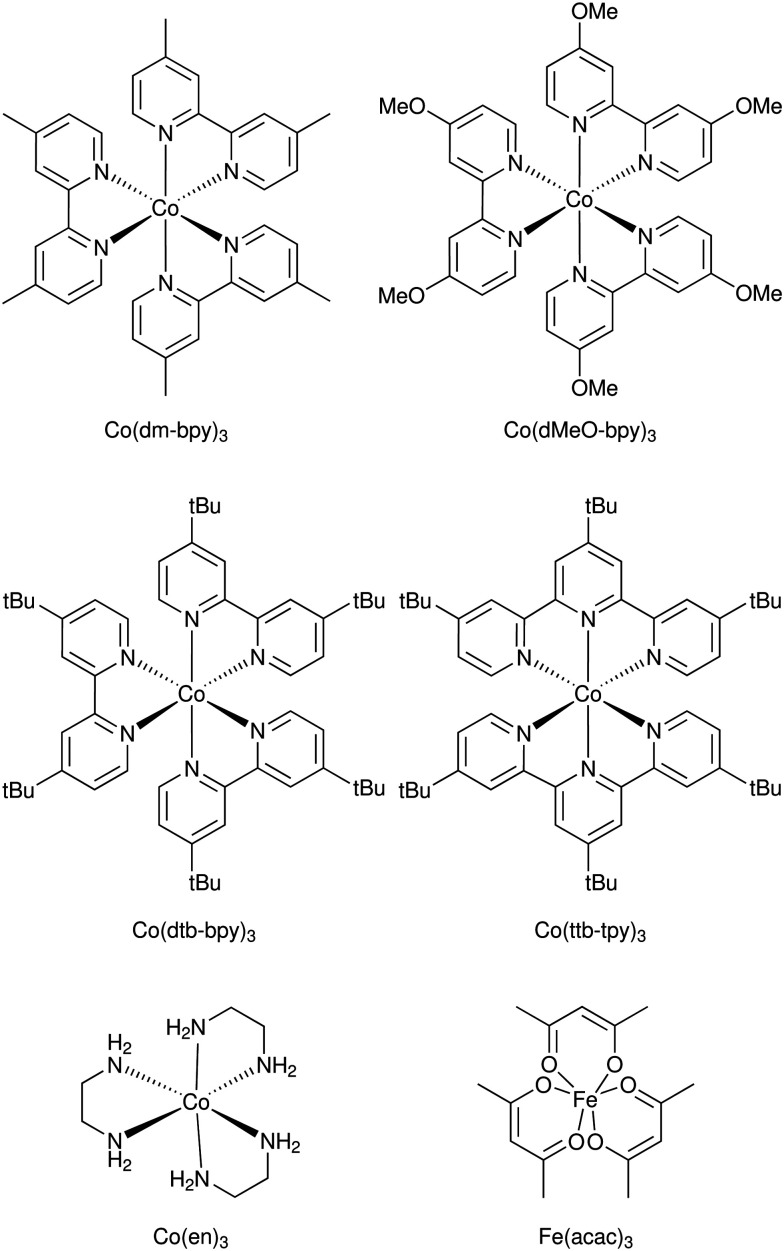
Structures of different redox mediators applied in p-DSCs.

Coordination complexes have given the most encouraging improvement to device efficiency (see Table 12). Co(iii/ii) complexes (Fig. 47) offer better optical transparency and tunable redox potentials compared to I^−^/I_3_^−^.^697^ Slower recombination at the electrolyte/electrode interface and more negative redox potentials than I^−^/I_3_^−^ frequently translate to higher *V*_OC_ (*ca.* 200–300 mV).^698,699^ However, a long-lived charge-separated state (dye^−^/NiO^+^) is required for dye regeneration with transition metal-based electrolytes to be efficient and in return, not all dyes are suitable. As mentioned above, a secondary electron acceptor, such as PMI or NDI, is required to generate long-lived dye radical anions.^697,698^ PMI-NDI sensitized NiO and a [Co(dtb-bpy)_3_]^2+/3+^ redox electrolyte led to a high *V*_OC_ of 350 mV and an overall PCE of 0.20%.^627^ Modification of the peripheral ligands leads to differences in recombination rate and redox potential, leading to efficiencies ranging from 0.04 to 0.24%.^697^ The first example of a p-type DSC with an efficiency exceeding 1% was with PMI-6T-TPA and Co(iii/ii) tris(1,2-diaminoethane) ([Co(en)_3_]^2+/3+^).^698^ Interestingly, this redox mediator also performs well in aqueous electrolytes (PCE = 2%, IPCE_max_ = ∼40% between pH 8–11).^700^ The device efficiency was raised from 1.3% to 2.51% by substituting Co(en)_3_ for [Fe(acac)_3_]^0/−^.^103^ This is the highest reported efficiency to date for a p-type DSC.

**Table 12:** Photovoltaic characteristics of p-type DSCs employing various redox mediators or solid-state ETMS. IPCE values with the approximation sign are a visual estimate taken from plotted data

Mediator/HTM	Sensitizer	*V*_OC_ (mV)	*J*_SC_ (mA cm^−2^)	FF (%)	PCE (%)	IPCE max (%)	Year	Ref.
Co(dtb-bpy)_3_	DPP-NDI	379	1.52	29	0.17	Not reported	2017	573
Co(dtb-bpy)_3_	PP2-NDI	342	1.72	39.7	0.31	∼21	2018	696
Co(dtb-bpy)_3_	PMI-NDI	340	2.00	35	0.24	33	2011	697
Co(dtb-bpy)_3_	PMI-PhNDI	210	0.78	29.3	0.048	∼14	2011	699
Co(dtb-bpy)_3_	PMI-PhC_60_	180	0.58	38.8	0.040	∼23	2011	699
Co(dtb-bpy)_3_	18	85	0.342	23.6	0.007	Not reported	2011	699
Co(dtb-bpy)_3_	19	85	0.250	28.9	0.006	Not reported	2011	699
Co(dtb-bpy)_3_	C343	190	0.25	32	0.015	∼2	2009	627
Co(dtb-bpy)_3_	PI	80	0.26	26	0.006	∼3	2009	627
Co(dtb-bpy)_3_	PINDI	350	1.66	34	0.20	31	2009	627
T^−^/T_2_	PMI-CO_2_H	161	1.52	25.4	0.062	∼20	2020	668
T^−^/T_2_	PMI-HQ	164	2.21	23.8	0.086	∼21.5	2020	668
T^−^/T_2_	PMI-DPA	168	1.33	24.6	0.055	∼26	2020	668
T^−^/T_2_	PMI-acac	169	2.08	27.9	0.098	∼32	2020	668
T^−^/T_2_	PMI-PO_3_H_2_	181	1.27	17.7	0.041	∼20	2020	668
T^−^/T_2_	P1	304	1.73	44	0.23	∼19	2013	694
T^−^/T_2_	PMI-6T-TPA	285	5.3	34	0.51	∼50	2015	695
T^−^/T_2_	PP1	169	1.60	30.5	0.082	∼17	2018	696
T^−^/T_2_	PP2	158	1.82	31.5	0.090	∼17	2018	696
T^−^/T_2_	PP2-NDI	212	4.31	33.9	0.23	∼30	2018	696
Co(dm-bpy)_3_	PMI-NDI	125	2.32	29	0.08	28	2011	697
Co(dMeO-bpy)_3_	PMI-NDI	200	2.42	34	0.17	30	2011	697
Co(ttb-tpy)_2_	PMI-NDI	240	1.61	33	0.13	31	2011	697
Co(en)_3_	PMI-6T-TPA	654	5.23	43	1.48	Not reported	2016	700
Fe(acac)_3_	PMI-6T-TPA	645	7.65	51	2.51	57	2015	103
PCBM	DPP-PYRO	228	0.32	32	0.023	∼3	2017	701
PCBM	DPP-Br	198	0.45	32	0.028	∼4.5	2017	701
ZnO	BH4	480	0.346	39.4	0.07	∼3	2019	702
ZnO	TIP	535	0.855	39.8	0.18	∼5	2019	702
ZnO	PB6	440	0.68	45	0.135	∼4	2019	703
TiO_2_	PB6	480	0.020	66	0.006	∼0.08	2018	704

In addition to metal complex-based electrolytes, anionic metal oxide clusters known as polyoxometalates (POMs) are versatile and transparent electron reservoirs.^709^ POMs co-adsorbed on the surface of NiO can slow down the rate of charge-recombination and increase the *V*_OC_.^710^ Lindqvist *et al.* applied POMs (M_6_O_19_^2−^) directly as redox mediators in p-DSCs, giving a four to five-fold increase in *V*_OC_ compared to I^−^/I_3_^−^.^711^ Increasing the solubility of POMs could increase the short-circuit current of these cells to deliver competitive efficiencies.

Recently, a few solid-state p-DSCs (p-ssDSC) have been reported.^712^ Phenyl-C_61_-butyric acid methyl ester (PCBM) is a well-known solid electron-transfer material used in organic photovoltaics. Tian *et al.* found that the PCE of their p-ssDSC with P1 and PCBM was low due to slow dye regeneration by the electron transport material.^712^ Applying molecular dyads such as DPP (diketopyrrolopyrrole)-pyromellitimide (PYRO) can improve the performance.^701^ However, much improvement is required to deliver an efficient solid-state p-type DSC. Tian *et al.* followed up their work with organic electron transport mediators by completely removing the electrolyte/organic charge transport component and directly depositing TiO_2_ or ZnO on the NiO, so that the dye injects electrons directly into the n-type semiconductor and holes directly into the p-type semiconductor.^702–704,713^ This concept was first introduced by Bandara *et al.* but incomplete pore filling by the n-type semiconductor limited the cell performance.^591,714^ Tian *et al.* ha d optimised the interface between the dye and the semiconductors by engineering the structure of the dye and the deposition of the n-type semiconductor. Solar cells based on the TIP dye, containing an indacenodithieno[3,2-*b*]thiophene linker, gave PCE = 0.18%, *J*_SC_ = 0.86 mA cm^−2^, *V*_OC_ = 535 mV, FF = 40% and max IPCE of 5%.^702^

### 5.5 Photoelectrochemistry and photovoltaic performance

The key charge transfer processes that occur in a p-DSC under operation are summarised in Fig. 42 and the reactions important to photocathodes are:

Electron transfer to the excited dye D* from the NiO valence band (“hole injection”):D* + NiO → D^−^ + NiO|h^+^

Re-oxidation of the dye by the redox electrolyte (“dye regeneration”):^693^D^−^ + I_3_^−^ → D + I_2_^−^˙ + I˙

Diiodide disproportionation to form triiodide and iodide:2I_2_^−^˙ → I_3_^−^ + I^−^

Recombination between the reduced dye and a hole in NiO:D^−^ + NiO|h^+^ → D + NiO

Recombination of a hole in NiO with the reduced species in the electrolyte:2NiO|h + 3I^−^ → 2NiO + I_3_^−^

Over the last ten years, there have been extensive studies into the dynamics of each process. Charge injection is typically a fast process, between 100 femtoseconds to 100 picoseconds according to transient absorption spectroscopy and time-resolved infrared spectroscopy.^668,688^ The surface electronic states at the interface between NiO and a series of bodipy dyes have been studied by hard and soft XPS and the good overlap between the dye HOMO and semiconductor valence states was consistent with rapid light-induced charge transfer.^660^ Recombination at the dye^−^/NiO^+^ interface, however, is also fast, occurring on a picosecond to nanosecond time scale in simple dye systems such as bodipy and perylene.^660,715,716^ Regeneration occurs from a nanosecond up to microsecond time scale. Competition between recombination and regeneration is responsible for the poor efficiency for p-type DSCs.^642,661,699^ Recombination between holes in NiO with the reduced dyes contributes to the low FFs.^717,718^ A hole-hopping charge transport mechanism has been proposed for NiO, arising from “trap states” such as Ni^3+^ and Ni^4+^.^568,610,719^

The Ni^3+^ states are important for charge transport and charge recombination at the NiO/electrolyte and NiO/dye interfaces.^565,566,577,720^ Competition between these processes leads to the short diffusion length and low fill factors observed in NiO-based DSCs.^721^ Unlike TiO_2_, the charge carrier lifetime is independent of light intensity or charge density and a charge hopping process, regulated by ions in the electrolyte, takes place at the NiO surface.^722^ The NiO preparation and deposition route affects both the charge lifetime and transport time.^560,564,565^ Small amplitude light-modulated transient photocurrent and photovoltage decay measurements and electrochemical impedance spectroscopy (EIS) have also been used to study the effect of doping, of applying an insulating blocking layer and of varying the redox mediator and dye structure on the hole lifetime and transport time.^576,697,699,723,724^ Application of a NiO blocking layer to suppress charge recombination led to a higher photocurrent and fill factor.^725^ A Ni(CH_3_COO)_2_ treatment to the NiO film was also shown to suppress the hole recombination and led to a 31.3% improvement in the photovoltaic performance.^726^ Insulating coatings of Al_2_O_3_ and TiO_2_ on the NiO surface increase the recombination resistance and increase the *V*_OC_ and efficiency of the device.^570,571^ Chemical treatments such as immersing in NaBH_4_ or NaOH have also been used to improve the *V*_OC_ and FF by addressing the Ni^3+^ surface states and decreasing recombination.^568,727,728^

Developing new semiconductors, such as alternative metal oxides with better hole mobility compared to NiO or reducing electronic vacancies present above the valence band edge could favour charge transport over recombination.^719^ Lithium ions ha ve been well-characterized as dopants for NiO and improve the electrical properties of the films, shifting the valence band position to more positive potential, altering the density of states, narrowing the trap energy distribution and increasing the energy barrier for charge recombination.^577^ Doping NiO with Co has been shown to increase the charge transport lifetime from ∼5 ms for pure NiO to more than two-fold for 2% and 6% Co-doped NiO films. The *V*_OC_ increased from 122 mV up to a maximum of 158 mV with >6% cobalt doping due to a lowering of the flat-band potential of the NiO by a few tens of mV and also to higher hole lifetimes for the Co-doped cells than those for pure NiO cells.^576^ Guldi *et al.* studied the charge transfer processes in CuO photocathodes with I^−^/I_3_^−^ using electrochemical impedance spectroscopy.^585^ They probed the effect of calcination temperature, electrode thickness, and electrolyte ratio on the charge transfer resistance *R*_CT_, charge collection efficiency *η*_cc_, diffusion coefficient *D* and hole lifetime *τ*_h_ and determined that a 300 °C calcination temperature, a film thickness of 5.0 μm and an I^−^/I_3_^−^ electrolyte ratio of 2.5 : 1 gave the optimum balance of dynamics and best device performance. The experiments also revealed less recombination at the electrode/electrolyte interface for CuO compared to NiO.

The dye structure has been shown to affect the charge transfer dynamics. Push–pull donor–acceptor dyes and molecular dyad and triad structures have been developed to extend the charge-separated state lifetimes from tens of picoseconds into the microsecond to millisecond regime.^637,666,673,679,699,715,729–731^ By extending the linker it is possible to increase the charge-separated state lifetime without decelerating the rate of charge separation.^550,657^ Varying the coupling between the chromophore and the linker increases the charge-separated state lifetime, but this comes with a sacrifice to the charge injection yield, so a balance must be struck to optimize the performance.^634^ Adding bulky alkyl chains to the dye, or forming a compact arrangement of dye molecules at the electrode surface inhibit charge recombination at the semiconductor/electrolyte interface, leading to longer charge lifetimes.^645,646,725^ A surprise came from exploring the charge transfer dynamics of P1 and CAD3,^647^ which – despite having relatively short charge-separated state lifetimes (*ca.* <10 ns) – still generate relatively high photocurrents in NiO DSCs. When iodine and lithium iodide were added, the charge-separated state decayed over a one order of magnitude longer time scale compared to the lifetime recorded in the presence of an inert electrolyte. It is possible that there is pre-association of the electron acceptor in the electrolyte with the cationic dyes, or reduction of the high valence states on the surface of NiO by the electron donor in the electrolyte. I^−^ in the electrolyte has been shown to reduce the Ni^3+^ states, which are thought to be responsible for rapid charge recombination, so a dual effect might be responsible for the increased charge-separated state lifetime in the presence of the redox electrolyte.^566,568,691^

With electrolytes based on cobalt polypyridyl complexes, the hole lifetimes were shown to be – like with I^−^/I_3_^−^ – strongly dependent on light intensity, whereas the hole transport times were largely independent of light intensity. Charge transport times have been found to be almost independent from the structure of the cobalt complexes, but charge lifetimes depend on the steric bulk of the cobalt polypyridyl complex. Most importantly, charge lifetimes were shown to be longer with cobalt complexes (particularly with bulky ligands) compared to I^−^/I_3_^−^.^699^ Electrolyte additives, such as chenodeoxycholic acid, have also been shown to slow recombination at the electrode/electrolyte interface.^573^ In these examples, the longer charge lifetimes corresponded with higher open circuit voltage.

### 5.6 Tandem devices

Tandem DSCs offer an opportunity to increase the solar cell efficiency beyond what can be attained by a single photoelectrode. The top electrode captures the higher energy photons and the transmitted lower energy photons are captured by the bottom electrode. However, the low performance of the photocathodes limits the performance of tandem DSCs. Early studies focused on proving the principle that the *V*_OC_ of the tandem DSC is the sum of the individual n-type and p-type DSCs, but the devices suffered from very low photocurrents and poor fill factors.^547^ These first tandem DSCs typically contained I^−^/I_3_^−^ as the redox mediator, but substituting it for metal complexes and commercial photosensitizers for dyes designed specifically for photocathodes has led to an improved performance.^627^ In particular, advances have been made in developing dyes which absorb in the red to NIR region of the solar spectrum to complement state of the art photosensitizers for TiO_2_ devices. For example, Gibson *et al.* reported a tandem cell with up to 5.2 mA cm^−2^ employing the cationic charge-transfer dye CAD3 on NiO and a benchmark charge-transfer dye D35 on TiO_2_.^643^ Guldi *et al.* incorporated Zn(ii) phthalocyanines (ZnPc) in photocathodes based on CuO and assembled them in tandem devices with N719 on TiO_2_, giving a light harvesting range from 300 nm to 800 nm (*J*_SC_ = 1.28 mA cm^−2^, *V*_OC_ = 860 mV, FF = 63%, PCE = 0.69%).^732^ A more encouraging efficiency of 2.42% was reported by Bach *et al.* with PMI-6T-TPA as the dye and Fe(acac)_3_ as the electrolyte.^550^ Odobel *et al.* reported a dye-sensitized tandem cell with a diketopyrrolopyrrole (DPP)-based sensitizer at the photocathode (NiO/Th-DPP-NDI) and a TiO_2_/D35 photoanode. The tandem DSC efficiency was greater than that of the individual p-type and n-type devices (*J*_SC_ = 6.73 mA cm^−2^; *V*_OC_ = 910 mV; PCE = 4.1%).^548^

Deepa *et al.* reported the most efficient tandem cell to date at 9.76% for a device which included a photocathode with a nickel pthalocyanine dye (NiPcTs) on NiO supported over carbon fabric.^733^ The photoanode was assembled from conducting core/shell copper@carbon dots anchored to CdS quantum dots on TiO_2_ and a polysulfide electrolyte was used for compatibility with the CdS. The efficiency of the photocathode half-cell was quite low (0.039%) but when incorporated into the hybrid tandem device it improved the efficiency by almost 3% compared to the photoanode device with carbon fabric alone as the counter electrode (6.69%). Most of the improvement came from the higher photocurrent.

The key issue with tandem devices is that, although great steps have been made in improving the photocurrent density by developing new photosensitizers and improving the photovoltage through developing new redox mediators, the efficiency is still limited by the valence band position of the p-type semiconductor. A semiconductor with a lower valence band than NiO or replacing TiO_2_ with a material with a higher-lying conduction band is needed to improve the built-in potential of tandem devices. Other than the tandem device by Guldi *et al.* described above,^732^ a tandem cell by Kaya *et al.* assembled from a photocathode of CuCrO_2_ with a coumarin 6 organic dye, iodide-based redox mediator and N719-sensitized TiO_2_ photoanode gave a PCE of 2.33% with *V*_OC_ of 813 mV, *J*_SC_ of 4.83 mA cm^−2^, and fill factor of 59%.^734^ If an alternative p-type transparent semiconductor with a valence band 0.5 V deeper than NiO could be found, an efficiency above 20% would be possible. However, as described above, there is no obvious choice to replace NiO yet.

## 6 DSCs for solar fuel

The diffused and intermittent nature of solar energy dictates the requirement for energy storage in solar energy conversion strategies. Chemical bonds are arguably the most appealing choice for this goal. For over two billion years, nature's photosynthesis has been converting solar energy into chemical potential, while also sequestering CO_2_ and producing most of the oxygen in our planet. All fossil fuels we use today are derived from the natural photosynthetic process. Artificial photosynthesis aims to emulate natural photosynthesis to generate solar fuels and commodity chemicals from sunlight using H_2_O, CO_2_ and N_2_ as feedstocks. In the last decade, DSCs have played key roles in one of the fastest-growing artificial photosynthetic approaches, Dye-Sensitized Photoelectrosynthesis Cells (DSPECs). A DSPEC is a modified DSC in which the reduced form of the redox shuttle in the anode compartment is replaced with an oxidation catalyst (*e.g.* a water oxidation catalyst), while the oxidized form of the redox shuttle in the cathode compartment is replaced with a reducing catalyst (*e.g.* a proton reduction catalyst). In a DSC the goal is to convert sunlight into electricity to power a device or to charge a battery. In a DSPEC the goal is to convert and store sunlight into chemical bonds, producing O_2_ or a commodity chemical at the anode and a fuel at the cathode.

**Fig. 48:**
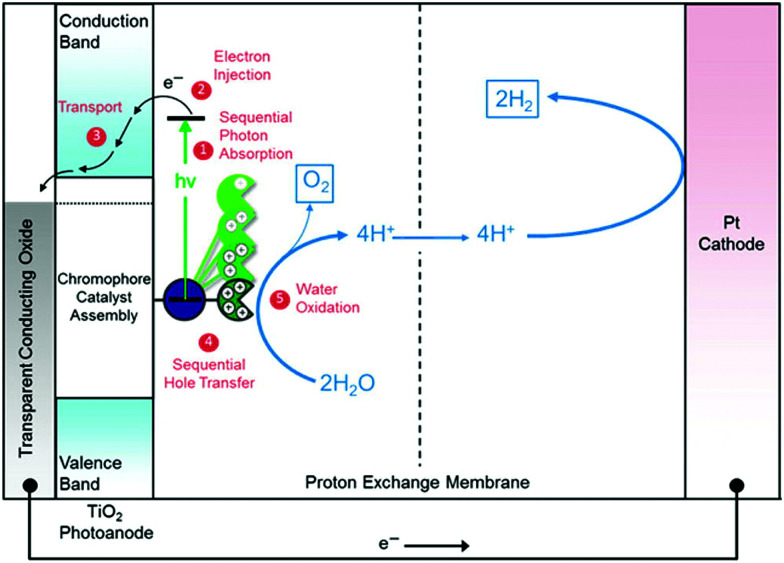
Schematic diagram of a DSPEC for light-driven water splitting with an assembly-derived TiO_2_ photoanode for water oxidation to O_2_ and a dark Pt cathode for proton/water reduction to H_2_. Reprinted with permission from ref. 735. Copyright 2015 American Chemical Society.

Fig. 48 shows a schematic representation of a DSPEC for water splitting. Light-driven water oxidation takes place at the photoanode, composed of a chromophore-catalyst assembly on a mesoporous n-type semiconductor film, and proton/water reduction occurs at a dark Pt cathode. At the photoanode, the chromophore in the chromophore-catalyst assembly is responsible for light absorption and subsequent electron injection from its excited state(s) into the conduction band of the semiconductor. The injected electrons are transported to a transparent conducting oxide (TCO) electrode and delivered to the cathode for proton/water reduction. Electron transfer from the water oxidation catalyst to the oxidized chromophore initiates the activation of the water oxidation catalyst and regenerates the chromophore. This process is repeated four times leading to O_2_ evolution at the photoanode and H_2_ evolution at the dark cathode, ideally in a 1 : 2 O_2_/H_2_ ratio, returning the chromophore-catalyst assembly to its initial state.

Meyer and co-workers reported the first DSPEC in 1999,^736^ almost a decade after the pioneering DSC work of O'Regan and Grätzel.^5^ The DSPEC carried out light-driven dehydrogenation of isopropanol to acetone at the photoanode with H_2_ generation at the dark Pt cathode. It took yet another decade for the development of the first DSPEC for water splitting by Mallouk and co-workers in 2009.^737^ Nevertheless, the last 12 years have seen an impressive development in this area.^735,738–775^ The first DSPEC for water splitting reported by Mallouk and co-workers generated a photocurrent of 12.7 μA cm^−2^ at pH 5.8 under 450 nm light irradiation (7.8 mW cm^2^) with an internal quantum yield of 0.9% and a faradaic efficiency for O_2_ generation of 20%.^737^ Just a decade later, DSPECs are reaching photocurrent densities of ∼2.2 mA cm^−2^ at pH 7.0 under 1 sun illumination with an incident photon to current efficiency (IPCE) of 29% at 450 nm and faradaic efficiencies for O_2_ generation over 70%. Correcting for the injection yield of only ∼42% for the chromophore at pH 7.0, the efficiency of the cell, excluding the losses at the core/shell interface, is a remarkable 67%.^774^

### 6.1 Photoanodes and photocathodes

In theory, a tandem DSPEC (discussed below in Section 6.5) with both a photoanode and a photocathode could provide significant advantages over a DSPEC with just a photoanode and a dark cathode. Absorption of one photon at the photoanode and one photon at the photocathode by two complementary dyes would emulate the Z-scheme in natural photosynthesis and enable coverage of a wider range of the solar spectrum. In addition, a photocathode would provide additional voltage that could eliminate the need for an applied bias to generate H_2_ at the photocathode or enable access to fuels from CO_2_ using catalysts with higher overpotentials than those used to produce H_2_ as the fuel. Unfortunately, as in the case of DSCs, the development of tandem DSPECs has been hampered by the lack of suitable p-type photocathode materials.

#### 6.1.1 Photoanodes

Most DSPECs reported to date function as a photoanode to drive oxidation reactions with a dark cathode to generate H_2_. The photoanode consists of a mesoporous 5–15 μm thick nanoparticle film of an n-type wide bandgap semiconductor deposited on a TCO, and a combination of a chromophore or sensitizer and an oxidation catalyst. DSPEC photoanodes have greatly benefited from prior developments of DSC photoanodes, both in terms of the n-type semiconductor material as well as in terms of the photosensitizer or chromophore.

In a typical DSC, the photosensitizer or chromophore is anchored to the semiconductor material, while the redox shuttle is free to diffuse from the anode to the cathode and back. In a DSPEC, on the other hand, the oxidation catalyst must be immobilized on the photoanode and it must undergo multiple, successive oxidations to complete one cycle or turnover. For this reason, the position and distance of the oxidation catalyst with respect to the photosensitizer and the semiconductor are key aspects in determining the overall cell performance. This has led to many approaches in the assembly of chromophores and catalysts on the nanoparticles' surfaces of the semiconductor.

The first DSPEC reported used a chromophore-catalyst assembly in which the two were chemically linked through a bridge prior to loading onto the semiconductor surface.^736^ This design allows precise control of the distance between chromophore and catalyst and positions the catalyst away from the semiconductor surface to inhibit recombination reactions between injected electrons and oxidized catalyst molecules. However, such chromophore-catalyst assembly designs require cumbersome synthetic procedures. The first chromophore-catalyst assembly for water splitting was not suitable for a DSPEC: In the excited state of the chromophore, the excited electron was localized in the bridging ligand and the injection yield into the conduction band of TiO_2_ was less than 5%.^776^ Other chromophore-catalyst assembly designs failed to perform in a DSPEC configuration because the oxidized chromophore did not have enough oxidizing power to generate the Ru^V^=O form of the catalyst, a key intermediate for the initial O–O bond formation step.^735,777,778^

Introduction of carbene-based water oxidation catalysts in chromophore-catalyst assemblies enabled access to O–O bond formation already at the less-oxidized Ru^IV^=O form of the catalyst with additional redox power available from the weakly-coupled Ru(iii) chromophore. Water-splitting DSPECs involving a single-site water oxidation catalyst in the chromophore-catalyst assembly were successfully developed.^742,749,769^

The discovery of the [Ru(bda)(L)_2_] (bda: 2,2′-bipyridine-6,6′-dicarboxylate; L is a monodentate ligand , Fig. 51) water oxidation catalysts by Sun and co-workers^779,780^ and their incorporation into chromophore-catalyst assemblies led to significant improvements on DSPEC performance because of their low overpotential and high rates for water oxidation.^763,766^ This type of catalysts was first used on a DSPEC configuration by loading the catalyst into a Nafion overlayer deposited on top of a Ru(bpy)_3_-sensitized TiO_2_ mesoporous film.^738^ Nevertheless, the first significant DSPEC breakthrough was achieved by co-loading a Ru(bpy)_3_-type chromophore and a Ru-bda catalyst on TiO_2_.^743^ Photocurrent densities up to 1.7 mA cm^−2^ at pH 6.8 were obtained with a 14% IPCE at 450 nm and 83% faradaic efficiency for O_2_ generation. This co-loading strategy has been successfully used in DSPEC photoanodes with a variety of chromophore-catalyst combinations.^748,753,759,765,768^

Mallouk and co-workers introduced a layer-by-layer approach to load chromophores and catalysts on the surface of the semiconductor.^737^ The authors prepared a Ru(bpy)_3_-type chromophore containing one phosphonated bipyridine ligand for TiO_2_-anchoring, and another ligand functionalized with a malonate group that was selective for binding and stabilizing the colloidal IrO_2_·*n*H_2_O water oxidation catalyst nanoparticles. A related layer-by-layer strategy for nanostructured metal oxide films was developed by Meyer and co-workers^781^ based on previous studies on Si and Au planar electrodes.^782,783^ This strategy takes advantage of the strong affinity of phosphonate groups for high valent cations such as Zr(iv), and it has been successfully applied in a variety of DSPEC photoanode designs as well as in photocathodes, discussed below.^741,745,759,784^ In yet another layer-by-layer strategy, a thin film of an oxide (TiO_2_, Al_2_O_3_, *etc.*) a few nm thick is deposited by atomic layer deposition (ALD) on top of the pre-loaded chromophore. The water oxidation catalyst is then loaded onto this oxide layer using typical metal-oxide anchoring groups. In addition to enabling loading of the catalyst, the ALD overlayer stabilizes and protects the chromophore. The ALD layer-by-layer approach has been extensively used in DSPEC photoanodes.^764,767,785^

Electropolymerization techniques have also been used to prepare DSPEC photoanodes. In this approach, electropolymerizable groups (*e.g.* vinyl groups) are introduced in both chromophore and catalysts which end up chemically linked during the electropolymerization process.^750,754,761^ A variation of this strategy simply electropolymerizes a film of the catalyst on top of a dye-functionalized electrode. The low water solubility of the polymer retains the catalyst molecules on the pores of the mesoporous electrode.^752^

**Fig. 49:**
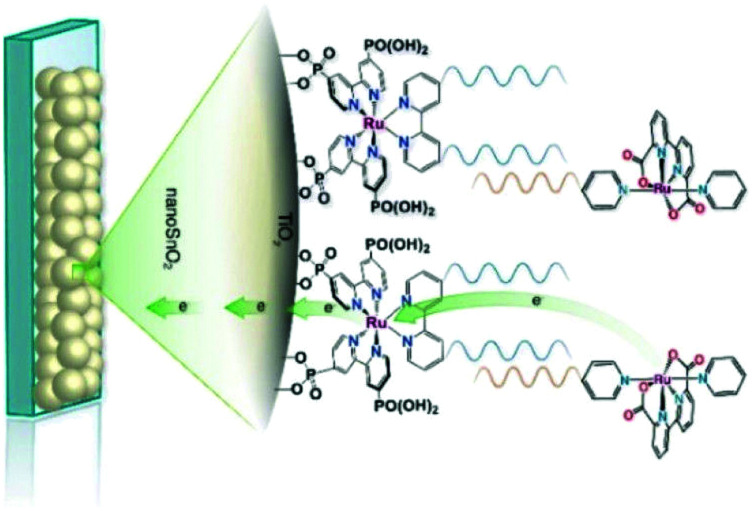
Self-assembled bilayer of a chromophore-catalyst assembly on a metal oxide. Reprinted with permission from ref. 774. Copyright 2019 American Chemical Society.

A recent development for the assembly of chromophores and catalysts on an electrode surface takes advantage of hydrophobic interactions between long alkyl chains to build self-assembled bilayers (SAB, Fig. 49).^786^ In this approach, a chromophore containing both anchoring groups and long alkyl chains is loaded onto an electrode surface and the resulting chromophore-functionalized electrode is then immersed in a solution of the water oxidation catalyst which has also been functionalized with long alkyl chains. The long alkyl chains in the catalyst molecules self-assemble with the long alkyl chains in the chromophore to create a SAB. This approach allows easy combination of various chromophores and catalysts with the distance between them controlled by the length of the alkyl chains.

A water splitting DSPEC built using this strategy reached photocurrent densities of ∼2.2 mA cm^−2^ under 1 sun illumination at pH 7.0 with an IPCE of 29% at 450 nm and faradaic efficiencies for O_2_ generation over 70%. Correcting for the injection yield of only ∼42% for the chromophore at pH 7.0, the efficiency of the cell – excluding the losses at the core/shell interface – is a remarkable 67%. At pH 4.7, the cell was operated over a 3 hour period with an 86% faradaic efficiency for O_2_ generation.^774^

#### 6.1.2 Photocathodes

The development of photocathodes for DSCs and DSPECs has been hampered by the lack of suitable p-type semiconductor materials. As it is the case for photoanodes, a DSPEC photocathode comprises a semiconductor material deposited on a TCO glass, a chromophore and a catalyst. For the last two decades, NiO has been the dominant wide bandgap p-type semiconductor material for sensitized photocathodes since its first report as a photocathode in a DSC.^549^ Problems associated with the high density of traps and the low hole mobility have been identified as the main limitations of this material.^566^ Target atomic deposition (TAD) has been used as a method to passivate defect states and improve the optical and electronic properties of NiO.^572,787,788^ For example, TAD of Al increases the *V*_OC_ of NiO in DSCs, leading to a ∼ three-fold improvement in their performance.^572^ DSPECs operate in aqueous solutions and this introduces additional complications due to the appearance of localized electronic states centered on surface –OH groups associated with Ni vacancies. The thereby enabled proton-coupled charge transfer processes are deleterious to the performance of aqueous NiO photocathodes.^789^

**Fig. 50:**
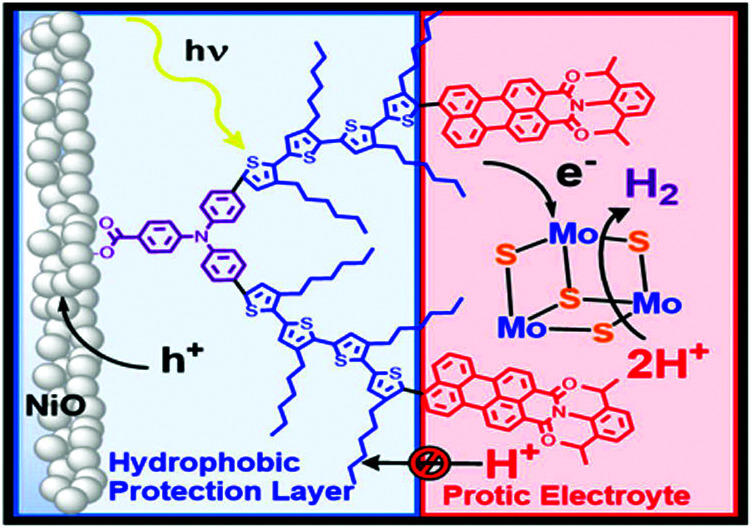
Photocathode for hydrogen generation. Reprinted with permission from ref. 791. Copyright 2016 American Chemical Society.

The first sensitized photocathode for light-driven hydrogen generation was reported by Sun and co-workers.^790^ It consisted of a cobaloxime molecular catalyst in solution and an organic triphenylamine-type dye anchored on nanostructured NiO. An analogous photocathode, but with the cobaloxime catalyst also anchored to the NiO, was used to prepare an organic dye tandem water splitting DSPEC.^753^ The cell reached photocurrent densities of −300 μA cm^−2^ at pH 7 with an IPCE of 25% at 380 nm. Wu and co-workers reported a dye-sensitized photocathode that displayed high stability in strongly acidic solutions.^791^ As shown in Fig. 50, the organic dye was composed of a triphenylamine (TPA) donor moiety that was linked to two perylenemonoimide (PMI) acceptor groups *via* oligo-3-hexylthiophene-conjugated π-linker groups on each side of the donor moiety. Carboxylic acid groups on the TPA donors allowed the anchoring on NiO, while the hydrophobic hexyl groups in the thiophene linkers offered protection for both the anchors and the NiO from the very acidic environment in which they were embedded. An acid-stable cubane molybdenum sulphide cluster – [Mo_3_S_4_]^4+^ – was chosen as the proton reduction catalyst. The cell sustained photocurrents beyond −180 μA cm^−2^ for more than 16 hours at pH 0 in 1.0 M HCl with a 49% faradaic efficiency for H_2_ generation. Artero and co-workers also reported a NiO-based photocathode using a TPA chromophore covalently linked to a cobaloxime catalyst.^792^

Wasielewski and co-workers used ALD to deposit a thick Al_2_O_3_ layer on top of the NiO film with a modified perylene-3,4-dicarboximide chromophore (PMI). In addition to providing protection for the NiO from the aqueous solution, the Al_2_O_3_ layer films allowed longer charge separated lifetimes as characterized *via* femtosecond transient absorption spectroscopy and photoelectrochemical techniques. Light-driven H_2_ generation was demonstrated with both cobaloxime and Dubois' Ni(L)_2_-type catalysts (L is a diphosphine).^793^ Meyer and co-workers also used an ALD layer of Al_2_O_3_ on NiO as a bridge between a Ru(bpy)_3_-type chromophore and a Ni(L)_2_ proton reduction catalyst, an assembly strategy similar to that reported above for photoanodes.^764,767,785^

The shortcomings of NiO as a p-type material for photocathodes has prompted scientists to look for new alternatives. Reisner and co-workers have used the delafossite-type material CuCrO_2_ as a suitable p-type semiconductor for visible light-driven H_2_ generation.^794^ The semiconductor was functionalized by co-loading a phosphonated diketopyrrolopyrrole dye with a Ni(L)_2_ proton reduction catalyst. The hybrid CuCrO_2_ photocathode displayed a photocurrent of −15 μA cm^−2^ at 0.0 V *vs.* RHE in pH 3 aqueous electrolyte solution under UV-filtered simulated solar irradiation. The photocathode displayed good stability and a turnover number of 126 for H_2_ production was recorded for their Ni(L)_2_ catalyst during a 2 hour operation. The CuCrO_2_-based system outperformed a similar photocathode based on NiO, but product generation was limited by the low dye and catalyst loadings. In a follow-up study, macropore architectures of inverse opal CuCrO_2_ led to a five-fold increase in loading.^795^

More recently, Meyer and co-workers used boron-doped Si as the p-type material.^784^ Si nanowires ∼18 μm long were modified by physical vapor deposition of a thin Ti layer (∼10 nm), followed by ALD of a ∼3.0 nm TiO_2_ layer. The latter protected the p-type Si electrode from photodegradation and allowed anchoring of phosphonate-functionalized perylene-diimide (PDI) chromophores. Ni(L)_2_ proton reduction catalysts were introduced using the Zr-bridged layer-by-layer approach.^781^ The integrated photocathode was capable of delivering a photocurrent density of about −1.0 mA cm^−2^ under zero applied bias (*vs.* NHE).

Photocathodes for CO_2_ reduction are even more challenging due to the larger overpotentials of CO_2_ reduction catalysts compared to proton reduction catalysts. Nevertheless, significant progress has been made on this front in recent years. Ishitani and co-workers reported a photocathode for reduction of CO_2_ to CO using a NiO electrode functionalized with a Ru(ii)-Re(i) supramolecular complex.^796^ During a 5 hour operation, the photocathode carried out 32 turnovers with a faradaic efficiency of 65% for CO, although the experiments were carried out in a DMF : triethanolamine (5 : 1) mixture with an applied bias of −1.2 V *vs.* Ag/AgNO_3_. The same Ru(ii)–Re(i) supramolecular complex on a CuGaO_2_ p-type semiconductor displayed photoelectrochemical activity for the conversion of CO_2_ to CO with 68% faradaic efficiency in an aqueous electrolyte solution with an applied bias of −0.7 V *vs.* Ag/AgCl.^797^

More recently, Meyer and co-workers developed photocathodes using a novel method based on a binary p–n junction to convert sunlight into electrons with high energy to drive the CO_2_ reduction reaction to produce formate in an efficient way.^798^ Such photocathodes featured a semiconductor p–n junction constituted of GaN nanowire arrays on silicon together with surface-bound molecular assemblies to perform light absorption and catalysis. The reduction of CO_2_ to formate proceeded at a stable photocurrent density of about −1.1 mA cm^−2^ during 20 h of irradiation, with faradaic efficiencies of up to 64%.

### 6.2 Photosensitizers

The photosensitizers (or chromophores) used in DSPECs must meet additional demands compared to those used in DSCs. In the photoanode, the oxidized photosensitizer must be capable of oxidizing the water oxidation catalyst through a series of increasingly challenging oxidation states during the water oxidation cycle. In addition to the thermodynamic requirements for such a task, some (or all) of the oxidation steps of the catalysts are proton-coupled in nature and this adds to the kinetic barriers for these oxidations. Because of this, in a DSPEC the photosensitizer remains for longer times in its oxidized form compared to DSCs, which leads to significantly faster decomposition of the photosensitizer. Another important issue is that injection efficiency into the conduction band of the semiconductor is pH-dependent due to the pH dependence of the latter.^799,800^ In addition, in the aqueous environment where DSPECs operate, long-term stability of the anchoring groups of the photosensitizer remains a challenge. Phosphonic acid groups have been the dominant choice in this regard for both photoanodes and photocathodes, although recent studies include the use of significantly more robust silanes.^801–804^

[Ru(bpy)_3_]^2+^-Type chromophores have dominated the DSPEC literature in the photoanode side^737,738,741,743,745,748,750,752,754,759,761,765–767,774,784^ with a few other examples including zinc porphyrins^763^ and triphenylamine derivatives.^753,762,764^ Recent efforts have been made on developing new chromophores with higher oxidation potentials that could enable faster oxidation of the water oxidation catalyst, the use of water oxidation catalysts with higher overpotentials, and DSPEC operation at low pH. Unfortunately, tuning the ground state redox potential of the chromophore commonly also affects their excited state energy levels. Brudvig and co-workers developed a series of CF_3_-substituted free-base and metalated porphyrins that displayed redox potentials in the 1.25–1.56 V *vs.* NHE range, higher than the unsubstituted analogues.^82^ The new porphyrins showed high efficiency for injection into SnO_2_ but poor injection into TiO_2_. Meyer and co-workers prepared a series of complexes of the type [Ru(bpy)_2_(N–N)]^2+^ (N–N is a polypyridyl ligand with low-lying π* levels). With this approach, the absorption spectra of the new chromophores could be red-shifted up to *λ*_max_ = 564 nm for the lowest MLCT, compared to 449 nm for the parent [Ru(bpy)_3_]^2+^ complex. In addition, the redox potentials for the Ru^3+/2+^ couples could be enhanced by more than 250 mV. However, these improvements came at the expense of the excited state energy becoming more positive than the conduction band of TiO_2_, rendering these chromophores unsuitable for excited state electron injection.^805^ In a follow-up work, introduction of electron-withdrawing groups on the bipyridine ligands enabled a ∼200 mV increase in the Ru^3+/2+^ couple for surface-bound chromophores. But once again, this improvement resulted in more positive excited state energies and smaller driving forces for electron injection.^806^ More recently, the introduction of –CF_3_ and/or –PO_3_H_2_ groups on all ligands in tris-homoleptic [Ru(bpy)_3_]^2+^-type chromophores resulted in redox potential upshifts of the Ru^3+/2+^ couple up to 1.6 V *vs.* NHE while retaining a similar absorption profile and photophysical properties compared to the [Ru(bpy)_3_]^2+^ complex.^807^ These chromophores enabled photochemical water oxidation to be carried out at pH 1 for the first time.

Significant efforts have been also made on developing organic chromophores for both photoanodes and photocathodes. This subject has been recently reviewed by Abbotto and co-workers and it is beyond the scope of this review.^808^ A recent review on chromophores/sensitizers for photocathodes for both DSCs and DSPECs has been published by Odobel and co-workers.^809^

### 6.3 Catalysts

**Fig. 51:**
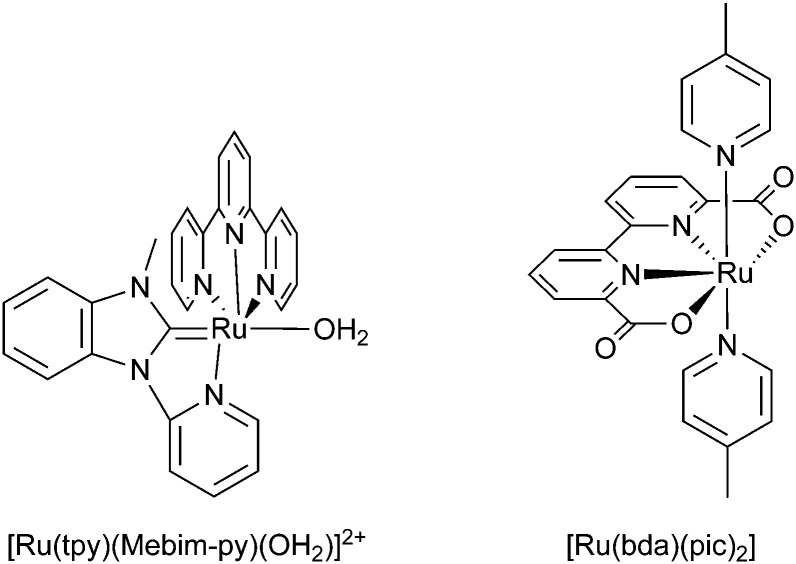
Structures of Ru-based water oxidation catalysts.

Most studies reported to date in DSPECs have used only a handful of catalysts for both photoanodes and photocathodes. After the first DSPEC for water splitting reported by Mallouk and co-workers^737^ that used IrO_x_ nanoparticles as water oxidation catalyst in the photoanode, the majority of the reports that followed used either [Ru(tpy)(Mebim-py)(OH_2_)]^2+^ (tpy: 2,2′:6′,2′′-terpyridine; Mebim-py: 1-methyl-3-(pyridin-2-yl)-1*H*-benzo[*d*]imidazol-3-ium-2-ide)^810–813^ or [Ru(bda)(L)_2_], Fig. 51.^779,780^

[Ru(tpy)(Mebim-py)(OH_2_)]^2+^ is a single-site water oxidation catalyst and retains its homogeneous catalytic performance when immobilized on the surface of photoanode materials. Nevertheless, its high overpotential and low rates for water oxidation resulted in poor performances for DSPECs using this catalyst. [Ru(bda)(L)_2_]-type catalysts, on the other hand, follow a bimolecular pathway for water oxidation and do not retain their impressive homogeneous catalytic performance when heterogenized, generating μ-oxo bridged, blue dimer-like structures on the surface of the electrode.^814,815^ These structures are the true water oxidation catalysts on the surface and their number is only a fraction of all the heterogenized monomeric catalysts that have the proper distance and orientation to generate μ-oxo bridged species. Nevertheless, their high water oxidation activity and low overpotential enable DSPECs using these catalysts to display remarkable performance.

Single-site water oxidation catalysts capable of oxidizing water at high rates and low overpotentials, and which retain their homogeneous catalytic activity when heterogenized could potentially lead to significant improvements in DSPEC performance. Llobet and co-workers have reported single-site water oxidation catalysts with impressive rates although at neutral and basic pH values.^816,817^ Combining the features of single-site bisphosphonate catalysts ([Ru(bpaH_2_)(L)_2_], bpaH_2_ is 2,2′-bipyridine-6,6′-diphosphonic acid)^818^ and fast bimolecular [Ru(bda)(L)_2_]-type catalysts, Concepcion and co-workers have developed hybrid water oxidation catalysts ([Ru(bpHc)(L)_2_], bpHc is 6′-(hydroxyoxidophosphoryl)-[2,2′-bipyridine]-6-carboxylate) that are faster than the parent catalysts under identical conditions in both chemical and photochemical water oxidation.^815,819^ Nevertheless, the performance of these catalysts in DSPEC configurations has not been reported to date.

On the photocathode side, catalysts can be separated into two groups: catalysts for proton/water reduction (other than platinum) and catalysts for CO_2_ reduction. Most studies where a molecular catalyst was used to carry out proton/water reduction at the photocathode used either cobaloxime-type catalysts^753,792,793^ or the Ni(ii) bis(diphosphine) complexes developed by DuBois and co-workers.^784,793–795,820–823^ A cubane molybdenum-sulfide cluster was also successfully used for proton reduction in extremely acidic (pH 0) conditions and displayed significant stability with up to 16 hours of H_2_ generation with no degradation.^791^ However, none of these catalysts have been able to perform at the level of a platinum electrode in a DSPEC. Bias voltages are required to drive H_2_ evolution even with platinum, with just a few exceptions. Nevertheless, the applied bias is typically due to improper alignment between the conduction band of the photoanode material and the redox potential of the H^+^/H_2_ couple rather than overpotential issues related to the proton reduction catalyst. DSPEC studies where water oxidation at the photoanode is accompanied by CO_2_ reduction at the photocathode are scarce. Ishitani and co-workers have reported CO_2_ reduction to CO at a CuGaO_2_ photocathode using a chromophore-catalyst assembly consisting of a [Ru(bpy)_3_]^2+^-type chromophore and a [Re(bpy)(CO)_3_(Br)] catalyst.^797^ Nevertheless this was not a true DSPEC, because water oxidation was carried out by direct bandgap excitation of the photoanode rather than by sensitization. Meyer and co-workers reported an integrated photocathode based on the [Re(bpy)(CO)_3_(Cl)] catalyst for CO_2_ reduction to CO in a CO_2_-saturated bicarbonate aqueous solution. The integrated photocathode was stable toward CO_2_ reduction for over 10 h with a faradaic efficiency of ∼65%.^802^ Meyer and co-workers also reported a series of photocathodes using [Ru(bpy)(CO)_2_Cl_2_] as the catalyst for CO_2_ reduction. The photocathodes reduced CO_2_ to formate at stable photocurrent densities of around −1.1 mA cm^−2^ during 20 h of irradiation with faradaic efficiencies of up to 64% in CO_2_-saturated bicarbonate aqueous solution.^798^

### 6.4 Electrode materials

Electrode materials play several key roles in DSPECs. They serve as the solid support for chromophores and catalysts, and in many cases they play a role in chromophore-catalyst integration strategies. In addition, electrode materials are also key in charge separation, and electron collection and/or delivery.

#### 6.4.1 Electrode materials for photoanodes

As in the case of DSCs, mesoporous thin films of TiO_2_ have been the workhorse electrode material for photoanodes in DSPECs since the initial reports of Meyer *et al.*^736^ and Mallouk *et al.*^737^ In the last decade, however, the use of core–shell electrode materials has proven to be more advantageous. Core–shell structures with a conductive core (tin-doped indium oxide, ITO, and tin-doped antimony oxide, ATO) for fast and efficient electron collection and transport, and a TiO_2_ shell for electron injection introduced by ALD were used in 2013 in a DSPEC for water splitting where the photoanode was the disc in a rotating ring-disc electrode system.^742^ A chromophore-catalyst assembly containing the catalyst [Ru(tpy)(Mebim-py)(OH_2_)]^2+^ (Fig. 51) was anchored to the TiO_2_ layer *via* phosphonic acid groups on the chromophore. Light was introduced from the bottom of the cell and the oxygen generated at the photoanode disc was detected and quantified at the ring (Pt). In 2015, the same chromophore-catalyst assembly was used in a more conventional DSPEC setup, but with a SnO_2_–TiO_2_ core–shell as photoanode material.^749^ The replacement of ITO with SnO_2_ as the core led to a 5-fold enhancement in photocurrent, reaching up to 1.97 mA cm^−2^ in a pH 7 phosphate buffer. The stability of the cell was improved by introducing Al_2_O_3_ or TiO_2_ overlayers *via* ALD to protect the anchoring groups, a clear example of the many roles played by electrode materials in DSPECs.

The use of SnO_2_–TiO_2_ core–shell electrode materials combined with the use of [Ru(bda)(pic)_2_]-type water oxidation catalysts (Fig. 51) has led to significant developments in DSPECs.^754,759,764–767,774,824,825^ In the case of SnO_2_–TiO_2_ core–shell electrodes, the initial rationale for their better performance compared to bare TiO_2_ electrodes was based on the difference in the conduction band positions of SnO_2_ and TiO_2_. The more positive conduction band of SnO_2_ should act as a sink from which recombination of injected electrons should be significantly slower. Initial studies by Meyer and co-workers supported this with oxidized chromophores persisting into the millisecond timescale when anchored onto SnO_2_–TiO_2_ core–shell surfaces.^826^ However, follow up studies by the same group discovered that there is actually a new electronic state at the SnO_2_–TiO_2_ interface located more positive than both SnO_2_ and TiO_2_.^800^ The success of core–shell electrode materials in DSPECs and other applications is a clear example that finding new materials is not always the only solution. Oftentimes creative solutions with known materials might provide similar or even better outcomes.

#### 6.4.2 Electrode materials for photocathodes

NiO has been the dominant wide bandgap p-type semiconductor material for sensitized photocathodes since its first report as a photocathode in a DSC.^549^ As previously mentioned, problems associated with the high density of traps and low hole mobility have been identified as the main limitations of this material.^566^ The use of aqueous solutions in DSPECs brings additional complications due to the appearance of localized electronic states centered on surface –OH groups associated with Ni vacancies. As a result, proton-coupled charge transfer processes affect the performance of aqueous NiO photocathodes.^789^ Other photocathode materials such as CuCrO_2_^794,795^ and CuGaO_2_^797^ have shown more promise than NiO but their performance is still lacking compared to the photoanode side.

Meyer and co-workers used boron-doped Si as the p-type material, protected by a 10 nm Ti layer with an additional 3.0 nm layer of TiO_2_ for anchoring of chromophores.^784^ The integrated photocathode was capable of delivering a photocurrent density of about −1.0 mA cm^−2^ for hydrogen generation under zero applied bias (*vs.* NHE) using a NiL_2_ catalyst for proton reduction to H_2_.

Strategies that creatively combine known materials could prove to be a viable alternative to finding new materials with ideal properties. For example, Meyer and co-workers reported a binary p–n junction strategy to prepare photocathodes that integrate a semiconductor p–n junction (Si/n-GaN) and surface-bound molecular assemblies for light absorption and catalysis. The photocathodes reduce CO_2_ to formate at stable photocurrent densities of −1.1 mA cm^−2^ during 20 h of irradiation with faradaic efficiencies of up to 64%.^798^

### 6.5 Tandem devices

**Fig. 52:**
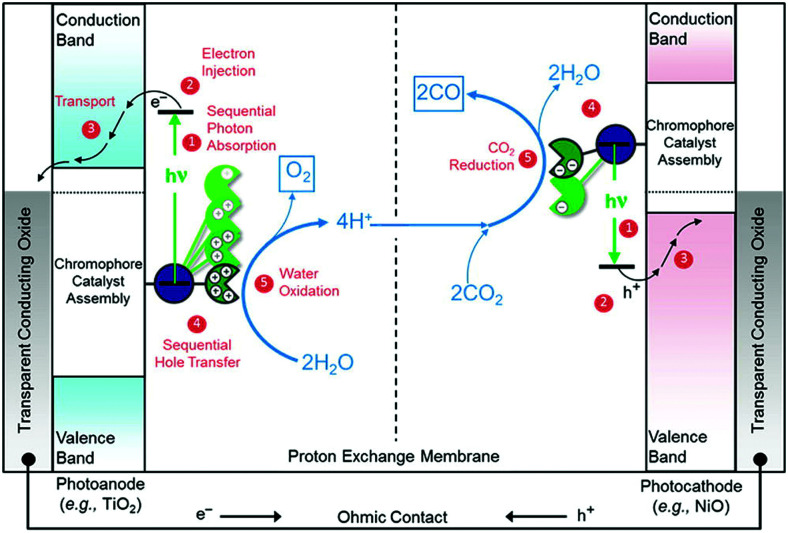
Schematic diagram for a DSPEC for light-driven CO_2_ splitting into CO and O_2_ with an assembly-derivatized TiO_2_ photoanode for water oxidation to O_2_ and an assembly-derivatized photocathode for CO_2_ reduction to CO. Reprinted with permission from ref. 735. Copyright 2015 American Chemical Society.

The net conversion of water and carbon dioxide to oxygen and reduced carbon products in natural photosynthesis is driven by the absorbed energy of two photons for each electron involved in the process (two photosystems in tandem). However, in natural photosynthesis, the two photosystems absorb essentially the same spectral range, which is one of the reasons why this process is relatively inefficient.^827,828^ A thermodynamic analysis indicates that an approach in which the two photosystems absorb different parts of the light spectrum (tandem junction) is crucial to maximize the capability of converting solar energy into fuels for both natural and artificial photosynthetic systems.^827,829,830^Fig. 52 shows a schematic diagram of a tandem DSPEC for solar-driven CO_2_ splitting into CO and O_2_ by the net reaction 2CO_2_ + 4*hν* → 2CO + O_2_.^735^ Replacement of the CO_2_ reduction catalyst in the photocathode with a proton/water reduction catalyst results in a DSPEC for water splitting into O_2_ and H_2_. Ideally, the chromophores in the photoanode and photocathode should have complementary spectral absorption profiles.

**Fig. 53:**
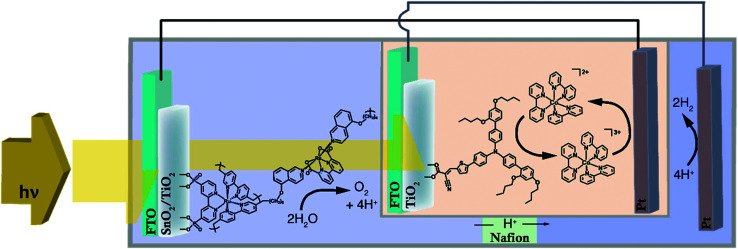
Schematic diagram of a DSPEC wired in series with a DSC. Reprinted with permission from ref. 761. Copyright 2016 American Chemical Society.

Sun and co-workers reported an organic dye-sensitized tandem DSPEC for light-driven water splitting. The photoanode consisted of a thin film (8 μm) of TiO_2_ as electrode material, a triphenylamine-based organic dye and a molecular Ru-based catalyst for water oxidation. The photocathode consisted of a thin film (1 μm) of NiO, a triphenylamine-based organic dye and a molecular Co-based catalyst for proton reduction.^753^ In a 50 mM phosphate buffer at pH 7, the cell reached photocurrent densities of 70 μA cm^−2^ for water splitting under 100 mW cm^−2^ irradiation with no applied bias. Meyer and co-workers reported a tandem DSPEC with sustained photocurrents of 250 μA cm^−2^ over a 2.5 h irradiation time with faradaic efficiencies of 73% and 54% for O_2_ and H_2_, respectively.^784^ The photoanode consisted of a SnO_2_–TiO_2_ core–shell electrode with a RuP_2_^2+^ chromophore and a Ru(bda) water oxidation catalyst assembled using the layer-by-layer approach. The photocathode, described in the previous section, consisted of a boron-doped p-type Si protected with a 10 nm Ti layer with an additional 3.0 nm layer of TiO_2_ for PDI′ chromophore anchoring. A NiL_2_ proton reduction catalyst was assembled with the PDI' chromophore *via* a zirconyl bridge using the layer-by-layer assembly strategy. High energy photons were used at the photoanode for water oxidation and low energy photons were used at the photocathode for proton reduction. The performance of the tandem device was limited by the photoanode. Sherman and co-workers reported an alternative approach to tandem DSPEC devices for water splitting. It combines a typical water splitting DSPEC with a DSC to use more efficiently the solar spectrum and eliminate the need for an applied bias, Fig. 53.^760,761^

The fully assembled tandem cell system consisted of a DSPEC incorporating a SnO_2_–TiO_2_ core–shell electrode, a RuP_2_^2+^ chromophore and a Ru(bda) water oxidation catalyst. The chromophore and catalyst were assembled on the surface of the core–shell electrode *via* electropolymerization. The photoanode and a dark Pt cathode were wired in series with a DSC employing either the N719 dye and I^−^/I_3_^−^ mediator or a D35 dye and the Co(bpy)_3_ mediator. The tandem cell achieved unbiased photocurrents of 40 μA cm^−2^ under simulated solar illumination with a solar to hydrogen efficiency of 0.06%.

## 7 Industrialization and commercialization

The Nature paper by Grätzel and O'Regan^5^ triggered expectations for a novel low-cost photovoltaic technology with potential to challenge silicon solar cells, which at the time were still forecast to be expensive to manufacture on a large scale. Shortly thereafter, a few pioneering device manufacturing companies initiated DSC development with commercial ambitions, such as Glas Trösch, Leclanché, and Asulab from Switzerland, ABB and INAP in Germany, Ekologisk Energi in Sweden, Solterra in Italy, and Dyesol in Australia. Since then, a range of industrialisation initiatives in different parts of the world have been created. The most intense period was during 2000–2010, when Asian activities were intense, dominated by Japan. An example of the vast Japanese development activities is the fact that >50% of the >2000 novel DSC patent families submitted in the years 2000–2010 had Japanese origin.^831^ Examples of Japanese companies with strong DSC development during this period are Sharp, Sony, Toyota, Hitachi Maxell, Sanyo, Nippon Oil, Fuji Film, Aisin-Seiki, Fujikura, J-Power Co., Gunze Ltd, Mitsubishi Paper Mills., Sekisui Jushi Corporation, Dai Nippon Printing Company, Nissha Printing, Taiyo Yuden Co., Panasonic Denko, TDK, Spark Plug Co. and Eneos Co Ltd. Equivalent examples from other Asian countries are Dongjin Semichem and Samsung SDI from South Korea and J touch from Taiwan. Further examples of companies with DSC activities during this period are BASF, Bosch, Merck and Tata Steel. Most of these industrial DSC initiatives have been abandoned, whereas some have changed direction during their development, typically from outdoor panels to low-power devices targeting IoT (Internet of Things) applications. In the past ten years, commercial-oriented DSC device activities have been more or less exclusively directed towards see-through aesthetic devices for BIPV applications and small-area devices for low-power applications. Looking at commercialization efforts of the DSC technology throughout the past 30 years, three categories appear: (i) panels to challenge Si, (ii) BIPV *via* aesthetic devices, (iii) niche products for electronic applications. These diverse efforts are discussed in Section 7.3. Throughout the DSC commercialization, a set of module concepts have been used and thoroughly investigated, each one with their respective strengths and challenges (Section 7.1). In parallel to the device-oriented commercialization activities, there has been supplementary industrialization of required material components, manufacturing equipment and services. However, as the major DSC commercial breakthrough has not taken place yet, these industries still operate at a small scale, with various peaks during the most intense DSC commercialization periods.

According to Hagfeldt and co-authors,^8^ a difficulty of evaluating the performance of DSC modules stems from the fact that various definitions of device efficiency are employed. The efficiency of the active area is used in certain situations, whereas the efficiency of the modules' entire area is used in others. In addition, several module sizes are used, and measurements are performed at varying light intensities. In general, publications dealing with module stability provide lower efficiency figures. The comparison of DSC module findings from various publications should then be evaluated with a grain of salt. Sharp's DSC mini-module, with efficiency of 10.7% from the year 2013, is included in the current table of record solar cell efficiencies.^390^

### 7.1 DSC module design

The thorough overview of the five basic DSC module designs presented by Hagfeldt *et al.* is still relevant.^8^ This applies to their definition of a DSC module as well, *i.e.* a device that is considerably larger in both the x and y dimensions compared to a single lab-scale solar cell, and that employs particular solutions to reduce the resistive energy (electron transport) losses. Sandwich and monolithic are still terms used to describe a device construction that has the working and counter electrodes on two separate substrates or on the same one.

The bigger size of a DSC module complicates the manufacturing, performance, and stability compared to those of a test cell. Furthermore, the interconnection of cells in a DSC module may create additional efficiency loss routes, such as mismatched performance of linked cells or undesired electrolyte mass transfer between neighbouring cells. The five sandwich and monolithic module concepts, *i.e.* (i) sandwich Z-interconnection, (ii) sandwich W-interconnection, (iii) sandwich current collection, (iv) monolithic serial connection, and (v) monolithic current collection, have constituted the basis throughout 30 years of DSC device development and commercialization. Their respective advantages and challenges are discussed by Hagfeldt *et al.*^8^ Even though there has been an evolution in DSC chemistry, represented by *e.g.* organic dyes, Cu-based redox mediators and the so-called “zombie cell”,^485^ the five module designs remain.

One complementary module design deserving attention is the work by Takashima *et al.* from NGK Spark Club.^832^ Their so-called ball-grid DSC solution is based on a hybrid copper polyimide flexible substrate covered with a dense carbon counter electrode. The working electrode is contacted to the copper *via* polymer-cored solder balls. The design efficiently enlarges a DSC cell by combining an efficient current collection grid with a high ratio of active area (95%). In addition, a few interesting novel DSC module design options – driven by simplified production processes – have been presented in the past few years at conferences by representatives of the present DSC industry, such as Exeger in Sweden and Song Textile in South Korea. However, as these designs – to the best of our knowledge – have not been presented in the literature, they are not part of this review. Moreover, Ricoh in Japan have recently launched commercial solid-state DSC products where the device concept has not been found in the literature.

### 7.2 DSC stability

For any relevant application, good long-term stability of the DSC is crucial. Degradation of the DSC can have various origins:^336^ (i) dye degradation: dyes can desorb from the TiO_2_ electrode, a process which is accelerated at higher temperatures. Dyes can also be damaged due to chemical reactions; for instance, they can be unstable in their oxidized state, which is the case for N719. (ii) Electron collection: the TiO_2_ electrode can change its performance due to loss of electrical contact between neighboring particles or with the FTO substrate. Furthermore, the energy levels of the TiO_2_ can shift due to changes in the electrolyte. (iii) Redox electrolyte: the redox mediator can undergo chemical changes, such as ligand exchange for cobalt and copper complexes. There can be a loss of the oxidized form of the redox mediator when other species are oxidized due to excitation of TiO_2_ (*e.g.*, loss of triiodide when holes in TiO_2_ oxidize solvent molecules). Lastly, evaporation of the solvent can occur. (iv) Counter electrode: the catalyst can be unstable due to the corrosive nature of the redox mediator or it can be poisoned. The stability of Pt-free counter electrodes was reviewed by S. Yun *et al.*^833^ (v) Sealing: imperfect sealing can lead to loss of electrolyte and/or introduction of water and oxygen into the system, with detrimental effects. (vi) UV light: direct excitation of TiO_2_ can lead to damage due to highly oxidizing holes. Typically, a UV filter needs to be included in practical DSC systems for outdoor use for this reason.

Best stability data to date is obtained for DSCs based on the iodide/triiodide redox system and ruthenium sensitizers. High-temperature stability of such systems was investigated by Desilvestro and co-workers using electrolytes with different solvents – “HSS” (presumably based on sulfolane), 3-methoxypropionitrile (MPN) and γ-butyrolactone (GBL) – which led, respectively, to final relative PCE values of 83%, 60% and 20% after 1000 h at 85 °C in the dark.^834^ Sauvage *et al.* found evidence for solid/electrolyte interphase formation on TiO_2_ nanoparticles using MPN under such conditions, suggesting that TiO_2_ acts as a catalyst for electrolyte degradation.^835^ Mastroianni *et al.* found that degradation under MPP conditions was much more severe than under open circuit conditions.^705^ While negligible degradation was found during 3200 h of outdoor testing, significant degradation was found during controlled testing at elevated temperature (1 sun, 85 °C), which was largely attributed to loss of I_3_^−^ and band edge shifts of the TiO_2_. The Z907 dye, with hydrophobic tails, was found to be stable upon 1200 h of illumination with iodide-based electrolyte and MPN solvent, even in the presence of large concentrations of water.^706^ Good stability data for organic sensitizers was reported by Peng Wang *et al.*^269^ They used co-sensitized organic dyes C268 and SC-4 in combination with an electrolyte containing DMII and EMII ionic liquids and sulfolane, and recorded just 3% loss of PCE of their solar cells (initial PCE 10.1%) after 1000 h of 1 sun illumination at 60 °C. A 1000 h stability test in the dark at 85 °C led to a 9% loss for the same system (Table 13).

**Table 13:** Stability studies of DSC devices with different redox systems

Redox system – solvent	Sensitizer(s)	Conditions	Initial PCE (%)	Final PCE (relative %)	Year	Ref.
I^−^/I_3_^−^ – MPN	N719	3200 h, 1 sun, 85 °C, OC	4.6	67	2012	705
I^−^/I_3_^−^ – MPN	N719	3200 h, 1 sun, 85 °C, MPP	4.7	28	2012	705
I^−^/I_3_^−^ – MPN	Z907	1200 h, 1 sun, 25 °C, OC	7.0	104	2019	706
I^−^/I_3_^−^ – MPN + 20% H_2_O	Z907	1200 h, 1 sun, 25 °C, OC	5.3	123	2019	706
I^−^/I_3_^−^ – DMII, EMII, sulfolane	C268/SC-4	1000 h, 1 sun, 60 °C, OC	10.1	97	2018	269
I^−^/I_3_^−^ – DMII, EMII, sulfolane	C268/SC-4	1000 h, dark, 85 °C, OC	10.1	91	2018	269
Co(bpy)_3_ – MeCN	SM315	500 h, 1 sun, 25 °C, MPP	12.5	80	2014	286
Co(bpy)_3_ – MPN	Z907	2000 h, 1 sun, 25 °C, OC	4.0	91	2014	707
Co(bpy)_3_ – MeCN	D35	1000 h, 60 °C, OC	6.4	85	2014	17
Cu(tmby)_2_ – MeCN	MS5/YX1b	1000 h, 1 sun, 40 °C, OC	13.5	93	2021	12
Cu(tmby)_2_ – MeCN, MPN	Y123	432 h, 1 sun, OC	9.49	79	2021	708
Cu(tme) – MeCN, MPN	Y123	432 h, 1 sun, OC	8.25	91	2021	708

The stability of cobalt-based mediators was reviewed by Bella *et al.* in 2016.^836^ Mathew *et al.* performed 500 h light soaking tests under MMP conditions of high-performing porphyrin-sensitized DSCs, after which a loss of 20% was found, partly attributed to dye desorption.^286^ Jiang *et al.* investigated long-term stability of Z907-sensitized devices with Co(bpy)_3_. With MPN as electrolyte solvent, PCE retained 91% of its initial value after 2000 h of continuous 1 sun illumination with cells kept at open circuit.^707^ 1000 h tests for MeCN-based cells under 1 sun and MPP conditions gave no significant degradation for the best cells. Gao *et al.* performed 1000 h illumination tests at 60 °C for DSC devices with MeCN-based cobalt bipyridine electrolytes and found remarkably good stability for electrolytes with increased concentration of Co^2+^ and Co^3+^.^17^ Boschloo and co-workers investigated the thermal stability of cobalt-based electrolytes with MPN as solvent. They found that addition of bipyridine to the electrolyte could decrease DSC degradation in a 50 days storage test at 70 °C in the dark. With bipyridine and MBI as additives, a 12% loss in PCE was found, compared to a 20% loss with *t*BP as additive.^18^ Cobalt complexes with hexadentate ligands were shown to lead to improved stability in DSC illumination tests in comparison to cobalt trisbipyridine, with no degradation after 100 h in 1 sun.^355,366^

In recent work, Zhang *et al.* demonstrated good long-term performance for Cu(tmby)_2_-based electrolytes in a 1000 h light soaking test at 40 °C.^12^ Ligand exchange with, for instance, *t*BP could be a problem for long-term stability of these copper complexes.^404^ Sun and co-workers developed a stable Cu complex with a pentadendate ligand, which did not display facile ligand exchange. PCE remained at 90% of its initial value after 400 h at 1 sun (25 °C), compared to 80% for devices with Cu(tmby)_2_-based electrolyte.^708^

For all redox electrolytes, more long-term stability tests under MPP 1 sun illumination conditions are needed to reliably assess the performance of DSCs. Testing under open-circuit conditions will not stress the counter electrode at all. Furthermore, the full redox cycle is not occurring under these conditions, as all electrons in TiO_2_ will recombine the oxidized dye and redox couple.

#### 7.2.1 Accelerated and outdoor testing of DSC modules

Over the years, DSC module stability has proven to be possible but challenging. In order to realize DSC modules with long life, a robust device chemistry must be used in combination with a functional encapsulation technique that is chemically compatible with the electrolyte, and which provides a tight barrier against the surroundings, *i.e.* mechanically, thermally and UV light stable. In case serial connections are applied, undesired mass transport of ions between adjacent cells must be avoided. All of this should preferably be realized over small distances to avoid significant surface losses and thus reduced module performance. Experience has shown that such internal barriers often function well at first but cause stability issues over time. In addition, serial-connected cells face the possibility of reverse bias degradation effects, *i.e.* one or several cells in a module that are electrically mismatched, from *e.g*. partial shade, are exposed to high currents. Apart from reduced module performance, this can lead to device degradation. Th is issue can, however, be avoided by using protecting diodes.

In 2010, Hagfeldt *et al.*^8^ reviewed the status of DSC module stability up to that year. They highlighted the observation that publications dealing with module stability generally have lower efficiency values than the publications where stability is not mentioned, likely due to more space for encapsulation and/or use of different device chemistry with lower efficiency values. Still, already in 2010, it was evident that long-term stable DSC modules could be realized. A module stability paper that was highlighted was the one from Kato *et al.*,^837^ who presented results from 2.5 years of outdoor module tests, resulting in approximately 20% degradation of the initial device performance. By comparing the outdoor module ageing results to accelerated illumination tests on the single cell level, the acceleration factor of the light-soaking test was estimated at 11. Another highlighted paper was the one from Dai *et al.*,^838^ who performed one year outdoor testing of their modules resulting in a minor performance decrease, which was not numerically stated in the publication. High temperature storage tests have traditionally been challenging for DSCs. A third highlighted publication was that from Matsui *et al.*,^839^ who demonstrated that it is feasible to obtain excellent module stability over 1000 h storage in darkness at 85 °C and 85% relative humidity. An important module stability paper after 2010 is that from Rong *et al.*^840^ Monolithic serial-connected devices with a side of 100 cm with solid-state electrolyte passed the following two tests with minor performance decrease: (i) 1000 h at 60 °C, 85% relative humidity (RH) and (ii) 300 temperature cycles between −10 and 60 °C (3 h per cycle). In 2011, Kato *et al.*^841^ presented results from 160 days of outdoor tests of DSC modules integrated in solar light devices. They concluded that the *J*_SC_ gradually increased the first two months before it stabilized, whereas the *V*_OC_ gradually decreased as the outdoor exposure time proceeded. The overall device efficiency hardly changed. Another publication involving module stability after 2010 is the work from Hinsch *et al.*^842^ They present impressive DSC demonstrators with size 60 × 100 cm. However, the stability results (1000 h at 85 °C in darkness) are obtained by a device size of 100 cm^2^ ( Table 13 ).

It stands clear that the number of publications dealing with DSC module stability in the past 10 years has decreased in relation to the period 2005–2010. We were quite surprised to find a lack of published stability data from the semi-transparent BIPV demonstrators that have been realized around the world (see Section 7.3.2) and the shortage of recent field tests comparing DSC modules with other PV technologies. Likewise, we have not found any recent papers about the stability of low-power DSC modules, likely explained by the fact that this work is carried out by industry where the driving force for publication is low. In addition, Pettersson *et al.* already in 2001 showed that DSC modules can be very stable under such conditions by demonstrating a mere 4% decrease of the initial performance of a DSC device after half a year of illumination with a fluorescent light (5000 lx).^843^

### 7.3 Application categories and commercialization efforts

Despite the different nature of commercialization initiatives performed over the past 30 years, there are few main product categories that can be identified. As a consequence of this, we have divided the targeted applications for DSC into three categories: (i) panels to challenge Si, (ii) BIPV *via* aesthetic devices, and (iii) niche products for electronic applications. The evolution of each category and their status are discussed below.

#### 7.3.1 Challenge Si

In the nineties, solar cells were still treated as a highly interesting energy source for the future. Even though there was a rapidly increasing amount on photovoltaic installations, they originated from a low level. In addition, most installations were the results of various national programs. The German so-called 1000-roof program (1990–1994) was followed by *e.g.* the Japanese Residential Roofs Program (1994–1995). However, it was the German 100 000 Roof Program in 1999 that dramatically changed the market for photovoltaics. All of this was realized under the assumption that silicon solar cells would face difficulties in reaching manufacturing costs that would make it competitive with conventional energy sources; *i.e.* there was a need for novel photovoltaic technologies with lower production costs. The leading technologies from this aspect were thin-film PV such as CIS, CIGS and CdTe. Whereas these technologies were targeting high efficiencies and advanced manufacturing processes, characterized by massive investment costs, DSC entered the field from a totally different and unexpected angle, characterized by lower efficiency but basic manufacturing processes and low-cost, scalable raw materials. The investment costs for initiating a DSC production line were foreseen to be a fraction compared to silicon or thin-film technologies. As a result of all of this, DSC attracted many companies that wanted to take on the challenge to commercialize the technology. Moreover, it was a possibility for companies that were not active in the photovoltaic industry to enter the field. As a result of all of this, almost all industrial DSC efforts during 1990–2005 targeted the future global massive PV market. In their 2010 review, Hagfeldt *et al.* presented a number of DSC device examples from this period that were driven by the target of challenging silicon.^8^ In the ten-year period 2005–2015, the manufacturing costs of silicon solar cells decreased as a result of the massive Chinese commercialization activities. The previous dream target of manufacturing costs of 1 USD per *W*_peak_ was suddenly dramatically undercut. As a result of this, more emphasis was given to the increase of device efficiency. Consequently, the arguments for DSC as a candidate for future large-scale photovoltaic establishments disappeared, as dramatic efficiency improvements were now required. Even though this coincided with the DSC efficiency breakthroughs from Feldt *et al.*^270^ and Yella *et al.*,^284^ the entrance of the perovskite technology in 2012 changed the prerequisites for DSCs overnight.^844,845^ The perovskite technology shared the basic features of DSC, namely cost-efficient scalable manufacturing methods and material components. Even the recent DSC record efficiency of 13.0% in year 2021^12^ is still low compared to those obtained by perovskite solar cells, with a present efficiency record of 25.2%.^846^ As a result of all this, there are today very few industrial DSC initiatives targeted at challenging silicon PV. In order to change this situation, a significant fundamental scientific breakthrough is required, opening for massive efficiency improvements. Nevertheless, the collective industrial and academic efforts devoted to developing competitive DSC devices for outdoor applications have left important technology testimonies such as module and production technology, proven durability at outdoor conditions, life cycle^847^ and cost analyses.^848^ In fact, this collective output has dramatically influenced the development of DSC for BIPV (Section 7.3.2) and low-power applications (Section 7.3.3), as well as the entire perovskite technology.

#### 7.3.2 BIPV *via* aesthetic devices

**Fig. 54:**
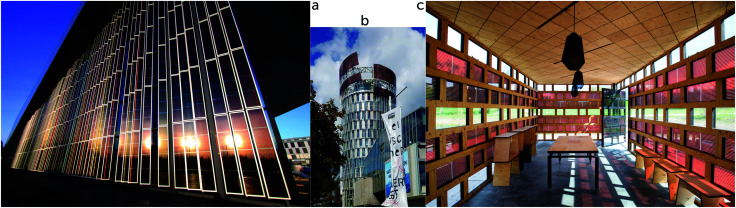
(a) The DSC installation at the Conference centre in Lausanne, Switerland, consisting of 1400 W-connected modules of the size 35 × 50 cm^2^ (in total approx. 150 m^2^), manufactured by Solaronix in Switzerland. Reproduced with permission from Solaronix S.A., copyright 2021. (b) The DSC installation at the Science Tower in Graz, Austria, consisting of 896 W-connected red DSC devices of 0.6 m^2^ area each (in total approx. 500 m^2^), manufactured by H.Glass in Switzerland. Reproduced with permission from H.Glass S.A., copyright 2021. (c) The DSC installation at the Solar Pavillon at Roskilde University in Denmark, consisting of 196 W-connected red DSC panels of area 900 cm^2^ each (in total approx. 180 m^2^) made by Dongjin Semichem in South Korea. Architect Jane Ostermann-Petersen. Reproduced with permission from Karina Tengberg, copyright 2021.

The aesthetic properties of the DSC technology have been known since the beginning. The fact that dye molecules have a key role immediately started discussions regarding colourful devices in one or several colors, in both opaque and see-through variations. However, the activities for these applications were initially minor in relation to the hunt for a low-cost DSC solar cell technology to challenge silicon photovoltaics. During the past ten years, however, see-through DSC panels in various colors for BIPV applications have been increasingly investigated by various companies. One of the early publications in the field was from Sastrawan *et al.*, who in the year 2006 displayed red semi-transparent DSC modules.^849^ Examples of early industrial initiatives to develop aesthetic see-through DSC for BIPV applications came from TDK, Samsung, Dongjin Semichem, Dyesol, Peccell, Aisin Seiki and Toyota. Despite many impressive prototypes, the milestone for aesthetic DSC panels occurred in 2014 when the novel Conference centre at EPFL in Lausanne was inaugurated, containing a see-through wall of DSC modules in five different colors: light red, dark red, light green, dark green and orange. In total, 1400 modules of the size 35 × 50 cm^2^ have been produced and installed at the Conference centre by Solaronix in Switzerland, Fig. 54a. The installation is impressive and displays the attractive architectural features of DSC. However, from visual inspections at the site, it stands clear that many modules have experienced various degradation modes, such as leakage, electrophoresis, chemical reactions between current collectors and electrolyte, and vertical electrolyte concentration gradients, likely caused by the formation of polyiodide chains. The EPFL installation was followed by a range of aesthetic installations from H.Glass in Switzerland (originally glass2energy). Their most impressive installation is the Science Tower in Graz, Austria, where 896 red DSC devices (each 0.6 m^2^) are placed on top of the 60 m tall building, Fig. 54b. Another DSC see-through installation deserving attention is the Solar Pavillon at Roskilde University in Denmark (Fig. 54c). The 196 DSC panels (each 900 cm^2^), made by Dongjin Semichem, are integrated directly into the pavilion's glass facade constituting the basic element of its architectural motive, and providing charge stations for mobile phones and tablets to visitors. Further examples of intense industrial development of similar see-through DSC devices came from the Dyepower consortium in Italy. In 2015, they reported an active area conversion efficiency of 5.6% on a Z-connected 600 cm^2^ device realized in their pilot line facility.^850^ In addition, these devices successfully passed the UV preconditioning test, the humidity freeze test and the damp heat test of the IEC 61646 Standard. The Dyepower consortium also performed a thorough evaluation of the environmental profile of semi-transparent DSC.^851^

**Fig. 55:**
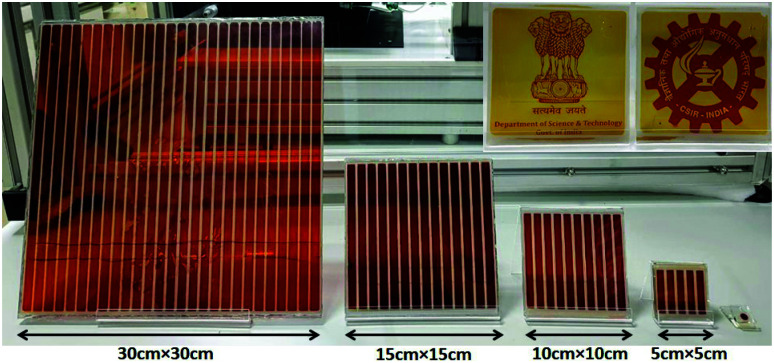
Indian semi-transparent DSC prototypes from Elixir Technologies and CSIR-National Institute for Interdisciplinary Science & Technology (NIIST). Reproduced with permission from the Indian Ministry of Science and Technology, copyright 2021.

All of the aforementioned initiatives were foreseen to represent the commercial breakthrough of aesthetic DSCs for BIPV applications. However, this has not been realized. On the contrary, the industrial activities on see-through aesthetic DSCs seem to have decreased in the past 2–3 years. A tentative explanation for this is that the energy production, *i.e.* the device efficiencies, were too low to balance the additional cost compared to coloured glass or alternative architectural features, potentially in combination with question marks regarding the product life. However, other similar initiatives are still ongoing, such as the Indian collaboration between Elixir Technologies and CSIR-National Institute for Interdisciplinary Science & Technology (NIIST) (Fig. 55).

All devices in Fig. 54a and b use a module idea based on W-interconnects, *i.e.* the double-substrate module design contains cells with alternating working and counter electrodes on each substrate. As a consequence, every second cell is irradiated from the counter electrode side, which generally leads to lower current values than irradiation from the working electrode side. A challenge involved is thus to match the current output from adjacent inverted cells. This has commonly been overcome by making the cells illuminated through the counter electrode slightly broader, *i.e.* a larger active area to compensate for the lower current output. One drawback of this solution is that the ratio of current output from front- and back-side illumination varies with light intensity and illumination angle. Moreover, as semi-transparent devices are illuminated from both sides, the illumination conditions are complicated and unpredictable. Consequently, it is practically impossible to avoid an imbalance in current output between cells. Such imbalance will decrease the overall device performance but it may also result in performance degradation over time. Interestingly enough, we have not found any literature on *e.g.* the device chemistry and/or the delivered energy values from these installations. This is surprising and unfortunate as these installations would provide highly interesting results and information ranging from device performance to potential degradation modes over time.

#### 7.3.3 Niche products for electronic applications

As for aesthetic devices, the low-light properties of the DSC technology have been known since the beginning. The nanostructured working electrode efficiently absorbs diffused light, making it an ideal candidate for low-power devices. Two industrial pioneers in the fields were the Swiss companies Asulab and Leclanché, which already in the mid-nineties were active in prototyping DSC devices for watch-making applications and various electronic gadgets, respectively. Papageorgiou *et al.*,^852^ Pettersson *et al.*^853^ and S. Burnside *et al.*^854^ are all examples of early papers regarding material components, cell and modules performance, long-term stability and manufacturing methods for low-power DSCs. Recently, Kokkonen *et al.* reviewed all these aspects with artificial light applications in mind.^855^

**Fig. 56:**
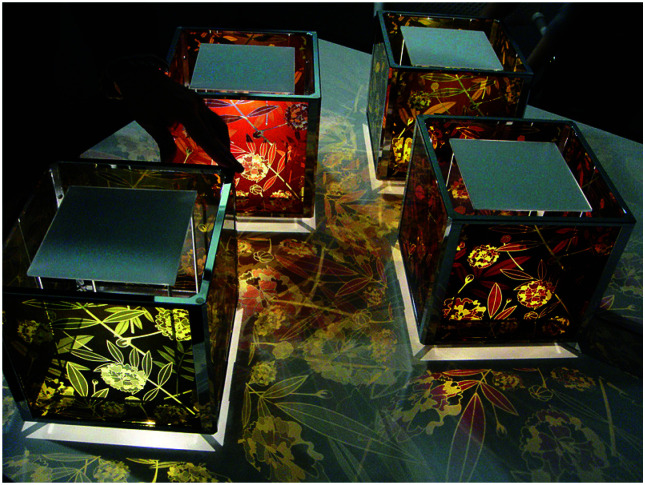
An example of artistic DSC devices from Sony displayed at the 10th Eco-Products Conference in Tokyo in 2008. Reproduced with permission from Satoshi Uchida, copyright 2021.

Around the beginning of the millennium, activities on flexible DSC were taking off. Companies such as Konarka Technologies, USA, and Sekisui Chemical, Taiyo Yuden Co. and Peccell Technologies, Japan, developed such technologies. The DSC technology of Konarka was a few years later taken over by G24 Innovations (later G24 Power), who initiated a massive effort to commercialize the technology for low-power applications. Their factory in Wales is generally considered as the first large-scale mass production facility for DSC. Various products, such as Logitech keyboards, solar backpacks, solar chargers and solar iBeacons were launched. Whereas G24 targeted large-volume production for broad applications, there were several parallel Japanese initiatives where DSCs were used in solar art demonstrators, *e.g.* aesthetic devices powering lamps and fans. The lamp charger Hana-Akiri from Sony received a lot of attention, Fig. 56. Similar artistic DSC devices from the same period came from *e.g.* J Touch Co., Aisin Seiki and Nissha Printing. Retrospectively, it can be concluded that all of these, and many other low-power DSC commercial initiatives in the period 2000–2010, did not trigger a sustainable market demand.

The arguments for indoor low-power DSC received novel fuel from the work of Feldt *et al.*,^270^ where it stood clear that the combination of organic dyes and one-electron Co-based redox mediators resulted in major performance improvements, with high voltage levels even at low light conditions. In addition, low-power PV became of interest as a result of the increased global activities on IoT applications with forecast billions of small systems requiring low-power supply. As a result, there has been a revival for and a rapid increase in industrial initiatives targeting low-power DSC. The interest for low-power DSC was taken to the next level by the work of Freitag *et al.*^348^ By using Cu-based one-electron redox mediators in combination with organic dyes, low-power efficiencies of 28.9% were obtained at 1000 lux. This was followed up by a 32% cell efficiency at 1000 lux by Cao *et al.*,^320^ a 34% cell efficiency at 1000 lux by Michaels *et al.*,^26^ and a 34.5% cell efficiency at 1000 lux by Zhang *et al.*^12^ Interestingly enough, all these pieces of work used the same illumination source (Osram 930 Warm White fluorescent light). However, we highlighted above that characterization of low-power devices is a somewhat confusing part of the PV world since there is no established standard for the illumination and caution should be taken when comparing values (see Section 2.2).^53,856^ An interesting comparison to low-power perovskite solar cells, however, can be made by the values reported by Meng Li *et al.*^857^ They achieved conversion efficiencies up to 35.2% at a device size of 9 mm^2^ (23.2% at 4 cm^2^) and 1000 lux using a fluorescent light source (Osram L18W/82). In contrast to the DSC values from Michaels *et al.*,^26^ the efficiencies for the perovskite devices were dramatically reduced at lower light intensit ies: 25.7% and 19.5% efficiencies were obtained at 500 and 100 lux, respectively. These perovskite devices include lead, which may be a limitation for commercial exploitation in electronic applications. In addition to DSC and perovskite solar cells, organic solar cells (OPV) represent an additional technology candidate for low-power applications, with confirmed efficiency values up to 28.1% at 1000 lux.^858^ It is thus a product segment that is becoming crowded by various upcoming technologies. From a strict efficiency point of view, it appears that DSC devices deliver the highest efficiency values at indoor illumination, at least at 500 lux and 100 lux, and at 1000 lux for device size d >1 cm^2^. This gives companies commercializing low-power DSC the prerequisites to realize the best-performing low-power products. In the commercial race, however, other additional selling points other than indoor efficiency will likely be important, such as price, colour, weight, thickness and flexibility in size and voltage.

**Fig. 57:**
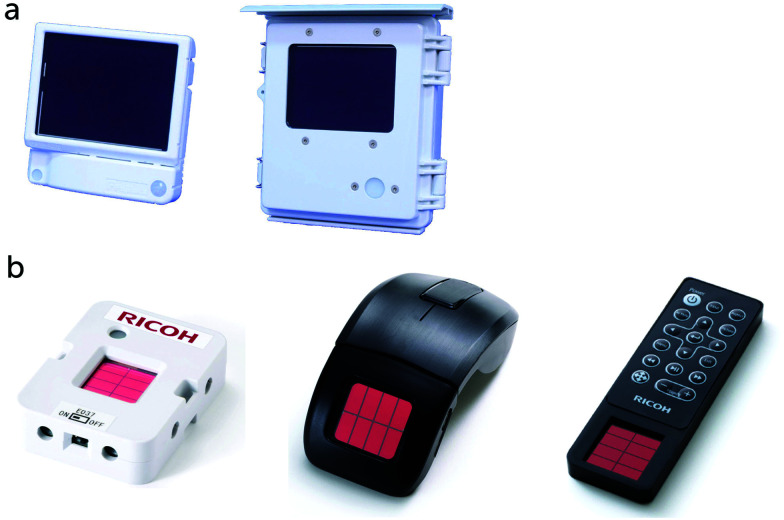
(a) DSC-containing sensor systems from Fujikura in Japan for indoor (left) and outdoor (right) applications, respectively. Reproduced with permission from Fujikura Ltd, copyright 2021. (b) Examples of products from Ricoh containing their solid-state DSC devices: environmental sensors for measuring temperature, humidity, illumination, atmospheric pressure, *etc.*, wireless mouse and remote controls for projectors. Reproduced with permission from Ricoh Company Ltd, copyright 2021.

The new era of DSC industrialization for niche applications in general, and low-power devices in particular, is confirmed by recent product launches. The DSCs of Fujikura in Japan are already used in wireless multi-sensor device systems such as heatstroke prevention systems and management of large warehouses in Japan, Fig. 57a.^859^ 3GSolar in Israel introduced several DSC options with different transparency and colors to fit many diverse niche applications, including wireless sensor networks, medical and sports devices, security sensors and cameras, agricultural monitors, beacons and electronic signs, computer peripherals, and wearable electronics. Exeger in Sweden has announced that their DSC devices will be used in various consumer electronics devices such as headphones, safe helmets and soft goods. In 2020, Ricoh in Japan launched their solid-state RICOH EH DSC series. These devices are used in applications such as remote controls for projectors and to power IoT sensor systems, Fig. 57b.

**Fig. 58:**
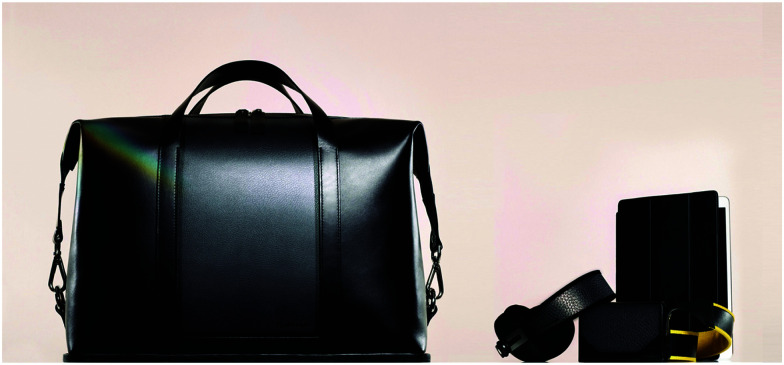
Various prototypes including non-visible DSC devices from Exeger in Sweden. Reproduced with permission from Exeger A.B., copyright 2021.

Out of these DSC products, it is noticeable that Fujikura has different devices for outdoor and indoor use (Fig. 57a). This is likely attributed to the fact that Fujikura worked on outdoor DSC module development before focusing on low-power devices, *i.e.* they had access to the required chemistry and manufacturing methods for outdoor applications.^839^ Ricoh appears to be the only producer using solid-state DSCs. Moreover, it is worth noticing that devices from Exeger are marketed as solar cells that are integrated without being seen, Fig. 58, opening for their vision to implement their light harvesting cells on all imaginary surfaces ranging from electronic gadgets to buildings *via e.g.* blinds, walls, vehicles, bags and furniture.

An unexpected side effect of low-power DSC development is the technology Focus-Induced Photoresponse (FIP technique). This technology is based on the discovery that the power output from a DSC is not only dependent on the total flux of incident photons, but also on the size of the area in which they fall. Consequently, when probe light from an object is cast on a detector through a lens, the sensor response depends on how far in or out of focus the object is, *i.e.* a novel way to measure distances with photodetectors.^860^ The technology was invented and commercialized by the company Trinamix in Germany, a wholly owned subsidiary of BASF.

## 8 Outlook: Colourful

Every significant advance over the previous decade in the development of DSCs has been made by the introduction of new principles, techniques, and materials. DSCs are becoming part of the future of electric power generation due to the following characteristics: (i) they are easy to fabricate, (ii) they are manufactured from low-cost materials, (iii) they are environmentally friendly, (iv) they have high conversion efficiencies, and (v) they perform well in diffused light and at high temperatures, conditions in which other technologies cannot compete. Based on creative research work, power conversion efficiencies of up to 20% under sunlight and 45% for ambient light can be anticipated from future DSCs.

Detailed understanding of many aspects of the dye-sensitized solar cell is still lacking. Charge recombination is currently the major cause of efficiency loss in DSCs and other solar cells. When one of the components (dye, redox shuttle, or semiconductor) is modified, many processes are impacted, which may boost or lower the overall performance. This needs to be considered at all times when new materials are introduced, and the overall system has to be adapted. DSCs are complex devices and the improvement of only one of their components will not lead to the desired targets in efficiency and stability.

### Theory and computation

From the computational perspective, new theoretical tools are needed to push forward our understanding of DSCs beyond the established, successful applications outlined above. Fortunately, thanks to continuously increasing computer power and new computational paradigms, this is the right time for such developments. *In silico* design and optimization of materials will need to shift from single components to coupled dye/electrode or, ideally, electrode/dye/electrolyte ensembles. New algorithms based on artificial intelligence and machine learning fit this purpose, with training databases obtained from high-throughput computations. Still, the results of such automated discoveries will need to be validated with the magnifying glass of atomistic first-principles calculations, able to dissect electronic and dynamic properties beyond the ideal picture of interfaces considered so far. In particular, we foresee a crucial role of studies addressing defects and additives that can be game changers for reaching desired efficiencies and, regarding processes, charge transfer and recombination events under operating conditions. These advancements in models and methods will bridge the gap between theory and experiments, so that computer and laboratory bench can jointly tackle the design and optimization of new DSCs.

### Materials

High efficiency and panchromatic organic dye systems have been developed. These are a non toxic, low cost, sustainable, and conveniently accessible option. The next step will be to achieve a fundamental understanding of electron injection from the dye in its excited state into the conduction band of the semiconductor, in order to minimize potential and overall conversion efficiency losses at this interface. The semiconductor requires a modification of the position and of the nature of its conduction band, which can be reached through doping, morphology variation or the use of alternatives to TiO_2_. The dyes' LUMO level should be tuned to match the potential of the conduction band edge of the semiconductor closely to provide efficient electron injection and minimize energy losses.

In a more idealistic direction, DSCs could significantly benefit from the design of a photoinduced molecular rectification strategy built into the chromophore design. The idea of a facile electron transfer to the semiconductor with the cation trapped away from the surface for extended time could ease demands on the rate of dye regeneration by slowing down the competitive back reaction, which could lead to high fill factors thanks to an increase in regeneration efficiency at the maximum power point. The D–π–A dye design is a simple example of this approach that revolutionized the DSC field. If new designs with dramatically higher rectification effects retaining near unity quantum yields for electron injection could be put forward, another revolution within DSCs could be induced, leading to another massive gain in power conversion efficiencies.

Another consideration is the position and packing of molecules on the semiconductor surface, as well as how these factors influence electron transfer kinetics in DSCs. With examples of dyes having exceptionally low recombination losses and exceptionally high conversion efficiencies in devices operating with absorption onsets up to 700 nm in mind, several key directions remain important with regard to DSC dye design. The utilization of photons with >800 nm wavelength with the same efficiency as is observed at 700 nm is another target of the DSC field, with maximal single photoelectrode devices expected to peak at absorption onsets of 950 nm. Additionally, tandem type systems require new chromophores at both high and low energy absorption onsets (high voltage dyes and NIR dyes) paired with appropriate redox shuttles for devices where dye energy levels are well positioned to minimize energy losses. The development of these systems is key for DSCs to exceed the single photoelectrode Shockley–Queisser limit. DSCs have shown exceptional photovoltage outputs from higher energy visible light photons, and the design of dyes maximizing performance in the blue spectral region and of more positive potential redox shuttle systems could be transformative in providing tandem systems to be paired with any smaller-bandgap solar cell technology. The development of one-electron redox shuttles with high performances with transition metal-based sensitizers could provide a needed answer to the lower energy absorption onset challenge, since good sensitizer options already exist but are incompatible with most redox shuttle systems. Furthermore, electron transport in mesoporous semiconductor electrodes is normally described in terms of multiple trapping/detrapping, but the nature of the traps involved is unclear. It has been suggested that the electrostatic interaction between electrons in the semiconductor and ions in the electrolyte could in fact be the origin of such traps.

Future research should further concentrate on electrolyte interactions with electrodes and sensitized dyes, as well as on the impact of these interactions on photoelectrical conversion processes, and on the creation of alternative charge carrier materials to increase charge carriers' transport performance, minimize recombination losses, and improve long-term stability. Another factor to consider in these systems is the replacement of the liquid electrolyte with a solid-state electrolyte or charge transport material to avoid leakage, solvent volatilization, dye photodegradation and desorption, and counter electrode corrosion. This goal has been partially reached thanks to the introduction of metal coordination complexes, but their development is still far behind the efforts made in dye development.

### p-type DSCs

Much of the improvement in performance for p-type DSCs has arisen from developments in dyes and new electrolytes. In order to reach efficiencies that compete with thin-film solar cells, the *V*_OC_ needs to be improved by *ca.* 0.5 V to match that of typical n-type DSCs. This requires a replacement for NiO which is transparent, conductive, stable and non-toxic. There are very few single materials with all of these properties. Moreover, there are still gaps in our understanding of electron transfer at the interface of p-type metal oxides and dye molecules. Currently, beyond NiO itself, it is not clear what the limitations to p-type DSCs are, but so far, there has been a trade-off between current and voltage that needs to be understood for progress to be made. To realize the potential of p–n tandem DSCs, a concerted effort of materials development combined with state-of-the-art spectroscopy is necessary. Meanwhile, very few examples of solid-state p-type DSCs have been reported and this is a rich area for future development that may overcome some of the challenges associated with liquid cells. Moreover, the factors that limit the performance of solid state DSCs, such as the requirement for thin semiconductor films, may be less limiting in solid-state tandem DSCs.

### Solar fuels

Most DSPEC studies to date have been carried out at pH values between 4.5 and 8.0, where the injection efficiency of the most commonly used chromophores into the conduction band of wide bandgap semiconductors such as TiO_2_ is below 50%. In addition, stability of catalysts, chromophores and anchors also decrease as the pH is increased. There are opportunities for significant improvements in DSPEC performance and stability at low pH (*e.g.* pH 1) where injection efficiencies are close to 100%. Most DSPEC require an applied bias for efficient H_2_ generation and release. Combining DSPECs and DSCs will eliminate the need for an applied bias and open the door for CO_2_ reduction photocathodes which typically operate at larger overpotentials than proton reduction photocathodes.

### Applications

The high sensitivity and efficiency of DSCs in low and ambient light conditions is one of their major benefits. They can be used where diffused solar light prevails over direct solar illumination. For this reason, the essential use of DSCs in building windows is that they operate well not just on the roof, as is the case with direct solar light irradiation in silicon cells. In the light of the global energy report, this advantage of the DSC would also reduce the energy usage represented by buildings. This industry is a major contributor to greenhouse emissions, consuming between 34% and 39% of electricity worldwide. The colors that DSCs can implement are another appealing feature for businesses. DSCs can be used as thin colored and transparent panels, transforming typical walls, skylights, and glass facades into electricity generators.

With continued research, it is certain that more interesting features will be revealed that could lead to improved performance of DSCs or to spin-off applications. The aforementioned directions are currently being pursued by researchers and exciting results are expected.

## Author contributions

ABMG and MP wrote Section 3. IB wrote Section 2.2, edited the manuscript, compiled tables and drew molecular structures. GB wrote Sections 2.1, 2.3, 2.4, 2.5, 4.1 and 7.2. JJC wrote Section 6. JHD wrote Section 4.2. EAG wrote Section 5. GJM wrote Section 2.6. HP wrote Section 7. AH wrote Section 1. MF wrote Sections 4.3 and 4.4., conceptualized and oversaw the completion of the article. All authors contributed to the writing of the outlook, substantially contributed to the conception and design of the article by interpreting the relevant literature, and contributed to the critical revision and review of the manuscript.

## Conflicts of interest

GB, HP and AH are co-founders and co-owners of Dyenamo AB.

## Supplementary Material
